# Differences in X-Chromosome Transcriptional Activity and Cholesterol Metabolism between Placentae from Swine Breeds from Asian and Western Origins

**DOI:** 10.1371/journal.pone.0055345

**Published:** 2013-01-31

**Authors:** Steve R. Bischoff, Shengdar Q. Tsai, Nicholas E. Hardison, Alison A. Motsinger-Reif, Bradley A. Freking, Dan J. Nonneman, Gary A. Rohrer, Jorge A. Piedrahita

**Affiliations:** 1 Department of Molecular Biomedical Sciences, College of Veterinary Medicine, North Carolina State University, Raleigh, North Carolina, United States of America; 2 USDA, ARS, U.S. Meat Animal Research Center, Clay Center, Nebraska, United States of America; 3 Center for Comparative Medicine and Translational Research, North Carolina State University, Raleigh, North Carolina, United States of America; 4 Program in Statistical Genetics, Department of Statistics, North Carolina State University, Raleigh, North Carolina, United States of America; Huazhong Agricultural University, China

## Abstract

To gain insight into differences in placental physiology between two swine breeds noted for their dissimilar reproductive performance, that is, the Chinese Meishan and white composite (WC), we examined gene expression profiles of placental tissues collected at 25, 45, 65, 85, and 105 days of gestation by microarrays. Using a linear mixed model, a total of 1,595 differentially expressed genes were identified between the two pig breeds using a false-discovery rate q-value ≤0.05. Among these genes, we identified breed-specific isoforms of XIST, a long non-coding RNA responsible X-chromosome dosage compensation in females. Additionally, we explored the interaction of placental gene expression and chromosomal location by DIGMAP and identified three Sus scrofa X chromosomal bands (Xq13, Xq21, Xp11) that represent transcriptionally active clusters that differ between Meishan and WC during placental development. Also, pathway analysis identified fundamental breed differences in placental cholesterol trafficking and its synthesis. Direct measurement of cholesterol confirmed that the cholesterol content was significantly higher in the Meishan versus WC placentae. Taken together, this work identifies key metabolic pathways that differ in the placentae of two swine breeds noted for differences in reproductive prolificacy.

## Introduction

The placenta serves as a critical transport organ between the developing fetus and mother to regulate nutrient exchange, excretion of waste, oxygen and hormones [1]. Interactions among transcriptional/epigenetic circuits and environmental cues influence intrauterine growth and may lead to aberrant physiological programs in the adult through fetal programming [2]. Dissecting trophoblast physiology pathways by functional genomic tools could help to clarify how the fetus is sensitized to environmental inputs, such as undernutrition or uterine crowding, and alleviate pregnancy complications and in utero programming of adult diseases.

Due to its simplicity, the swine placenta provides an excellent model to study some of the fundamental factors that affect maternal-fetal-placental function [3]. The porcine placenta consists of an epithelial bilayer with no active invasion into the maternal uterine stroma and is classified as a diffuse epitheliochorial [4]. The placenta forms the maternal-fetal transport interface and sensitizes the developing fetus to environmental perturbations; indeed, pregnancies irrespective of identical genetic background, e.g. same mother, can significantly vary by litter size, fetal birth weights and placental weights. When compared to commercial western breeds of pigs such as the white composite breed (WC), the Chinese Meishans farrow three to five more piglets per litter, and this enhanced prolificacy has been attributed to major differences in placental morphology and physiology [5], [6]. Increased placental vascularization and reduced uterine surface area, are thought to account for increased nutrient exchange to the Meishan fetus, and is predicted to yield larger litter sizes, albeit with lower birth weights [7]. Thus, both its simplicity and the existence of breed-to-breed variation provide a unique tool to examine how gene expression profiles relate to breed-specific placental function.

Additionally, improvements in swine reproductive fitness can impact food production as the incidence of stillborn, growth-restriction and postnatal morbidity limits fecundity and raises agribusiness costs [8]. Major losses during swine embryonic development primarily occur prior to day 40 of gestation [9], [10]. Genetic selection strategies have identified rate-limiting determinants for maximizing number of piglets, which include ovulation rate, fertilization rate, pre-implantation embryonic survival, placental efficiency and post-natal health [11], [12]. Enhancement of ovulation rate reduces early embryo viability attributed to uterine crowding and low egg quality [13]. Furthermore, breeding schemes that select sows with larger litters result in low-birth weight piglets and higher incidence of postnatal mortality [14].

Building on our previous studies [15], we surveyed differential placental gene expression between White Composite (WC) and Meishan (MS) breeds throughout gestation at 20-day intervals (days 25, 45, 65, 85 and 105) to identify historic breed differences throughout the gestational period. Using functional genomics classification tools, we identify cholesterol biosynthesis and transport as major functional pathways that differ in the placentae of each breed. Furthermore, we present an intriguing molecular phenotype between breed placentae by mapping transcriptionally active clusters across the X-chromosome and RNA structural differences in *XIST*.

## Materials and Methods

### 1 Breed Description

A four-breed composite population, namely white composite (WC), derived from maternal lines consisting of 1/4 Yorkshire, 1/4 Landrace, 1/4 Large White, and 1/4 Chester White breeds was used to provide placental tissue. This population averaged 9 piglets per litter and birth weights of 1117 g from gilt matings. Reproductive performance of the WC USDA-MARC population used in this study has been described previously by Cassady and colleagues [16]. Noted for their enhanced fecundity, Meishans as well as Fenjing, Jiaxing-Black and Erhualian are derived from the Taihu strain and are native to the Yangtze River basin. The USDA obtained Meishan germplasm in 1989 as a gift from the Chinese government and remains as a restricted bioresource due to its status as a natural treasure [17]. Reproductive performance of Meishans (MS) obtained from this germplasm has been summarized previously [17], [18]. At approximately 90 days, MS become sexually mature; gilts farrow 14–17 piglets on average, and birth weights average 900 g [17]. All animal tissues used for these studies were derived from cohorts maintained at ARS-USDA-MARC and described in the aforementioned references.

### 2 Experimental Design

To determine overall breed differences independent of gestational age, each breed was sampled at five different time points (D25, D45, D65, D85 and D105) with three biological replicates per time point, for a total of fifteen replications per breed. Biological replicates consisted of three randomly selected female placentas from each pregnancy. The time points were selected to cover all periods of gestation starting from D25 when the placenta is fully formed. This design allowed us to look at overall breed differences independent of stage of gestation, as well as temporal differences. Additionally, fetuses were sexed either visually (D65, D85, and D105) or by PCR (D25 and D45) using primers to *SRY* or X-specific *AMELX* or Y-specific *AMELY* [19]. Females were chosen with the exception of one male sample at D65_MS_B (GEO accession GSM264145) due to sample limitations (only two females in the D65 litter). The choice of females allows closer examination of X-inactivation as well as comparisons with a previously generated female-only dataset (8).

### 3 Fetal Tissue Collection and RNA Isolation

Briefly, naturally mated WC or Meishan gilts were sacrificed to collect fetal tissues at 20-day gestational intervals including days 25, 45, 65, 85 and 105 (D25, D45, D65, D85, D105) at the USMARC abattoir according to USDA regulations. The WC placental samples were derived from control line gilts in a serial slaughter experiment as described in Freking et al. 2007 [20]. Meishan gilts were matched to the same slaughter ages represented. The Meishan gilts were housed and reared separately in similar breeding and gestation pen facilities and were fed similar diets. For sampling consistency, sections of 2×2 cm^2^ chorioallantoic (placental) tissues were dissected cleanly away from maternal endometrium or fetal amnion. Biopsied placental tissues were sourced dorsal to the fetal amnion, harvested within 5–8 minutes, flash-frozen in liquid nitrogen, and stored at −80°C until further processing. Handling of animals complied with the procedures as specified in [21]. Animal protocols were approved by the Institutional Animal Care & Use Committee at North Carolina State University and the USMARC-ARS-USDA. The procurement, care, and use of animals were in accordance with the regulations and terms of the federal Animal Welfare Act and the Health Research Extension Act of 1985, and subsequent revisions. All research projects and educational or extension activities using vertebrate animals under the jurisdiction or control of NCSU are reviewed and approved by the Institutional Animal Care and Use Committee (IACUC).

Frozen tissues were pulverized by mortar and pestle in preparation for RNA extraction. After tissue disruption, total chorioallantoic RNA from both WC and Meishan animals were isolated according to a commercial kit with minor modifications (RNAqueous kit, Ambion, Austin, TX). Briefly, 100 mg pulverized tissue was immediately added to 1.2 ml RNA lysis and stabilization buffer [4 M LiCl, 5% Triton-X100, 5% DGME, 10 mM EDTA, 50 mM TCEP, 1% Na_2_WO_4_, 100 mM HEPES at pH 8.8] (W509043, DMGE; Sigma, St. Louis, MO) contained the sulfhydryl reductant tris-2-carboxyethyl phosphine 50 mM (TCEP; PolyOrganix, Houston, TX; ) in lieu of dithiothreitol [22], and acid phenol:BCP (B9673, Sigma, St. Louis, MO) extraction was omitted from all isolation steps. Total RNA was selectively precipitated with 6 M LiCl and 10 microgram total RNA aliquots were stored in 1 mM sodium citrate, pH 6.4 at −80°C to preserve integrity until microarray hybridization or quantitative real-time reverse-transcription PCR (RT-qPCR). Quantitation by UV-spectrophotometry of A260/280 ratios, an indicator of RNA purity, generally exceeded 1.90, and A260/230 ratios (organic contamination) were generally greater than 2.0 as gauged by NanoDrop ND-1000 spectrophotometer (NanoDrop Technologies, Wilmington, DE). RNA quality was judged by ribosomal banding 28∶18 Svedberg ratios from denaturing 1% agarose lithium acetate gels or RNA integrity scores (RIN) of 9 or better using a commercial chip analyzer (RNA Lab-on-a-chip, Agilent 2100 BioAnalyzer).

### 4. Microarray Analyses

#### 4.1 In vitro transcription and hybridization to affymetrix porcine GeneChip

A detailed description of in vitro transcription to produce cRNA and its hybridization to short-oligonucleotide arrays (900623, Porcine GeneChip, Affymetrix, Santa Clara, CA) is previously described in Bischoff et al, 2008 [15]. The array contains 23,937 probe sets that interrogate approximately 23,256 transcripts from 20,201 Sus scrofa genes. The data discussed in this publication have been deposited in NCBI’s Gene Expression Omnibus (GEO) [23], and the Affymetrix Porcine GeneChip *.cel files are accessible through GEO Series accession numbers GSE10446, GSE10447. Datasets used in this publication are compliant with the standards adopted by the MIAME consortium for reporting microarray datasets.

#### 4.2. Statistical modeling of gene expression

Minimal normalization was performed using a linear-mixed model normalization procedure [24], [25] to essentially re-center the mean intensity of each expression array. Log_2_-transformed perfect-match (PM) intensities for all observations were fit to a linear mixed model [24], [25]. A gene-specific mixed model was fit to the normalized intensities (residuals from first model) accounting for fixed breed, probe, and breed-by-probe interaction effects and a random array effect. A description of fixed and random effects is described elsewhere [25], [26]. To discover the magnitude and significance of differential expression between pig breeds at the transcript level, we implemented JMP Genomics 5.0 (SAS, Cary, NC) using analysis of variance (ANOVA), e.g. PROC MIXED as implemented in SAS, while correcting for multiple tests and adjusting for covariates and random effects [27]. A normal distribution of the random error ε is assumed with its center at zero. Specifically, differential expression was determined by the following ANOVA model using JMP Genomics 5.0:
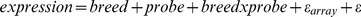



We used a perfect-match only gene-by-gene model, as some reports indicated that incorporating the mismatch probes increases noisiness of the data when estimating differential expression [28], [29]. JMP 5.0 software was executed according to the default settings described by the version 5 software workflow to calculate estimate statements for breed comparisons using all thirty arrays.

To correct for multiple testing, we implemented Storey’s procedure [30], [31] by conversion of p-values from linear-mixed model procedures to q-values using QVALUE (software downloaded from [32]. Comparisons between treatment group (breed) for differential gene expression were made based on the following criteria: 1) statistical cut-off of q-value <0.05 for false discovery rates (FDR), and 2) a stringent presence threshold p-value <0.001 as calculated by the MAS 5.0 present/absent algorithm using the following equation:
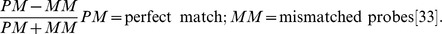



Using JMP 8.0/JMP Genomics 5 software (SAS, Cary, NC) principal component analysis (PCA) [34] was used to rapidly visualize the similarity of the placental transcriptional signatures [35] observed across the thirty arrays. Scatter plots, e.g. volcano plots of fold-change (log_2_-transformed data estimates) versus significance [–log_10_(p-values)], were constructed to rapidly identify gene expression differences in Meishan (positive) and WC (negative) placentae. We used an updated annotation of the porcine Affymetrix microarray platform as described in [36] with improved annotation to *Sus scrofa* genome build 9.2 available at reference [37].

### 5 Extraction of Endothelial Biomarkers from Array Datasets to Indirectly Assess Breed-specific Placental Vascularity Differences

In order to indirectly determine the degree of placental vascularization by examining the normalized expression level of endothelial cell markers, we compared expression of *CDH5* (VE-cadherin), *ENG* (endoglin), COLEC11 (collectin sub-family member 11), *FLT1* (FMS-like tyrosine kinase 1/vascular permeability factor receptor) and *PECAM1* (platelet endothelial cell adhesion molecule) [38], [39], all known endothelial cell biomarkers.

### 6 Validation of Microarray Data by Real-time Quantitative Reverse Transcription Polymerase Chain Reaction (RT-qPCR)

#### 6.1 Production of first-strand cDNA

Total RNA 200 ng µl^−1^ was pretreated with 3 µl (2 U µl^−1^) hypermorphic DNase I [37°C, 60′] (AM2239, Turbo DNase, Ambion/Applied Biosystems, Austin, TX). First-strand cDNA synthesis was conducted using 5 µg total RNA, oligodT_n = 20_ with slight modifications to the thermocycling parameters [42°C @10′; 50°C @ 5′; 55°C @ 5′; 42°C @ 90′] and reaction master mixes contained a thermostable, RNase H-null reverse transcriptase (600109, AffinityScript, Stratagene/Agilent, LaJolla, CA), a hypermorphic RNase inhibitor (No. AM2696, SUPERase In, Ambion, Austin, TX) was substituted in lieu of the placental ribonuclease inhibitor and addition of thermostable single-stranded binding protein (ET-SSB, H0230S, Biohelix, Beverly, MA).

#### 6.2 EvaGreen two-step RT-qPCR

To evaluate the quality of PCR primers for RT-qPCR assays, efficiency curves were generated by serial dilution (1∶3, 1∶6, 1∶9) of cDNA from the first-strand reaction, and only efficiencies ranging 95–105% were considered (data not shown). To identify candidate housekeeping genes, expression criteria included moderate to high expression, invariant across gestational time points, and ideally spanned exon-intron junctions. *RPL18* [40], [41] and *RPS20* [42] were identified as housekeeping genes based on these criteria.

A two-step master mix (No. 172–5203, SsoFast EvaGreen Supermix, BioRad, Hercules, CA) containing an enhanced double-stranded DNA fluorescent dye was chosen based on flexibility to change array target sequences and compatibility with thermocycler (iCycler® iQ, BioRad, Hercules**,** *CA*). The addition of 4 ng µl^−1^ thermostable single-stranded DNA binding protein (No. M2401S, ET-SSB, Biohelix, Beverly, MA) was added as it has been previously shown to improve PCR multiplexing and specificity. Triplicate biological samples with technical duplicates of 25 µl RT-qPCR reactions [initial denaturation 95°C for 2 minutes, (95°C @ 15″, 57°C @ 15″, 72°C @15″)_n = 40_ cycles] were run using 33 ng oligo-dT_n = 20_-primed first-strand D25, D45, D65, D85 and D105 cDNA and 500 nanomolar primers (Table S1).

A melting curve [98°C, −0.1°C second^−1^] was examined by plotting temperature on the x-axis and the derivative of EvaGreen fluorescence over temperature (−dF/dT) on the y-axis to verify correct amplification. In each case, examination of melting curves and visualization by SYBR Gold (S-11494, Molecular Probes, Eugene, OR) staining on 2% agarose 10 mM Li_2_B_4_O_7_, pH 6.5 gel electrophoresis [43] yielded RT-qPCR amplicons of representative T_m_ or product size as compared to a DNA ladder (No. N3200L, 2-log DNA ladder, New England BioLabs, Ipswich, MA). Non-template negative controls were verified as negative after 40 cycles.

#### 6.3 Statistical analysis of RT-qPCR

Reverse-transcription quantitative PCR (RT-qPCR) was employed to confirm array-based gene differential expression essentially as described in Tsai *et al* 2006 [38] using comparative C_T_ method, where fold change  = 2^−(ΔΔCT)^ [(C_T_ gene of interest−C_T_ internal control) Meishan – (C_T_ gene of interest−C_T_ internal control) WC)] [44], [45]. Established pregnancies from a single gilt per breed were used to screen placental gene expression from three littermates (three biological replicates per breed) by RT-qPCR. For each biological replicate, at least two technical replicates were used: 2 breeds ×3 biological replicates ×2 technical replicates. A two-tailed heteroscedastic (unequal variance) Student *t*-test was used to determine significance (p<0.05) and standard error was calculated from observed Ct levels per breed [46].

### 7 PCR Analysis of XIST Genomic Locus and mRNA Expression

In experiments to confirm *XIST* presence in genomic DNA and RNA isoform screens by PCR, three biological replicates per breed (genomic DNA: 3 MS, 3 WC; cDNA: 3 Meishan, 3 White Composite) were used. We used a thermostable DNA polymerase fused to the processivity factor Sso7d (Pfu:Sso7d, No. F-549L, HotStart Phusion II or No. F-122L, Phire II, New England Labs, Ipswich, MA), and thermocycling conditions were used according to the manufacturer’s protocol [47]–[49]. A list of primers (25 nmole synthesis, desalting only; Integrated DNA Technologies, Coralville, IA) used in this study and target sequence accessions is provided in Table S1.

### 8 Functional Enrichment Analysis

#### 8.1 Gene ontology analysis

Gene functional classification using DAVID [50], [51] and pathway analysis using KEGG and Ingenuity were performed as described [34], [52], [53]. To assist with the selection of gene ontology (GO) software suited for our microarray datasets, we used the freely available SerbGO [54] and identified the Database for Annotation, Visualization, and Integrated Discovery, commonly referred to as DAVID [50]. Differentially expressed genes at q-value <0.05 from breed analyses (Meishan – White Composite) were used as data input.

#### 8.2 Ingenuity pathway analysis (IPA)

Briefly, pathways from the Ingenuity library of canonical pathways that were most significant to the data set were identified. Molecules from the data set that met the q <0.05 cut-off and were associated with a canonical pathway in Ingenuity’s Knowledge Base were considered for the analysis. The significance of the association between the data set and the canonical pathway was measured in two ways: 1) a ratio of the number of molecules from the data set that map to the pathway divided by the total number of molecules that map to the canonical pathway; 2) Fisher’s exact test was used to calculate a p-value determining the probability that the association between the genes in the dataset and the canonical pathway is explained by chance alone (Ingenuity® Systems, www.ingenuity.com). A description of IPA symbols and glyphs is provided in Figure S3.

#### 8.3 Additional bioinformatics analysis tools

To better understand isoform transcript structure and gene behavior, we utilized Aceview [55] and WikiGene [56]. To facilitate mapping genes by chromosome location, we used our annotated microarray data sets with DIGMAP [57]. Briefly, Affymetrix probes were converted to chromosomal locus coordinates using the *Sus scrofa* genome build 9.2 available at Ensembl [58].

### 9 Analysis of Cholesterol Concentrations

Free and esterified cholesterol concentrations were measured by the fluorometric Amplex Red cholesterol assay (No. A12216, CAS 119171-73-2, Life Technologies, Carlsbad, CA) according to the manufacturer’s protocol with minor modifications. Briefly, ∼1 gram of frozen placental tissues were allowed to thaw on ice and then sonicated to homogeneity (3–5 pulses, 10-seconds, 800W). The placental tissues were diluted with an equal volume of phosphate buffer saline, and equal volumes of aliquots were made to analyze free cholesterol, esterified cholesterol, and bulk cellular protein. Samples were normalized according to total amount of bulk cellular protein using UV spectroscopy at 280 nm or a modified Bradford assay (No. 500-0001, Bio-Rad Protein Assay, Bio-Rad, Hercules, CA). Triplicates of controls and samples were measured at emission 590 nm for the Amplex Red assay. For control experiments, a standard curve was performed as described by the manufacturer’s protocol and a regression line was fit with adjusted R-square  = 0.989 for cholesterol concentrations ranging from 0–10 micromolar (data not shown). Samples were diluted in 1X PBS to be within the linear range of the standard curve.

## Results

### 1 Comparisons of Meishan Versus WC [MS vs. WC] Placental Gene Expression Profiles during Fetal Development

#### 1.1 Principal component analysis (PCA) of 30 short-oligonucleotide arrays

For initial exploratory analysis of the thirty placental gene expression arrays, PCA [34], [59] was performed using JMP Genomics. Figure S1 depicts the first three principal components and each component explains variance across all microarrays, respectively. At each of the gestational intervals, microarrays cluster by gestational day and also by breed. In general, with the exception of D25 samples, each breed/date combinations cluster separately. We also note the array containing a male fetus at D65 (D65_M_B) shows similar larger variance to female-only sample D65_M_C sample.

#### 1.2 Volcano plot depicts breed specific differences of MS vs. WC placental gene expression profiles during fetal development

**Figure 1:**
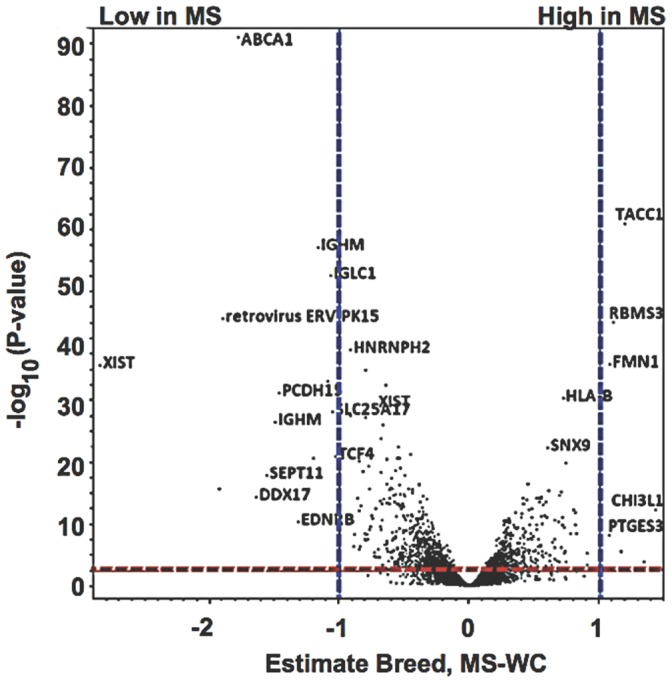
Differential placental gene expression in Meishan versus WC swine breeds. Volcano plots were used to visualize differential expression between Meishan and White Composite placental tissues against level of significance surveyed for breed specific differences. The x-axis is the log_2_ fold-change difference of the Meishan minus WC breed groups. The vertical axis represents the statistical evidence as a measure of the –log_10_ transformation of the p-value for each test of differences between samples. Each of the ∼24,000 oligonucleotide probe sets is plotted. A red dashed line indicates the FDR adjustment (approximately q-value <0.05) to correct for multiple testing. Blue dashed lines showed estimates of 1, and −1, which corresponds to a 2-fold (inverse natural logarithm of estimates) increase or decrease respectively.

In order to visualize genes differentially expressed between the two breeds, volcano plots were used to show estimates of change (abscissa, log_2_-transformed) against significance (ordinate axis, −log_10_-transformed) between Meishan and WC breed placental tissues (Figure 1). Positive estimates correspond to genes up-regulated in Meishans. In the upper right (Meishan, upregulated) and upper left (WC upregulated) corners of the volcano plot are gene products expressed at greater than a two-fold change (vertical dashed lines) and cyan-colored probe sets are labeled for convenience where q-value <0.05. It should be noted, that these differences are not due to a single probe hybridization defect as the linear mixed model contained a covariate to account for identified probe-by-breed effects [15].

A total of 1,595 genes were differentially expressed (log_2_-transformed, q-value ≤0.05, Figure 1, Table S2) in the combined analysis comparing breed across all time points. *ABCA1*–a cholesterol efflux regulatory protein–and *XIST*–a long non-coding RNA involved in X-chromosome inactivation–were highly expressed in the WC placentae. By comparison, formin (*FMN1*), a cartilage glycoprotein (chitinase 3-like 1; *CHI3L1*), and *TACC1* (transforming, acidic coiled-coil containing protein 1) were highly expressed in MS placental tissues and are implicated in cell adhesion, remodeling and structural architecture of the placenta. Comparisons of the differentially expressed genes (log_2_-transformed, q <0.05) by breed are summarized in Table S2. Whenever possible, a description of gene function or protein activity is provided for top candidates that showed significant expression differences.

### 2 Microarray Validation

In addition to using principal component analysis (Figure S1) and array group correlations to assess the quality of our microarray hybridization data (data not shown) [60], we sought to evaluate the short-oligonucleotide microarray results by the orthogonal reverse-transcription quantitative polymerase chain reaction (RT-qPCR) method as outlined by the Microarray Quality Control (MAQC) project [61], [62]. The housekeeping genes *RPL18* and *RPS20* were used as internal controls to compare across samples and similar amplification efficiencies (>95%) were observed for all primers used. A summary of RT-qPCR results is presented in Table 1. The direction of fold change is concordant with microarray results and thus validates the microarray findings. We also explored a subset of cholesterol pathway genes by RT-qPCR, and these results were also concordant with microarray findings (discussed in Results, Section 3.8).

**Table 1:** EvaGreen RT-qPCR analysis of select genes across placental datasets.

Gene	Gene Description	Probe	Description	Day	N	Std ErrMS	Std ErrWC	Fold Change(MS-WC)	Student *t*-Test ΔC_t_ (Normalization)
***ABCA1***	ATP-binding cassette transporter member 1	Ssc.7146.A1_a1	Cholesterol Efflux	25	6	0.14	0.30	−6.63	3.11E–4§
				45	6	0.10	0.21	−3.65	2.55E–3§
				65	6	0.24	0.86	−4.40	7.15E–4§
				85	6	0.08	0.63	−5.32	6.21E–3§
				105	6	0.17	0.60	−8.15	3.955E–3§
***XIST*** *	X (inactive)-specific transcript	Ssc.2434.1.A1_at	X-Cs inactivation	25	6	0.68	0.42	−1573.8	3.08E–02§
				45	6	0.19	0.25	−14.02	5.38E–08§
				65	6	0.60	0.96	−138.14	4.28E–02§
				85	6	0.31	0.69	−2149.8	3.82E–02§
				105	6	0.44	0.41	−576.03	1.47E–02§
***XIST*** *	X (inactive)-specific transcript	Ssc.31029.1.A1_at	X-Cs inactivation	25	6	0.30	0.56	−2.19	5.11E–06§
				45	6	0.45	1.00	−1.84	1.46E–04§
				65	6	0.59	0.95	−1.12	3.56E–02§
				85	6	0.35	0.28	−4.72	1.82E–02§
				105	6	0.25	0.23	−1.68	3.67E–02§
***PHLDA2***	pleckstrin homology-like domain, family A, member 2	Ssc.9796.1.A1_at	Genomic Imprinting	65	6	0.57	0.82	2.27	1.48E–02§
***CDKN1C***	cyclin-dependent kinase inhibitor 1C (p57, Kip2)	Ssc.8871.1.S1_at	Genomic Imprinting	65	6	0.15	0.29	1.82	4.08E–06§
***RPS20***	ribosomal protein S20	Ssc.20036.1.S1_at	Internal Reference	65	6	0.49	0.07	–	–
***RPL18*** *	ribosomal protein L18	Ssc.10553.1.A1_a_at	Internal Reference	25	6	0.38	0.83	–	–
***RPL18*** *	ribosomal protein L18	Ssc.10553.1.A1_a_at	Internal Reference	45	6	0.43	0.50	–	–
***RPL18*** *	ribosomal protein L18	Ssc.10553.1.A1_a_at	Internal Reference	65	6	0.62	0.91	–	–
***RPL18*** *	ribosomal protein L18	Ssc.10553.1.A1_a_at	Internal Reference	85	6	0.21	0.44	–	–
***RPL18*** *	ribosomal protein L18	Ssc.10553.1.A1_a_at	Internal Reference	105	6	0.26	0.51	–	–

* Denotes ΔC_t_ values normalized with *RPL18*.

§ Denotes significances p<0.05.

### 3 Expression of XIST in Meishan and White Composite Breeds

**Figure 2:**
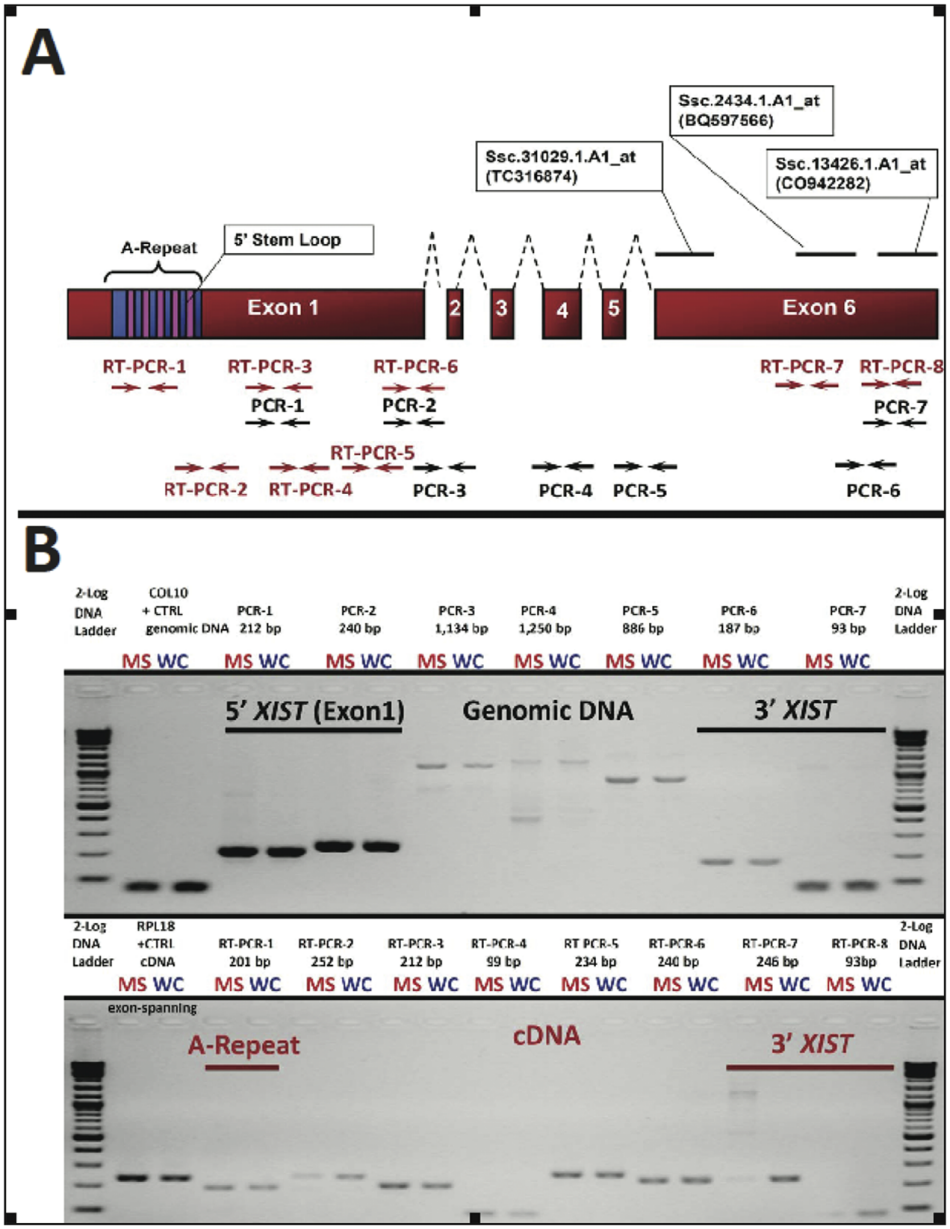
*XIST* structure and transcription in Meishan and WC placentas; expression of a short isoform in the Meishan breed. Swine *XIST* was discovered and annotated from reciprocal BLAT with bovine *XIST* as it is not annotated in the current pig 9.2 genome assembly. (A) Specifically, we identified BAC CH242-76N1 (GI: 219925014) that contained porcine *XIST*. Biochemical studies involving mutated or truncated *XIST* transcripts revealed the A-repeat region as the functional element responsible for X-chromosome inactivation [65]. We mapped Affymetrix probesets and the corresponding ESTs to porcine *XIST* and designed a series of RT-qPCRs (bracketed numbers) to validate microarray results (Table 1). (B) Agarose gel electrophoresis depicting representative PCR assays to amplify regions of genomic DNA or cDNA from D25 MS or WC fetuses for the chromosomal interval SSCX: 58,375,000-58,400,000 based on the assembly (SGSC Sscrofa9.2/susScr2 and BAC CH242-76N1 (GI: 219925014). A postive control for genomic DNA isolation is shown for *COL10*. For analysis of placental RNA isolation and cDNA generation, we performed reverse-transcription PCR with exon-spanning primers for the positive control *RPL18* (Figure 2B, bottom panel, lanes 2+3). The short *XIST* isoform was absent from additional MS gestations (Table 1).

*XIST*, a long non-coding RNA that facilitates X-chromosome inactivation (XCI) to balance sex chromosomes in placental mammals [63], and that has been shown to be imprinted in extraembryonic tissues, was differentially expressed between the two breeds with virtually no detection in the MS breed at any time point. Non-coding RNAs are known to be less-conserved than protein-coding sequences; however, re-annotation by BLAT analysis using bovine *XIST* (genbank accession: NR_001464.2) identified multiple probe sets (*Ssc.31029.1.A1_at*, *Ssc.2434.1.A1_at* and *Ssc.13426.1.A1_at)*, which mapped to the 3′ region of the bovine *XIST* confirming the correct annotation of the array probes. For clarity, an illustration of the putative swine *XIST* mRNA is shown in Figure 2A.

As X-inactivation is initiated at the *XIST* gene locus by an inside-out mechanism [64], we hypothesized that neighboring genes known to be inactivated by *XIST* should be upregulated in Meishan expression profiles due to abnormal X-inactivation. Expression of ten dosage-compensated genes (*HSD17B10, KLF8, MSN, MTCP1, OCRL*, *SLC25A6, SLC25A5, SNX12, RBBP7* and *TIMM17B*) was examined by microarray linear-mixed model analysis. Seven dosage-compensated genes were not upregulated in Meishans placentae (*MSN, MTCP1, OCRL, RBBP7, SLC25A6, SNX12,* and *TIMM17B*) supporting that the *XIST* is functional in Meishan placentae, thus placing the microarray *XIST* expression results into question. As multiple *XIST* 3′-ESTs were identified by our transcriptome profiling at D25, D45, D85, D105 gestational intervals, we sought to clarify if *XIST* expression was concordant with our array findings by using RT-qPCR (Table 1). Similar trends in fold-change were observed by both methods, and therefore validate our microarray observations.

Because we were unable to detect 3′ regions of *XIST* by both microarray and RT-qPCR in Meishans, we next sought to clarify if 5′ regions were detectable. Human EST databases support at least 10 human *XIST* spliced variants, and multiple *XIST* isoforms that differ by truncation of both 5′ and 3′ ends [55]. Importantly, Wutz *et al* 2002 [65] identified a series of stem-loops within conserved *XIST* exon 1 (A-repeat region) required for chromosomal silencing, and subsequent reports have shown the 5′ A-stem loops are necessary and sufficient to recruit polycomb repressive complex machinery, facilitate splicing of *XIST* RNA, and maintain random X-inactivation. We designed a series of RT-PCR’s to investigate whether the functionally conserved element (A-stem loops) of porcine *XIST* is present in Meishans and expressed in Meishans (Table 1 and Figure 2). We also examined whether the inability to detect the 3′ end of the Meishan *XIST* transcript was due to a genomic deletion. As shown in Figure 2, there were no structural differences between the two breeds in the regions examined, and the data indicates that the 3′ end of the *XIST* is present, but not transcribed, in the Meishan breed. Combined these data suggest that while the *XIST* gene appears to be processed differently between the breeds (short isoform in the MS); in both cases, it is capable of X-inactivation.

### 4 Breed-specific X-chromosome Regional Gene Expression Differences

**Figure 3:**
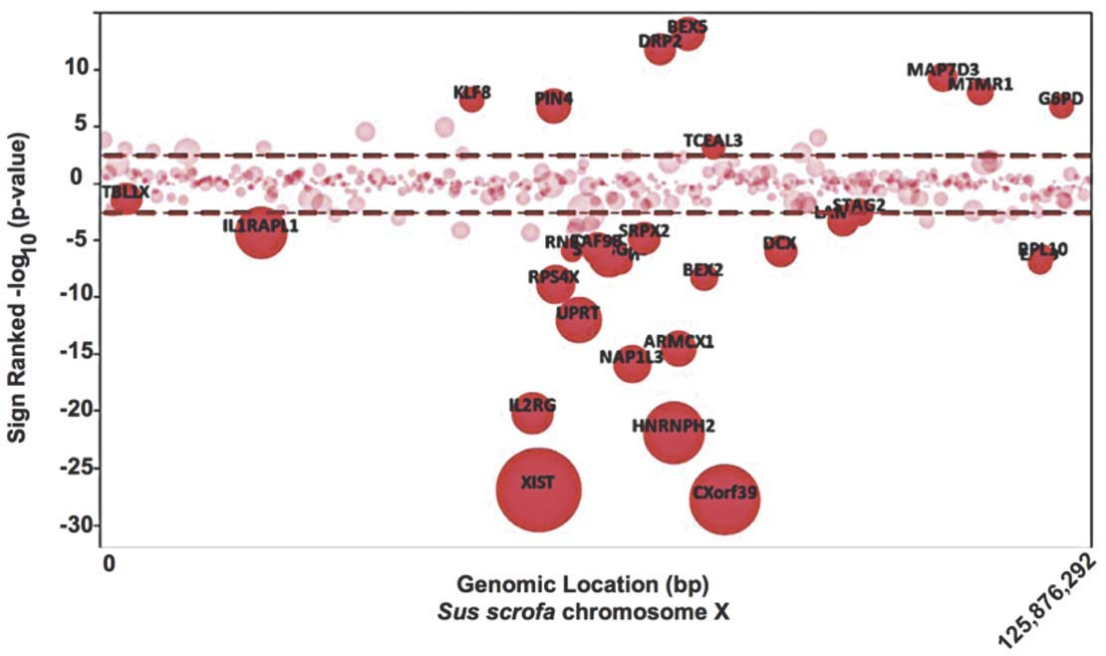
Non-random distribution of Meishan versus WC differentially expressed genes along the X-chromosome. A bubble plot is depicted for the swine X chromosome (SSCX) in which physical coordinates are plotted (abscissa, x-axis) against sign-ranked –log_10_(p-values) (ordinate, y-axis). Estimate values from the linear mixed model were used to calculate positive (Meishan) or negative (WC) signs. Each bubble represents a specific probe set printed on the short-oligonucleotide array.

In order to determine whether there were other breed differences with respect to the X chromosome, regional differences in gene expression were determined. A bubble plot of X chromosome location versus sign-ranked significance modeling only for breed effect is presented in Figure 3. Additionally, differential gene locus mapping (DIGMAP) [57] was used to determine if the differentially expressed genes were randomly distributed along the X chromosome or located in specific regions. As shown in Figure 3 and Table 2 several clusters or enriched regions were identified. The chromosomal band Xq13 (p-value <2.29E-04) corresponding to genes CHIC1, *DLG3*, *IL2RG*, *OGT, PIN4*, *RNF12*, *RPS4X*, *SH3BGRL, SNX12, TAF9B, XIST, YIPF6* and *ZMYM3* ranked highest by criteria of placental gene expression and chromosomal location. Also, the Xq13 interval has been associated with several quantitative trait loci (QTL) including pig fat deposition and carcass musculature [12], [66]–[70].

**Table 2:** Gene expression and DIGMAP analysis of *Sus scrofa* chromosome X.

Band	p-value	Gene	Description
**Xq13**	2.29E-04*	*CHIC1*	cysteine-rich hydrophobic domain 1
		*DLG3*	discs, large homolog 3 (neuroendocrine-dlg, Drosophila)
		*IL2RG*	interleukin 2 receptor, gamma (severe combined immunodeficiency)
		*OGT*	O-linked N-acetylglucosamine (GlcNAc) transferase (UDP-N-acetylglucosamine:polypeptide-N-acetylglucosaminyl transferase)
		*PIN4*	protein (peptidylprolyl cis/trans isomerase) NIMA-interacting, 4 (parvulin)
		*RNF12*	ring finger protein 12
		*RPS4X*	ribosomal protein S4, X-linked
		*SH3BGRL*	SH3 domain binding glutamic acid-rich protein like
		*SNX12*	sorting nexin 12
		*TAF9B*	TAF9B RNA polymerase II, TATA box binding protein (TBP)-associated factor, 31kDa
		*XIST*	X (inactive)-specific transcript
		*YIPF6*	Yip1 domain family, member 6
		*ZMYM3*	zinc finger, MYM-type 3
**Xq21**	2.94E-02*	*ARMCX1*	armadillo repeat containing, X-linked 1
		*ATRX*	alpha thalassemia/mental retardation syndrome X-linked (RAD54 homolog, S. cerevisiae)
		*CHM*	choroideremia (Rab escort protein 1)
		*COX7B*	cytochrome c oxidase subunit VIIb
		*NAP1L3*	nucleosome assembly protein 1-like 3
		*PABPC5*	poly(A) binding protein, cytoplasmic 5
		*SRPX2*	sushi-repeat-containing protein, X-linked 2
**Xp11**	5.05E-02*	*ARID4B*	AT rich interactive domain 4B (RBP1- like)
		*CASK*	calcium/calmodulin-dependent serine protein kinase (MAGUK family)
		*CFP*	complement factor properdin
		*EBP*	emopamil binding protein (sterol isomerase)
		*FUNDC1*	FUN14 domain containing 1
		*KLF8*	Krüppel-like factor 8
		*MAGED2*	melanoma antigen family D, 2
		*RGN*	regucalcin (senescence marker protein-30)
		*TFE3*	transcription factor binding to IGHM enhancer 3

* Denotes significant at adjusted p-value <0.05.

### 5 Cholesterol Synthesis Differences Predicted by Gene Ontology and Pathway Analysis

A common approach to clarify transcriptome datasets is to enrich for functionality using the controlled gene ontology vocabulary of molecular function, biological process and cellular component. By annotating gene lists with GO terms, the goal is to reduce the complexity of the data in such a way that differentially expressed genes can be targeted to a common process(es) which can be investigated further. The Database for Annotation, Visualization, and Integrated Discovery, commonly referred to as DAVID (9) allowed us to explore coordinated biological processes in the placental datasets and unveiled cholesterol biosynthesis (GO: 0006695, p<1×10^−5^) as the top-ranked molecular term describing differences between the pig breeds (Table 3).

**Table 3:** Summary of top-ranking common gene ontology (GO) molecular processes in swine placentae.

Gene Ontology	P-value for MS-WC
cholesterol biosynthesis	0.00001*
peroxisome	0.00001*
isoprenoid biosynthesis	0.00002*
pigmentation	0.00066*
nuclear heterochromatin	0.00082*

* Denotes significance at p<0.05 after multiple correction testing.

**Figure 4:**
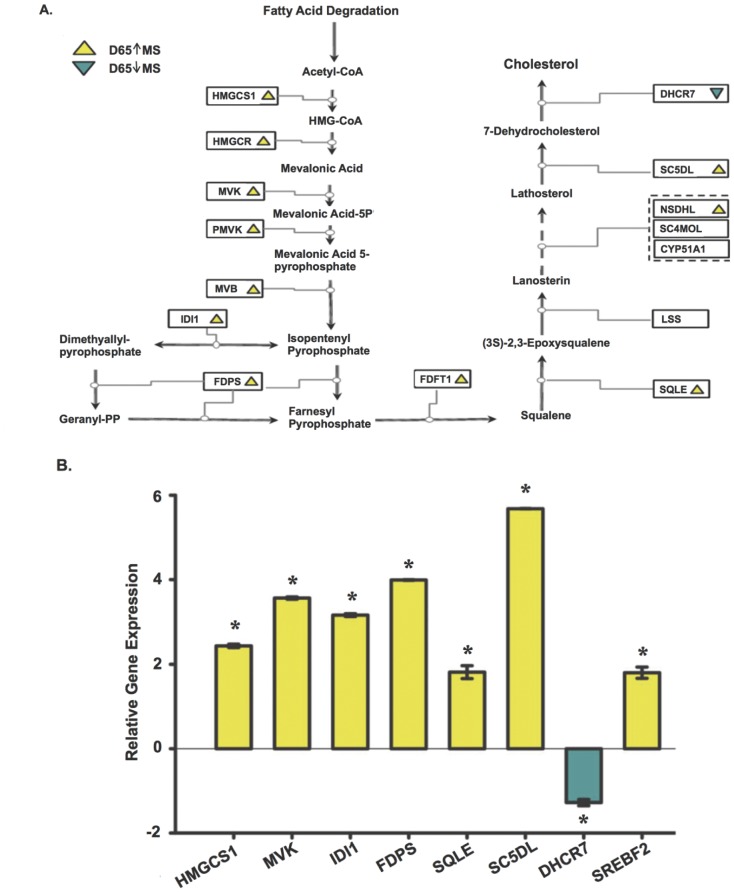
Gene targets enriched in Gene Ontology and KEGG cholesterol biosynthetic pathways. (A) Collective analyses by DAVID and Ingenuity pathway tools indicated significant upregulation of sterol biosynthesis (cholesterol) in the placentae of Meishan breed. Using the KEGG cholesterol biochemical pathway as a template, we mapped expression pattern differences (yellow, upregulation in MS; blue, downregulation in MS) corresponding to placental expression breed differences at D65. The final catalytic step of cholesterol production is mediated by the reductase *DHCR7*, an imprinted gene [40]. (B) Bar graphs indicating relative quantitation by EvaGreen RT-qPCR of D65 placentae were used to determine gene expression intensities of a subset of cholesterol biosynthetic genes. Normalization across biological replicates and breed groups were performed using housekeeping gene *RPS20*. A two-tailed heteroscedastic Student *t*-test was used to report significance (p<0.05). Error bars reflect standard error of the mean for three placental samples after three repeated measurements of the same group (technical replicates).

**Figure 5:**
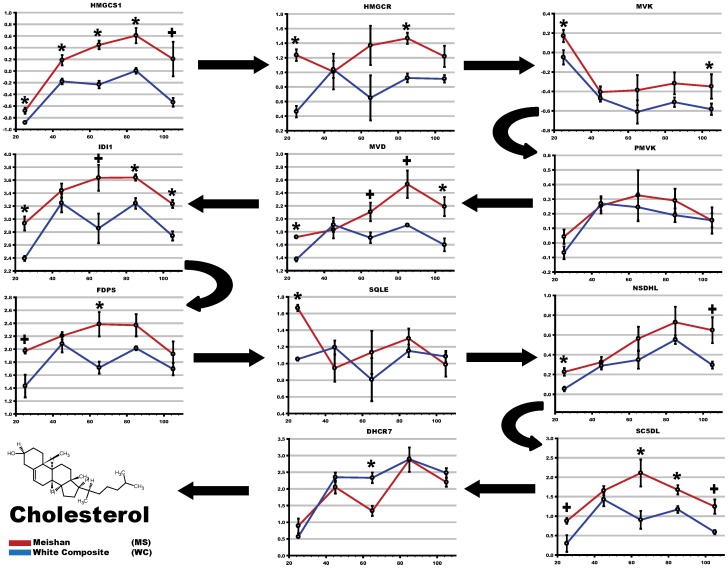
Temporal changes in cholesterol biosynthesis gene expression throughout gestation. To visualize and identify patterns of gene expression, the KEGG cholesterol biosynthetic genes were plotted at each gestational time point (x-axis) using mean intensities (y-axis) of normalized microarray data. Arrows denote the metabolic flux through biochemical pathway: that is, the biochemical steps in which acetyl co-enzyme A is processed into cholesterol. A shaded grey box is overlaid for convenience to show the D45–D65 breed-specific cholesterol pattern.

Mapping enriched genes into established metabolic pathways is an attractive approach to deconstruct molecular phenotypes from microarray datasets. To better visualize the fraction of microarray data contributing to canonical such as KEGG [52] networks, we used Ingenuity Pathway Analysis (IPA). As data input, we used differentially expressed genes (q <0.05) to construct the networks (Table 4). Analysis using the canonical pathways [52], [71] revealed upregulation of mevalonic acid and HMG-CoA reductase pathways in Meishan placental tissues. This observation is likely to contribute to the principal increases observed by expression profiling in sterol metabolism [72], as MVK is a major component of both cholesterol and terpenoid pathways [73]. Taken together, cholesterol metabolism genes showing significant differential expression were *CYP51A1*, *EBP, FDFT1, FDPS*, *HMGCS1*, *IDI1, MVD*, *MVK*, *SC5DL*, *SQLE, SREBF2* and *TM7SF2* (Figures 4, 5). The biochemical committed step in cholesterol synthesis is catalyzed by squalene epoxide (p<0.02, Figure 4A) [74]. Our analysis revealed several genes epistatic to the catalytic step for commitment of cholesterol synthesis, e.g. *FDPS*, *FDFT1*, *HMGCR*, *IDI1*, *MVK*, *MVD* (Table 5, Figures 4, 5), and upregulation may serve to modulate flux through multiple sterol pathways, e.g. isoprenoid (2×10^−5^, Table 3). Intriguingly, DHCR7, an enzyme that mediates the last catalytic step for cholesterol synthesis, is downregulated with respect to Meishan. *DHCR7* (Figure 4B, RT-qPCR p<5.82×10^−8^) is also implicated as a negative regulator of the hedgehog signaling cascade, and we speculate downregulation may serve to increase SHH signaling in the placenta.

**Table 4:** Summary of Ingenuity ranked canonical (KEGG) pathways.

Ingenuity Canonical Pathways	−log(B-H p-value)	Genes
**Biosynthesis of Steroids**	4.12*	*MVD, FDPS, FDFT1, EBP, CYP7B1, IDI1, MVK, NQO1, HMGCR, SC5DL*
**Antigen Presentation Pathway**	2.35*	*HLA-DMA, HLA-DRB4, HLA-A, HLA-B, HLA-DRB1, CANX, TAP1, HLA-C*
**Glutathione Metabolism**	2.07*	*MGST1, MGST2, GSTA4, GSTM3 (includes EG:2947), GSTA1, G6PD, GGT6, IDH2, GPX7, ANPEP, GSTO1*
**LPS/IL-1 Mediated Inhibition of** **RXR Function**	1.94*	*MGST1, GSTM3 (includes EG:2947), GSTA1, ABCB11, ACSL6, GSTO1, ABCA1, LY96, SULT4A1, GSTA4, UST, MGST2, MAP3K7, SLC27A6, FABP4, XPO1, NR5A2, LBP, PLTP, TNFRSF1B, ACOX3, HMGCS1, CYP4A11, ALDH7A1*
**Complement System**	1.93*	*C1R, C1S, CD55, C1QC, C1QA, CD46, C1QB*
**LXR/RXR Activation**	1.88*	*RXRG, LY96, CCL2, FASN, ACACA, LBP, PLTP, TNFRSF1B, HMGCR, HADH, ABCA1*
**Valine, Leucine and Isoleucine Degradation**	1.88*	*HSD17B10, PCCA, ACAD8, ECH1, ELOVL2, OXCT1, AOX1, HMGCS1, HADH, ALDH7A1, MCCC2*
**Hepatic Fibrosis/Hepatic Stellate Cell Activation**	1.82*	*MYH10, CCR5, VEGFB (includes EG:7423), CTGF, MYL6, EDNRB, MMP2, COL1A2, COL1A1, LY96, CCL2, IGFBP3, LBP, TNFRSF1B, A2M, EGFR, COL3A1*
**Caveolar-mediated Endocytosis Signaling**	1.66*	*FYN, HLA-A, ACTB, CD55, ITGA2, COPA, HLA-B, ACTG1, EGFR, HLA-C, COPG*
**Arachidonic Acid Metabolism**	1.65*	*TMEM87B, CYP4A22, CYP2U1, PLA2G10, PTGS1, YWHAZ, GGT6, CYP2D6, GPX7, PLOD1, CYP1B1, LAMB2, PTGES3 (includes EG:10728), MGST2, CYP19A1, CYP4B1, CYP4A11, CYP51A1*
**Actin Cytoskeleton Signaling**	1.59*	*MYH10, TIAM1, PFN1, PIK3C2A, MYL6, ARPC1B, GRB2, ACTB, GNA12, ITGA2, C3ORF10, PIKFYVE, GSN, ACTG1, PDGFC, PTK2, PAK1, TIAM2 (includes EG:26230), FGF23, PPP1R12A, DIAPH2, LBP, PDGFD, PPP1CA*
**Fatty Acid Metabolism**	1.47*	*HSD17B10, CYP4A22, ACSL6, ECH1, CYP2D6, CYP1B1, CYP19A1, PECI, CYP4B1, ACAD8, SLC27A6, ACOX3, CYP4A11, HADH, CYP51A1, ALDH7A1*
**Aryl Hydrocarbon Receptor Signaling**	1.36*	*MGST1, GSTM3 (includes EG:2947), GSTA1, NQO1, SMARCA4, GSTO1, CYP1B1, RXRG, CCND2, PTGES3 (includes EG:10728), CCNA1, MGST2, GSTA4, NFIB, CDK2, ALDH7A1*
**Type I Diabetes Mellitus Signaling**	1.32*	*HLA-DMA, JAK1, ICA1, CD28, IKBKG, NFKBIA, HLA-A, MAP3K7, HLA-B, TNFRSF1B, SOCS5, CPE, HLA-C*

* Denotes significance greater than 1.30, corresponds to –(log of Benjami-Hochberg corrected p-value <0.05.

**Table 5:** Breed-specific microarray expression levels of sterol biosynthesis genes identified as enriched by Ingenuity Pathway Analysis.

Gene	Gene Description	Pathway or Process	D25 MS Mean+StD	D25 WC Mean+StD	D45 MS Mean+StD	D45WC Mean+StD	D65 MS Mean+StD	D65WC Mean+StD	D85 MS Mean+StD	D85 WC Mean+StD	D105 MS Mean+StD	D105 WC Mean+StD	Breed Fold Change (MS-WC)
ABCA1	atp-binding cassette, sub-family a (abc1), member 1	cholesterol biosynthesis	0.14±0.032	1.48±0.053	0.02±0.048	1.71±0.128	0.19±0.139	2.3±0.127	0.47±0.032	2.43±0.129	0.62±0.131	2.41±0.078	-3.43 q <0*
APOF	apolipoprotein F	cholesterol biosynthesis	−0.09±0.091	−0.38±0.091	−0.2±0.052	−0.48±0.097	−0.28±0.223	−0.56±0.164	−0.16±0.046	−0.67±0.071	−0.39±0.074	−0.6±0.151	1.24 q <1E-3*
CYB5R3	cytochrome B5 reductase 3	cholesterol biosynthesis	2.26±0.061	1.87±0.139	1.32±0.092	1.92±0.108	1.69±0.085	2.06±0.116	1.99±0.097	2.37±0.02	2.47±0.18	2.73±0.006	−1.2 q <3E-3*
CYP4B1	cytochrome P450, family 4, subfamily b, polypeptide 1	cholesterol biosynthesis	−0.28±0.099	−0.27±0.047	1.46±0.188	0.48±0.063	0.25±0.387	0.55±0.269	1.22±0.259	0.25±0.062	1.6±0.274	0.55±0.09	1.48 q <1E-3*
CYP51A1	cytochrome P450, family 51, subfamily a, polypeptide 1	cholesterol biosynthesis	1.89±0.157	1.26±0.115	3.17±0.089	2.98±0.083	3.18±0.299	2.66±0.301	3.07±0.101	2.56±0.077	2.8±0.149	2.27±0.033	1.38 q <1E-4*
CYP7B1	cytochrome P450, family 7, subfamily b, polypeptide 1	cholesterol biosynthesis	0.92±0.165	1.44±0.081	2.65±0.039	2.8±0.026	1.83±0.284	2.3±0.034	1.29±0.236	1.43±0.259	0.72±0.116	0.86±0.135	−1.21 q <0.05*
DHCR7	7-dehydrocholesterol reductase	cholesterol biosynthesis	0.89±0.218	0.56±0.037	2.05±0.19	2.35±0.139	1.34±0.15	2.33±0.158	2.88±0.366	2.89±0.057	2.2±0.136	2.48±0.145	−1.23 q <0.08
EBP	emopamil binding protein (sterol isomerase)	cholesterol biosynthesis	2.21±0.303	1.3±0.05	1.99±0.083	2.11±0.111	2.16±0.153	1.77±0.131	2.54±0.118	2.13±0.127	1.99±0.103	1.57±0.146	1.32 q <1E-3*
FABP4	fatty acid binding protein 4	cholesterol biosynthesis	−1.39±0.069	−1.4±0.026	−1.17±0.046	−1.1±0.062	−1.34±0.087	−0.47±0.434	−0.97±0.055	−0.64±0.333	−1.01±0.097	−0.37±0.118	−1.31 q <0.02*
FDFT1	farnesyl-diphosphate farnesyltransferase 1	cholesterol biosynthesis	2.58±0.079	1.87±0.131	2.07±0.048	2.12±0.084	2.17±0.124	1.85±0.089	2.56±0.193	2.09±0.064	2.23±0.164	1.88±0.063	1.3 q <6E-4*
FDPS	farnesyl diphosphate synthase	cholesterol biosynthesis	1.97±0.041	1.43±0.175	2.21±0.057	2.08±0.132	2.38±0.188	1.71±0.093	2.37±0.171	2.01±0.031	1.92±0.195	1.69±0.094	1.31 q <3E-4*
G6PD	glucose-6-phosphate dehydrogenase	cholesterol biosynthesis	0.33±0.042	0.08±0.036	0.24±0.064	−0.09±0.035	0.15±0.2	0.12±0.123	0.49±0.108	−0.1±0.061	−0.08±0.07	−0.34±0.049	1.24 q <1E-4*
HMGCR	3-hydroxy-3-methylglutaryl-coA reductase	cholesterol biosynthesis	−1.47±0.071	−1.48±0.029	−1.36±0.036	−1.47±0.048	−1.44±0.133	−1.38±0.107	−1.38±0.07	−1.5±0.042	−1.3±0.094	−1.43±0.049	1.38 q <9E-4*
HMGCS1	3-hydroxy-3-methylglutaryl-coA synthase 1	cholesterol biosynthesis	1.59±0.118	0.81±0.052	2.33±0.171	2.25±0.122	2.62±0.237	1.86±0.258	2.7±0.181	2.14±0.095	2.15±0.185	1.65±0.056	1.45 q <3E-4*
HMGCS1	3-hydroxy-3-methylglutaryl-coa synthase 1	cholesterol biosynthesis	−0.68±0.052	−0.89±0.012	0.19±0.089	−0.18±0.049	0.45±0.074	−0.23±0.065	0.61±0.129	0±0.05	0.2±0.295	−0.54±0.075	1.43 q <2E-8*
IDI1	isopentenyl-diphosphate delta isomerase 1	cholesterol biosynthesis	2.93±0.109	2.39±0.039	3.44±0.108	3.25±0.144	3.64±0.2	2.86±0.229	3.64±0.049	3.24±0.083	3.23±0.062	2.74±0.072	1.41 q <1E-6*
INSIG1	insulin induced gene 1	cholesterol biosynthesis	0.04±0.104	−0.19±0.084	−0.42±0.105	−0.41±0.131	−0.22±0.275	−0.53±0.197	−0.4±0.017	−0.55±0.08	−0.61±0.087	−0.55±0.067	1.23 q <0.04*
LPL	lipoprotein lipase	cholesterol biosynthesis	−0.72±0.11	−0.74±0.108	0.48±0.254	−0.37±0.172	−0.56±0.224	−0.47±0.14	−0.59±0.091	−0.87±0.032	−0.61±0.094	−0.87±0.092	1.21 q <0.07
MVD	mevalonate (diphospho) decarboxylase	cholesterol biosynthesis	1.72±0.004	1.37±0.032	1.83±0.131	1.91±0.111	2.11±0.143	1.71±0.08	2.53±0.212	1.9±0.019	2.19±0.147	1.6±0.099	1.3 q <8E-4*
MVK	mevalonate kinase	cholesterol biosynthesis	0.99±0.058	0.57±0.034	0.91±0.061	0.91±0.083	1.11±0.127	0.77±0.115	1.34±0.149	0.99±0.017	1.18±0.106	0.69±0.057	1.25 q <3E-5*
MVK	mevalonate kinase	cholesterol biosynthesis	0.17±0.063	−0.05±0.074	−0.41±0.059	−0.47±0.033	−0.39±0.155	−0.61±0.119	−0.32±0.112	−0.51±0.049	−0.35±0.126	−0.58±0.059	1.15 q <0.02*
NPC2	niemann-pick disease, type C2	cholesterol biosynthesis	1.8±0.042	2.14±0.099	1.98±0.059	2.31±0.097	2.29±0.09	3.49±0.275	3.07±0.151	3.43±0.211	3.52±0.1	4.22±0.263	−1.5 q <1E-5*
NR5A2	nuclear receptor subfamily 5, group a, member 2	cholesterol biosynthesis	0.09±0.12	−0.16±0.069	−0.19±0.016	−0.32±0.047	−0.28±0.126	−0.39±0.138	−0.32±0.12	−0.41±0.031	−0.09±0.082	−0.4±0.019	1.13 q <0.04*
NSDHL	NAD(P) dependent steroid dehydrogenase-like	cholesterol biosynthesis	0.23±0.038	0.05±0.023	0.32±0.053	0.29±0.039	0.56±0.12	0.35±0.089	0.73±0.158	0.55±0.043	0.65±0.131	0.29±0.036	1.14 q <0.02*
NSDHL	NAD(P) dependent steroid dehydrogenase-like	cholesterol biosynthesis	0.52±0.076	0.05±0.02	0.7±0.055	0.77±0.079	0.86±0.155	0.65±0.177	1.15±0.14	0.89±0.077	0.91±0.085	0.68±0.077	1.16 q <0.04*
OSBPL1A	oxysterol binding protein-like 1A	cholesterol biosynthesis	−0.59±0.08	−0.64±0.06	0.21±0.077	1.05±0.087	0.33±0.13	0.88±0.17	0.43±0.055	0.79±0.16	−0.05±0.159	0.42±0.126	−1.34 q <2E-5*
PRKAA2	protein kinase, amp-activated, alpha 2 catalytic subunit	cholesterol biosynthesis	−0.89±0.003	−0.98±0.06	−0.4±0.103	−0.66±0.08	−0.07±0.126	−0.51±0.125	−0.22±0.151	−0.32±0.062	−0.14±0.186	−0.56±0.009	1.2 q <7E-3*
SC5DL	sterol-c5-desaturase -like	cholesterol biosynthesis	0.87±0.074	0.3±0.216	1.66±0.117	1.43±0.172	2.11±0.346	0.9±0.229	1.68±0.118	1.17±0.087	1.24±0.186	0.59±0.055	1.56 q <8E-6*
SREBF2	sterol regulatory element binding transcription factor 2	cholesterol biosynthesis	2.04±0.05	1.77±0.079	2.09±0.095	2.19±0.095	2.36±0.152	1.86±0.098	2.4±0.155	2.2±0.03	2.14±0.133	1.8±0.117	1.18 q <0.02*
TM7SF2	transmembrane 7 superfamily member 2	cholesterol biosynthesis	0.32±0.361	−0.11±0.051	1.61±0.226	1.91±0.184	2.36±0.109	1.32±0.143	3.11±0.171	2.09±0.091	2.07±0.206	1.08±0.223	1.57 q <4E-5*
ABCA1	atp-binding cassette, sub-family a, member 1	LXR/RXR activation	0.14±0.032	1.48±0.053	0.02±0.048	1.71±0.128	0.19±0.139	2.3±0.127	0.47±0.032	2.43±0.129	0.62±0.131	2.41±0.078	−3.43 q <0*
ACACA	acetyl-coA carboxylase alpha	LXR/RXR activation	0.09±0.037	−0.01±0.031	−0.13±0.087	−0.22±0.026	−0.14±0.201	−0.24±0.16	−0.07±0.079	−0.29±0.026	−0.17±0.072	−0.3±0.045	1.22 q <8E-5*
CCL2	chemokine (c-c motif) ligand 2	LXR/RXR activation	−0.56±0.085	−0.45±0.262	−0.65±0.021	−0.66±0.029	−0.66±0.102	−0.53±0.144	−0.45±0.105	−0.45±0.133	−0.66±0.127	0.81±0.373	−1.27 q <0.02*
CYP4A11	cytochrome P450, family 4, subfamily A, polypeptide 11	LXR/RXR activation	−0.19±0.538	−0.65±0.074	−0.32±0.231	−0.67±0.05	−0.31±0.255	−0.62±0.103	0.9±0.342	−0.75±0.03	−0.07±0.435	−0.61±0.054	0.62 q <3E-3*
CYP4A22	cytochrome P450, family 4, subfamily A, polypeptide 22	LXR/RXR activation	−0.83±0.117	−0.95±0.018	−0.61±0.065	−0.8±0.024	−0.67±0.058	−0.54±0.071	−0.3±0.16	−0.87±0.073	−0.41±0.05	−0.51±0.055	0.17 q <0.02*
FASN	fatty acid synthase	LXR/RXR activation	1.59±0.061	1.03±0.051	1.17±0.174	1.35±0.08	1.33±0.292	0.88±0.212	1.15±0.049	0.84±0.064	0.5±0.125	0.54±0.18	1.27 q <8E-3*
HADH	fatty acid synthase hydroxylacyl-coA dehydrogenase	LXR/RXR activation	2.03±0.051	2.17±0.094	1.84±0.101	2.1±0.103	1.69±0.129	2.21±0.224	2.09±0.15	2.34±0.073	1.97±0.118	2.25±0.033	−1.22 q <8E-3
LBP	lipopolysaccharide binding protein	LXR/RXR activation	−0.73±0.042	−0.68±0.024	−0.17±0.05	−0.45±0.111	0.5±0.209	−0.67±0.13	0.29±0.073	−0.81±0.007	0.09±0.174	−0.47±0.081	1.53 q <1E-13*
LY96	lymphocyte antigen 96	LXR/RXR activation	−0.65±0.055	−0.27±0.035	−0.58±0.019	−0.56±0.081	−0.45±0.069	−0.09±0.258	−0.29±0.098	−0.13±0.013	−0.31±0.098	0.25±0.137	−1.23 q <3E-3*
LYCAT	lysocardiolipin acyltransferase 1	LXR/RXR activation	−0.51±0.039	−0.65±0.059	0.71±0.032	−0.6±0.319	0.28±0.126	0.12±0.063	0.49±0.028	0.45±0.032	0.34±0.132	−0.38±0.324	0.45 q <3E-4*
OSBPL10	oxysterol binding protein-like 10	LXR/RXR activation	−1.03±0.045	−0.93±0.094	−0.64±0.02	−0.22±0.029	−0.56±0.117	−0.25±0.061	−0.66±0.085	−0.27±0.142	−0.67±0.084	−0.35±0.058	−0.3 q <1.3E-5*
OSBPL3	oxysterol binding protein-like 3	LXR/RXR activation	−0.85±0.037	−0.71±0.066	−0.31±0.083	−0.33±0.049	−0.34±0.171	0.34±0.134	−0.14±0.043	0.33±0.082	0.4±0.045	0.77±0.059	−0.33 q <0.03*
OSBPL9	oxysterol binding protein-like 9	LXR/RXR activation	2.61±0.174	2.94±0.238	1.76±0.055	2.24±0.205	2.01±0.065	2.26±0.31	2.13±0.011	2.33±0.061	1.75±0.097	2.13±0.124	−0.31 q <4E-7*
PLTP	phospholipid transfer protein	LXR/RXR activation	0.25±0.055	0.5±0.121	−0.07±0.028	0.78±0.317	−0.06±0.09	0.36±0.409	0.54±0.291	0.46±0.147	−0.35±0.132	0.1±0.022	−1.3 q <0.05*
PROM2	prominin 2	LXR/RXR activation	0.74±0.277	0.62±0.043	2.63±0.198	2.71±0.073	2.83±0.322	2.07±0.173	2.33±0.111	1.98±0.085	1.68±0.131	1.14±0.197	0.34 q <0.04*
RXRG	retinoid x receptor, gamma	LXR/RXR activation	0.02±0.021	0.02±0.045	0.08±0.022	−0.07±0.044	0.04±0.108	−0.35±0.136	−0.38±0.094	−0.42±0.029	−0.28±0.076	−0.35±0.044	1.15 q <3E-3*
STARD3	star-related lipid transfer (start) domain containing 3	LXR/RXR activation	0.87±0.018	0.91±0.045	1.25±0.017	1.61±0.079	1.48±0.182	1.47±0.087	1.05±0.036	1.19±0.112	0.9±0.088	1.08±0.099	−0.14 q <0.09
TNFRSF1B	tumor necrosis factor receptor superfamily, member 1b	LXR/RXR activation	0.74±0.057	0.85±0.095	0.67±0.069	0.84±0.05	0.83±0.043	1.03±0.15	1.02±0.062	1.13±0.068	1.4±0.033	1.79±0.176	−1.14 q <0.03*

* Denotes significant at adjusted q-value <0.05.

Placental differences in cholesterol homeostasis through transcriptional activation programs, transport mechanisms and membrane specialization were also revealed by pathway analysis (Figure S2). Transcriptional control of cholesterol metabolism is mediated in part by sterol regulatory element binding proteins (SREBP), e.g. *SREBF2* (q <0.02; Table 5), in which binding of the cholesterol ligand yields nuclear translocation and de novo transcription at sterol consensus binding sequence target genes [75]. Cholesterol metabolism, reverse cholesterol transport, lipoprotein remodeling, lipogenesis and cholesterol efflux are controlled in part by modulating transcriptional activation of the nuclear liver × receptor (LXR) and retinoic acid (RXR) complex (p<0.05; Table 4, Figure S2) [75]–[76].

### 6 RT-qPCR and Biochemical Analyses Support Differences in Cholesterol Biosynthesis

To confirm that the cholesterol synthesis pathway was affected, we analyzed a subset of cholesterol genes in the D65 samples by RT-qPCR and as shown in Figure 4B, the data supports GO and pathway analyses. Moreover, the observed upregulation at D65 in the Meishan was not due to the presence of the single male placental sample (D65_MS_B) as the RT-qPCR results showed that this sample was not an outlier. This observation is also supported by the similar variances between the Meishan and WC samples shown in Figure 4B. Additionally, to more clearly visualize cholesterol biosynthetic changes throughout gestation in each of the two breeds, we plotted normalized expression of the different cholesterol pathway enzymes over time (gestational interval) and observed upregulation of cholesterol synthetic genes between D45 and D65 in the Meishan placentae (Figure 5).

**Figure 6:**
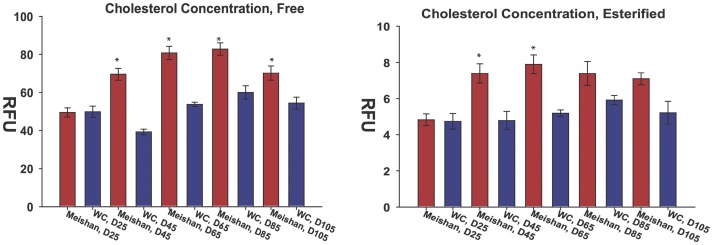
Biochemical analysis of cholesterol concentrations in swine placentae. (A) Free and (B) esterified cholesterol concentrations were measured in swine placentae by the Amplex Red assay at each gestational interval. No differences in free or esterfied cholesterol concentrations were observed at D25. At D45, both free and esterified cholesterol levels showed significant differences. These differences in cholesterol concentration by breed were maintained throughout gestation at the sampled time points. Error bars reflect standard error of the mean for six placental samples with three repeated measurements of the same group (technical replicates).

We next measured free and esterified cholesterol levels in placental tissue homogenates by a fluorometric Amplex Red assay. Cholesterol concentrations were similar at D25 for both breeds. However, increased cholesterol production in the Meishan placental tissues was detected at D45 and continued throughout gestation (Figure 6).

### 7 Extraction of Endothelial Biomarkers from Array Datasets to Assess Breed-specific Placental Vascularity Differences

**Figure 7:**
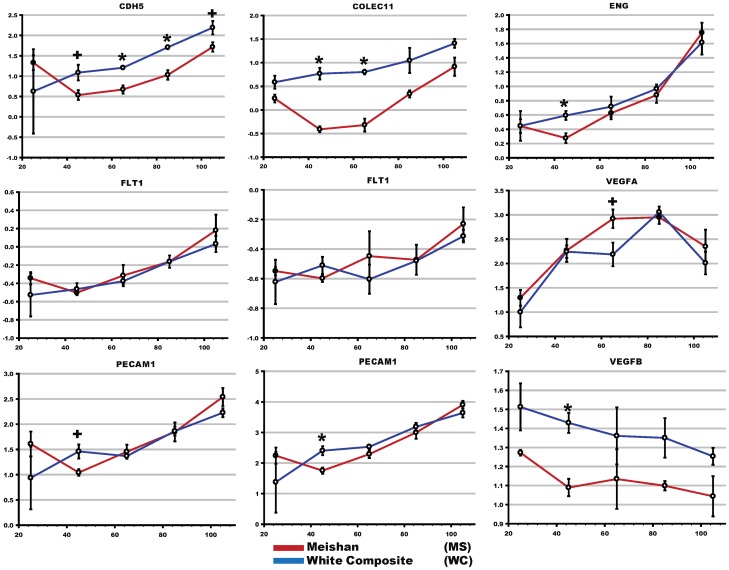
Differential expression of endothelial markers across gestation in swine placentae. Canonical biomarkers specific to endothelial cells were used as a surrogate measure of placental vascularity. Biomarkers are plotted by gestational time with respect to normalized expression. Asterisks denote corrected multiple-testing significance (p<0.05) and crosses denote a trend (p<0.1). Multiple plots are shown for *FLT1* and *PECAM*, and this reflects the gene estimate measurements for each of the multiple probe sets printed on the short-oligonucleotide array.

As shown in Figure 7, endothelial cell markers increased during gestation as would be expected due to increased placental vascularization as the pregnancy progresses. Differences (*COLEC11* (p<0.01), *ENG* (p<0.03), *PECAM1* (p<0.03) and a trend towards significance of *CDH5* (p<0.08) were observed at D45 and D65 with increased expression in the White Composite compared to Meishan.

## Discussion

In order to identify fundamental differences in gene expression patterns between the WC and Meishan breed of swine we compared their transcriptome throughout gestation. Linear mixed models analysis looking at breed effects identified 1,595 differentially expressed genes at q<0.05. A shown in Fig. 1, *XIST* was highly down regulated in the Meishan breed. As *XIST* is responsible for epigenetic silencing of one female X-chromosome, which results in chromosomal dosage compensation [77], we examined the expression of other X-linked genes and found no evidence for abnormal X-chromosome inactivation. We followed up this observation with a series of PCR assays that spanned the length of both genomic and RNA *XIST* biotypes and concluded from these experiments that (1) the A-repeat element is expressed in both breeds of swine placentae; (2) breed-specific *XIST* isoforms are readily detected by microarray and PCR methods; and (3) the breed-specific isoforms are not due to structural breed-specific differences in the *XIST* locus (Figure 2). While aberrant *Xist* expression can affect developmental outcomes, as has been shown in mouse embryos that ectopically expressed *Xist* from the active X chromosome after nuclear reprogramming by somatic cell nuclear transfer [78], it is not known what developmental outcome expression of different *Xist* isoforms may have. In mice, two distinct and developmentally regulated *Xist* isoforms (referred to as large and small) have been identified that differ in their 3' end [79]. In mice, it is the large *Xist* form that seems to play a key role in embryonic and fetal X-inactivation. What is unusual in these two pig breeds is that the two isoforms are not developmentally regulated but are breed-specific. That is, the large *XIST* form is unique to the White composite, and is expressed throughout gestation, not just at a specific developmental time point (Table 1). While we have no direct evidence that the two isoforms lead to differences in X-inactivation, the DIGMAP data is suggestive of differences in X-chromosome behavior in the placenta of the two breeds. We described three chromosomal bands on *Sus scrofa* X (Xq13, Xq21, Xp11) that were significantly different between the two breeds. Additionally, genetic crosses between the Meishan and the WC support X-chromosome transcriptional differences [12], [69], further reinforcing our own observations.

The imprinted gene family represents a unique cluster of genes that broadly contribute to mammalian developmental potential, fetal growth and normal physiology of the placenta. Although the biological functions of imprinted genes range diversely from growth factors to transcription factors, many function to regulate fetal and placental growth and often lead to embryonic lethality when inactivated by knockout gene-targeting studies. Indeed, genetic rescue in *trans* of a disrupted imprinting control region completely ameliorated placental defects (placentomegaly). Because imprinted genes collectively play critical roles in feto-placental development, we reasoned their expression pattern might be particularly important during gestation between the MS versus WC breeds. A recent study by Zhou *et al* 2009 [80] comparing placental transcriptome profiles at D75 and D90 of gestation between the prolific Chinese indigenous Erhualian versus Western composite breed identified several differentially expressed imprinted genes *DIRAS3*, *DIO3*, *NAP1L5*, *PON2*, *PLAGL1* and *SDHD*. Taken together both functional and expression profiling studies of imprinted genes warrant their relevancy for targeted exploratory analysis in our placental transcriptome datasets. Expression data presented in Table S2, survey imprinted genes that met significance criteria at q <0.05. Breed specific differences were observed for several imprinted genes. Three paternally expressed genes, *NAP1L5*, *SNORD107*, *SNRPN* and the maternally expressed *PHLDA2* showed significantly higher expression in Meishan placentae. In WC placentae, significantly higher expression of paternally expressed *IGF2*, *INPP5F*, *MEST*, *PEG10*, *PEG3* and maternally expressed *IGF2R, MEG3*, *OSBPL1A* were observed.

**Figure 8:**
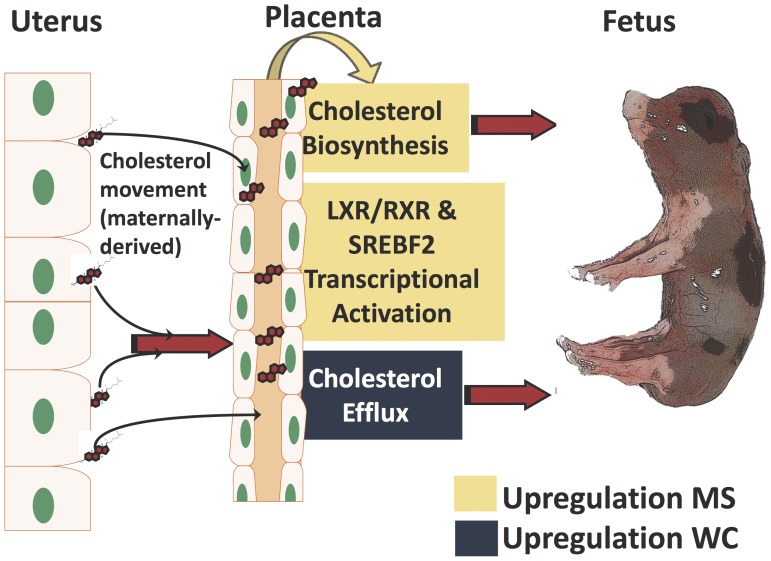
Model of cholesterol utilization in swine placentae. Combined, our results support differential cholesterol synthesis, transport and transcriptional activation in the placentae of two breeds of swine. Specifically, our results predict 1) increased cholesterol biosynthetic activity in Meishan placentae, 2) increased cholesterol efflux by transporters *ABCA1* towards the fetal blood lumen in WC placentae and 3) increased gene expression by transcriptional activation of cholesterol enzymes mediated in part by SRE-binding proteins and RXR/LXR signaling in Meishan.

In addition to differences in behavior of X-chromosome linked genes and imprinted genes, lipid and cholesterol metabolism, cholesterol transcriptional activation and transport were identified as being different between the two breeds and forms the basis for the model presented in Figure 8. Placental synthesis, transcriptional activation, and transport of cholesterol differ between breeds of swine. We propose a model of differential cholesterol utilization in the placentae of Meishan and White Composite swine breeds (Figure 8). Specifically, the model predicts:

### 

#### 1) Increased cholesterol biosynthetic activity in Meishan placentae

Evidence for the increased synthesis of cholesterol in Meishan placentae is supported by microarray observations, RT-qPCR, pathway analyses and biochemical determination of cholesterol levels. Cholesterol metabolic genes were upregulated by D65 and point to increased biosynthetic flux of cholesterol consistent with microarray and RT-qPCR findings (Figures 4, 5). Additionally, free and esterified cholesterol concentration differences support increased activity in Meishan placentas by D45, and these increased levels are maintained throughout gestation (Figure 6). While we have not measured cholesterol intermediates and oxidation products (oxysterols), these may refine or clarify differences in cholesterol signaling between swine breeds. Functional studies using small molecule inhibitors that selectively target synthetic enzymes of cholesterol metabolic enzymes such as squalene synthase, e.g. FsPP, BPH-652, BPH-698, BPH-700, may also lend clues to these differences.

#### 2) Differences in transport or kinetics of cholesterol efflux partially compensate for reduced local synthesis routes in WC placentae

Transport of cholesterol by efflux and intracellular mechanisms differs between swine breeds. In contrast to Meishans where cholesterol is locally synthesized in the placenta, our data supports increased ABCA1 activity in WC placentae. Why might transport be different in the swine placentae? We hypothesize that upregulation of *ABCA1* in WC placentae enhances the kinetics of efflux of maternally-derived cholesterol; that is, as cholesterol diffuses or is moved across the endometrium into the fetal side, ABCA1 may serve as an alternative route to partially compensate for reduced local placental cholesterol synthesis. While there is conflicting evidence in the literature with respect to human trophoblastic ABCA1 subcellular localization and its function in maternal-fetal cholesterol efflux [81], treatment with the ABCA1-inhibitor glyburide decreased cholesterol efflux relative to controls [82]. Additionally, small molecule complementation with a LXR agonist can induce *Abca1*’s expression in wild-type mouse littermates, and increase rates of maternal-fetal cholesterol transfer to the fetus [83]. Our data also points to differences in intracellular movement of cholesterol. Movement of cholesterol out of late endosomes is mediated by *NPC2*; this was downregulated (q <4.0×10^−4^; Table 5, Figure S2) in the Meishan placentae. Shuttling cholesterol between the plasma membrane and endoplasmic reticulum is mediated in part by the oxysterol-binding protein *OSPBL3* (q <4.0×10^−4^; Table 5, Figure S2), and this transport mode is reduced in Meishans. Curiously, subcellular immuno-staining of human ABCA1 in larger trophoblast villi also localized the protein to the endoplasmic reticulum [81] and implicated ABCA1 as a mediator to expel cytotoxic oxysterols from the placenta [82]. Placental trophoblast cells may use additional modes of cholesterol efflux including secretion through complexing of apolipoproteins or lipoproteins, and we document differences in apolipoprotein remodelers, e.g. *LPL*, *LCLAT1*, *PLTP* (Table 5, Figure S2) [84].

#### 3) Differences in transcriptional circuits for cholesterol synthesis and movement between swine breeds

Genome-wide expression profiling revealed striking differences in cholesterol synthetic and transport enzymes, and this begs the question: is cholesterol homeostasis in the placentae differently regulated at the transcriptional level? Indeed, we observed upregulation in sterol response binding transcription factor *SREBF2* (q <0.02; Table 5, Figure S2) that facilitates transcriptional activation of cholesterol metabolic enzymes. Supporting this view, we also documented upregulation of the entire suite of cholesterol biosynthetic enzymes (except the notable exception *DHCR7*), presumably mediated through upregulation of *SREBF2*. Cholesterol efflux is coordinated, in part, by transcriptional activation of the nuclear liver X receptor and (LXR) and retinoic acid (RXR) complex (p<0.05; Table 4, Figure S2). Differences in hetero- and homo-dimerization partners of LXR and RXR isotypes as well as ligand binding are implicated in the wide ranging physiological processes of reverse cholesterol transport, lipoprotein remodeling, lipogenesis, and cholesterol efflux among others [75], [76]. Additionally, recent biochemical studies support a role of transcriptional regulation by *TACC1* (highly expressed in MS placentas), and its interaction with nuclear receptors devoid of their respective ligands (aporeceptors) including AR, RXRα, RARα, PPARγ, ERα, GR, TRα1 and TRα2 [85]. In short, mechanisms that regulate proper cholesterol homeostasis via transport and biosynthesis are crucial to reproductive fitness [86]. The ability to manipulate the flux of cholesterol from mother to fetus and modulate local biosynthetic routes in the placenta could improve fetal growth trajectories, enhance pregnancy outcomes, and reduce neonatal loss [87], [88].

Finally, previous studies suggested that Meishan enhanced placental efficiency compared to occidental breeds may be due to increased vascularity [89], [90]. Concordant with these reports, recent experiments carried out on the placentas of Taihu pig strains (Meishan and Erhualian) and comparison to Western breeds also support increased placental angiogenesis. For example, a gene expression survey of D75 and D90 placentae from the prolific Chinese Erhualian breed as compared to the Large White reported that *VEGF* pathway genes responsible for angiogenesis were overrepresented in Erhualian placentae [80]. Wu et al, 2009 reported similar increases for *VEGF* signal transduction genes in Erhualians, but observed a decrease in vascular endothelial cadherin (*CDH5*) and *β*-arrestin 2 (*ARRB2*) when compared to Landrace breeds [27]. The swine placenta is composed of multiple cell types including trophoblast epithelial cells that form the chorionic bilayer and endothelial cells that comprise blood capillaries and line blood vessels. Analysis of multiple endothelial markers, e.g. *COLEC11, ENG, PECAM1, CDH5*, extracted from our transcriptome datasets indicated higher expression levels in the White Composite compared to Meishan. In addition to extracting these biomarkers, we analyzed *VEGFA*, *VEGFB*, *VEGFC*, the VEGF receptor *FLT1*. Later stages of gestation in both breeds had higher total amounts of endothelial cell markers (*CDH5*, *ENG*) which we infer to have increased amounts of vascularity. At D25 no differences were observed in either breed; however, at D45 breed vascularity markers became apparent with significant upregulation in WC of *ENG* (p<0.03) and a trend towards significance of CDH5 (p<0.08). Upregulation of CDH5 was noted in WC in D65 and D85 gestations and a trend in D105 gestations; in comparison, ENG did not exhibit breed specific differences in subsequent gestational time points. Furthermore, no statistical differences were observed for the vascular endothelial growth factor receptor 1 also known as *FLT1* or *VEGFA* (See Figure 7) and *VEGFC* (data not shown). *VEGFB* was expressed higher in WC (−1.2, q <0.01; data not shown), but its expression decreased throughout gestation. Overall, however, our data does not support increased vascularity in the Meishan placenta as has been reported previously (Figure 7).

## Summary

We sought to investigate gene expression differences between commercial swine populations and the Chinese Meishan placentae to potentially uncover candidates for placental efficiency [91]. Our findings include differences in *XIST* isoforms expression between the two breeds, differences in X-chromosome gene expression as identified by DIGMAP, and marked differences in lipid and cholesterol biosynthesis and transport between the two breeds. We have also confirmed these results by quantitative real-time PCR, and directly measured physiological concentrations of cholesterol. Specifically, these analyses reveal a number of common and unique candidate genes that may confer enhanced placental efficiency through modulation of steroid biosynthetic pathways. This report provides information to target physiological studies in any swine population to see if modulation of cholesterol biosynthetic pathways can favorably influence placental efficiency and fetal survival.

## Supporting Information

Figure S1 **2-Dimensional PCA of swine placental changes at 20 day gestational intervals.** To scrutinize the behavior of individual microarrays, we used mathematical deconstruction by principal component analysis in order to visualize global changes of gene expression throughout gestation in swine placentae. The distance or proximity of each plot to neighboring plots indicates relative similarity. Ellipses were manually drawn to better visualize intra-sample variation for breed and gestational day.
(TIFF)

Figure S2 **Comparison of LXR/RXR and** ***SREBF2*** **signaling cascade in swine D65 placentae from WC and Meishan.** Pathways analysis facilitated the identification of sterol transcriptional activation circuits previously unrealized by gene ontology analysis. The diagram depicts gene expression breed differences in swine placentae of the LXR/RXR and *SREBF2* signaling cascades. The blue to yellow color intensity denotes downregulation in Meishan (blue) or upregulation in Meishan (yellow). Cholesterol metabolism, reverse cholesterol transport, lipoprotein remodeling, lipogenesis, and cholesterol efflux are controlled in part by modulating transcriptional activation of the LXR/RXR complex. In the presence of agonists including oxysterols and 9-cis-retinoic acid, transrepression mediated by NCORs is overcome to produce mRNAs of LXR/RXR target genes. A downstream target of LXR/RXR transcriptional activation is *ABCA1* and this transmembrane protein is responsible for movement of cholesterol out of the trophoblast (efflux) to HDL. Coincident with this, lipoprotein remodeling proteins that alter the discoid to spherical shape of HDL and intracellular cholesterol transporters e.g. *NPC2*, *OSBPL1A*, *OSBPL3* and *STARD3*, are also affected indicative of LXR/RXR transcriptional activation. Regulation of the cholesterol biosynthetic pathway is controlled in part by transcriptional activation of sterol binding protein. *SREBF2* is upregulated in Meishans and may explain why the cholesterol synthetic enzymes are overexpressed in Meishan placentae. A description of IPA symbols is provided in Figure S3.
(TIFF)

Figure S3 **Symbols used in Ingenuity Pathway Analyses.**
(TIFF)

Table S1 Primers used in this study for RT-qPCR and identifying *XIST* structure.
(DOCX)

Table S2 Summary of placental gene expression differences.
(PDF)
