# Supplementary figures and images for: Differences in X-Chromosome Transcriptional Activity and Cholesterol Metabolism between Placentae from Swine Breeds from Asian and Western Origins
Source: PLoS One. 2013 Jan 31;8(1):e55345. doi: 10.1371/journal.pone.0055345 (PMC3561265; doi:10.1371/journal.pone.0055345)

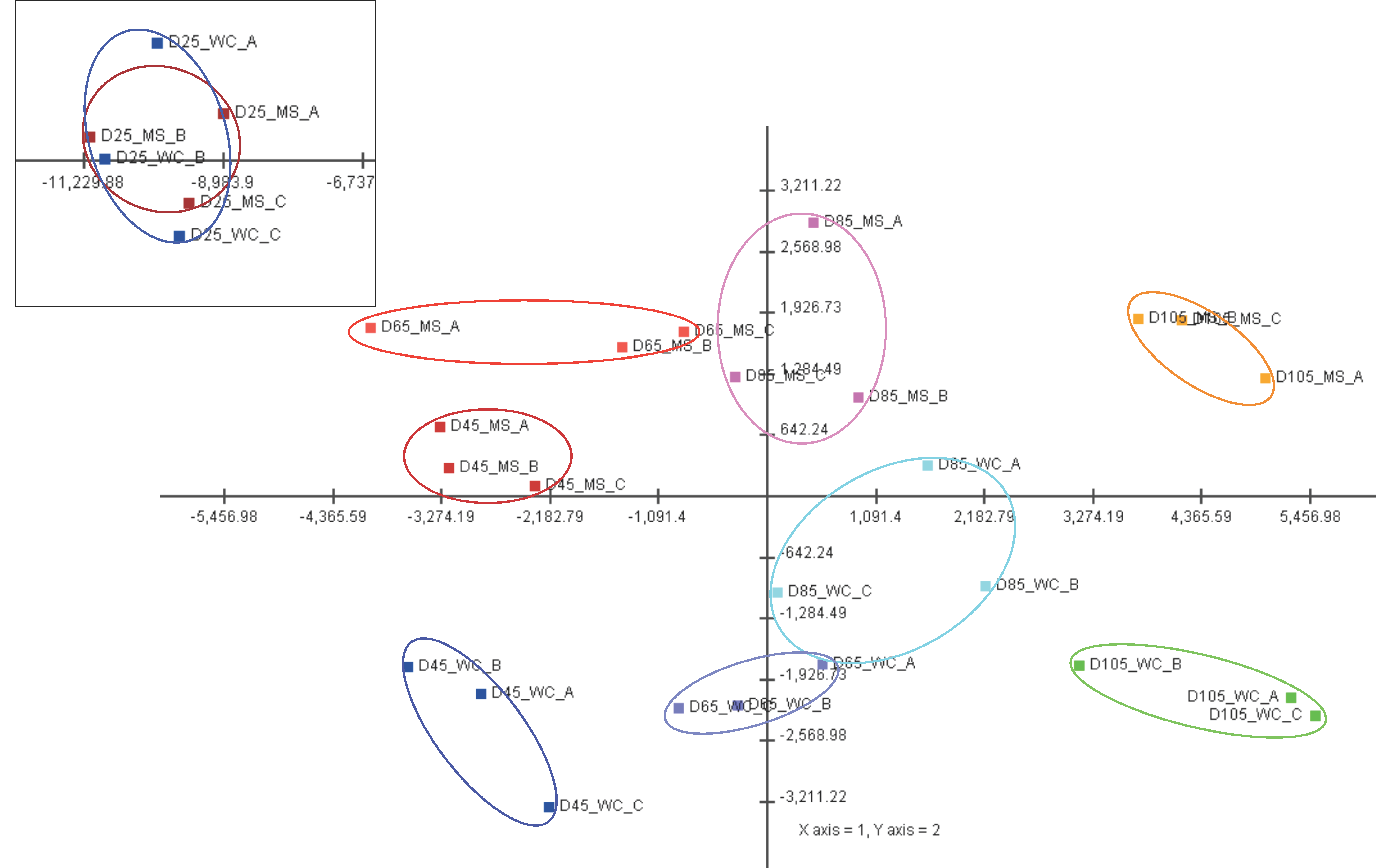

Supplement: Figure S1 — 2-Dimensional PCA of swine placental changes at 20 day gestational intervals. To scrutinize the behavior of individual microarrays, we used mathematical deconstruction by principal component analysis in order to visualize global changes of gene expression throughout gestation in swine placentae. The distance or proximity of each plot to neighboring plots indicates relative similarity. Ellipses were manually drawn to better visualize intra-sample variation for breed and gestational day. (TIFF) [file pone.0055345.s001.tiff]

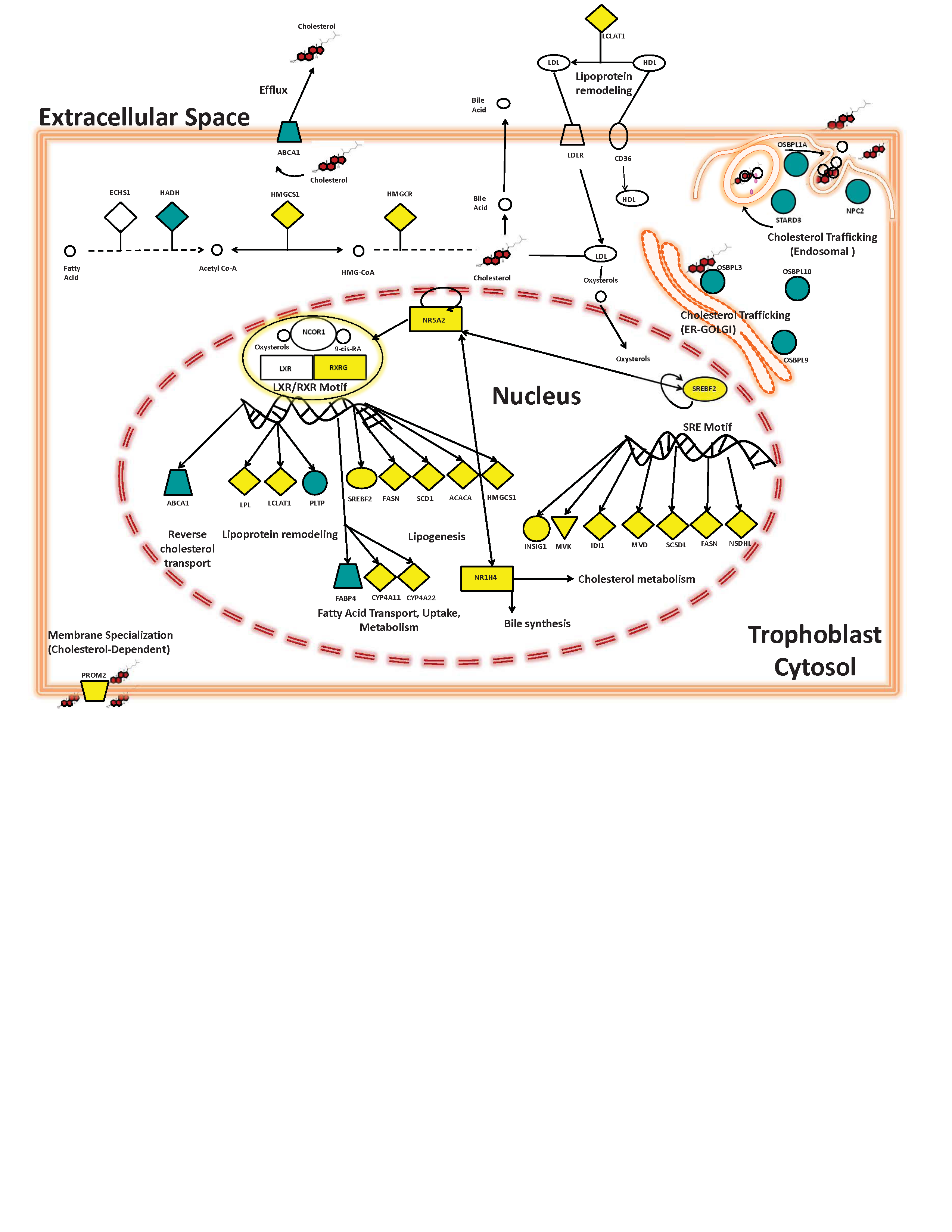

Supplement: Figure S2 — Comparison of LXR/RXR and SREBF2 signaling cascade in swine D65 placentae from WC and Meishan. Pathways analysis facilitated the identification of sterol transcriptional activation circuits previously unrealized by gene ontology analysis. The diagram depicts gene expression breed differences in swine placentae of the LXR/RXR and SREBF2 signaling cascades. The blue to yellow color intensity denotes downregulation in Meishan (blue) or upregulation in Meishan (yellow). Cholesterol metabolism, reverse cholesterol transport, lipoprotein remodeling, lipogenesis, and cholesterol efflux are controlled in part by modulating transcriptional activation of the LXR/RXR complex. In the presence of agonists including oxysterols and 9-cis-retinoic acid, transrepression mediated by NCORs is overcome to produce mRNAs of LXR/RXR target genes. A downstream target of LXR/RXR transcriptional activation is ABCA1 and this transmembrane protein is responsible for movement of cholesterol out of the trophoblast (efflux) to HDL. Coincident with this, lipoprotein remodeling proteins that alter the discoid to spherical shape of HDL and intracellular cholesterol transporters e.g. NPC2, OSBPL1A, OSBPL3 and STARD3, are also affected indicative of LXR/RXR transcriptional activation. Regulation of the cholesterol biosynthetic pathway is controlled in part by transcriptional activation of sterol binding protein. SREBF2 is upregulated in Meishans and may explain why the cholesterol synthetic enzymes are overexpressed in Meishan placentae. A description of IPA symbols is provided in Figure S3. (TIFF) [file pone.0055345.s002.tiff]

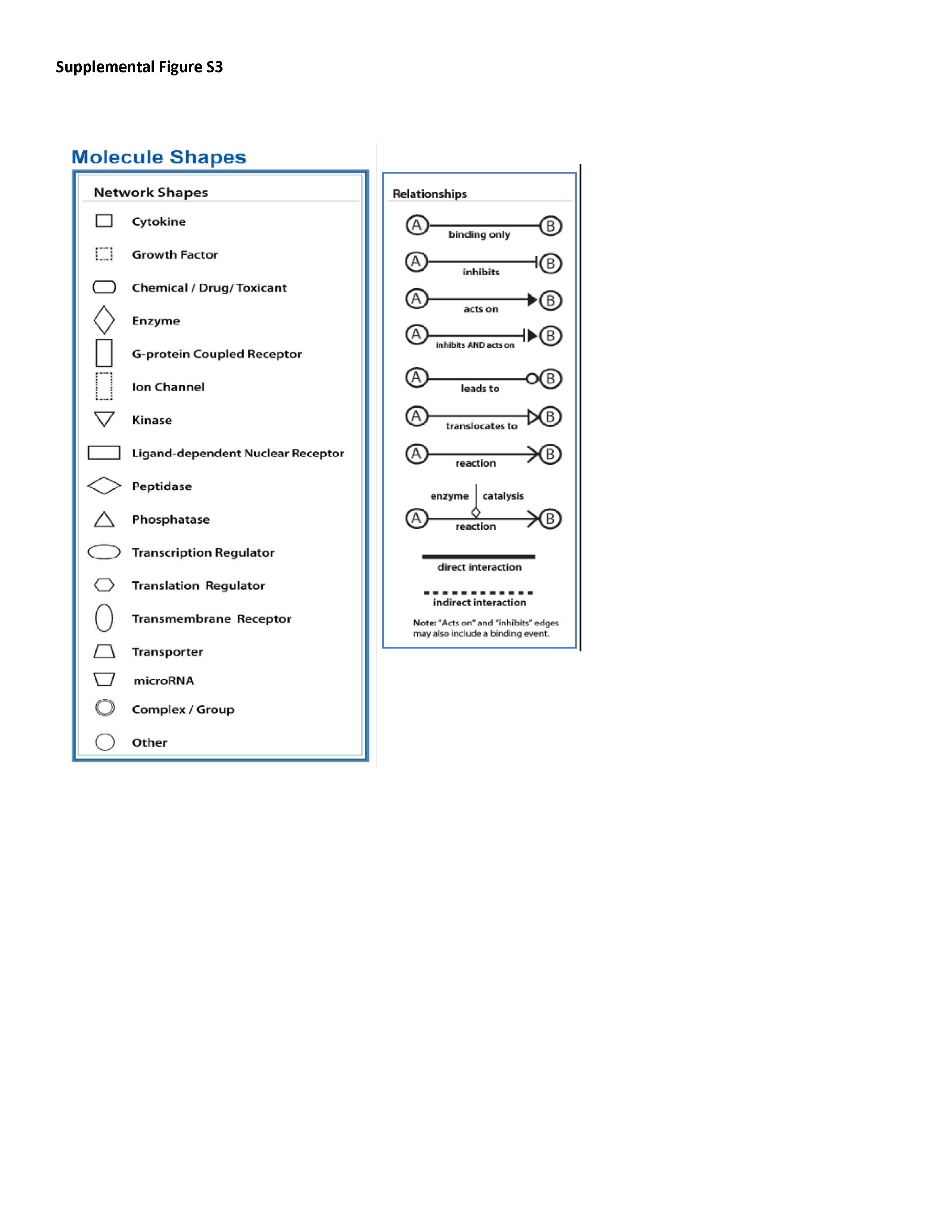

Supplement: Figure S3 — Symbols used in Ingenuity Pathway Analyses. (TIFF) [file pone.0055345.s003.tiff]
