# Supplementary material for: Differences in X-Chromosome Transcriptional Activity and Cholesterol Metabolism between Placentae from Swine Breeds from Asian and Western Origins
Source: PLoS One. 2013 Jan 31;8(1):e55345. doi: 10.1371/journal.pone.0055345 (PMC3561265; doi:10.1371/journal.pone.0055345)
Supplement: Table S1 — Primers used in this study for RT-qPCR and identifying XIST structure. (DOCX) [file pone.0055345.s004.docx]

**Table 1S. Primers used in this study for RT-qPCR and identifying *XIST* structure.**

| **Description** | **Sequence 5’🡪3’** |
| --- | --- |
| Zfx- AF132077 QPCR FWD-1 | GGTGGCACTGTGGACATTGTG |
| Zfx- AF132077 QPCR REV-1 | CCTTGGCACACGAATACTGCTATTC |
| RPL18_AK232122 FWD | ATAACCGATGACGTGCGTGT |
| RPL18_AK232122 REV | TAGGGTTTGGTGTGGCTGTG |
| XIST - TC316874 qPCR FWD | GGGACAGTTAATCCGCACAG |
| XIST - TC316874 qPCR REV | AAGGGTCCTCCTGACACACC |
| [XIST - TC323364 qPCR FWD](http://www.ncbi.nlm.nih.gov/UniGene/seq.cgi?ORG=Ssc&SID=35171791) | AATGGAATTGTGCAAGGCAGT |
| XIST - TC323364 qPCR REV | TCAATCAGGCAAAGGAGCAA |
| SIAH2 CF181241 FWD | ATTTGCGAACGGAACCATAA |
| SIAH2 CF181241 REV | GGTCTCCACCAGGTCAGTGT |
| SSFA2, pig XM_001928406 FWD | GAGGACCTGACCTTGCTGCT |
| SSFA2, pig XM_001928406 REV | GGTTCCGCCTCATTACTTGG |
| XIST CF175718 FWD | GATCCCATCCCTCCTACTGG |
| XIST CF175718 REV | CCCTGTACTCCCTTTGGTAATG |
| XIST BQ597566 FWD | GGGATCTGAGCCGCATCTAC |
| XIST BQ597566 REV | TGCCAGATAACATTCTAGGCATT |
| HMGCS1 BQ602833 FWD | AAGGCCACAGAGATCCTCCA |
| HMGCS1 BQ602833 REV | CCAAGATCATACCCAGGCTTC |
| IDI1 CO946661 FWD | ATGACGCGATTGGAGTGAGA |
| IDI1 CO946661 REV | GGGATCTGGATTCAGGGTTACA |
| MVK BI182626 FWD | AGAACATTCCTCCGGCTTCA |
| MVK BI182626 REV | CGGCAGATGGTCAGGTACAA |
| ABCA1 BI182779 FWD | ATTCAGCGTCCTTTCCCAGA |
| ABCA1 BI182779 REV | TCATCATCACTTTGGTCCTTG |
| TACC1 CN025137 FWD | TTGCTCGCAGACTCAGATGG |
| TACC1 CN025137REV | CGCCCAGATTATAGCTGTTCTT |
| FMN1 BI403565 FWD | GAATGAAGAGGTTCCCAAGGT |
| FMN1 BI403565 REV | CACCACAGATAGCAGCATAGCA |
| XIST_pig_ FWD | ATTCCTCTTCCGCCACCT |
| XIST_pig_ REV | CCCTCCAGCATTACCACCT |
| XIST_pig_ FWD2 | TAAGGATGGGAACAAAGCAAGA |
| XIST_pig_(A-repeat) FWD | ATACAAAGGTCCGATGGGTGATG |
| XIST_pig_(A-repeat) REV | TTTGGTTGACTCTTCTGGTTT |
| XIST_pig_(A-repeat) REV2 | CAGGGTAATGGATACCTGCT |
| XIST_pig_ BP437119 FWD | GGGTATGACAACTGGGACTGCT |
| XIST_pig_ BP437119 REV | CATCTCAATCTCCTGCCTTT |
| XIST_pig_ AK238028 FWD | GTGACGTACTAGCAACG TCCCTTGTATCCTCCCTTGT |
| XIST_pig_ AK238028 REV | TAGCAGGATACGACTATC ATGCCCTCCTACTCCATTT |
| XIST_pig_ AK238028 SNP Y PSQ-F | TAGGCCCATTAACAGTCCC |
| RPS20 + Ssc.20036.1.S1_at | ATTCGGATCACCCTCACC |
| RPS20 - Ssc.20036.1.S1_at | TTCTTCTCCTTCGCGCCTCT |
