# Supplementary material for: Differences in X-Chromosome Transcriptional Activity and Cholesterol Metabolism between Placentae from Swine Breeds from Asian and Western Origins
Source: PLoS One. 2013 Jan 31;8(1):e55345. doi: 10.1371/journal.pone.0055345 (PMC3561265; doi:10.1371/journal.pone.0055345)
Supplement: Table S2 — Summary of placental gene expression differences. (PDF) [file pone.0055345.s005.pdf]

Supplemental Table S2. Summary of placental gene expression differences

Gene Name	Gene Description	Probe Set ID	Breed MS-WC	Day 25 MS-WC	Day 45 MS-WC	Day 65 MS-WC	Day 85 MS-WC	Day 105 MS-WC
ABCA1	ATP-binding cassette, sub-family A (ABC1), member 1	Ssc.7146.1.A1_at	-3.43 q < 0	-2.54 q < 0	-3.37 q < 0	-4.05 q < 0	-3.96 q < 0	-3.47 q < 0
ALDH7A1	aldehyde dehydrogenase 7 family, member A1	Ssc.17284.1.A1_at	-1.74 q < 0	-1.67 q < 0.000225	-2.06 q < 0.0000000116	-1.57 q < 0.00184	-1.71 q < 0.000152	-1.71 q < 0.0000725
ALDH7A1	aldehyde dehydrogenase 7 family, member A1	Ssc.11751.1.S1_at	-1.11 q < 0.232	-1.06 q < 1	-1.45 q < 0.039	1.07 q < 0.79	-1.03 q < 1	-1.15 q < 1
CCL28	chemokine (C-C motif) ligand 28	Ssc.7568.1.A1_at	-1.71 q < 0	-2.39 q < 0.00000000991	-1.55 q < 0.0282	-1.51 q < 0.0322	-1.58 q < 0.0299	-1.63 q < 0.00825
CPSF3	cleavage and polyadenylation specific factor 3, 73kDa	Ssc.786.1.S1_at	-1.58 q < 0	-1.58 q < 0.000123	-1.64 q < 0.0000148	-1.53 q < 0.000306	-1.57 q < 0.00019	-1.59 q < 0.0000573
CXorf39	family with sequence similarity 199, X-linked	Ssc.3940.2.A1_at	-1.73 q < 0	-1.83 q < 0.0000000167	-1.87 q < 0.00000000702	-1.71 q < 0.00000125	-1.76 q < 0.000000375	-1.53 q < 0.000361
CXorf39	family with sequence similarity 199, X-linked	Ssc.3940.1.S1_at	-1.45 q < 0	-1.51 q < 0.000303	-1.45 q < 0.00142	-1.35 q < 0.0147	-1.52 q < 0.000216	-1.42 q < 0.00319
EXOC2	exocyst complex component 2	Ssc.1468.1.S1_at	-1.55 q < 0	-1.53 q < 0.0146	-1.74 q < 0.000246	-1.45 q < 0.0372	-1.56 q < 0.0113	-1.48 q < 0.0312
FAM105B	family with sequence similarity 105, member B	Ssc.31074.1.A1_at	-1.37 q < 0	-1.31 q < 0.00722	-1.44 q < 0.0000526	-1.37 q < 0.000741	-1.39 q < 0.000503	-1.32 q < 0.00629
FAM105B	family with sequence similarity 105, member B	Ssc.25709.1.A1_at	-1.14 q < 0.0135	-1.16 q < 0.852	-1.27 q < 0.157	-1.07 q < 0.738	-1.12 q < 1	-1.09 q < 1
FAM118A	family with sequence similarity 118, member A	Ssc.2869.1.S1_at	-1.56 q < 0	-1.38 q < 0.00241	-1.61 q < 0.000000398	-1.58 q < 0.000001	-1.52 q < 0.0000206	-1.74 q < 0.000000000484
GRN	granulin	Ssc.20986.2.S1_at	-2.12 q < 0	-2.1 q < 0.00000266	-2.93 q < 0	-1.84 q < 0.000232	-1.89 q < 0.000188	-2 q < 0.0000135
HLA-B	major histocompatibility complex, class I, B	Ssc.18553.1.S1_at	1.65 q < 0	1.69 q < 0.00000198	1.6 q < 0.000039	1.46 q < 0.00266	1.46 q < 0.00335	2.12 q < 0
HLA-B	major histocompatibility complex, class I, B	Ssc.13780.12.S1_x_at	1.76 q < 0.0000000246	1.69 q < 0.229	1.92 q < 0.0449	1.55 q < 0.246	2.34 q < 0.00367	1.45 q < 0.747
HLA-B	major histocompatibility complex, class I, B	Ssc.13780.12.S1_a_at	1.6 q < 0.00312	1.79 q < 0.527	2.2 q < 0.126	1.37 q < 0.611	1.89 q < 0.686	1.03 q < 1
HLA-B	major histocompatibility complex, class I, B	Ssc.13780.6.S1_a_at	-1.28 q < 0.0193	-1.47 q < 0.432	-1.38 q < 0.521	-1.06 q < 0.901	-1.33 q < 1	-1.19 q < 1
HNRNPH2	heterogeneous nuclear ribonucleoprotein H2 (H')	Ssc.3216.2.A1_at	-1.89 q < 0	-2.46 q < 0	-1.96 q < 0.00000000702	-1.62 q < 0.000135	-1.83 q < 0.000000575	-1.67 q < 0.0000336
HNRNPH2	heterogeneous nuclear ribonucleoprotein H2 (H')	Ssc.3216.1.A1_at	-1.29 q < 0.000126	-1.32 q < 0.399	-1.49 q < 0.0441	-1.01 q < 0.969	-1.35 q < 0.495	-1.3 q < 0.515
IAH1	isoamyl acetate-hydrolyzing esterase 1 homolog (S. cerevisiae)	Ssc.2629.1.S1_at	-1.52 q < 0	-1.44 q < 0.0285	-1.56 q < 0.00271	-1.48 q < 0.0106	-1.61 q < 0.00115	-1.49 q < 0.0101
KCND3	potassium voltage-gated channel, Shal-related subfamily, member 3	Ssc.1445.1.S1_at	-1.46 q < 0	-1.52 q < 0.0000836	-1.55 q < 0.00003	-1.44 q < 0.000784	-1.39 q < 0.00719	-1.41 q < 0.00271
PCDH15	protocadherin-related 15	Ssc.30063.1.A1_at	-2.75 q < 0	-2.39 q < 0.000198	-2.56 q < 0.0000374	-3.09 q < 0.000000178	-4.35 q < 0.00000000000179	-1.93 q < 0.0142
PXDN	peroxidasin homolog (Drosophila)	Ssc.3128.1.A1_at	-1.88 q < 0	-1.73 q < 0.000716	-2.06 q < 0.00000105	-1.75 q < 0.000409	-1.75 q < 0.000733	-2.16 q < 0.0000000954
RAB18	RAB18, member RAS oncogene family	Ssc.6119.1.A1_at	-1.74 q < 0	-1.85 q < 0.00000159	-1.86 q < 0.00000125	-1.69 q < 0.0000784	-1.43 q < 0.05	-1.92 q < 0.000000196
RAB18	RAB18, member RAS oncogene family	Ssc.3766.1.S1_at	-1.35 q < 0.000000000000369	-1.42 q < 0.00289	-1.45 q < 0.00126	-1.23 q < 0.145	-1.23 q < 0.519	-1.46 q < 0.000744
RBMS3	RNA binding motif, single stranded interacting protein 3	Ssc.12996.1.A1_at	2.16 q < 0	1.47 q < 0.0259	1.52 q < 0.00891	3.15 q < 0	3.18 q < 0	2.08 q < 0.0000000168
SLC12A1	solute carrier family 12 (sodium/potassium/chloride transporters), member 1	Ssc.10238.1.A1_at	-1.51 q < 0	-1.48 q < 0.0000507	-1.59 q < 0.000000714	-1.34 q < 0.00682	-1.67 q < 0.0000000178	-1.5 q < 0.000022
SLC16A1	solute carrier family 16, member 1 (monocarboxylic acid transporter 1)	Ssc.20473.2.S1_at	-1.55 q < 0	-1.64 q < 0.000162	-1.43 q < 0.0223	-1.45 q < 0.0118	-1.79 q < 0.00000404	-1.48 q < 0.00808
SLC25A17	solute carrier family 25 (mitochondrial carrier; peroxisomal membrane protein, 34kDa), member 17	Ssc.4477.1.S1_at	-2.07 q < 0	-1.85 q < 0.00104	-2.21 q < 0.00000346	-2.08 q < 0.0000243	-1.94 q < 0.000353	-2.33 q < 0.000000307
TACC1	transforming, acidic coiled-coil containing protein 1	Ssc.28305.1.A1_at	2.29 q < 0	2.27 q < 0	1.72 q < 0.00000229	2.6 q < 0	2.6 q < 0	2.39 q < 0
TACC1	transforming, acidic coiled-coil containing protein 1	Ssc.7712.1.A1_at	-1.72 q < 0.00000313	-1.58 q < 0.536	-1.99 q < 0.0725	-1.51 q < 0.353	-2.13 q < 0.0587	-1.47 q < 0.86
XIST	X (inactive)-specific transcript (non-protein coding)	Ssc.31029.1.A1_at	-7.15 q < 0	-8.29 q < 0.0000000227	-8.94 q < 0.00000000702	-3.96 q < 0.00127	-9.23 q < 0.00000000418	-6.91 q < 0.000000413
XIST	X (inactive)-specific transcript (non-protein coding)	Ssc.2434.1.A1_at	-1.65 q < 0	-1.95 q < 0.00000000076	-1.81 q < 0.0000000826	-1.37 q < 0.0217	-1.72 q < 0.00000224	-1.48 q < 0.00212
XIST	X (inactive)-specific transcript (non-protein coding)	Ssc.13426.1.A1_at	-2.35 q < 0.000000000000191	-3.7 q < 0.00000531	-2.95 q < 0.00044	-1.51 q < 0.375	-2.39 q < 0.0167	-1.82 q < 0.234
A2M	alpha-2-macroglobulin	Ssc.16603.1.A1_at	-2.29 q < 0	-2.31 q < 0.00124	-2.63 q < 0.0000743	-2.77 q < 0.0000176	-1.81 q < 0.115	-2.07 q < 0.00882
A2M	alpha-2-macroglobulin	Ssc.26317.1.S1_at	-1.63 q < 0.0396	-2.19 q < 0.515	-2.12 q < 0.452	-1.66 q < 0.53	-1.04 q < 1	-1.4 q < 1
C20orf108	chromosome 20 open reading frame 108	Ssc.19192.2.A1_at	-1.41 q < 0	1.05 q < 1	-1.95 q < 0.0000000000352	-1.37 q < 0.0107	-1.52 q < 0.000288	-1.45 q < 0.00154
DYNLL2	dynein, light chain, LC8-type 2	Ssc.12207.1.A1_at	1.36 q < 0	1.46 q < 0.000489	1.34 q < 0.0188	1.3 q < 0.0391	1.3 q < 0.0832	1.43 q < 0.00105
FMN1	formin 1	Ssc.12769.1.A1_at	2.12 q < 0	1.06 q < 1	2.8 q < 0	2.43 q < 0.000000000382	2.33 q < 0.00000000356	2.52 q < 0.0000000000234

Gene Name	Gene Description	Probe Set ID	Breed MS-WC	Day 25 MS-WC	Day 45 MS-WC	Day 65 MS-WC	Day 85 MS-WC	Day 105 MS-WC
IL2RG	interleukin 2 receptor, gamma	Ssc.15739.1.S1_at	-1.8 q < 0	-1.09 q < 1	-1.72 q < 0.00558	-1.67 q < 0.0089	-3.07 q < 0.00000000000179	-1.96 q < 0.000121
KIT	v-kit Hardy-Zuckerman 4 feline sarcoma viral oncogene homolog	Ssc.16096.2.S1_a_at	-1.48 q < 0	-1.55 q < 0.000664	-1.38 q < 0.0335	-1.32 q < 0.0689	-1.76 q < 0.00000182	-1.43 q < 0.0119
MANEA	mannosidase, endo-alpha	Ssc.8193.1.A1_at	-1.6 q < 0	-1.34 q < 0.255	-1.51 q < 0.0178	-1.83 q < 0.0000295	-1.65 q < 0.00238	-1.7 q < 0.000583
PSMC1	proteasome (prosome, macropain) 26S subunit, ATPase, 1	Ssc.1911.1.A1_at	-1.46 q < 0	-1.16 q < 0.927	-1.54 q < 0.00145	-1.49 q < 0.0034	-1.44 q < 0.0225	-1.7 q < 0.0000202
SNX9	sorting nexin 9	Ssc.15467.2.A1_at	1.52 q < 0	1.69 q < 0.00000295	1.31 q < 0.0824	1.65 q < 0.0000108	1.54 q < 0.000434	1.43 q < 0.00637
SNX9	sorting nexin 9	Ssc.15467.1.A1_at	1.39 q < 0.00000000197	1.71 q < 0.000208	1.39 q < 0.0834	1.36 q < 0.0931	1.29 q < 0.607	1.26 q < 0.541
SNX9	sorting nexin 9	Ssc.24257.1.S1_at	-1.07 q < 0.497	-1.48 q < 0.00832	1.02 q < 0.983	-1.01 q < 0.98	-1.02 q < 1	1.05 q < 1
TBX1	T-box 1	Ssc.13166.1.A1_at	-1.38 q < 0	-1.35 q < 0.0365	-1.39 q < 0.00928	-1.33 q < 0.0291	-1.54 q < 0.00018	-1.3 q < 0.0894
UPRT	uracil phosphoribosyltransferase (FUR1) homolog (S. cerevisiae)	Ssc.10699.1.A1_at	-1.6 q < 0	-1.19 q < 0.751	-2.18 q < 0.0000000000143	-1.72 q < 0.00000939	-1.6 q < 0.000434	-1.47 q < 0.0067
LSM3	LSM3 homolog, U6 small nuclear RNA associated (S. cerevisiae)	Ssc.1234.1.S1_at	-1.44 q < 0.000000000000878	-1.46 q < 0.0149	-1.48 q < 0.0104	-1.47 q < 0.00856	-1.19 q < 1	-1.64 q < 0.000184
ARMCX1	armadillo repeat containing, X-linked 1	Ssc.5616.1.S1_at	-1.41 q < 0.00000000000134	-1.47 q < 0.00521	-1.41 q < 0.0195	-1.41 q < 0.014	-1.37 q < 0.0756	-1.37 q < 0.0491
SLC46A3	solute carrier family 46, member 3	Ssc.6055.1.A1_at	1.45 q < 0.0000000000026	-1.04 q < 1	1.63 q < 0.000739	1.54 q < 0.00346	1.55 q < 0.00602	1.73 q < 0.0000614
SLC46A3	solute carrier family 46, member 3	Ssc.23758.1.S1_at	1.29 q < 0.0000000979	-1.04 q < 1	1.42 q < 0.0131	1.43 q < 0.00663	1.36 q < 0.0745	1.34 q < 0.062
SLC46A3	solute carrier family 46, member 3	Ssc.23758.2.A1_at	1.17 q < 0.00131	-1.08 q < 1	1.27 q < 0.177	1.33 q < 0.0389	1.16 q < 1	1.23 q < 0.41
SH3BGRL	SH3 domain binding glutamic acid-rich protein like	Ssc.7229.1.S1_at	-1.53 q < 0.00000000000268	-1.32 q < 0.381	-1.65 q < 0.00359	-1.48 q < 0.0348	-1.61 q < 0.0105	-1.6 q < 0.00808
SH3BGRL	SH3 domain binding glutamic acid-rich protein like	Ssc.7229.3.S1_at	-1.11 q < 0.00888	-1.13 q < 0.68	-1.14 q < 0.5	-1.09 q < 0.548	-1.09 q < 1	-1.1 q < 1
KIAA1370	KIAA1370	Ssc.14101.1.A1_at	-1.26 q < 0.00000000000314	-1.07 q < 1	-1.31 q < 0.00344	-1.27 q < 0.0142	-1.4 q < 0.000129	-1.27 q < 0.0179
KIAA1370	KIAA1370	Ssc.10620.1.A1_at	-1.13 q < 0.037	-1.2 q < 0.617	-1.07 q < 0.923	-1.1 q < 0.667	-1.22 q < 0.815	-1.08 q < 1
SERPINB1	serpin peptidase inhibitor, clade B (ovalbumin), member 1	Ssc.1303.1.S1_at	-1.46 q < 0.0000000000169	1.05 q < 1	-1.76 q < 0.000117	-1.58 q < 0.00324	-1.61 q < 0.00387	-1.56 q < 0.00637
TIAM1	T-cell lymphoma invasion and metastasis 1	Ssc.27360.1.A1_at	-1.17 q < 0.000022	-1.19 q < 0.302	-1.27 q < 0.0297	-1.4 q < 0.000218	-1.31 q < 0.0142	1.27 q < 0.0366
CNTF	ciliary neurotrophic factor	Ssc.10473.1.A1_at	1.67 q < 0	1.81 q < 0.000155	1.41 q < 0.118	2.21 q < 0.0000000443	1.45 q < 0.126	1.61 q < 0.00641
CSTB	cystatin B (stefin B)	Ssc.17323.1.S1_at	-1.76 q < 0	-1.52 q < 0.102	-1.77 q < 0.00379	-2.09 q < 0.0000207	-1.53 q < 0.126	-1.97 q < 0.000141
HUS1	HUS1 checkpoint homolog (S. pombe)	Ssc.21138.1.S1_at	-1.45 q < 0	-1.12 q < 1	-1.87 q < 0.00000000276	-1.12 q < 0.547	-1.53 q < 0.000343	-1.74 q < 0.000000152
HUS1	HUS1 checkpoint homolog (S. pombe)	Ssc.31013.1.A1_s_at	-1.31 q < 0	1.1 q < 1	-1.77 q < 0.00000000000429	-1.09 q < 0.552	-1.48 q < 0.0000143	-1.46 q < 0.0000204
IGHM	immunoglobulin heavy constant mu	Ssc.24103.1.A1_s_at	-2.82 q < 0	-1.75 q < 0.167	-3.4 q < 0.000000925	-1.85 q < 0.0545	-2.94 q < 0.0000447	-5.45 q < 0
IGHM	immunoglobulin heavy constant mu	Ssc.15945.1.A1_x_at	1.34 q < 0.0000000000207	1.42 q < 0.00542	1.3 q < 0.0834	1.23 q < 0.194	1.3 q < 0.146	1.47 q < 0.00136
IGHM	immunoglobulin heavy constant mu	Ssc.13778.1.S1_at	-2.24 q < 0	1.01 q < 1	-1.03 q < 0.977	1.24 q < 0.208	-1.09 q < 1	-63.01 q < 0
IGHM	immunoglobulin heavy constant mu	Ssc.11190.1.S1_at	-1.59 q < 0.00000000426	-1.02 q < 1	-1.15 q < 0.894	-1.22 q < 0.565	1.01 q < 1	-7.28 q < 0
IGHM	immunoglobulin heavy constant mu	Ssc.11070.1.S1_at	-1.26 q < 0.0000005	-1.02 q < 1	1.01 q < 0.991	1.02 q < 0.931	1.01 q < 1	-3.31 q < 0
IGHM	immunoglobulin heavy constant mu	Ssc.7706.1.A1_at	-1.1 q < 0.0933	-1.05 q < 1	1.06 q < 0.923	1.14 q < 0.494	-2.04 q < 0.00000000000306	1.07 q < 1
IL1RAPL1	interleukin 1 receptor accessory protein-like 1	Ssc.18626.1.A1_at	-1.41 q < 0	-1.14 q < 0.536	-1.2 q < 0.134	-1.5 q < 0.000000255	-1.65 q < 0.0000000000321	-1.65 q < 0.0000000000157
RABIF	RAB interacting factor	Ssc.22533.1.A1_at	-1.31 q < 0	-1.3 q < 0.0129	-1.47 q < 0.0000108	-1.23 q < 0.0715	-1.44 q < 0.0000605	-1.12 q < 0.93
TCF4	transcription factor 4	Ssc.1745.1.A1_at	-2.04 q < 0	-4 q < 0	-1.07 q < 0.954	-2.53 q < 0.00000355	-2.51 q < 0.00000691	-1.29 q < 0.958
TCF4	transcription factor 4	Ssc.28646.1.A1_at	-1.55 q < 0.0000000000145	-1.83 q < 0.000489	-1.71 q < 0.00344	-1.56 q < 0.024	-1.47 q < 0.174	-1.25 q < 0.862
TCF4	transcription factor 7-like 2 (T-cell specific, HMG-box)	Ssc.1745.1.A1_at	-2.04 q < 0	-4 q < 0	-1.07 q < 0.954	-2.53 q < 0.00000355	-2.51 q < 0.00000691	-1.29 q < 0.958
TCF4	transcription factor 7-like 2 (T-cell specific, HMG-box)	Ssc.28646.1.A1_at	-1.55 q < 0.0000000000145	-1.83 q < 0.000489	-1.71 q < 0.00344	-1.56 q < 0.024	-1.47 q < 0.174	-1.25 q < 0.862
CMTM3	CKLF-like MARVEL transmembrane domain containing 3	Ssc.25175.2.S1_at	-1.51 q < 0.000000000000191	-2.04 q < 0.0000000733	-1.49 q < 0.0178	-1.34 q < 0.118	-1.47 q < 0.0421	-1.29 q < 0.384

Gene Name	Gene Description	Probe Set ID	Breed MS-WC	Day 25 MS-WC	Day 45 MS-WC	Day 65 MS-WC	Day 85 MS-WC	Day 105 MS-WC
CMTM3	CKLF-like MARVEL transmembrane domain containing 3	Ssc.25175.3.S1_at	-1.42 q < 0.000000000865	-1.71 q < 0.000482	-1.56 q < 0.00744	-1.46 q < 0.0307	-1.23 q < 1	-1.22 q < 0.87
CMTM3	CKLF-like MARVEL transmembrane domain containing 3	Ssc.25175.1.S1_at	1.35 q < 0.000000269	1.74 q < 0.000187	1.05 q < 0.956	1.31 q < 0.173	1.45 q < 0.07	1.27 q < 0.555
COLEC11	collectin sub-family member 11	Ssc.4688.2.A1_a_at	-1.7 q < 0.000000000000191	-1.27 q < 0.808	-2.38 q < 0.000000953	-2.04 q < 0.000116	-1.66 q < 0.0408	-1.41 q < 0.358
LBP	lipopolysaccharide binding protein	Ssc.15980.1.S1_at	1.53 q < 0.000000000000191	-1.04 q < 1	1.15 q < 0.812	2.4 q < 0.000000000132	2.12 q < 0.000000106	1.48 q < 0.0343
TIMM22	translocase of inner mitochondrial membrane 22 homolog (yeast)	Ssc.1157.1.S1_at	-1.35 q < 0.000000000000191	-1.22 q < 0.323	-1.43 q < 0.00142	-1.28 q < 0.0598	-1.47 q < 0.00065	-1.38 q < 0.00637
KIAA0774	microtubule associated tumor suppressor candidate 2	Ssc.26369.1.S1_at	1.63 q < 0.000000000000369	1.45 q < 0.171	1.56 q < 0.0402	2.13 q < 0.00000631	1.71 q < 0.009	1.41 q < 0.252
KIAA0774	microtubule associated tumor suppressor candidate 2	Ssc.3936.1.S1_at	1.43 q < 0.00000000000268	1.16 q < 0.924	1.36 q < 0.0846	1.85 q < 0.000000925	1.63 q < 0.000352	1.25 q < 0.511
KIAA0774	microtubule associated tumor suppressor candidate 2	Ssc.26369.2.S1_at	1.24 q < 0.0002	1.14 q < 1	1.15 q < 0.796	1.55 q < 0.00335	1.3 q < 0.495	1.12 q < 1
C14orf147	chromosome 14 open reading frame 147	Ssc.5692.1.S1_at	1.62 q < 0.00000000000102	1.33 q < 0.511	1.74 q < 0.00393	2.05 q < 0.0000212	1.89 q < 0.000487	1.23 q < 0.994
RPS4X	ribosomal protein S4, X-linked	Ssc.14003.2.S1_at	-1.5 q < 0.00000000000102	-1.35 q < 0.217	-1.71 q < 0.00037	-1.35 q < 0.115	-1.5 q < 0.0361	-1.61 q < 0.00259
RPS4X	ribosomal protein S4, X-linked	Ssc.13017.1.A1_at	-1.15 q < 0.0181	-1.06 q < 1	-1.22 q < 0.442	-1.06 q < 0.796	-1.08 q < 1	-1.35 q < 0.0843
KIAA1755	KIAA1755	Ssc.23015.1.S1_at	-1.99 q < 0.00000000000165	1.01 q < 1	-3.04 q < 0.00000423	-2.27 q < 0.00197	-2.72 q < 0.000105	-1.67 q < 0.218
C12orf45	chromosome 12 open reading frame 45	Ssc.21490.1.S1_a_at	-1.28 q < 0.00000000000176	-1.12 q < 0.867	-1.23 q < 0.0769	-1.34 q < 0.00203	-1.39 q < 0.000409	-1.32 q < 0.0051
C12orf45	chromosome 12 open reading frame 45	Ssc.21490.1.S1_at	-1.11 q < 0.0122	1.02 q < 1	-1.08 q < 0.853	-1.2 q < 0.12	-1.15 q < 0.921	-1.14 q < 0.695
DDX17	DEAD (Asp-Glu-Ala-Asp) box polypeptide 17	Ssc.8674.1.A1_at	-3.12 q < 0.00000000000176	-2.59 q < 0.114	-2.66 q < 0.0762	-3.05 q < 0.0197	-4.25 q < 0.00141	-3.29 q < 0.014
DDX17	DEAD (Asp-Glu-Ala-Asp) box polypeptide 17	Ssc.30341.1.S1_at	1.24 q < 0.0000488	1.39 q < 0.0597	1.18 q < 0.616	1.12 q < 0.631	1.25 q < 0.731	1.29 q < 0.255
AASDHPPT	aminoadipate-semialdehyde dehydrogenase-phosphopantetheinyl transferase	Ssc.7455.1.A1_at	1.47 q < 0.00000000000221	1.5 q < 0.0148	1.36 q < 0.122	1.64 q < 0.000671	1.55 q < 0.009	1.32 q < 0.272
AASDHPPT	aminoadipate-semialdehyde dehydrogenase-phosphopantetheinyl transferase	Ssc.23863.1.S1_at	-1.24 q < 0.00251	-1.32 q < 0.459	-1.27 q < 0.515	-1.23 q < 0.428	-1.21 q < 1	-1.18 q < 1
RALGPS1	Ral GEF with PH domain and SH3 binding motif 1	Ssc.25517.1.S1_at	-1.82 q < 0.0000000000026	1.03 q < 1	-2.44 q < 0.0000477	-2.52 q < 0.0000161	-1.78 q < 0.0568	-1.87 q < 0.0148
TM9SF2	transmembrane 9 superfamily member 2	Ssc.26464.1.A1_at	1.47 q < 0.00000000000304	1.21 q < 0.803	1.28 q < 0.325	1.66 q < 0.000509	1.7 q < 0.000343	1.56 q < 0.00479
SLC12A8	solute carrier family 12 (potassium/chloride transporters), member 8	Ssc.10023.1.A1_at	1.36 q < 0.00000000000473	1.39 q < 0.0149	1.48 q < 0.00145	1.12 q < 0.547	1.1 q < 1	1.82 q < 0.00000000984
DLG1	discs, large homolog 1 (Drosophila)	Ssc.10952.1.S1_at	1.46 q < 0.00000000000991	1.07 q < 1	1.34 q < 0.172	1.93 q < 0.00000134	1.62 q < 0.00213	1.46 q < 0.0301
DLG1	discs, large homolog 1 (Drosophila)	Ssc.30931.1.A1_at	-1.17 q < 0.0154	-1.18 q < 0.925	-1.19 q < 0.654	-1.11 q < 0.676	-1.15 q < 1	-1.24 q < 0.666
DLG1	discs, large (Drosophila) homolog-associated protein 5	Ssc.10952.1.S1_at	1.46 q < 0.00000000000991	1.07 q < 1	1.34 q < 0.172	1.93 q < 0.00000134	1.62 q < 0.00213	1.46 q < 0.0301
DLG1	discs, large (Drosophila) homolog-associated protein 5	Ssc.30931.1.A1_at	-1.17 q < 0.0154	-1.18 q < 0.925	-1.19 q < 0.654	-1.11 q < 0.676	-1.15 q < 1	-1.24 q < 0.666
PKIB	protein kinase (cAMP-dependent, catalytic) inhibitor beta	Ssc.11036.1.S1_at	-1.54 q < 0.0000000000137	-1.6 q < 0.0179	-1.45 q < 0.103	-1.91 q < 0.0000771	-1.28 q < 1	-1.54 q < 0.0369
MAP4K4	mitogen-activated protein kinase kinase kinase kinase 4	Ssc.21192.3.S1_at	-1.43 q < 0.0000000000163	-1.27 q < 0.385	-1.57 q < 0.00218	-1.46 q < 0.0145	-1.33 q < 0.311	-1.52 q < 0.00626
BEX5	brain expressed, X-linked 5	Ssc.2607.1.S1_at	1.33 q < 0.0000000000199	1.02 q < 1	1.5 q < 0.000392	1.61 q < 0.00000789	1.41 q < 0.00753	1.21 q < 0.483
SF1	splicing factor 1	Ssc.30179.1.A1_at	-1.47 q < 0.0000000000201	-1.72 q < 0.000457	-1.39 q < 0.109	1 q < 0.991	-1.77 q < 0.000199	-1.63 q < 0.00232
ACOX3	acyl-CoA oxidase 3, pristanoyl	Ssc.7981.1.A1_at	1.48 q < 0.0000000000232	1.59 q < 0.00656	1.56 q < 0.0106	1.5 q < 0.0209	1.34 q < 0.454	1.42 q < 0.0911
RCN1	reticulocalbin 1, EF-hand calcium binding domain	Ssc.7715.1.A1_at	-1.78 q < 0.0000000000341	-1.53 q < 0.314	-2.06 q < 0.00344	-1.71 q < 0.0487	-1.68 q < 0.161	-1.98 q < 0.00695
NP_057359	-	Ssc.8455.1.A1_at	-1.43 q < 0.0000000000366	-1.46 q < 0.0285	-1.54 q < 0.00558	-1.41 q < 0.0415	-1.44 q < 0.0556	-1.31 q < 0.288
NP_057359	-	Ssc.6767.1.S1_at	1.13 q < 0.202	-1.03 q < 1	-1.25 q < 0.579	-1.04 q < 0.917	1.52 q < 0.094	1.65 q < 0.00926

Gene Name	Gene Description	Probe Set ID	Breed MS-WC	Day 25 MS-WC	Day 45 MS-WC	Day 65 MS-WC	Day 85 MS-WC	Day 105 MS-WC
PCCA	propionyl CoA carboxylase, alpha polypeptide	Ssc.10223.1.A1_at	-1.42 q < 0.0000000000527	-1.08 q < 1	-1.8 q < 0.00000978	-1.5 q < 0.00692	-1.6 q < 0.00177	-1.22 q < 0.732
PCNT	pericentrin	Ssc.6277.1.A1_at	-1.29 q < 0.0000000000717	-1.23 q < 0.217	-1.49 q < 0.0000612	-1.35 q < 0.00514	-1.31 q < 0.0376	-1.08 q < 1
TGFBI	transforming growth factor, beta-induced, 68kDa	Ssc.16671.1.S1_at	-1.65 q < 0.0000000000905	-1.86 q < 0.00541	-1.61 q < 0.07	-1.66 q < 0.0325	-1.4 q < 0.747	-1.76 q < 0.0154
PKD2	polycystic kidney disease 2 (autosomal dominant)	Ssc.840.1.A1_at	1.39 q < 0.00000000011	1.19 q < 0.789	1.52 q < 0.00385	1.7 q < 0.0000438	1.06 q < 1	1.6 q < 0.000666
PKD2	protein kinase D2	Ssc.840.1.A1_at	1.39 q < 0.00000000011	1.19 q < 0.789	1.52 q < 0.00385	1.7 q < 0.0000438	1.06 q < 1	1.6 q < 0.000666
CHI3L1	chitinase 3-like 1 (cartilage glycoprotein-39)	Ssc.90.1.S1_at	2.7 q < 0.000000000141	1.11 q < 1	4.71 q < 0.000112	4.79 q < 0.0000726	3.23 q < 0.016	1.76 q < 0.768
MYO5A	myosin VA (heavy chain 12, myoxin)	Ssc.6843.1.A1_at	1.37 q < 0.000000000147	1.13 q < 1	1.56 q < 0.000939	1.74 q < 0.00000609	1.48 q < 0.00838	1.07 q < 1
MYO5A	myosin VA (heavy chain 12, myoxin)	Ssc.1957.1.A1_at	1.19 q < 0.00353	-1.02 q < 1	1.32 q < 0.163	1.47 q < 0.0103	1.31 q < 0.389	-1.05 q < 1
BAZ2B	bromodomain adjacent to zinc finger domain, 2B	Ssc.28060.1.A1_at	1.33 q < 0.000000000483	1.33 q < 0.0811	1.36 q < 0.0339	-1.02 q < 0.933	1.49 q < 0.00227	1.58 q < 0.0000798
PTP4A2	protein tyrosine phosphatase type IVA, member 2	Ssc.11158.1.A1_at	-1.36 q < 0.000000000639	-1.52 q < 0.00255	-1.45 q < 0.0124	-1.38 q < 0.0324	-1.25 q < 0.686	-1.22 q < 0.603
ESD	esterase D	Ssc.217.1.S1_at	-1.38 q < 0.000000000824	-1.04 q < 1	-1.49 q < 0.011	-1.25 q < 0.251	-1.52 q < 0.00909	-1.73 q < 0.0000377
STXBP1	syntaxin binding protein 1	Ssc.6797.1.S1_at	1.5 q < 0.000000000997	1.33 q < 0.443	1.89 q < 0.000212	1.57 q < 0.0217	1.78 q < 0.00212	1.06 q < 1
ATP9A	ATPase, class II, type 9A	Ssc.3410.2.S1_a_at	-1.36 q < 0.000000000999	-1.16 q < 0.913	-1.4 q < 0.0337	-1.43 q < 0.0145	-1.51 q < 0.00528	-1.31 q < 0.167
ATP9A	ATPase, class II, type 9A	Ssc.3410.1.S1_a_at	-1.15 q < 0.0119	-1.05 q < 1	-1.15 q < 0.67	-1.19 q < 0.349	-1.25 q < 0.519	-1.11 q < 1
C20orf27	chromosome 20 open reading frame 27	Ssc.3560.3.A1_at	-1.28 q < 0.00000000118	-1.27 q < 0.124	-1.32 q < 0.032	-1.36 q < 0.00896	-1.35 q < 0.026	-1.14 q < 0.957
CLOCK	clock homolog (mouse)	Ssc.24285.1.S1_at	-1.29 q < 0.00000000264	-1.21 q < 0.399	-1.39 q < 0.0067	-1.32 q < 0.0325	-1.35 q < 0.0309	-1.17 q < 0.716
CLOCK	clock homolog (mouse)	Ssc.4897.1.A1_at	-1.85 q < 0.0000377	-2.05 q < 0.261	-2.16 q < 0.147	-1.01 q < 0.988	-2.4 q < 0.123	-2.03 q < 0.293
TCP1	t-complex 1	Ssc.4426.1.S1_at	1.58 q < 0.00000000431	1.82 q < 0.00746	1.11 q < 0.922	1.9 q < 0.00227	1.52 q < 0.302	1.66 q < 0.0449
TCP1	t-complex 1	Ssc.4426.2.S1_at	1.42 q < 0.000131	1.75 q < 0.0572	1.01 q < 0.997	1.73 q < 0.0382	1.51 q < 0.547	1.26 q < 1
MGST1	microsomal glutathione S-transferase 1	Ssc.1013.1.A1_at	-2.12 q < 0.00000000464	-1.14 q < 1	-2.33 q < 0.0389	-4.31 q < 0.00000398	-2.42 q < 0.0459	-1.57 q < 0.838
A2ML1	alpha-2-macroglobulin-like 1	Ssc.9422.1.A1_at	1.22 q < 0.00000000499	1 q < 1	1.36 q < 0.000749	1.3 q < 0.00453	1.16 q < 0.72	1.3 q < 0.00674
MAP3K7	mitogen-activated protein kinase kinase kinase 7	Ssc.10311.2.A1_at	1.24 q < 0.00000000683	1.16 q < 0.595	1.18 q < 0.311	1.27 q < 0.0349	1.32 q < 0.0142	1.27 q < 0.0451
MAP3K7	mitogen-activated protein kinase kinase kinase 7	Ssc.24116.1.A1_at	1.11 q < 0.0216	1.2 q < 0.34	1.02 q < 0.986	1.2 q < 0.181	1.04 q < 1	1.12 q < 1
EDNRB	endothelin receptor type B	Ssc.3295.2.A1_at	-2.49 q < 0.00000000882	-3.01 q < 0.0275	-4.27 q < 0.000628	-2.69 q < 0.0424	-1.54 q < 1	-1.79 q < 0.752
EDNRB	endothelin receptor type B	Ssc.3295.1.S1_at	-1.29 q < 0.0466	-2.02 q < 0.0285	-1.35 q < 0.711	-1.43 q < 0.38	-1.23 q < 1	1.33 q < 1
FRYL	FRY-like	Ssc.14247.2.A1_at	1.48 q < 0.0000000194	1.75 q < 0.00387	-1.02 q < 0.989	1.59 q < 0.0242	2.15 q < 0.0000126	1.2 q < 1
EIF3D	eukaryotic translation initiation factor 3, subunit D	Ssc.6735.1.A1_at	-1.21 q < 0.0000000376	1.11 q < 0.897	-1.51 q < 0.00000181	-1.33 q < 0.00284	-1.13 q < 1	-1.29 q < 0.0154
EIF3D	eukaryotic translation initiation factor 3, subunit D	Ssc.11296.1.A1_at	-1.26 q < 0.000000000641	-1.21 q < 0.228	-1.3 q < 0.0232	-1.16 q < 0.303	-1.38 q < 0.00229	-1.25 q < 0.0817
Q9H6J2	-	Ssc.4917.2.S1_a_at	1.29 q < 0.0000000742	1.1 q < 1	1.47 q < 0.00395	1.45 q < 0.00411	1.4 q < 0.0353	1.1 q < 1
Q9H6J2	-	Ssc.4917.1.S1_at	1.13 q < 0.012	-1.04 q < 1	1.14 q < 0.647	1.4 q < 0.0022	1.14 q < 1	1.04 q < 1
KCTD3	potassium channel tetramerisation domain containing 3	Ssc.21138.3.S1_a_at	-1.22 q < 0.0000000983	1.05 q < 1	-1.45 q < 0.0000536	-1.02 q < 0.918	-1.39 q < 0.00116	-1.35 q < 0.00288
FAM73B	family with sequence similarity 73, member B	Ssc.19153.2.A1_at	-1.23 q < 0.000000197	-1.11 q < 0.979	-1.33 q < 0.0115	-1.31 q < 0.0152	-1.37 q < 0.00636	-1.04 q < 1
Q8N682	-	Ssc.9726.1.A1_at	-1.51 q < 0.000000294	-1.09 q < 1	-1.21 q < 0.823	-1.68 q < 0.0303	-1.99 q < 0.00212	-1.77 q < 0.0165
PLA2G10	phospholipase A2, group X	Ssc.1762.1.S1_at	1.19 q < 0.00000457	-1.02 q < 1	1 q < 0.997	1.28 q < 0.0298	1.31 q < 0.0216	1.46 q < 0.0000443
PLA2G10	phospholipase A2, group X	Ssc.22078.1.A1_at	1.48 q < 0.000565	1.16 q < 1	1.1 q < 0.95	1.62 q < 0.195	2.08 q < 0.04	1.63 q < 0.37
PLA2G10	phospholipase A2, group X	Ssc.1762.2.A1_at	1.11 q < 0.0293	1.01 q < 1	1.13 q < 0.658	1.21 q < 0.156	1.11 q < 1	1.09 q < 1
GMDS	GDP-mannose 4,6-dehydratase	Ssc.28503.1.S1_at	-1.29 q < 0.00000583	1.24 q < 0.567	-1.44 q < 0.0367	-1.67 q < 0.000304	-1.46 q < 0.0425	-1.27 q < 0.456

Gene Name	Gene Description	Probe Set ID	Breed MS-WC	Day 25 MS-WC	Day 45 MS-WC	Day 65 MS-WC	Day 85 MS-WC	Day 105 MS-WC
GMDS	GDP-mannose 4,6-dehydratase	Ssc.8896.1.A1_at	-1.13 q < 0.0188	-1.19 q < 0.563	-1.07 q < 0.917	-1.12 q < 0.55	-1.16 q < 1	-1.12 q < 1
GMDS	GDP-mannose 4,6-dehydratase	Ssc.13360.1.A1_at	-1.17 q < 0.0511	-1.92 q < 0.0000393	-1.06 q < 0.954	-1.06 q < 0.857	1.05 q < 1	-1.06 q < 1
POP1	processing of precursor 1, ribonuclease P/MRP subunit (S. cerevisiae)	Ssc.23194.1.A1_at	-1.17 q < 0.0000418	-1.4 q < 0.000343	-1.33 q < 0.00414	-1.25 q < 0.0473	-1.05 q < 1	1.14 q < 0.768
POP1	blood vessel epicardial substance	Ssc.23194.1.A1_at	-1.17 q < 0.0000418	-1.4 q < 0.000343	-1.33 q < 0.00414	-1.25 q < 0.0473	-1.05 q < 1	1.14 q < 0.768
POP1	PYD (pyrin domain) containing 1	Ssc.23194.1.A1_at	-1.17 q < 0.0000418	-1.4 q < 0.000343	-1.33 q < 0.00414	-1.25 q < 0.0473	-1.05 q < 1	1.14 q < 0.768
CASP2	caspase 2, apoptosis-related cysteine peptidase	Ssc.9673.1.A1_at	-1.48 q < 0.000000000000726	-1.44 q < 0.0519	-1.65 q < 0.000905	-1.35 q < 0.0989	-1.42 q < 0.095	-1.58 q < 0.00318
CAPZB	capping protein (actin filament) muscle Z-line, beta	Ssc.10297.3.S1_a_at	-1.41 q < 0.000000000000878	-1.55 q < 0.000735	-1.61 q < 0.000183	-1.32 q < 0.0858	-1.32 q < 0.202	-1.3 q < 0.199
CAPZB	capping protein (actin filament) muscle Z-line, beta	Ssc.10297.1.S1_at	-1.42 q < 0.00000031	-1.52 q < 0.0746	-1.65 q < 0.0132	-1.1 q < 0.759	-1.33 q < 0.828	-1.58 q < 0.0335
ADA	adenosine deaminase	Ssc.11171.1.S1_at	-1.58 q < 0.00000000000338	1.04 q < 1	-1.47 q < 0.0932	-2.54 q < 0.00000000243	-1.29 q < 1	-2.13 q < 0.0000029
GATA3	GATA binding protein 3	Ssc.21354.2.S1_at	-1.61 q < 0.00000000000632	-1.18 q < 1	-1.22 q < 0.715	-2.16 q < 0.00000792	-2.34 q < 0.000000776	-1.49 q < 0.128
GATA3	GATA binding protein 3	Ssc.21354.1.S1_at	-1.24 q < 0.00854	-1.15 q < 1	-1.33 q < 0.434	-1.32 q < 0.305	-1.25 q < 1	-1.16 q < 1
Q9UI52	-	Ssc.14086.1.A1_at	1.51 q < 0.00000000000949	-1.24 q < 0.772	2.04 q < 0.00000157	2.36 q < 0.00000000192	1.41 q < 0.222	1.43 q < 0.102
RPS19BP1	ribosomal protein S19 binding protein 1	Ssc.3542.1.S1_at	-1.3 q < 0.0000000000103	-1.14 q < 0.773	-1.44 q < 0.000419	-1.24 q < 0.098	-1.3 q < 0.0593	-1.41 q < 0.00118
PCOLCE	procollagen C-endopeptidase enhancer	Ssc.1122.1.S1_at	-1.59 q < 0.0000000000164	-1.49 q < 0.124	-2.07 q < 0.0000389	-1.34 q < 0.255	-1.53 q < 0.121	-1.6 q < 0.0317
FKBP14	FK506 binding protein 14, 22 kDa	Ssc.6848.1.A1_at	-1.53 q < 0.0000000000181	-1.12 q < 1	-2.29 q < 0.0000000482	-1.31 q < 0.254	-1.51 q < 0.0809	-1.65 q < 0.00637
FKBP14	FK506 binding protein 14, 22 kDa	Ssc.20986.1.S1_at	-1.3 q < 0.00000885	-1.03 q < 1	-1.69 q < 0.00044	-1.35 q < 0.112	-1.07 q < 1	-1.45 q < 0.0392
ARPC1B	actin related protein 2/3 complex, subunit 1B, 41kDa	Ssc.15456.1.S1_at	-1.45 q < 0.0000000000388	-1.64 q < 0.00154	-1.2 q < 0.656	-2.33 q < 0.000000000288	-1.1 q < 1	-1.29 q < 0.44
LIMS1	LIM and senescent cell antigen-like domains 1	Ssc.24149.1.A1_at	-1.44 q < 0.0000000000826	-1.23 q < 0.672	-1.75 q < 0.000139	-1.4 q < 0.057	-1.42 q < 0.117	-1.44 q < 0.0449
ANPEP	alanyl (membrane) aminopeptidase	Ssc.820.1.S1_at	1.74 q < 0.000000000145	1.25 q < 1	1.44 q < 0.402	2.99 q < 0.000000178	1.83 q < 0.0421	1.64 q < 0.131
YWHAZ	tyrosine 3-monooxygenase/tryptophan 5-monooxygenase activation protein, zeta polypeptide	Ssc.17423.1.S1_at	-1.8 q < 0.000000000159	-1.55 q < 0.338	-1.57 q < 0.229	-2.22 q < 0.00124	-2.03 q < 0.0115	-1.7 q < 0.116
YWHAZ	tyrosine 3-monooxygenase/tryptophan 5-monooxygenase activation protein, zeta polypeptide	Ssc.24859.1.A1_at	1.32 q < 0.00000000169	1.25 q < 0.314	1.24 q < 0.276	1.71 q < 0.00000169	1.26 q < 0.423	1.19 q < 0.682
YWHAZ	tyrosine 3-monooxygenase/tryptophan 5-monooxygenase activation protein, zeta polypeptide	Ssc.9681.1.A1_at	1.11 q < 0.0883	1.08 q < 1	1.03 q < 0.973	1.38 q < 0.015	1.03 q < 1	1.06 q < 1
TPT1	tumor protein, translationally-controlled 1	Ssc.14343.1.S1_at	-1.28 q < 0.000000000231	-1.23 q < 0.2	-1.42 q < 0.000848	-1.19 q < 0.225	-1.24 q < 0.239	-1.32 q < 0.0162
SLC25A36	solute carrier family 25, member 36	Ssc.27130.1.A1_at	-1.3 q < 0.000000000247	-1.62 q < 0.00000287	-1.25 q < 0.155	-1.18 q < 0.29	-1.39 q < 0.0103	-1.13 q < 1
CD55	CD55 molecule, decay accelerating factor for complement (Cromer blood group)	Ssc.31082.1.A1_at	1.77 q < 0.000000000375	1.16 q < 1	2.36 q < 0.00037	2.93 q < 0.00000133	1.55 q < 0.535	1.39 q < 0.788
CD55	CD55 molecule, decay accelerating factor for complement (Cromer blood group)	Ssc.271.1.A1_at	1.71 q < 0.00000038	1.23 q < 1	2.22 q < 0.00924	2.77 q < 0.000144	1.38 q < 1	1.43 q < 0.867
CD55	CD55 molecule, decay accelerating factor for complement (Cromer blood group)	Ssc.7698.1.S1_at	1.29 q < 0.0000881	1.06 q < 1	1.48 q < 0.0554	1.71 q < 0.00127	1.18 q < 1	1.15 q < 1
CD55	CD55 molecule, decay accelerating factor for complement (Cromer blood group)	Ssc.7698.2.A1_at	1.24 q < 0.0000983	1.02 q < 1	1.34 q < 0.113	1.65 q < 0.00021	1.11 q < 1	1.16 q < 1
CD55	CD55 molecule, decay accelerating factor for complement (Cromer blood group)	Ssc.272.1.S1_a_at	1.87 q < 0.000298	1.12 q < 1	2.43 q < 0.134	3.3 q < 0.00949	1.61 q < 1	1.58 q < 1
NFIB	nuclear factor I/B	Ssc.724.1.S1_at	-2.04 q < 0.000000000784	-1.11 q < 1	-1.79 q < 0.206	-3.68 q < 0.0000056	-1.99 q < 0.163	-2.42 q < 0.00912
NFIB	nuclear factor I/B	Ssc.19423.1.A1_at	1.1 q < 0.122	-1.01 q < 1	-1.01 q < 0.99	1.23 q < 0.22	1.01 q < 1	1.36 q < 0.0394

Gene Name	Gene Description	Probe Set ID	Breed MS-WC	Day 25 MS-WC	Day 45 MS-WC	Day 65 MS-WC	Day 85 MS-WC	Day 105 MS-WC
LOC777593	-	Ssc.26112.1.S1_at	-1.69 q < 0.00000000109	1 q < 1	-1.58 q < 0.155	-2.61 q < 0.00000579	-1.75 q < 0.0756	-1.9 q < 0.0116
PITRM1	pitrilysin metallopeptidase 1	Ssc.3063.1.S1_at	-1.36 q < 0.00000000137	-1.11 q < 1	-1.57 q < 0.000937	-1.35 q < 0.0577	-1.42 q < 0.045	-1.37 q < 0.062
PITRM1	pitrilysin metallopeptidase 1	Ssc.3063.2.S1_at	-1.21 q < 0.00041	-1.13 q < 1	-1.42 q < 0.017	-1.07 q < 0.759	-1.24 q < 0.709	-1.19 q < 0.815
ZMAT3	zinc finger, matrin type 3	Ssc.25122.1.S1_at	-1.48 q < 0.00000000142	-1.88 q < 0.000159	-1.33 q < 0.34	-1.47 q < 0.0578	-1.2 q < 1	-1.59 q < 0.0183
FRY	furry homolog (Drosophila)	Ssc.6930.2.A1_at	1.47 q < 0.00000000144	1.77 q < 0.000836	1.22 q < 0.663	1.23 q < 0.421	1.25 q < 1	2.05 q < 0.00000398
FRY	furry homolog (Drosophila)	Ssc.6930.1.A1_at	-1.16 q < 0.0498	1.02 q < 1	-1.28 q < 0.403	-1.14 q < 0.608	-1.43 q < 0.126	-1.02 q < 1
CA7	carbonic anhydrase VII	Ssc.30512.1.A1_at	1.64 q < 0.00000000159	-1 q < 1	1.59 q < 0.115	2.83 q < 0.00000016	1.36 q < 1	1.96 q < 0.00355
UGCGL2	UDP-glucose glycoprotein glucosyltransferase 2	Ssc.3654.1.A1_at	1.51 q < 0.00000000169	1.43 q < 0.222	1.3 q < 0.488	1.34 q < 0.251	1.74 q < 0.00747	1.83 q < 0.00115
C5orf23	chromosome 5 open reading frame 23	Ssc.7890.1.S1_at	-1.68 q < 0.00000000222	-2.19 q < 0.000698	-1.55 q < 0.193	-1.86 q < 0.0133	-1.68 q < 0.149	-1.25 q < 1
C5orf23	chromosome 5 open reading frame 23	Ssc.26802.1.S1_at	-1.49 q < 0.000105	-2.05 q < 0.0149	-1.48 q < 0.437	-1.63 q < 0.144	-1.32 q < 1	-1.13 q < 1
C5orf23	chromosome 5 open reading frame 23	Ssc.15291.1.A1_at	-1.44 q < 0.000186	-2.12 q < 0.00344	-1.46 q < 0.425	-1.42 q < 0.333	-1.43 q < 1	1 q < 1
C5orf23	chromosome 5 open reading frame 23	Ssc.24172.1.A1_at	-1.25 q < 0.000964	-1.58 q < 0.0134	-1.23 q < 0.62	-1.08 q < 0.808	-1.31 q < 0.73	-1.11 q < 1
EFHA1	EF-hand domain family, member A1	Ssc.13850.2.S1_at	-1.3 q < 0.00000000249	-1.05 q < 1	-1.47 q < 0.00145	-1.22 q < 0.214	-1.26 q < 0.346	-1.57 q < 0.000058
SNAPC3	small nuclear RNA activating complex, polypeptide 3, 50kDa	Ssc.26370.1.S1_at	-1.32 q < 0.00000000259	-1.27 q < 0.269	-1.08 q < 0.91	-1.26 q < 0.165	-1.54 q < 0.000749	-1.54 q < 0.000556
CASP8AP2	caspase 8 associated protein 2	Ssc.24845.1.S1_at	1.21 q < 0.0000000056	1.24 q < 0.0408	1.23 q < 0.0398	1.2 q < 0.0796	1.14 q < 0.899	1.22 q < 0.0678
CASP8AP2	caspase 8 associated protein 2	Ssc.30354.1.A1_at	1.17 q < 0.00111	1.3 q < 0.105	1.15 q < 0.658	1.1 q < 0.634	1.13 q < 1	1.19 q < 0.673
CCNB1IP1	cyclin B1 interacting protein 1	Ssc.19486.1.S1_at	-1.25 q < 0.00000000563	-1.14 q < 0.775	-1.23 q < 0.132	-1.23 q < 0.109	-1.3 q < 0.0457	-1.35 q < 0.00563
COX4I1	cytochrome c oxidase subunit IV isoform 1	Ssc.12219.1.A1_at	-1.32 q < 0.00000000683	-1.17 q < 0.82	-1.51 q < 0.00151	-1.29 q < 0.111	-1.25 q < 0.583	-1.4 q < 0.0215
TCA	-	Ssc.15848.1.S1_at	1.21 q < 0.00000000818	1.1 q < 0.987	1.34 q < 0.00114	1.18 q < 0.157	1.21 q < 0.224	1.25 q < 0.0322
TCA	-	Ssc.428.5.S1_at	-1.07 q < 0.568	-1.03 q < 1	1.08 q < 0.913	-1 q < 0.991	-1.02 q < 1	-1.44 q < 0.0273
TCA	-	Ssc.29007.1.A1_at	1.05 q < 0.633	-1.1 q < 1	1.03 q < 0.972	1.38 q < 0.00577	-1.04 q < 1	1.02 q < 1
CCDC90B	coiled-coil domain containing 90B	Ssc.12887.1.A1_at	1.24 q < 0.000000011	1.05 q < 1	1.17 q < 0.423	1.5 q < 0.0000176	1.3 q < 0.0353	1.23 q < 0.171
C1orf55	chromosome 1 open reading frame 55	Ssc.11105.1.S1_at	-1.22 q < 0.0000000116	-1.05 q < 1	-1.43 q < 0.0000735	-1.15 q < 0.281	-1.25 q < 0.0993	-1.26 q < 0.0367
C1orf55	chromosome 1 open reading frame 55	Ssc.11105.2.S1_at	-1.14 q < 0.0045	-1.02 q < 1	-1.28 q < 0.0703	-1.07 q < 0.706	-1.16 q < 1	-1.18 q < 0.6
RAD50	RAD50 homolog (S. cerevisiae)	Ssc.14035.1.S1_at	-1.28 q < 0.0000000165	-1.34 q < 0.0416	-1.44 q < 0.00239	-1.06 q < 0.782	-1.29 q < 0.185	-1.3 q < 0.0843
TMEM130	transmembrane protein 130	Ssc.27421.1.A1_at	1.64 q < 0.0000000212	1.01 q < 1	2.11 q < 0.00231	1.57 q < 0.131	1.05 q < 1	3.42 q < 0.00000000222
ALCAM	activated leukocyte cell adhesion molecule	Ssc.10323.1.A1_at	-1.32 q < 0.000000023	-1.19 q < 0.733	-1.42 q < 0.0227	-1.29 q < 0.128	-1.5 q < 0.00643	-1.24 q < 0.493
MCF2L2	MCF.2 cell line derived transforming sequence-like 2	Ssc.30296.2.A1_at	1.38 q < 0.0000000233	1.17 q < 1	1.29 q < 0.332	1.78 q < 0.0000664	1.28 q < 0.747	1.45 q < 0.0497
MCF2L2	MCF.2 cell line derived transforming sequence-like 2	Ssc.30296.1.S1_at	1.18 q < 0.00127	1.11 q < 1	1.14 q < 0.717	1.27 q < 0.118	1.22 q < 0.747	1.15 q < 1
HMGCS1	3-hydroxy-3-methylglutaryl-CoA synthase 1 (soluble)	Ssc.13604.1.A1_at	1.43 q < 0.0000000247	1.15 q < 1	1.23 q < 0.651	1.71 q < 0.00202	1.5 q < 0.0992	1.67 q < 0.00489
HMGCS1	3-hydroxy-3-methylglutaryl-CoA synthase 1 (soluble)	Ssc.11126.1.A1_at	1.45 q < 0.0000364	1.71 q < 0.0691	1 q < 1	1.8 q < 0.0209	1.46 q < 0.712	1.41 q < 0.608
ACTB	actin, beta	Ssc.4003.1.S1_at	1.21 q < 0.0000000266	1.19 q < 0.242	1.16 q < 0.317	1 q < 0.989	1.33 q < 0.00343	1.39 q < 0.000133
ACTB	actin, beta	Ssc.4003.2.A1_at	-1.23 q < 0.0000448	-1 q < 1	-1.31 q < 0.117	-1.08 q < 0.743	-1.36 q < 0.0982	-1.46 q < 0.00659
SPOCK3	sparc/osteonectin, cwcv and kazal-like domains proteoglycan (testican) 3	Ssc.6472.1.S1_at	1.29 q < 0.0000000562	1.05 q < 1	1.21 q < 0.404	1.1 q < 0.65	1.73 q < 0.00000231	1.48 q < 0.00271
SPOCK3	sparc/osteonectin, cwcv and kazal-like domains proteoglycan (testican) 3	Ssc.25051.1.S1_at	1.21 q < 0.000136	-1.02 q < 1	1.23 q < 0.354	1.05 q < 0.825	1.48 q < 0.00528	1.4 q < 0.0218
SNX29	sorting nexin 29	Ssc.5001.2.A1_at	-1.3 q < 0.0000000637	-1.19 q < 0.683	-1.41 q < 0.0188	-1.21 q < 0.291	-1.5 q < 0.00392	-1.22 q < 0.591
HOXA10	homeobox A10	Ssc.15962.1.S1_at	-1.36 q < 0.0000000732	-1.28 q < 0.453	-1.53 q < 0.00924	-1.43 q < 0.0378	-1.2 q < 1	-1.37 q < 0.144
HOXA10	homeobox A10	Ssc.26254.1.S1_at	-1.3 q < 0.0000135	-1.22 q < 0.796	-1.36 q < 0.181	-1.53 q < 0.0123	-1.2 q < 1	-1.25 q < 0.703
HOXA10	homeobox A10	Ssc.27606.1.S1_at	-1.22 q < 0.00138	-1.23 q < 0.684	-1.24 q < 0.501	-1.43 q < 0.045	-1.07 q < 1	-1.17 q < 1
HOXA10	homeobox A10	Ssc.12652.1.A1_at	-1.24 q < 0.0245	1.02 q < 1	-1.41 q < 0.356	-1.34 q < 0.346	-1.32 q < 1	-1.2 q < 1

Gene Name	Gene Description	Probe Set ID	Breed MS-WC	Day 25 MS-WC	Day 45 MS-WC	Day 65 MS-WC	Day 85 MS-WC	Day 105 MS-WC
UGT8	UDP glycosyltransferase 8	Ssc.17490.1.S1_at	1.65 q < 0.0000000735	1.66 q < 0.168	2.41 q < 0.000325	2.35 q < 0.000435	1.24 q < 1	1.07 q < 1
CLEC10A	C-type lectin domain family 10, member A	Ssc.1590.1.S1_at	-1.46 q < 0.0000000808	-1.16 q < 1	-1.65 q < 0.0168	-1.53 q < 0.0456	-1.45 q < 0.318	-1.54 q < 0.0693
CHURC1	churchill domain containing 1	Ssc.10209.1.A1_at	1.33 q < 0.0000000837	-1.05 q < 1	1.33 q < 0.147	1.68 q < 0.000159	1.35 q < 0.239	1.48 q < 0.0153
FOXJ2	forkhead box J2	Ssc.2463.2.A1_at	-1.21 q < 0.0000000955	-1.23 q < 0.119	-1.32 q < 0.00591	-1.1 q < 0.515	-1.29 q < 0.0309	-1.13 q < 0.895
LIMA1	LIM domain and actin binding 1	Ssc.9740.1.A1_at	1.43 q < 0.000000128	1.08 q < 1	1.37 q < 0.265	2.36 q < 0.0000000982	1.64 q < 0.0221	1.05 q < 1
NANS	N-acetylneuraminic acid synthase	Ssc.30961.1.A1_at	-1.31 q < 0.000000145	-2.01 q < 0.00000000958	-1.4 q < 0.0387	-1.24 q < 0.268	-1.09 q < 1	-1.03 q < 1
Q8TEH8	-	Ssc.21221.1.S1_at	-1.26 q < 0.000000197	-1.22 q < 0.387	-1.28 q < 0.11	-1.07 q < 0.743	-1.41 q < 0.009	-1.34 q < 0.0343
C12orf57	chromosome 12 open reading frame 57	Ssc.15584.1.S1_at	-1.3 q < 0.000000197	-1.17 q < 0.838	-1.43 q < 0.015	-1.39 q < 0.0243	-1.28 q < 0.422	-1.22 q < 0.6
EFHD1	EF-hand domain family, member D1	Ssc.27399.1.S1_at	1.35 q < 0.000000239	-1.03 q < 1	1.33 q < 0.216	1.64 q < 0.00141	1.59 q < 0.00637	1.33 q < 0.277
ERVWE1	endogenous retroviral family W, env(C7), member 1	Ssc.4026.1.S1_at	-1.35 q < 0.000000239	1.02 q < 1	-1.61 q < 0.00277	-1.38 q < 0.0795	-1.35 q < 0.35	-1.51 q < 0.0164
SLC3A2	solute carrier family 3 (activators of dibasic and neutral amino acid transport), member 2	Ssc.2330.1.S1_at	-1.33 q < 0.000000341	-1.27 q < 0.441	-1.45 q < 0.03	-1.85 q < 0.00000466	-1.02 q < 1	-1.18 q < 1
TBC1D22A	TBC1 domain family, member 22A	Ssc.30377.1.A1_at	1.23 q < 0.0000004	1.38 q < 0.00461	1.13 q < 0.677	1.24 q < 0.102	1.05 q < 1	1.38 q < 0.00451
PEG10	paternally expressed 10	Ssc.24007.1.S1_at	-1.37 q < 0.000000447	-1.13 q < 1	-1.23 q < 0.615	-1.97 q < 0.00000927	-1.16 q < 1	-1.54 q < 0.025
PEG10	paternally expressed 10	Ssc.13476.1.A1_at	-1.32 q < 0.00289	1.31 q < 0.848	-2.08 q < 0.00131	-1.44 q < 0.23	-1.13 q < 1	-1.55 q < 0.205
BAT1	HLA-B associated transcript 1	Ssc.12005.1.A1_at	1.77 q < 0.000000462	1.43 q < 0.838	2.34 q < 0.00924	1.6 q < 0.263	1.26 q < 1	2.59 q < 0.00184
BAT1	HLA-B associated transcript 1	Ssc.11719.1.A1_at	-1.09 q < 0.524	1.06 q < 1	-1.54 q < 0.0286	1.12 q < 0.692	-1.06 q < 1	-1.09 q < 1
OSBPL10	oxysterol binding protein-like 10	Ssc.27055.1.A1_at	-1.24 q < 0.000000464	-1.08 q < 1	-1.41 q < 0.00397	-1.16 q < 0.369	-1.34 q < 0.0437	-1.25 q < 0.213
SNX10	sorting nexin 10	Ssc.11006.1.S1_at	1.39 q < 0.000000481	1.02 q < 1	1.36 q < 0.265	1.28 q < 0.326	1.62 q < 0.0167	1.8 q < 0.000564
TMCC2	transmembrane and coiled-coil domain family 2	Ssc.14043.1.A1_at	-1.29 q < 0.000000481	-1.39 q < 0.0478	-1.25 q < 0.31	-1.37 q < 0.0377	-1.36 q < 0.119	-1.1 q < 1
DDEF2	ArfGAP with SH3 domain, ankyrin repeat and PH domain 2	Ssc.5337.1.S1_at	-1.37 q < 0.000000486	-1.11 q < 1	-1.7 q < 0.00145	-1.27 q < 0.312	-1.75 q < 0.000749	-1.14 q < 1
NAV2	neuron navigator 2	Ssc.27911.1.S1_at	-1.84 q < 0.000000535	1.01 q < 1	-1.72 q < 0.304	-2.49 q < 0.0068	-2.54 q < 0.0103	-1.96 q < 0.136
NAV2	neuron navigator 2	Ssc.5613.1.A1_at	1.21 q < 0.0392	1.24 q < 0.943	-1.05 q < 0.973	1.56 q < 0.0541	1.29 q < 1	1.08 q < 1
DCX	doublecortin	Ssc.27479.1.A1_at	-1.28 q < 0.00000065	-1.04 q < 1	-1.15 q < 0.707	-1.58 q < 0.000283	-1.28 q < 0.444	-1.42 q < 0.0161
DCX	doublecortin	Ssc.19017.1.A1_at	-1.22 q < 0.00674	-1.13 q < 1	-1.13 q < 0.876	-1.31 q < 0.251	-1.3 q < 0.866	-1.23 q < 0.93
CPE	carboxypeptidase E	Ssc.5464.1.A1_at	-1.73 q < 0.000000651	1.27 q < 1	-1.92 q < 0.0744	-1.61 q < 0.225	-2.97 q < 0.000129	-2.11 q < 0.0255
MTMR1	myotubularin related protein 1	Ssc.29318.1.A1_at	1.25 q < 0.000000809	1.34 q < 0.0457	1.07 q < 0.91	1.35 q < 0.0234	1.29 q < 0.187	1.21 q < 0.487
APPL2	adaptor protein, phosphotyrosine interaction, PH domain and leucine zipper containing 2	Ssc.18176.1.S1_at	-1.33 q < 0.00000106	-1.2 q < 0.853	-1.59 q < 0.00446	-1.18 q < 0.507	-1.26 q < 0.901	-1.47 q < 0.0335
APPL2	amyloid beta (A4) precursor-like protein 2	Ssc.18176.1.S1_at	-1.33 q < 0.00000106	-1.2 q < 0.853	-1.59 q < 0.00446	-1.18 q < 0.507	-1.26 q < 0.901	-1.47 q < 0.0335
KIF26A	kinesin family member 26A	Ssc.11712.1.A1_at	-1.24 q < 0.0000013	-1.22 q < 0.353	-1.39 q < 0.00879	-1.32 q < 0.0341	-1.1 q < 1	-1.17 q < 0.726
CBX1	chromobox homolog 1	Ssc.27174.1.S1_at	-1.24 q < 0.00000145	-1.1 q < 1	-1.32 q < 0.0485	-1.32 q < 0.0329	-1.22 q < 0.631	-1.24 q < 0.283
NP_002127	-	Ssc.17290.1.A1_at	1.34 q < 0.0000017	1.69 q < 0.00122	1.53 q < 0.0187	1.24 q < 0.352	1.19 q < 1	1.13 q < 1
CEPT1	choline/ethanolamine phosphotransferase 1	Ssc.30316.1.A1_at	1.31 q < 0.00000203	1.49 q < 0.0228	1.25 q < 0.466	1.59 q < 0.0026	1.19 q < 1	1.12 q < 1
STX3	syntaxin 3	Ssc.2000.1.A1_at	1.39 q < 0.00000229	1.04 q < 1	1.25 q < 0.63	1.88 q < 0.000359	1.62 q < 0.0309	1.31 q < 0.628
CPEB4	cytoplasmic polyadenylation element binding protein 4	Ssc.8038.3.S1_at	1.22 q < 0.0000023	1.09 q < 1	1.2 q < 0.335	1.31 q < 0.0265	1.16 q < 1	1.34 q < 0.0159
CPEB4	cytoplasmic polyadenylation element binding protein 4	Ssc.24187.1.S1_at	-1.09 q < 0.213	-1.01 q < 1	-1.4 q < 0.0159	-1.09 q < 0.694	1.02 q < 1	-1.02 q < 1
NGLY1	N-glycanase 1	Ssc.26695.1.S1_at	1.4 q < 0.00000246	1.06 q < 1	1.24 q < 0.663	1.59 q < 0.0243	1.23 q < 1	2.07 q < 0.0000277
NGLY1	N-glycanase 1	Ssc.13314.1.A1_at	1.29 q < 0.00423	1.11 q < 1	1.03 q < 0.988	1.57 q < 0.0711	1.11 q < 1	1.78 q < 0.0126
CHIC1	cysteine-rich hydrophobic domain 1	Ssc.26747.1.A1_at	1.25 q < 0.00000249	1.08 q < 1	1.15 q < 0.695	1.5 q < 0.000784	1.42 q < 0.0113	1.14 q < 1
C12orf64	chromosome 12 open reading frame 64	Ssc.24631.1.S1_at	1.57 q < 0.0000027	-1.35 q < 0.866	1.95 q < 0.0205	2.34 q < 0.000645	1.7 q < 0.223	1.68 q < 0.159
ANP32B	acidic (leucine-rich) nuclear phosphoprotein 32 family, member B	Ssc.28928.1.A1_at	-1.25 q < 0.00000279	-1.16 q < 0.853	-1.39 q < 0.0178	-1.34 q < 0.041	-1.07 q < 1	-1.31 q < 0.101
EFEMP2	EGF-containing fibulin-like extracellular matrix protein 2	Ssc.4267.1.A1_at	-1.34 q < 0.00000331	-1.53 q < 0.0338	-1.58 q < 0.0131	-1.2 q < 0.474	-1.15 q < 1	-1.32 q < 0.44
EFEMP2	EGF-containing fibulin-like extracellular matrix protein 2	Ssc.4267.3.S1_at	-1.17 q < 0.0358	-1.27 q < 0.543	-1.31 q < 0.301	-1.1 q < 0.731	-1.05 q < 1	-1.12 q < 1
KLF8	Kruppel-like factor 8	Ssc.10105.1.A1_at	1.16 q < 0.00000349	1.09 q < 0.997	1.31 q < 0.00131	1.27 q < 0.00406	1.08 q < 1	1.06 q < 1
FGL2	fibrinogen-like 2	Ssc.12579.1.A1_s_at	-1.74 q < 0.00000352	-1.33 q < 1	-1.32 q < 0.824	-2.3 q < 0.0126	-1.72 q < 0.569	-2.28 q < 0.02

Gene Name	Gene Description	Probe Set ID	Breed MS-WC	Day 25 MS-WC	Day 45 MS-WC	Day 65 MS-WC	Day 85 MS-WC	Day 105 MS-WC
FGL2	fibrinogen-like 2	SscAffx.9.1.S1_at	-1.47 q < 0.00626	-1.06 q < 1	-1.15 q < 0.94	-1.85 q < 0.144	-1.41 q < 1	-2.14 q < 0.0693
FGL2	fibrinogen-like 2	Ssc.22050.1.S1_s_at	-1.32 q < 0.0302	1.03 q < 1	-1.11 q < 0.947	-1.45 q < 0.375	-1.34 q < 1	-1.93 q < 0.0642
PUS7L	pseudouridylate synthase 7 homolog (S. cerevisiae)-like	Ssc.9977.1.A1_at	-1.27 q < 0.00000365	-1.63 q < 0.000172	-1.38 q < 0.0459	-1.06 q < 0.83	-1.19 q < 1	-1.17 q < 0.973
WDR35	WD repeat domain 35	Ssc.8078.1.A1_at	-1.3 q < 0.00000464	-1.27 q < 0.457	-1.49 q < 0.0177	-1.17 q < 0.495	-1.58 q < 0.00537	-1.06 q < 1
CD2BP2	CD2 (cytoplasmic tail) binding protein 2	Ssc.19558.1.S1_at	-1.18 q < 0.0000056	-1.17 q < 0.391	-1.25 q < 0.0396	-1.1 q < 0.507	-1.09 q < 1	-1.27 q < 0.0285
CD2BP2	CD2 (cytoplasmic tail) binding protein 2	Ssc.19558.2.S1_a_at	-1.14 q < 0.00641	-1.14 q < 0.855	-1.19 q < 0.379	-1.1 q < 0.576	-1.05 q < 1	-1.21 q < 0.403
APOBEC3H	apolipoprotein B mRNA editing enzyme, catalytic polypeptide-like 3H	Ssc.5332.1.S1_at	-1.2 q < 0.00000573	-1.35 q < 0.0091	-1.15 q < 0.524	-1.1 q < 0.587	-1.11 q < 1	-1.31 q < 0.025
RPL12	ribosomal protein L12	Ssc.939.1.A1_at	-1.27 q < 0.00000767	-1.15 q < 1	-1.4 q < 0.0469	-1.28 q < 0.175	-1.13 q < 1	-1.44 q < 0.0242
RPL38	ribosomal protein L38	Ssc.28256.1.A1_at	-1.41 q < 0.000011	-1 q < 1	-2.74 q < 0.0000000233	-1.23 q < 0.529	1.05 q < 1	-1.75 q < 0.0126
EMD	emerin	Ssc.4513.2.A1_a_at	-1.21 q < 0.0000116	-1.47 q < 0.00051	-1.32 q < 0.0309	1.02 q < 0.921	-1.09 q < 1	-1.25 q < 0.205
OSBPL3	oxysterol binding protein-like 3	Ssc.27522.1.S1_at	-1.23 q < 0.0000132	-1 q < 1	-1.04 q < 0.964	-1.51 q < 0.000797	-1.41 q < 0.0197	-1.3 q < 0.144
OSBPL3	oxysterol binding protein-like 3	Ssc.21874.1.S1_at	-1.24 q < 0.000462	-1.11 q < 1	-1.08 q < 0.923	-1.36 q < 0.11	-1.35 q < 0.384	-1.36 q < 0.205
OSBPL3	oxysterol binding protein-like 3	Ssc.6070.1.A1_at	-1.16 q < 0.0453	-1.08 q < 1	1.03 q < 0.985	-1.38 q < 0.109	-1.34 q < 0.495	-1.1 q < 1
FOXO1	forkhead box O1	Ssc.11251.1.A1_at	1.2 q < 0.0000133	1.11 q < 1	1.14 q < 0.634	1.33 q < 0.0191	1.14 q < 1	1.31 q < 0.0373
FOXO1	forkhead box O1	Ssc.73.1.S1_at	-1.21 q < 0.000494	-1.04 q < 1	-1.12 q < 0.842	-1.07 q < 0.797	-1.48 q < 0.0115	-1.42 q < 0.0267
NP_057626	-	Ssc.8803.1.A1_at	1.46 q < 0.0000177	1.9 q < 0.01	1.05 q < 0.978	1.78 q < 0.0216	1.38 q < 1	1.35 q < 0.802
TSC22D2	TSC22 domain family, member 2	Ssc.21290.1.S1_at	-1.32 q < 0.0000232	-1.25 q < 0.735	1.04 q < 0.973	-1.25 q < 0.388	-1.65 q < 0.00827	-1.62 q < 0.00807
KIAA1128	family with sequence similarity 190, member B	Ssc.24593.1.A1_at	-1.17 q < 0.0000237	1.04 q < 1	-1.26 q < 0.0353	-1.25 q < 0.0336	-1.22 q < 0.185	-1.16 q < 0.484
RPL10	ribosomal protein L10	Ssc.9130.1.A1_at	-1.13 q < 0.0000292	-1.03 q < 1	-1.24 q < 0.00715	-1.08 q < 0.505	-1.02 q < 1	-1.3 q < 0.000227
RPL10	ribosomal protein L15	Ssc.9130.1.A1_at	-1.13 q < 0.0000292	-1.03 q < 1	-1.24 q < 0.00715	-1.08 q < 0.505	-1.02 q < 1	-1.3 q < 0.000227
HSDL2	hydroxysteroid dehydrogenase like 2	Ssc.22023.1.S1_at	-1.3 q < 0.0000302	1.25 q < 0.681	-1.45 q < 0.0629	-1.34 q < 0.156	-1.55 q < 0.0221	-1.52 q < 0.0219
HSDL2	hydroxysteroid dehydrogenase like 2	Ssc.29859.1.A1_at	-1.16 q < 0.0166	1.06 q < 1	-1.22 q < 0.488	-1.18 q < 0.457	-1.38 q < 0.119	-1.13 q < 1
KIAA0415	KIAA0415	Ssc.27938.1.S1_at	-1.28 q < 0.0000333	-1.15 q < 1	-1.47 q < 0.0291	-1.12 q < 0.66	-1.55 q < 0.0112	-1.16 q < 1
BBX	bobby sox homolog (Drosophila)	Ssc.1026.1.S1_at	-1.27 q < 0.0000421	-1.14 q < 1	-1.47 q < 0.0223	-1.12 q < 0.657	-1.47 q < 0.0446	-1.2 q < 0.937
BBX	bobby sox homolog (Drosophila)	Ssc.5318.1.A1_at	-1.16 q < 0.0161	-1.25 q < 0.512	-1.11 q < 0.879	-1.09 q < 0.723	-1.29 q < 0.53	-1.1 q < 1
RPL32	ribosomal protein L32	Ssc.3284.1.A1_at	-1.15 q < 0.0000423	1.05 q < 1	-1.29 q < 0.004	-1.08 q < 0.563	-1.12 q < 1	-1.32 q < 0.00111
TM7SF2	transmembrane 7 superfamily member 2	Ssc.4715.1.S1_at	1.57 q < 0.000043	1.42 q < 0.809	-1.29 q < 0.814	2.19 q < 0.0089	2 q < 0.069	1.99 q < 0.0449
CALD1	caldesmon 1	Ssc.17313.2.S1_at	-1.41 q < 0.0000566	-1.18 q < 1	-1.7 q < 0.0398	-1.68 q < 0.0349	-1.27 q < 1	-1.3 q < 0.935
CALD1	caldesmon 1	Ssc.17313.1.A1_at	-1.43 q < 0.000036	-1.16 q < 1	-1.94 q < 0.00454	-1.45 q < 0.215	-1.32 q < 1	-1.39 q < 0.643
STXBP5L	syntaxin binding protein 5-like	Ssc.4671.1.A1_at	1.29 q < 0.0000809	1.07 q < 1	1.49 q < 0.0389	1.66 q < 0.00204	1.3 q < 0.734	1.03 q < 1
GCNT2	glucosaminyl (N-acetyl) transferase 2, I-branching enzyme (I blood group)	Ssc.12815.1.A1_at	1.26 q < 0.000106	1.07 q < 1	1.58 q < 0.00379	1.62 q < 0.00132	1.08 q < 1	1.07 q < 1
PKN2	protein kinase N2	Ssc.19164.1.A1_at	-1.26 q < 0.000124	-1.05 q < 1	-1.69 q < 0.000684	1.2 q < 0.441	-2.06 q < 0.000000197	-1.05 q < 1
PKN2	protein kinase N2	Ssc.9980.1.S1_at	-1.37 q < 0.0000375	-1.53 q < 0.12	-1.62 q < 0.0398	-1.05 q < 0.901	-1.48 q < 0.33	-1.27 q < 0.923
TXNIP	thioredoxin interacting protein	Ssc.16648.1.S1_at	-1.25 q < 0.000158	-2.38 q < 0.0000000000238	-1.03 q < 0.982	-1.41 q < 0.0473	-1.02 q < 1	1.14 q < 1
RNF167	ring finger protein 167	Ssc.20226.1.S1_at	-1.27 q < 0.00016	-1.09 q < 1	-1.57 q < 0.00869	1.03 q < 0.925	-1.26 q < 0.997	-1.56 q < 0.0116
TC2N	tandem C2 domains, nuclear	Ssc.16844.1.A1_at	1.41 q < 0.000167	1.15 q < 1	1.78 q < 0.0309	1.8 q < 0.0197	1.07 q < 1	1.4 q < 0.656
WFDC2	WAP four-disulfide core domain 2	Ssc.27921.1.S1_at	1.35 q < 0.000176	-1.03 q < 1	1.27 q < 0.646	1.81 q < 0.004	1.15 q < 1	1.73 q < 0.0138
WFDC2	WAP four-disulfide core domain 2	Ssc.3968.1.S1_at	1.78 q < 0.0755	-1.37 q < 1	2.16 q < 0.659	4.55 q < 0.0472	1.72 q < 1	1.43 q < 1
ACTG1	actin, gamma 1	AFFX-Ssc-actin-3_at	-1.11 q < 0.000178	1 q < 1	-1.14 q < 0.188	-1.18 q < 0.0374	-1.04 q < 1	-1.2 q < 0.025
ACTG1	actin, gamma 1	Ssc.13874.1.S1_at	-1.1 q < 0.00461	1.01 q < 1	-1.14 q < 0.344	-1.16 q < 0.14	-1.03 q < 1	-1.19 q < 0.117
ACTG1	actin, gamma 1	AFFX-Ssc-actin-5_x_at	-1.25 q < 0.0354	1.08 q < 1	-1.38 q < 0.494	-1.37 q < 0.344	-1.05 q < 1	-1.64 q < 0.112
ACTG1	actin, gamma 1	AFFX-Ssc-actin-M_at	-1.17 q < 0.0468	1.03 q < 1	-1.23 q < 0.613	-1.19 q < 0.511	-1.06 q < 1	-1.46 q < 0.0907
ACTG1	actin, gamma 1	AFFX-Ssc-actin-5_at	-1.35 q < 0.066	1.05 q < 1	-1.35 q < 0.816	-1.41 q < 0.518	-1.04 q < 1	-2.36 q < 0.0296
IGF2BP1	insulin-like growth factor 2 mRNA binding protein 1	Ssc.26377.1.A1_at	-1.22 q < 0.000198	-1.47 q < 0.00803	-1.47 q < 0.00821	-1.13 q < 0.566	-1.04 q < 1	-1.06 q < 1
PARP11	poly (ADP-ribose) polymerase family, member 11	Ssc.28870.1.S1_at	1.17 q < 0.000274	1.21 q < 0.348	1.35 q < 0.0125	1 q < 0.995	1.01 q < 1	1.33 q < 0.0206
ARSE	arylsulfatase E (chondrodysplasia punctata 1)	Ssc.4033.2.S1_at	1.51 q < 0.00031	-1.37 q < 0.924	2.22 q < 0.0105	2.71 q < 0.000295	1.4 q < 1	1.27 q < 1

Gene Name	Gene Description	Probe Set ID	Breed MS-WC	Day 25 MS-WC	Day 45 MS-WC	Day 65 MS-WC	Day 85 MS-WC	Day 105 MS-WC
ARSE	arylsulfatase E (chondrodysplasia punctata 1)	Ssc.1707.1.S1_at	1.13 q < 0.273	-1.29 q < 0.749	1.3 q < 0.52	1.56 q < 0.0463	1.08 q < 1	1.1 q < 1
PPP1R1A	protein phosphatase 1, regulatory (inhibitor) subunit 1A	Ssc.21985.1.S1_at	1.26 q < 0.000385	1.5 q < 0.0388	1.17 q < 0.792	1.14 q < 0.619	1.03 q < 1	1.55 q < 0.0164
MFSD1	major facilitator superfamily domain containing 1	Ssc.18528.2.A1_at	-1.33 q < 0.000435	-1.18 q < 1	-1.85 q < 0.00393	1.1 q < 0.79	-1.15 q < 1	-1.83 q < 0.00466
EPAS1	endothelial PAS domain protein 1	Ssc.5260.1.A1_at	1.18 q < 0.000836	-1.15 q < 0.91	1.18 q < 0.54	1.41 q < 0.0105	1.12 q < 1	1.44 q < 0.00691
C10orf72	chromosome 10 open reading frame 72	Ssc.16442.1.S1_at	-1.23 q < 0.000986	-1.45 q < 0.0457	-1.47 q < 0.0286	-1.24 q < 0.321	-1.09 q < 1	1.05 q < 1
FHIT	fragile histidine triad gene	Ssc.19442.1.A1_at	1.15 q < 0.00138	-1.03 q < 1	1.28 q < 0.0481	1.28 q < 0.0341	1.12 q < 1	1.12 q < 1
RCAN2	regulator of calcineurin 2	Ssc.18504.1.S1_at	1.18 q < 0.00173	1.16 q < 0.852	1.1 q < 0.878	1.35 q < 0.0428	-1.04 q < 1	1.38 q < 0.034
SNX12	sorting nexin 12	Ssc.2628.1.S1_at	-1.24 q < 0.00174	-1.09 q < 1	-1.63 q < 0.0053	-1.12 q < 0.695	1.03 q < 1	-1.51 q < 0.0361
RPS12	ribosomal protein S12	Ssc.13774.1.S1_at	-1.13 q < 0.00374	-1.04 q < 1	-1.27 q < 0.039	1.03 q < 0.879	-1.04 q < 1	-1.35 q < 0.00274
RPS12	mitochondrial ribosomal protein S12	Ssc.13774.1.S1_at	-1.13 q < 0.00374	-1.04 q < 1	-1.27 q < 0.039	1.03 q < 0.879	-1.04 q < 1	-1.35 q < 0.00274
KDELC2	KDEL (Lys-Asp-Glu-Leu) containing 2	Ssc.31019.1.S1_at	-1.11 q < 0.00432	-1.01 q < 1	1 q < 0.997	1.03 q < 0.839	-1.29 q < 0.00528	-1.31 q < 0.00115
RPS5	ribosomal protein S5	Ssc.792.1.S1_at	-1.11 q < 0.00496	-1 q < 1	-1.24 q < 0.0449	-1.09 q < 0.568	-1.01 q < 1	-1.25 q < 0.0306
SLC25A37	solute carrier family 25, member 37	Ssc.21282.2.S1_at	1.19 q < 0.00576	1.59 q < 0.00196	1.13 q < 0.842	1.64 q < 0.000631	-1.14 q < 1	-1.1 q < 1
C20orf199	non-protein coding RNA 275	Ssc.1256.1.A1_at	1.2 q < 0.0104	-1.22 q < 0.837	-1.05 q < 0.954	1.22 q < 0.414	1.54 q < 0.0324	1.68 q < 0.00154
ZC3H11A	zinc finger CCCH-type containing 11A	Ssc.19135.1.A1_at	-1.1 q < 0.0114	-1.31 q < 0.00394	-1.34 q < 0.00117	1.1 q < 0.5	-1.1 q < 1	1.07 q < 1
IFI44L	interferon-induced protein 44-like	Ssc.10588.1.A1_at	-1.23 q < 0.0368	-1.77 q < 0.0216	-1.68 q < 0.0449	-1.41 q < 0.247	1.33 q < 1	1.11 q < 1
IFI44L	interferon-induced protein 44-like	Ssc.10593.1.S1_at	-1.14 q < 0.0935	-1.57 q < 0.0111	-1.43 q < 0.0752	-1.18 q < 0.521	1.26 q < 1	1.07 q < 1
IGK	immunoglobulin kappa locus	Ssc.11208.1.S1_at	-1.46 q < 0	-1.04 q < 1	1.01 q < 0.996	1.07 q < 0.755	1 q < 1	-6.98 q < 0
IGLC1	immunoglobulin lambda constant 1 (Mcg marker)	Ssc.19946.1.S1_at	-2.09 q < 0	1.01 q < 1	-1.05 q < 0.944	1.06 q < 0.816	-1.03 q < 1	-39.57 q < 0
NAP1L3	nucleosome assembly protein 1-like 3	Ssc.8352.1.A1_at	-1.52 q < 0	-2.7 q < 0	-1.36 q < 0.14	-1.4 q < 0.0591	-1.34 q < 0.351	-1.17 q < 1
IGHG	-	Ssc.16359.2.S1_s_at	-1.53 q < 0.000000000000191	1.01 q < 1	-1.06 q < 0.946	1 q < 0.992	-1.03 q < 1	-7.85 q < 0
PLEKHA8	pleckstrin homology domain containing, family A (phosphoinositide binding specific) member 8	Ssc.1878.1.S1_at	-1.49 q < 0.0000000000111	-1.36 q < 0.243	-2.14 q < 0.0000000996	-1.3 q < 0.222	-1.46 q < 0.0925	-1.35 q < 0.271
PLEKHA8	pleckstrin homology domain containing, family A (phosphoinositide binding specific) member 8	Ssc.30797.1.S1_at	-1.27 q < 0.0000074	-1.06 q < 1	-1.38 q < 0.0542	-1.25 q < 0.23	-1.51 q < 0.00643	-1.18 q < 0.936
TAF9B	TAF9B RNA polymerase II, TATA box binding protein (TBP)-associated factor, 31kDa	Ssc.27986.1.A1_at	-1.28 q < 0.000000000759	-1.78 q < 0.00000000076	-1.13 q < 0.686	-1.25 q < 0.0891	-1.17 q < 0.951	-1.16 q < 0.746
MTUS1	microtubule associated tumor suppressor 1	Ssc.1355.1.S1_at	-1.41 q < 0.00000000126	-2.13 q < 0.0000000251	-1.24 q < 0.513	-1.12 q < 0.65	-1.42 q < 0.124	-1.34 q < 0.227
CALM2	calmodulin 2 (phosphorylase kinase, delta)	Ssc.30607.1.A1_at	-1.41 q < 0.00000000226	-1.21 q < 0.807	-1.31 q < 0.267	-2 q < 0.000000522	-1.47 q < 0.0587	-1.18 q < 1
CALM2	calmodulin 2 (phosphorylase kinase, delta)	Ssc.11516.1.S1_at	-1.11 q < 0.0411	1.04 q < 1	-1.13 q < 0.7	-1.27 q < 0.0776	-1.2 q < 0.744	-1.02 q < 1
C6orf150	chromosome 6 open reading frame 150	Ssc.22696.1.S1_at	-1.32 q < 0.00000000238	-1.31 q < 0.11	-1.26 q < 0.202	-1.35 q < 0.0336	-1.35 q < 0.0841	-1.32 q < 0.0907
PDE1A	phosphodiesterase 1A, calmodulin-dependent	Ssc.9496.1.A1_at	1.24 q < 0.00000000263	1.04 q < 1	1.19 q < 0.243	1.61 q < 0.0000000211	1.2 q < 0.389	1.21 q < 0.212
PDE1A	phosphodiesterase 1A, calmodulin-dependent	Ssc.7144.1.S1_at	-1.04 q < 0.709	1.36 q < 0.0144	-1.16 q < 0.541	-1.15 q < 0.401	-1.25 q < 0.278	1 q < 1
SRPX2	sushi-repeat-containing protein, X-linked 2	Ssc.4899.1.S1_at	-1.33 q < 0.000000003	-2.13 q < 0.00000000000937	-1.13 q < 0.777	-1.05 q < 0.834	-1.28 q < 0.401	-1.28 q < 0.252
IGJ	immunoglobulin J polypeptide, linker protein for immunoglobulin alpha and mu polypeptides	Ssc.16506.1.A1_at	-1.4 q < 0.00000000504	-1.06 q < 1	1.06 q < 0.943	1.01 q < 0.984	1.06 q < 1	-5.88 q < 0
EFR3A	EFR3 homolog A (S. cerevisiae)	Ssc.10435.1.A1_at	-1.36 q < 0.00000000563	-1.31 q < 0.248	-1.78 q < 0.0000153	-1.2 q < 0.394	-1.41 q < 0.0814	-1.19 q < 0.903
EFR3A	EFR3 homolog A (S. cerevisiae)	Ssc.29551.1.A1_at	1.06 q < 0.497	-1.04 q < 1	-1.1 q < 0.804	1.32 q < 0.0185	1.04 q < 1	1.1 q < 1
GRIP2	glutamate receptor interacting protein 2	Ssc.6492.1.S1_at	1.21 q < 0.00000000685	1.36 q < 0.000441	1.2 q < 0.142	1.17 q < 0.17	1.17 q < 0.567	1.17 q < 0.354
C6orf103	chromosome 6 open reading frame 103	Ssc.3761.1.A1_at	1.61 q < 0.00000000924	2.39 q < 0.0000342	1.22 q < 0.82	1.46 q < 0.205	1.57 q < 0.27	1.62 q < 0.108
C6orf103	chromosome 6 open reading frame 103	Ssc.4492.1.A1_at	-1.15 q < 0.0208	-1.1 q < 1	-1.45 q < 0.00876	1.08 q < 0.749	-1.19 q < 1	-1.12 q < 1
RALA	v-ral simian leukemia viral oncogene homolog A (ras related)	Ssc.1560.1.S1_at	-1.31 q < 0.0000000198	-1.26 q < 0.314	-1.48 q < 0.00385	-1.24 q < 0.207	-1.26 q < 0.531	-1.33 q < 0.102
RALA	v-ral simian leukemia viral oncogene homolog A (ras related)	Ssc.1560.2.S1_at	-1.25 q < 0.00000035	-1.2 q < 0.484	-1.39 q < 0.00869	-1.18 q < 0.317	-1.23 q < 0.495	-1.25 q < 0.224

Gene Name	Gene Description	Probe Set ID	Breed MS-WC	Day 25 MS-WC	Day 45 MS-WC	Day 65 MS-WC	Day 85 MS-WC	Day 105 MS-WC
NSD1	nuclear receptor binding SET domain protein 1	Ssc.20770.1.S1_at	1.27 q < 0.000000023	1.61 q < 0.00000387	1.08 q < 0.886	1.24 q < 0.128	1.23 q < 0.501	1.23 q < 0.325
HERPUD2	HERPUD family member 2	Ssc.27248.1.S1_at	-1.34 q < 0.0000000313	-1.27 q < 0.375	-1.25 q < 0.374	-1.37 q < 0.0508	-1.49 q < 0.0142	-1.32 q < 0.206
COL6A1	collagen, type VI, alpha 1	Ssc.5895.1.A2_at	-1.69 q < 0.0000000423	-1.77 q < 0.0928	-1.97 q < 0.0179	-1.61 q < 0.145	-1.42 q < 1	-1.72 q < 0.125
COL6A1	collagen, type VI, alpha 1	Ssc.5895.1.A1_at	-1.55 q < 0.00000603	-1.63 q < 0.217	-1.85 q < 0.039	-1.42 q < 0.335	-1.31 q < 1	-1.58 q < 0.309
COL6A1	collagen, type VI, alpha 1	Ssc.21754.1.A1_at	-1.24 q < 0.0151	-1.35 q < 0.536	-1.46 q < 0.177	-1.16 q < 0.661	-1.1 q < 1	-1.15 q < 1
GOLGB1	golgin B1	Ssc.31053.1.A1_at	-1.24 q < 0.0000000423	-1.25 q < 0.123	-1.26 q < 0.0781	-1.38 q < 0.00269	-1.22 q < 0.423	-1.11 q < 1
SLC20A1	solute carrier family 20 (phosphate transporter), member 1	Ssc.1527.2.A1_at	-1.6 q < 0.0000000476	-1.68 q < 0.0944	-1.46 q < 0.336	-1.23 q < 0.584	-1.8 q < 0.0501	-1.97 q < 0.00637
TROVE2	TROVE domain family, member 2	Ssc.29839.1.A1_at	1.27 q < 0.0000000562	1.27 q < 0.187	1.3 q < 0.0869	1.33 q < 0.0329	1.23 q < 0.555	1.24 q < 0.307
MAP7D3	MAP7 domain containing 3	Ssc.10722.1.A1_at	1.23 q < 0.0000000621	1.19 q < 0.376	1.27 q < 0.057	1.31 q < 0.0143	1.2 q < 0.531	1.19 q < 0.391
TXNDC13	thioredoxin-related transmembrane protein 4	Ssc.6827.1.A1_at	-1.37 q < 0.0000000837	-1.25 q < 0.648	-1.51 q < 0.0211	-1.42 q < 0.0592	-1.34 q < 0.42	-1.36 q < 0.215
ALOX5AP	arachidonate 5-lipoxygenase-activating protein	Ssc.5822.1.S1_at	-1.35 q < 0.0000000989	-1.19 q < 0.869	-1.22 q < 0.548	-1.26 q < 0.261	-1.41 q < 0.117	-1.72 q < 0.000121
CSTA	cystatin A (stefin A)	Ssc.645.1.S1_at	1.21 q < 0.000000116	-1.07 q < 1	1.99 q < 0	1.2 q < 0.134	1.11 q < 1	1.06 q < 1
CSTA	cystatin A (stefin A)	Ssc.646.1.S1_at	1.13 q < 0.0116	-1.02 q < 1	1.52 q < 0.0000743	1.02 q < 0.924	1.14 q < 1	1.07 q < 1
OCIAD2	OCIA domain containing 2	Ssc.22715.1.S1_at	-1.6 q < 0.000000126	-1.68 q < 0.104	-1.89 q < 0.0159	-1.6 q < 0.105	-1.7 q < 0.142	-1.2 q < 1
SYN3	synapsin III	Ssc.3880.1.S1_at	-1.7 q < 0.000000136	-1.61 q < 0.335	-1.33 q < 0.731	-1.59 q < 0.189	-2.63 q < 0.000282	-1.6 q < 0.367
HTRA4	HtrA serine peptidase 4	Ssc.7990.1.A1_at	-1.4 q < 0.000000145	-1.39 q < 0.221	-1.63 q < 0.00708	-1.3 q < 0.253	-1.31 q < 0.769	-1.38 q < 0.252
LAMP2	lysosomal-associated membrane protein 2	Ssc.1955.1.A1_at	-1.4 q < 0.000000157	-1.23 q < 0.809	-2.04 q < 0.0000053	-1.21 q < 0.484	-1.18 q < 1	-1.5 q < 0.0533
LAMP2	lysosomal-associated membrane protein 2	Ssc.1955.2.S1_at	-1.08 q < 0.232	-1 q < 1	-1.4 q < 0.00958	1.05 q < 0.839	-1.07 q < 1	-1.05 q < 1
TEAD1	TEA domain family member 1 (SV40 transcriptional enhancer factor)	Ssc.13762.1.S1_at	-1.42 q < 0.000000172	-1.31 q < 0.554	-1.64 q < 0.0114	-1.48 q < 0.0637	-1.46 q < 0.222	-1.26 q < 0.856
TEAD1	TEA domain family member 1 (SV40 transcriptional enhancer factor)	Ssc.2932.1.A1_at	-1.09 q < 0.382	-1.6 q < 0.00336	-1.23 q < 0.53	1.27 q < 0.255	1.03 q < 1	-1.04 q < 1
GNA12	guanine nucleotide binding protein (G protein) alpha 12	Ssc.17298.1.A1_at	-1.31 q < 0.000000179	1.02 q < 1	-1.8 q < 0.00000423	-1.27 q < 0.198	-1.31 q < 0.387	-1.35 q < 0.12
GNA12	guanine nucleotide binding protein (G protein) alpha 12	Ssc.17298.2.S1_at	-1.13 q < 0.0417	1.07 q < 1	-1.42 q < 0.0199	1.06 q < 0.825	-1.2 q < 1	-1.24 q < 0.456
SCARA3	scavenger receptor class A, member 3	Ssc.25203.1.S1_at	-1.26 q < 0.000000207	-1.28 q < 0.169	-1.23 q < 0.283	-1.23 q < 0.184	-1.44 q < 0.00535	-1.16 q < 0.917
SSBP3	single stranded DNA binding protein 3	Ssc.3408.1.A1_at	1.21 q < 0.000000213	1.18 q < 0.399	1.11 q < 0.739	1.29 q < 0.015	1.27 q < 0.0806	1.23 q < 0.16
KLHL24	kelch-like 24 (Drosophila)	Ssc.29392.1.A1_at	1.35 q < 0.000000213	1.09 q < 1	1.41 q < 0.0764	1.55 q < 0.00643	1.39 q < 0.193	1.36 q < 0.201
SYNPO2	synaptopodin 2	Ssc.26929.1.S1_at	-1.47 q < 0.000000263	-3.38 q < 0.00000000000153	-1.46 q < 0.196	-1.21 q < 0.551	1.02 q < 1	-1.19 q < 1
C21orf66	GC-rich sequence DNA-binding factor 1	Ssc.7950.1.A1_at	-1.21 q < 0.000000315	-1.22 q < 0.191	-1.12 q < 0.658	-1.09 q < 0.584	-1.43 q < 0.000295	-1.22 q < 0.184
PIN4	protein (peptidylprolyl cis/trans isomerase) NIMA-interacting, 4 (parvulin)	Ssc.28431.1.A1_at	1.25 q < 0.00000034	1.08 q < 1	1.27 q < 0.123	1.51 q < 0.000137	1.24 q < 0.438	1.17 q < 0.738
PIN4	protein (peptidylprolyl cis/trans isomerase) NIMA-interacting, 4 (parvulin)	Ssc.18633.1.S1_at	1.21 q < 0.000368	1.05 q < 1	1.24 q < 0.403	1.5 q < 0.00409	1.26 q < 0.636	1.08 q < 1
COL5A1	collagen, type V, alpha 1	Ssc.9002.1.A1_at	-1.75 q < 0.000000399	-1.59 q < 0.471	-1.9 q < 0.0852	-1.63 q < 0.217	-1.46 q < 1	-2.28 q < 0.00953
COL5A1	collagen, type V, alpha 1	Ssc.16329.1.S1_at	-1.15 q < 0.0956	-1.63 q < 0.00662	-1.14 q < 0.849	-1.01 q < 0.979	-1.03 q < 1	-1.03 q < 1
CD276	CD276 molecule	Ssc.2042.1.S1_at	-1.28 q < 0.000000418	-1.49 q < 0.00329	-1.33 q < 0.0949	-1.08 q < 0.731	-1.25 q < 0.585	-1.3 q < 0.187
Q9NX89	-	Ssc.3574.1.A1_at	-1.32 q < 0.000000453	-1.24 q < 0.611	-1.51 q < 0.0108	-1.36 q < 0.0818	-1.27 q < 0.747	-1.26 q < 0.531
RPS2	ribosomal protein S2	Ssc.12073.1.A1_at	-1.18 q < 0.000000497	1.03 q < 1	-1.22 q < 0.0641	-1.2 q < 0.0706	-1.11 q < 1	-1.43 q < 0.0000066
C7orf61	chromosome 7 open reading frame 61	Ssc.4346.1.A1_at	1.24 q < 0.000000524	1.15 q < 0.784	1.18 q < 0.434	1.33 q < 0.017	1.22 q < 0.516	1.3 q < 0.0631
CCS	copper chaperone for superoxide dismutase	Ssc.15274.1.S1_a_at	-1.24 q < 0.00000059	-1.01 q < 1	-1.26 q < 0.15	-1.25 q < 0.13	-1.45 q < 0.0022	-1.3 q < 0.0798
FGF23	fibroblast growth factor 23	Ssc.27685.1.S1_at	-1.33 q < 0.000000624	-1.84 q < 0.0000117	-1.29 q < 0.305	-1.16 q < 0.529	-1.09 q < 1	-1.37 q < 0.129
BEX2	brain expressed X-linked 2	Ssc.3714.1.S1_a_at	-1.28 q < 0.00000065	1 q < 1	-1.49 q < 0.00332	-1.24 q < 0.214	-1.38 q < 0.0593	-1.34 q < 0.0905
ILF2	interleukin enhancer binding factor 2, 45kDa	Ssc.27246.1.S1_at	-1.32 q < 0.000000654	-1.02 q < 1	-1.66 q < 0.000749	-1.37 q < 0.0793	-1.29 q < 0.655	-1.37 q < 0.128
RNF19A	ring finger protein 19A	Ssc.29466.1.A1_at	1.26 q < 0.000000697	1.05 q < 1	1.24 q < 0.275	1.38 q < 0.0149	1.32 q < 0.125	1.34 q < 0.0573
FUT2	fucosyltransferase 2 (secretor status included)	Ssc.243.1.S1_at	1.33 q < 0.000000708	1.1 q < 1	1.32 q < 0.234	1.57 q < 0.00367	1.29 q < 0.638	1.42 q < 0.0721
RASSF6	Ras association (RalGDS/AF-6) domain family member 6	Ssc.27147.1.A1_at	1.24 q < 0.000000777	1.12 q < 1	1.3 q < 0.0722	1.52 q < 0.000118	1.2 q < 0.752	1.1 q < 1
RASSF6	Ras association (RalGDS/AF-6) domain family member 6	Ssc.9286.1.A1_at	1.16 q < 0.0145	1.06 q < 1	1.23 q < 0.413	1.41 q < 0.0231	1.03 q < 1	1.1 q < 1
Q8IXQ9	-	Ssc.24915.1.S1_at	1.26 q < 0.000000791	1.29 q < 0.158	1.1 q < 0.854	1.38 q < 0.0194	1.34 q < 0.0873	1.21 q < 0.545

Gene Name	Gene Description	Probe Set ID	Breed MS-WC	Day 25 MS-WC	Day 45 MS-WC	Day 65 MS-WC	Day 85 MS-WC	Day 105 MS-WC
BBS4	Bardet-Biedl syndrome 4	Ssc.15249.1.S1_at	1.21 q < 0.000000808	1.11 q < 0.987	1.2 q < 0.281	1.29 q < 0.0325	1.25 q < 0.187	1.23 q < 0.183
VPS26B	vacuolar protein sorting 26 homolog B (S. pombe)	Ssc.20621.1.S1_at	-1.2 q < 0.00000096	-1.16 q < 0.564	-1.5 q < 0.0000122	-1.07 q < 0.69	-1.2 q < 0.454	-1.13 q < 0.923
IDI1	isopentenyl-diphosphate delta isomerase 1	Ssc.6714.1.A1_at	1.41 q < 0.00000103	1.45 q < 0.18	1.09 q < 0.934	1.83 q < 0.000966	1.37 q < 0.636	1.41 q < 0.288
ARL5A	ADP-ribosylation factor-like 5A	Ssc.27149.1.S1_at	-1.48 q < 0.0000011	-1.13 q < 1	-1.75 q < 0.0208	-1.43 q < 0.212	-1.58 q < 0.202	-1.61 q < 0.0911
DDX21	DEAD (Asp-Glu-Ala-Asp) box polypeptide 21	Ssc.20522.1.S1_at	-1.33 q < 0.0000012	-1.29 q < 0.429	-1.49 q < 0.0232	-1.23 q < 0.364	-1.27 q < 0.836	-1.38 q < 0.139
GPR107	G protein-coupled receptor 107	Ssc.26127.1.S1_at	-1.23 q < 0.00000134	-1.04 q < 1	-1.35 q < 0.0167	-1.2 q < 0.244	-1.32 q < 0.0605	-1.26 q < 0.16
SF3B2	splicing factor 3b, subunit 2, 145kDa	Ssc.5474.1.S1_at	-1.27 q < 0.00000138	-1.16 q < 0.918	-1.4 q < 0.0229	-1.14 q < 0.524	-1.34 q < 0.145	-1.34 q < 0.091
TDRD3	tudor domain containing 3	Ssc.28339.1.A1_at	1.21 q < 0.00000165	1.12 q < 0.901	1.18 q < 0.361	1.3 q < 0.0178	1.23 q < 0.299	1.21 q < 0.305
TDRD3	tudor domain containing 3	Ssc.14320.1.A1_at	-1.18 q < 0.0228	-1.36 q < 0.217	-1.19 q < 0.706	-1.14 q < 0.607	-1.06 q < 1	-1.15 q < 1
UBR2	ubiquitin protein ligase E3 component n-recognin 2	Ssc.22803.1.S1_at	-1.29 q < 0.00000184	-1.55 q < 0.00219	-1.22 q < 0.474	-1.15 q < 0.532	-1.3 q < 0.42	-1.26 q < 0.482
GBE1	glucan (1,4-alpha-), branching enzyme 1	Ssc.8684.1.A1_at	-1.28 q < 0.00000184	-1.18 q < 0.819	-1.41 q < 0.0288	-1.32 q < 0.0902	-1.27 q < 0.564	-1.22 q < 0.613
SEC31A	SEC31 homolog A (S. cerevisiae)	Ssc.11165.3.A1_at	-1.34 q < 0.00000186	-1.46 q < 0.0637	-1.52 q < 0.0223	-1.13 q < 0.65	-1.22 q < 1	-1.4 q < 0.136
SEC31A	SEC31 homolog A (S. cerevisiae)	Ssc.21334.1.S1_at	1.2 q < 0.0101	-1.15 q < 1	1.15 q < 0.828	1.53 q < 0.017	1.24 q < 1	1.31 q < 0.458
SEC31A	SEC31 homolog A (S. cerevisiae)	Ssc.11165.2.S1_at	-1.3 q < 0.00059	-1.26 q < 0.853	-1.56 q < 0.0645	-1.18 q < 0.599	-1.16 q < 1	-1.41 q < 0.325
SEC31A	SEC31 homolog A (S. cerevisiae)	Ssc.11165.1.S1_at	-1.17 q < 0.00347	-1.25 q < 0.38	-1.28 q < 0.186	-1.03 q < 0.916	-1.22 q < 0.927	-1.11 q < 1
NPC2	Niemann-Pick disease, type C2	Ssc.529.1.S1_at	-1.5 q < 0.00000189	-1.27 q < 0.954	-1.32 q < 0.612	-2.14 q < 0.000453	-1.3 q < 1	-1.62 q < 0.113
LSAMP	limbic system-associated membrane protein	Ssc.24997.1.S1_at	-1.24 q < 0.00000203	-1.64 q < 0.00000863	-1.06 q < 0.93	-1.2 q < 0.268	-1.16 q < 1	-1.22 q < 0.41
LSAMP	limbic system-associated membrane protein	Ssc.24997.2.A1_at	-1.22 q < 0.0000616	-1.37 q < 0.0472	-1.1 q < 0.872	-1.27 q < 0.145	-1.19 q < 1	-1.2 q < 0.673
MRPL16	mitochondrial ribosomal protein L16	Ssc.2526.1.S1_at	-1.31 q < 0.00000234	-1.08 q < 1	-1.83 q < 0.00002	-1.18 q < 0.494	-1.24 q < 1	-1.35 q < 0.197
SLC16A3	solute carrier family 16, member 3 (monocarboxylic acid transporter 4)	Ssc.22067.1.A1_at	1.18 q < 0.00000237	1.75 q < 0	1.04 q < 0.94	1.15 q < 0.281	-1.01 q < 1	1.09 q < 1
NEK5	NIMA (never in mitosis gene a)-related kinase 5	Ssc.1742.1.S1_at	-1.32 q < 0.0000025	-1.29 q < 0.432	-1.46 q < 0.0373	-1.3 q < 0.211	-1.23 q < 1	-1.34 q < 0.255
SLC7A10	solute carrier family 7, (neutral amino acid transporter, y+ system) member 10	Ssc.21270.1.S1_at	-1.42 q < 0.00000251	-1.14 q < 1	-1.38 q < 0.327	-1.49 q < 0.0922	-2.1 q < 0.0000797	-1.16 q < 1
SF3B5	splicing factor 3b, subunit 5, 10kDa	Ssc.7328.1.S1_at	-1.31 q < 0.00000252	-1.12 q < 1	-1.41 q < 0.0607	-1.47 q < 0.017	-1.19 q < 1	-1.37 q < 0.131
C1R	complement component 1, r subcomponent	Ssc.983.1.S1_at	-1.74 q < 0.00000255	-1.45 q < 0.842	-1.82 q < 0.172	-1.76 q < 0.156	-1.55 q < 1	-2.23 q < 0.0243
PIP5K3	phosphoinositide kinase, FYVE finger containing	Ssc.7559.1.A1_at	-1.26 q < 0.00000258	-1.32 q < 0.125	-1.23 q < 0.381	-1.05 q < 0.834	-1.43 q < 0.0172	-1.31 q < 0.151
SLC15A3	solute carrier family 15, member 3	Ssc.2037.1.S1_at	1.37 q < 0.00000319	1.05 q < 1	1.31 q < 0.406	1.17 q < 0.595	1.28 q < 1	2.34 q < 0.0000000348
MYL6	myosin, light chain 6, alkali, smooth muscle and non-muscle	Ssc.10613.1.A1_at	-1.23 q < 0.00000325	-1.02 q < 1	-1.15 q < 0.625	-1.17 q < 0.344	-1.62 q < 0.00000798	-1.25 q < 0.24
MPP7	membrane protein, palmitoylated 7 (MAGUK p55 subfamily member 7)	Ssc.14264.1.A1_at	-1.51 q < 0.00000336	-1.37 q < 0.681	-1.53 q < 0.222	-1.73 q < 0.0365	-1.39 q < 1	-1.54 q < 0.259
MPP7	chromatin assembly factor 1, subunit B (p60)	Ssc.14264.1.A1_at	-1.51 q < 0.00000336	-1.37 q < 0.681	-1.53 q < 0.222	-1.73 q < 0.0365	-1.39 q < 1	-1.54 q < 0.259
PM20D2	peptidase M20 domain containing 2	Ssc.3479.1.A1_at	-1.3 q < 0.00000342	-1.29 q < 0.336	-1.08 q < 0.925	-1.28 q < 0.215	-1.51 q < 0.0142	-1.36 q < 0.136
PM20D2	peptidase M20 domain containing 2	Ssc.21928.1.A1_at	-1.33 q < 0.0000228	-1.32 q < 0.484	-1.26 q < 0.548	-1.29 q < 0.295	-1.5 q < 0.0992	-1.29 q < 0.653
CHRDL2	chordin-like 2	Ssc.26080.1.A1_at	-1.22 q < 0.00000343	-1.21 q < 0.376	-1.36 q < 0.0128	-1.12 q < 0.523	-1.28 q < 0.163	-1.14 q < 0.934
Q86T68	-	Ssc.5700.1.S1_at	-1.31 q < 0.00000349	-1.46 q < 0.0431	-1.25 q < 0.457	-1.34 q < 0.123	-1.23 q < 1	-1.28 q < 0.47
HOXB8	homeobox B8	Ssc.22848.1.S1_at	-1.24 q < 0.00000361	-1.13 q < 0.989	-1.57 q < 0.0000956	-1.12 q < 0.57	-1.12 q < 1	-1.31 q < 0.087
GPHN	gephyrin	Ssc.25182.1.A1_at	-1.26 q < 0.00000406	-1.38 q < 0.0439	-1.22 q < 0.425	-1.27 q < 0.146	-1.35 q < 0.103	-1.09 q < 1
TTC27	tetratricopeptide repeat domain 27	Ssc.27202.1.A1_at	1.2 q < 0.0000059	1.25 q < 0.148	1.14 q < 0.621	1.16 q < 0.326	1.32 q < 0.0379	1.15 q < 0.775
RBX1	ring-box 1	Ssc.18792.1.A1_at	1.22 q < 0.00000618	1.23 q < 0.31	1.12 q < 0.75	1.32 q < 0.0298	1.22 q < 0.621	1.22 q < 0.391
KTELC1	KTEL (Lys-Tyr-Glu-Leu) containing 1	Ssc.2008.1.A1_a_at	-1.26 q < 0.00000621	-1.3 q < 0.185	-1.39 q < 0.0346	-1.21 q < 0.321	-1.36 q < 0.106	-1.06 q < 1
KIAA0494	KIAA0494	Ssc.19401.2.A1_at	-1.3 q < 0.0000074	-1.38 q < 0.14	-1.46 q < 0.0353	-1.12 q < 0.646	-1.34 q < 0.37	-1.23 q < 0.786
KIAA0494	KIAA0494	Ssc.19401.1.S1_at	-1.26 q < 0.000529	-1.25 q < 0.693	-1.37 q < 0.19	-1.23 q < 0.417	-1.17 q < 1	-1.29 q < 0.565
PARD3B	par-3 partitioning defective 3 homolog B (C. elegans)	Ssc.15329.2.S1_at	-1.29 q < 0.00000817	-1.08 q < 1	-1.44 q < 0.0416	-1.2 q < 0.428	-1.4 q < 0.149	-1.4 q < 0.0899
PARD3B	par-3 partitioning defective 3 homolog B (C. elegans)	Ssc.11292.1.A1_at	1.16 q < 0.013	1.13 q < 1	1.09 q < 0.899	1.06 q < 0.805	1.21 q < 1	1.34 q < 0.131
SC5DL	sterol-C5-desaturase (ERG3 delta-5-desaturase homolog, S. cerevisiae)-like	Ssc.15727.1.A1_at	1.56 q < 0.00000842	1.49 q < 0.521	1.11 q < 0.94	2.46 q < 0.000325	1.44 q < 1	1.58 q < 0.36
MLL	myeloid/lymphoid or mixed-lineage leukemia (trithorax homolog, Drosophila)	Ssc.3659.1.S1_at	1.18 q < 0.00000897	1.33 q < 0.00567	1.1 q < 0.783	1.15 q < 0.325	1.15 q < 1	1.19 q < 0.325
MLL	myeloid/lymphoid or mixed-lineage leukemia (trithorax homolog, Drosophila)	Ssc.27953.1.S1_at	1.14 q < 0.00459	1.18 q < 0.515	1.1 q < 0.828	1.17 q < 0.317	1.11 q < 1	1.13 q < 1

Gene Name	Gene Description	Probe Set ID	Breed MS-WC	Day 25 MS-WC	Day 45 MS-WC	Day 65 MS-WC	Day 85 MS-WC	Day 105 MS-WC
KIAA0907	KIAA0907	Ssc.25140.1.A1_at	1.24 q < 0.00000924	1.1 q < 1	1.13 q < 0.791	1.47 q < 0.00271	1.26 q < 0.519	1.29 q < 0.18
KIAA0907	KIAA0907	Ssc.7902.1.A1_at	1.17 q < 0.000137	1.14 q < 0.807	1.2 q < 0.243	1.2 q < 0.165	1.18 q < 0.769	1.12 q < 1
SYNJ1	synaptojanin 1	Ssc.24586.1.S1_at	-1.27 q < 0.00000933	-1.15 q < 1	-1.28 q < 0.253	-1.21 q < 0.345	-1.31 q < 0.372	-1.41 q < 0.0422
MGC29506	-	Ssc.23658.1.S1_at	-1.19 q < 0.0000096	-1.04 q < 1	-1.09 q < 0.842	-1.06 q < 0.768	-1.01 q < 1	-2.01 q < 0
GTF3A	general transcription factor IIIA	Ssc.8694.1.A1_at	-1.2 q < 0.0000102	-1.17 q < 0.579	-1.23 q < 0.155	-1.06 q < 0.744	-1.31 q < 0.0421	-1.22 q < 0.249
RNF216	ring finger protein 216	Ssc.20827.1.S1_at	-1.19 q < 0.0000112	-1.17 q < 0.567	-1.36 q < 0.00539	-1.26 q < 0.0555	-1.24 q < 0.252	1.03 q < 1
G6PD	glucose-6-phosphate dehydrogenase	Ssc.2570.1.A1_at	1.24 q < 0.0000113	1.28 q < 0.247	1.2 q < 0.495	1.09 q < 0.692	1.48 q < 0.00528	1.2 q < 0.685
Q9HAZ8	-	Ssc.10308.1.A1_at	1.31 q < 0.0000116	1.48 q < 0.0441	1.13 q < 0.87	1.4 q < 0.0828	1.23 q < 1	1.34 q < 0.282
CHM	choroideremia (Rab escort protein 1)	Ssc.30885.1.A1_at	-1.21 q < 0.000012	-1.26 q < 0.142	-1.34 q < 0.0192	-1.12 q < 0.535	-1.16 q < 1	-1.16 q < 0.788
TAP1	transporter 1, ATP-binding cassette, sub-family B (MDR/TAP)	Ssc.27863.1.S1_at	-1.27 q < 0.0000122	-1.01 q < 1	-1.34 q < 0.143	-1.58 q < 0.00141	-1.12 q < 1	-1.41 q < 0.0537
HHATL	hedgehog acyltransferase-like	Ssc.29133.1.S1_at	1.18 q < 0.0000128	1.08 q < 1	1.24 q < 0.107	1.41 q < 0.000318	1.08 q < 1	1.14 q < 0.807
VIM	vimentin	Ssc.11131.1.S1_at	-1.55 q < 0.0000136	1.03 q < 1	-1.52 q < 0.357	-1.59 q < 0.175	-1.61 q < 0.477	-2.36 q < 0.00104
TMEM97	transmembrane protein 97	Ssc.13777.2.S1_at	1.34 q < 0.0000143	1.46 q < 0.113	1.15 q < 0.849	1.83 q < 0.000318	1.25 q < 1	1.11 q < 1
ZFPM2	zinc finger protein, multitype 2	Ssc.30847.1.A1_at	-1.24 q < 0.0000143	-1.8 q < 0.000000575	-1.04 q < 0.958	-1.16 q < 0.476	-1.16 q < 1	-1.18 q < 0.828
FAM82A1	family with sequence similarity 82, member A1	Ssc.7751.1.A1_at	1.21 q < 0.0000144	1.09 q < 1	1.19 q < 0.412	1.34 q < 0.0154	1.16 q < 1	1.26 q < 0.136
Q9BTR0	-	Ssc.16710.1.S1_at	-1.17 q < 0.0000146	-1.12 q < 0.832	-1.26 q < 0.0402	-1.16 q < 0.256	-1.16 q < 0.842	-1.17 q < 0.481
SHANK1	SH3 and multiple ankyrin repeat domains 1	Ssc.10878.1.A1_at	-1.22 q < 0.000015	-1.76 q < 0.0000000708	-1.19 q < 0.447	-1.09 q < 0.668	-1.03 q < 1	-1.15 q < 0.99
CCL15	chemokine (C-C motif) ligand 15	Ssc.9803.1.A1_at	-1.3 q < 0.000016	-1.15 q < 1	-1.19 q < 0.687	-1.06 q < 0.849	-1.37 q < 0.294	-1.84 q < 0.0000255
MRPS25	mitochondrial ribosomal protein S25	Ssc.19906.1.S1_at	1.23 q < 0.000016	1.31 q < 0.108	1.07 q < 0.913	1.48 q < 0.00148	1.2 q < 0.921	1.12 q < 1
OSBPL1A	oxysterol binding protein-like 1A	Ssc.24762.1.A1_at	-1.34 q < 0.000016	1.09 q < 1	-1.87 q < 0.000192	-1.37 q < 0.159	-1.31 q < 0.861	-1.38 q < 0.267
EBNA1BP2	EBNA1 binding protein 2	Ssc.26271.1.S1_at	1.2 q < 0.0000166	1.05 q < 1	1.19 q < 0.387	1.41 q < 0.00247	1.25 q < 0.288	1.15 q < 0.87
Q5VW16	-	Ssc.29093.1.S1_at	-1.21 q < 0.000017	-1.1 q < 1	-1.34 q < 0.0232	-1.19 q < 0.272	-1.17 q < 1	-1.26 q < 0.166
CXCL9	chemokine (C-X-C motif) ligand 9	Ssc.26146.1.S1_at	-1.32 q < 0.0000189	-1.02 q < 1	1.1 q < 0.913	1.03 q < 0.937	1 q < 1	-4.39 q < 0
GNAL	guanine nucleotide binding protein (G protein), alpha activating activity polypeptide, olfactory type	Ssc.23367.1.A1_at	-1.2 q < 0.0000203	-1.14 q < 0.824	-1.15 q < 0.597	-1.27 q < 0.0669	-1.35 q < 0.0232	-1.1 q < 1
TEP1	telomerase-associated protein 1	Ssc.11401.1.A1_a_at	-1.3 q < 0.0000203	-1.26 q < 0.567	-1.14 q < 0.843	-1.08 q < 0.787	-1.47 q < 0.0688	-1.61 q < 0.00428
TEP1	phosphatase and tensin homolog	Ssc.11401.1.A1_a_at	-1.3 q < 0.0000203	-1.26 q < 0.567	-1.14 q < 0.843	-1.08 q < 0.787	-1.47 q < 0.0688	-1.61 q < 0.00428
CRTAP	cartilage associated protein	Ssc.3508.1.A1_at	-1.17 q < 0.0000206	-1.27 q < 0.0381	-1.24 q < 0.0761	-1.06 q < 0.721	-1.09 q < 1	-1.21 q < 0.191
C1S	complement component 1, s subcomponent	Ssc.1177.1.S1_at	-1.56 q < 0.000021	-1.35 q < 0.928	-1.91 q < 0.0467	-1.71 q < 0.11	-1.37 q < 1	-1.55 q < 0.491
BCL2L14	BCL2-like 14 (apoptosis facilitator)	Ssc.30065.1.A1_at	-1.24 q < 0.0000212	-1.42 q < 0.0205	-1.28 q < 0.206	-1.25 q < 0.199	-1.26 q < 0.532	-1.03 q < 1
GSTM3	glutathione S-transferase mu 3 (brain)	Ssc.12273.1.A1_at	-1.35 q < 0.0000212	-1.84 q < 0.000906	-1.01 q < 0.997	-1.31 q < 0.294	-1.34 q < 0.804	-1.39 q < 0.315
TBL1X	transducin (beta)-like 1X-linked	Ssc.11444.1.S1_a_at	-1.32 q < 0.0000213	-1.15 q < 1	-1.38 q < 0.17	-1.16 q < 0.585	-1.37 q < 0.42	-1.56 q < 0.0165
TBL1X	transducin (beta)-like 1X-linked	Ssc.10389.1.A1_at	1.27 q < 0.02	1.49 q < 0.39	1.22 q < 0.834	1.51 q < 0.181	-1.15 q < 1	1.41 q < 0.634
FAM129C	family with sequence similarity 129, member C	Ssc.6341.3.A1_at	1.2 q < 0.0000244	1.07 q < 1	1.26 q < 0.112	1.41 q < 0.00177	1.12 q < 1	1.15 q < 0.866
MVK	mevalonate kinase	Ssc.3345.1.A1_at	1.25 q < 0.0000265	1.33 q < 0.136	-1.05 q < 0.951	1.35 q < 0.0608	1.26 q < 0.669	1.41 q < 0.0345
MVK	mevalonate kinase	Ssc.3345.2.S1_at	1.15 q < 0.0172	1.24 q < 0.496	-1 q < 0.997	1.25 q < 0.226	1.13 q < 1	1.18 q < 0.926
CLN3	ceroid-lipofuscinosis, neuronal 3	Ssc.19602.1.A1_at	-1.19 q < 0.00003	-1.12 q < 0.918	-1.3 q < 0.0311	-1.1 q < 0.571	-1.23 q < 0.348	-1.18 q < 0.489
CLN3	ceroid-lipofuscinosis, neuronal 3	Ssc.19602.2.S1_at	-1.23 q < 0.000137	-1.21 q < 0.7	-1.34 q < 0.118	-1.13 q < 0.574	-1.23 q < 0.951	-1.27 q < 0.41
ARL15	ADP-ribosylation factor-like 15	Ssc.8613.1.A1_at	1.24 q < 0.0000304	-1.01 q < 1	1.26 q < 0.287	1.16 q < 0.482	1.26 q < 0.59	1.62 q < 0.00017
ARL15	ADP-ribosylation factor-like 15	Ssc.14168.1.A1_at	1.11 q < 0.0265	-1.01 q < 1	1.11 q < 0.738	1.07 q < 0.68	1.17 q < 0.842	1.2 q < 0.328
PLXNA2	plexin A2	Ssc.28314.1.S1_at	1.22 q < 0.0000308	1.13 q < 1	1.19 q < 0.513	1.35 q < 0.031	1.21 q < 0.892	1.24 q < 0.374
IL34	interleukin 34	Ssc.19637.1.S1_at	1.2 q < 0.0000354	-1.12 q < 1	1.22 q < 0.301	1.52 q < 0.000137	1.25 q < 0.406	1.24 q < 0.291
C1QC	complement component 1, q subcomponent, C chain	Ssc.17316.1.S1_at	-1.79 q < 0.0000359	-1.06 q < 1	-1.57 q < 0.601	-1.41 q < 0.552	1 q < 1	-7.76 q < 0.0000000000271
CAPG	capping protein (actin filament), gelsolin-like	Ssc.18360.1.S1_at	-1.27 q < 0.000036	-1.03 q < 1	-1.28 q < 0.34	-1.99 q < 0.000000382	1.05 q < 1	-1.35 q < 0.189
RPL7A	ribosomal protein L7a	Ssc.13304.1.A1_at	-1.17 q < 0.0000366	-1.03 q < 1	-1.19 q < 0.253	-1.22 q < 0.0908	-1.07 q < 1	-1.36 q < 0.00223
NCK1	NCK adaptor protein 1	Ssc.4565.1.A1_at	1.46 q < 0.0000385	1.62 q < 0.168	1.14 q < 0.913	1.9 q < 0.0116	1.5 q < 0.622	1.27 q < 1
NCK1	NCK adaptor protein 1	Ssc.10338.1.A1_at	-1.17 q < 0.0204	-1.14 q < 1	-1.24 q < 0.459	-1.17 q < 0.496	-1.15 q < 1	-1.13 q < 1

Gene Name	Gene Description	Probe Set ID	Breed MS-WC	Day 25 MS-WC	Day 45 MS-WC	Day 65 MS-WC	Day 85 MS-WC	Day 105 MS-WC
MTSS1	metastasis suppressor 1	Ssc.7893.1.A1_at	-1.43 q < 0.0000396	-1.51 q < 0.286	-1.73 q < 0.0395	-1.03 q < 0.947	-1.38 q < 1	-1.63 q < 0.115
MTSS1	metastasis suppressor 1	Ssc.12805.1.A1_at	1.08 q < 0.0523	-1.04 q < 1	1.07 q < 0.844	1.22 q < 0.0393	-1.02 q < 1	1.2 q < 0.112
FAM62B	extended synaptotagmin-like protein 2	Ssc.1817.1.S1_at	-1.35 q < 0.00004	-1.36 q < 0.416	-1.68 q < 0.0115	-1.12 q < 0.718	-1.46 q < 0.272	-1.18 q < 1
FAM62B	extended synaptotagmin-like protein 2	Ssc.9284.1.S1_at	-1.2 q < 0.0408	-1.16 q < 1	-1.3 q < 0.521	-1 q < 1	-1.25 q < 1	-1.32 q < 0.676
TRIM16	tripartite motif-containing 16	Ssc.12463.1.A1_at	-1.31 q < 0.0000443	-1.36 q < 0.322	-1.19 q < 0.747	-1.36 q < 0.165	-1.17 q < 1	-1.52 q < 0.0448
LRBA	LPS-responsive vesicle trafficking, beach and anchor containing	Ssc.3206.1.S1_at	-1.23 q < 0.000045	-1.08 q < 1	-1.51 q < 0.00151	-1.1 q < 0.65	-1.33 q < 0.155	-1.17 q < 0.926
PBRM1	polybromo 1	Ssc.5809.1.A1_at	-1.22 q < 0.0000452	-1.18 q < 0.739	-1.45 q < 0.0067	-1.03 q < 0.903	-1.28 q < 0.348	-1.22 q < 0.562
KIF13A	kinesin family member 13A	Ssc.26196.1.A1_at	-1.22 q < 0.0000483	-1.29 q < 0.153	-1.29 q < 0.125	1.06 q < 0.782	-1.38 q < 0.0376	-1.23 q < 0.433
RORC	RAR-related orphan receptor C	Ssc.9081.1.S1_at	-1.25 q < 0.0000483	-1.05 q < 1	-1.33 q < 0.135	-1.26 q < 0.222	-1.22 q < 1	-1.42 q < 0.033
TFRC	transferrin receptor (p90, CD71)	Ssc.3753.1.S1_at	1.63 q < 0.000049	2.08 q < 0.0597	1.3 q < 0.837	2.3 q < 0.0116	1.28 q < 1	1.45 q < 0.931
C12orf42	chromosome 12 open reading frame 42	Ssc.31169.1.S1_at	-1.21 q < 0.0000495	-1.27 q < 0.195	-1.44 q < 0.00406	-1.08 q < 0.693	-1.34 q < 0.0653	1.03 q < 1
ABI3BP	ABI family, member 3 (NESH) binding protein	Ssc.11207.1.A1_at	-1.47 q < 0.0000495	-3.13 q < 0.000000139	-1.26 q < 0.787	-1.25 q < 0.566	-1.08 q < 1	-1.28 q < 1
SP100	SP100 nuclear antigen	Ssc.7207.2.A1_at	1.22 q < 0.0000546	1.04 q < 1	1.26 q < 0.216	1.36 q < 0.0249	1.34 q < 0.103	1.12 q < 1
SP100	SP100 nuclear antigen	Ssc.26520.1.A1_at	-1.2 q < 0.00105	-1.28 q < 0.34	-1.14 q < 0.799	-1.21 q < 0.346	-1.26 q < 0.682	-1.14 q < 1
PDXK	pyridoxal (pyridoxine, vitamin B6) kinase	Ssc.11170.1.S1_at	-1.24 q < 0.0000568	1.11 q < 1	-1.55 q < 0.00131	-1.19 q < 0.398	-1.26 q < 0.607	-1.37 q < 0.0572
UBE3A	ubiquitin protein ligase E3A	Ssc.29211.1.A1_at	1.19 q < 0.0000578	1.05 q < 1	1.02 q < 0.982	1.41 q < 0.00215	1.25 q < 0.261	1.27 q < 0.122
PLEKHA3	pleckstrin homology domain containing, family A (phosphoinositide binding specific) member 3	Ssc.25084.1.S1_at	-1.2 q < 0.0000598	-1.13 q < 0.962	-1.3 q < 0.0676	-1.08 q < 0.708	-1.38 q < 0.0167	-1.13 q < 1
FDFT1	farnesyl-diphosphate farnesyltransferase 1	Ssc.6418.1.S1_at	1.3 q < 0.0000606	1.72 q < 0.00127	-1.09 q < 0.925	1.34 q < 0.188	1.37 q < 0.42	1.28 q < 0.643
CSNK1E	casein kinase 1, epsilon	Ssc.28471.2.S1_at	-1.17 q < 0.0000616	-1.07 q < 1	-1.15 q < 0.508	-1.14 q < 0.354	-1.29 q < 0.0409	-1.19 q < 0.351
CD46	CD46 molecule, complement regulatory protein	Ssc.14518.1.S1_at	1.19 q < 0.000062	1.02 q < 1	1.16 q < 0.579	1.18 q < 0.317	1.44 q < 0.00235	1.22 q < 0.374
PARVB	parvin, beta	Ssc.21537.1.A1_at	-1.32 q < 0.00007	-1.05 q < 1	-1.92 q < 0.000176	-1.16 q < 0.61	-1.51 q < 0.115	-1.14 q < 1
BPHL	biphenyl hydrolase-like (serine hydrolase)	Ssc.2968.1.S1_at	-1.55 q < 0.0000724	-1.49 q < 0.642	-2.19 q < 0.0115	-1.19 q < 0.718	-1.47 q < 1	-1.58 q < 0.493
VGLL4	vestigial like 4 (Drosophila)	Ssc.6410.3.S1_at	1.35 q < 0.0000724	1.44 q < 0.244	1.05 q < 0.968	1.57 q < 0.0378	1.28 q < 1	1.45 q < 0.211
YIPF1	Yip1 domain family, member 1	Ssc.1236.1.S1_at	-1.22 q < 0.0000735	-1.15 q < 0.921	-1.47 q < 0.00454	-1.04 q < 0.877	-1.17 q < 1	-1.33 q < 0.102
ETV6	ets variant 6	Ssc.22398.1.A1_at	-1.43 q < 0.0000746	-1.93 q < 0.00875	-1.34 q < 0.587	-1.43 q < 0.265	-1.16 q < 1	-1.4 q < 0.671
ETV6	ets variant 6	Ssc.8148.1.S1_at	-1.15 q < 0.0392	-1.23 q < 0.619	-1.34 q < 0.148	-1.11 q < 0.664	-1.04 q < 1	-1.06 q < 1
OSGIN1	oxidative stress induced growth inhibitor 1	Ssc.26696.1.S1_at	1.25 q < 0.0000781	-1.01 q < 1	1.21 q < 0.559	1.67 q < 0.000213	1.27 q < 0.694	1.21 q < 0.815
BBS2	Bardet-Biedl syndrome 2	Ssc.4843.1.A1_at	-1.24 q < 0.0000792	-1.66 q < 0.000155	-1.17 q < 0.672	-1.19 q < 0.4	-1.17 q < 1	-1.08 q < 1
TCEAL3	transcription elongation factor A (SII)-like 3	Ssc.21736.1.S1_at	1.19 q < 0.0000804	1.07 q < 1	1.07 q < 0.913	1.6 q < 0.00000841	1.23 q < 0.513	1.08 q < 1
IMP1	histocompatibility (minor) 13	Ssc.17159.1.S1_at	-1.31 q < 0.000083	-1.03 q < 1	-1.12 q < 0.901	-1.17 q < 0.594	-1.19 q < 1	-2.45 q < 0.0000000056
AEBP1	AE binding protein 1	Ssc.16584.1.A1_at	-1.63 q < 0.0000845	-1.32 q < 1	-2.33 q < 0.0165	-1.57 q < 0.317	-1.26 q < 1	-1.86 q < 0.197
AEBP1	AE binding protein 1	Ssc.11603.1.S1_at	-1.25 q < 0.074	1.04 q < 1	-1.9 q < 0.0285	-1.18 q < 0.695	-1.11 q < 1	-1.25 q < 1
MVD	mevalonate (diphospho) decarboxylase	Ssc.4685.1.S1_at	1.3 q < 0.0000894	1.27 q < 0.671	-1.11 q < 0.91	1.41 q < 0.105	1.53 q < 0.0587	1.51 q < 0.049
DACT3	dapper, antagonist of beta-catenin, homolog 3 (Xenopus laevis)	Ssc.3327.1.A1_at	-1.16 q < 0.0000923	-1.25 q < 0.0714	-1.28 q < 0.0227	-1.21 q < 0.102	-1.08 q < 1	1.01 q < 1
TM2D2	TM2 domain containing 2	Ssc.13904.1.S1_at	-1.24 q < 0.0000945	-1.12 q < 1	-1.5 q < 0.00558	-1.11 q < 0.662	-1.29 q < 0.467	-1.2 q < 0.785
C1QB	complement component 1, q subcomponent, B chain	Ssc.11004.1.S1_at	-1.8 q < 0.0000978	-1.25 q < 1	-1.53 q < 0.702	-1.38 q < 0.611	-1.04 q < 1	-6.94 q < 0.0000000066
SLC44A5	solute carrier family 44, member 5	Ssc.24894.1.S1_at	1.21 q < 0.0000993	1.05 q < 1	1.18 q < 0.548	1.43 q < 0.00535	1.23 q < 0.714	1.19 q < 0.714
RSBN1L	round spermatid basic protein 1-like	Ssc.13280.1.A1_at	1.26 q < 0.000103	1.05 q < 1	1.11 q < 0.881	1.56 q < 0.00351	1.34 q < 0.346	1.27 q < 0.47
RSBN1L	round spermatid basic protein 1-like	Ssc.10678.1.A1_at	1.09 q < 0.211	1.01 q < 1	-1.02 q < 0.982	1.33 q < 0.0416	1.1 q < 1	1.06 q < 1
TMOD3	tropomodulin 3 (ubiquitous)	Ssc.16363.1.S2_at	-1.4 q < 0.000103	-1.51 q < 0.262	-1.77 q < 0.0218	-1.54 q < 0.111	-1.24 q < 1	-1.05 q < 1
TMOD3	tropomodulin 3 (ubiquitous)	Ssc.16363.1.S1_at	-1.49 q < 0.00000188	-1.35 q < 0.671	-1.61 q < 0.0932	-1.5 q < 0.147	-1.59 q < 0.215	-1.4 q < 0.56
UBE2V1	ubiquitin-conjugating enzyme E2 variant 1	Ssc.5681.1.S1_at	-1.25 q < 0.000103	-1.06 q < 1	-1.55 q < 0.00344	-1.11 q < 0.657	-1.36 q < 0.196	-1.2 q < 0.852
UBE2V1	ubiquitin-conjugating enzyme E2 variant 1	Ssc.19134.2.S1_at	-1.16 q < 0.000574	-1.12 q < 0.955	-1.26 q < 0.0796	-1.21 q < 0.165	-1.06 q < 1	-1.14 q < 0.867
SAP18	Sin3A-associated protein, 18kDa	Ssc.21860.2.S1_at	-1.12 q < 0.000105	1.01 q < 1	-1.07 q < 0.808	-1.4 q < 0.000000925	-1.09 q < 1	-1.1 q < 0.893
TSPAN32	tetraspanin 32	Ssc.1595.1.S1_a_at	1.36 q < 0.000105	1.83 q < 0.00542	1.32 q < 0.533	1.24 q < 0.503	1.01 q < 1	1.57 q < 0.091
EMP3	epithelial membrane protein 3	Ssc.11016.1.S1_at	-1.35 q < 0.000109	-1.05 q < 1	-1.39 q < 0.322	-1.22 q < 0.524	-1.42 q < 0.554	-1.78 q < 0.00637

Gene Name	Gene Description	Probe Set ID	Breed MS-WC	Day 25 MS-WC	Day 45 MS-WC	Day 65 MS-WC	Day 85 MS-WC	Day 105 MS-WC
AKAP3	A kinase (PRKA) anchor protein 3	Ssc.21834.1.S1_a_at	1.2 q < 0.000126	1.1 q < 1	1.28 q < 0.132	1.38 q < 0.0144	1.16 q < 1	1.11 q < 1
LARS	leucyl-tRNA synthetase	Ssc.1127.1.A1_at	1.26 q < 0.000126	1.55 q < 0.00644	1.25 q < 0.457	1.3 q < 0.186	1.14 q < 1	1.09 q < 1
SLC47A1	solute carrier family 47, member 1	Ssc.27371.1.S1_at	1.48 q < 0.00014	1.27 q < 1	1.39 q < 0.588	2.47 q < 0.000304	1.15 q < 1	1.42 q < 0.781
PPFIBP1	PTPRF interacting protein, binding protein 1 (liprin beta 1)	Ssc.9449.1.A1_at	-1.31 q < 0.000143	-1.52 q < 0.077	-1.74 q < 0.00359	-1.19 q < 0.547	-1.26 q < 1	1.01 q < 1
PPFIBP1	PTPRF interacting protein, binding protein 1 (liprin beta 1)	Ssc.11536.1.S1_at	1.11 q < 0.0315	-1.05 q < 1	1.01 q < 0.989	1.16 q < 0.301	1.1 q < 1	1.34 q < 0.0102
Q8N675	-	Ssc.1609.1.A1_at	-1.35 q < 0.000144	-1.04 q < 1	-1.26 q < 0.669	-1.97 q < 0.00052	-1.41 q < 0.617	-1.24 q < 1
DCN	decorin	Ssc.10245.2.A1_a_at	-1.54 q < 0.00015	-1.39 q < 0.893	-1.52 q < 0.47	-1.53 q < 0.308	-1.17 q < 1	-2.32 q < 0.00659
DCN	decorin	Ssc.12150.1.A1_at	-1.13 q < 0.0152	-1.35 q < 0.0267	-1.11 q < 0.799	-1.02 q < 0.917	-1.02 q < 1	-1.18 q < 0.711
SOCS5	suppressor of cytokine signaling 5	Ssc.26942.1.S1_at	-1.16 q < 0.000155	-1.14 q < 0.789	-1.35 q < 0.00393	-1.11 q < 0.524	-1.13 q < 1	-1.1 q < 1
MAZ	MYC-associated zinc finger protein (purine-binding transcription factor)	Ssc.12113.2.S1_at	-1.17 q < 0.000158	-1.08 q < 1	-1.38 q < 0.00379	-1.06 q < 0.771	-1.22 q < 0.454	-1.16 q < 0.768
LYZ	lysozyme	Ssc.670.1.S1_at	1.67 q < 0.000163	1.02 q < 1	1.59 q < 0.529	-1.17 q < 0.797	2.28 q < 0.0806	4.07 q < 0.00000906
LYZ	lysozyme	Ssc.670.2.S1_at	1.1 q < 0.154	1.03 q < 1	-1.08 q < 0.902	1.01 q < 0.957	1.19 q < 1	1.4 q < 0.0159
PRPSAP1	phosphoribosyl pyrophosphate synthetase-associated protein 1	Ssc.21552.2.S1_at	-1.23 q < 0.000166	-1.07 q < 1	-1.29 q < 0.249	-1.22 q < 0.34	-1.55 q < 0.00387	-1.1 q < 1
HDAC4	histone deacetylase 4	Ssc.15312.1.S1_at	-1.19 q < 0.00017	-1.4 q < 0.00901	-1.17 q < 0.533	-1.12 q < 0.536	-1.13 q < 1	-1.15 q < 0.937
PQLC3	PQ loop repeat containing 3	Ssc.16722.1.S1_at	-1.5 q < 0.00018	-1.26 q < 1	-2.09 q < 0.0168	-1.27 q < 0.595	-1.46 q < 1	-1.58 q < 0.445
PARP4	poly (ADP-ribose) polymerase family, member 4	Ssc.17953.1.S1_at	-1.17 q < 0.000181	-1.08 q < 1	-1.14 q < 0.603	-1.12 q < 0.51	-1.22 q < 0.444	-1.31 q < 0.0322
HPGD	hydroxyprostaglandin dehydrogenase 15-(NAD)	Ssc.15266.1.S1_at	2.25 q < 0.000182	1.36 q < 1	3.52 q < 0.0646	4.76 q < 0.00617	1.48 q < 1	1.71 q < 1
HPGD	hydroxyprostaglandin dehydrogenase 15-(NAD)	Ssc.15266.2.S1_at	1.15 q < 0.05	-1.04 q < 1	1.22 q < 0.556	1.43 q < 0.0408	1.17 q < 1	1.03 q < 1
ZNF354A	zinc finger protein 354A	Ssc.27217.2.A1_at	1.25 q < 0.000184	1.16 q < 1	1.19 q < 0.645	1.47 q < 0.0172	1.22 q < 1	1.22 q < 0.793
ZNF354A	zinc finger protein 354A	Ssc.27217.1.A1_at	1.12 q < 0.00822	1.03 q < 1	1.17 q < 0.366	1.23 q < 0.0806	1.06 q < 1	1.11 q < 1
C10orf76	chromosome 10 open reading frame 76	Ssc.10285.1.A1_at	-1.38 q < 0.00019	-1.09 q < 1	-1.98 q < 0.00217	-1.07 q < 0.861	-1.48 q < 0.496	-1.45 q < 0.397
SLC1A3	solute carrier family 1 (glial high affinity glutamate transporter), member 3	Ssc.26957.1.S1_at	1.33 q < 0.000203	1.07 q < 1	1.25 q < 0.663	1.89 q < 0.00105	1.36 q < 0.823	1.22 q < 1
SLC1A3	solute carrier family 1 (glial high affinity glutamate transporter), member 3	Ssc.8447.1.A1_at	1.12 q < 0.497	-1.03 q < 1	-1.19 q < 0.858	1.69 q < 0.0448	1.12 q < 1	1.15 q < 1
CCDC142	coiled-coil domain containing 142	Ssc.15679.1.A1_at	1.27 q < 0.000208	1.26 q < 0.652	1.04 q < 0.973	1.43 q < 0.0631	1.52 q < 0.0447	1.17 q < 1
THYN1	thymocyte nuclear protein 1	Ssc.12072.1.S1_a_at	-1.52 q < 0.00021	-1.13 q < 1	-2.09 q < 0.0237	-1.68 q < 0.159	-1.4 q < 1	-1.47 q < 0.775
HSF2BP	heat shock transcription factor 2 binding protein	Ssc.18482.1.S1_at	-1.2 q < 0.000221	-1.11 q < 1	-1.35 q < 0.0402	-1.1 q < 0.636	-1.18 q < 1	-1.28 q < 0.197
CCDC84	coiled-coil domain containing 84	Ssc.6964.1.A1_at	1.18 q < 0.00023	1.25 q < 0.197	1.04 q < 0.946	1.3 q < 0.044	1.24 q < 0.393	1.09 q < 1
IL18BP	interleukin 18 binding protein	Ssc.16985.1.S1_at	-1.19 q < 0.000234	-1.11 q < 1	-1.16 q < 0.57	-1.14 q < 0.484	-1.43 q < 0.00528	-1.12 q < 1
PTGS1	prostaglandin-endoperoxide synthase 1 (prostaglandin G/H synthase and cyclooxygenase)	Ssc.1986.1.S1_at	-1.42 q < 0.000235	-1.27 q < 1	-1.41 q < 0.488	-1.62 q < 0.109	-1.14 q < 1	-1.79 q < 0.0494
FBN1	fibrillin 1	Ssc.27981.1.A1_at	1.24 q < 0.000241	-1.05 q < 1	1.29 q < 0.263	1.43 q < 0.0316	1.31 q < 0.452	1.27 q < 0.49
SH3GL3	SH3-domain GRB2-like 3	Ssc.5165.1.S1_at	1.3 q < 0.000247	1.04 q < 1	1.41 q < 0.177	1.51 q < 0.041	1.34 q < 0.724	1.23 q < 0.967
HBZ	hemoglobin, zeta	Ssc.15708.1.S1_at	1.21 q < 0.000256	1.71 q < 0.0000227	1.2 q < 0.493	1.04 q < 0.862	1.07 q < 1	1.12 q < 1
HBZ	hemoglobin, zeta	Ssc.21870.2.S1_a_at	-1.02 q < 0.959	1.42 q < 0.123	-1.56 q < 0.0147	-1.08 q < 0.796	1 q < 1	1.09 q < 1
ACBD3	acyl-CoA binding domain containing 3	Ssc.9039.2.S1_at	-1.3 q < 0.000263	-1.55 q < 0.0456	-1.45 q < 0.126	-1.28 q < 0.349	-1.3 q < 1	1.02 q < 1
GRAMD3	GRAM domain containing 3	Ssc.5080.1.A1_at	-1.39 q < 0.000274	-1.02 q < 1	-2.04 q < 0.00282	-1.19 q < 0.65	-1.6 q < 0.256	-1.33 q < 0.893
GRAMD3	GRAM domain containing 3	Ssc.26356.1.S1_at	-1.23 q < 0.00126	-1.03 q < 1	-1.63 q < 0.00344	-1.21 q < 0.444	-1.3 q < 0.734	-1.1 q < 1
IDS	iduronate 2-sulfatase	Ssc.16646.1.S1_at	-1.23 q < 0.000274	-1.13 q < 1	-1.4 q < 0.0473	-1.14 q < 0.557	-1.22 q < 1	-1.27 q < 0.444
RPL15	ribosomal protein L15	Ssc.12364.1.S1_at	-1.19 q < 0.000279	-1.05 q < 1	-1.34 q < 0.0414	-1.21 q < 0.262	-1.1 q < 1	-1.28 q < 0.153
LPAR1	lysophosphatidic acid receptor 1	Ssc.31062.1.S1_at	-1.21 q < 0.000282	-1.04 q < 1	-1.23 q < 0.389	-1.37 q < 0.0307	-1.15 q < 1	-1.27 q < 0.3
TYMS	thymidylate synthetase	Ssc.6934.2.A1_at	-1.17 q < 0.000283	-1.18 q < 0.491	-1.11 q < 0.766	-1.08 q < 0.662	-1.16 q < 1	-1.32 q < 0.0281
TYMS	thymidylate synthetase	Ssc.6934.3.S1_at	1.08 q < 0.219	1.38 q < 0.0149	1.05 q < 0.943	1.1 q < 0.607	1.04 q < 1	-1.1 q < 1
NUP210	nucleoporin 210kDa	Ssc.28954.1.S1_at	1.23 q < 0.000291	1.24 q < 0.523	1.14 q < 0.808	1.2 q < 0.379	1.13 q < 1	1.44 q < 0.024
IGF2R	insulin-like growth factor 2 receptor	Ssc.422.1.S1_at	-1.29 q < 0.000294	-1.03 q < 1	-1.49 q < 0.0725	-1.56 q < 0.0218	-1.27 q < 1	-1.16 q < 1
IGF2R	insulin-like growth factor 2 receptor	Ssc.15818.1.S1_at	-1.24 q < 0.000529	-1.07 q < 1	-1.46 q < 0.0387	-1.3 q < 0.212	-1.11 q < 1	-1.31 q < 0.37
Q9NX95	-	Ssc.4505.1.A1_at	1.26 q < 0.000294	1.02 q < 1	-1.15 q < 0.827	1.4 q < 0.0747	1.77 q < 0.000259	1.42 q < 0.0983

Gene Name	Gene Description	Probe Set ID	Breed MS-WC	Day 25 MS-WC	Day 45 MS-WC	Day 65 MS-WC	Day 85 MS-WC	Day 105 MS-WC
RBPJ	recombination signal binding protein for immunoglobulin kappa J region	Ssc.29086.1.S1_at	-1.21 q < 0.000298	-1 q < 1	-1.27 q < 0.241	-1.18 q < 0.404	-1.19 q < 1	-1.46 q < 0.00861
TIAM2	T-cell lymphoma invasion and metastasis 2	Ssc.30458.1.A1_at	1.18 q < 0.000299	1.07 q < 1	1.37 q < 0.0115	1.2 q < 0.233	1.04 q < 1	1.24 q < 0.271
DOCK11	dedicator of cytokinesis 11	Ssc.3303.1.S1_at	-1.45 q < 0.000302	-1.49 q < 0.515	-1.9 q < 0.0344	-1.22 q < 0.645	-1.29 q < 1	-1.44 q < 0.695
NPNT	nephronectin	Ssc.31071.1.A1_at	1.29 q < 0.000305	1.48 q < 0.104	1.11 q < 0.913	1.91 q < 0.000137	1.17 q < 1	-1.01 q < 1
NPNT	nephronectin	Ssc.9527.1.S1_at	1.11 q < 0.22	1.2 q < 0.878	-1.04 q < 0.973	1.49 q < 0.0175	1.06 q < 1	-1.07 q < 1
TRPV6	transient receptor potential cation channel, subfamily V, member 6	Ssc.2896.1.S1_at	1.66 q < 0.000313	1.55 q < 0.795	1.56 q < 0.594	2.57 q < 0.0116	1.2 q < 1	1.66 q < 0.666
STEAP4	STEAP family member 4	Ssc.9114.1.S1_at	1.44 q < 0.000314	1.08 q < 1	1.29 q < 0.764	2.29 q < 0.000992	1.43 q < 1	1.37 q < 0.903
UHRF1BP1L	UHRF1 binding protein 1-like	Ssc.7768.1.A1_at	-1.29 q < 0.000316	-1.6 q < 0.0228	-1.35 q < 0.318	-1.18 q < 0.562	-1.16 q < 1	-1.22 q < 1
CTTNBP2	cortactin binding protein 2	Ssc.26418.1.A1_at	1.16 q < 0.000324	1.14 q < 0.787	1.13 q < 0.628	1.3 q < 0.0172	1.06 q < 1	1.17 q < 0.562
LYCAT	lysocardiolipin acyltransferase 1	Ssc.7504.1.A1_at	1.37 q < 0.000328	1.03 q < 1	2.36 q < 0.0000357	1.19 q < 0.626	1.01 q < 1	1.66 q < 0.0727
CGNL1	cingulin-like 1	Ssc.1447.1.A1_at	-1.24 q < 0.000346	1.06 q < 1	-1.4 q < 0.0676	-1.45 q < 0.0246	-1.34 q < 0.348	-1.13 q < 1
CGNL1	cingulin-like 1	Ssc.1447.3.S1_at	-1.25 q < 0.000418	-1.02 q < 1	-1.39 q < 0.129	-1.31 q < 0.199	-1.33 q < 0.519	-1.25 q < 0.713
SMAD5	SMAD family member 5	Ssc.7262.1.A1_at	-1.26 q < 0.000359	-1.34 q < 0.304	-1.54 q < 0.017	1.03 q < 0.938	-1.24 q < 1	-1.27 q < 0.652
SMAD5	SMAD family member 5	Ssc.5634.1.A1_at	1.23 q < 0.00869	1.38 q < 0.336	1.18 q < 0.816	1.08 q < 0.822	1.14 q < 1	1.41 q < 0.275
NP_922932	-	Ssc.30245.1.A1_s_at	-1.18 q < 0.000363	-1.61 q < 0.00000531	-1.02 q < 0.988	-1.14 q < 0.455	-1.08 q < 1	-1.11 q < 1
NP_922932	-	Ssc.22377.1.A1_at	-1.13 q < 0.0371	-1.5 q < 0.00171	1 q < 1	-1.05 q < 0.844	-1.1 q < 1	-1.06 q < 1
WDR42A	DDB1 and CUL4 associated factor 8	Ssc.19651.1.S1_at	-1.19 q < 0.000363	-1.05 q < 1	-1.54 q < 0.000289	-1.15 q < 0.468	-1.19 q < 1	-1.09 q < 1
RWDD4A	RWD domain containing 4A	Ssc.2855.1.S1_at	1.2 q < 0.000364	1.1 q < 1	1.25 q < 0.31	1.37 q < 0.034	1.27 q < 0.463	1.07 q < 1
MTHFR	methylenetetrahydrofolate reductase (NAD(P)H)	Ssc.2260.1.A1_at	1.19 q < 0.000377	1.08 q < 1	1.01 q < 0.997	1.38 q < 0.0115	1.26 q < 0.331	1.23 q < 0.351
FDPS	farnesyl diphosphate synthase	Ssc.12202.2.S1_at	1.31 q < 0.00038	1.45 q < 0.187	1.04 q < 0.979	1.7 q < 0.0067	1.26 q < 1	1.17 q < 1
FAM32A	family with sequence similarity 32, member A	Ssc.24189.1.S1_at	1.14 q < 0.000394	1.34 q < 0.0025	1.06 q < 0.908	1.1 q < 0.523	1.18 q < 0.561	1.06 q < 1
THBS3	thrombospondin 3	Ssc.11559.2.A1_at	-1.31 q < 0.000418	1.2 q < 1	-1.28 q < 0.556	-1.36 q < 0.235	-1.5 q < 0.193	-1.75 q < 0.00489
THBS3	thrombospondin 3	Ssc.11559.1.A1_at	-1.23 q < 0.0133	1.17 q < 1	-1.28 q < 0.566	-1.14 q < 0.679	-1.32 q < 0.988	-1.68 q < 0.0116
DYNC1LI2	dynein, cytoplasmic 1, light intermediate chain 2	Ssc.25763.1.A1_at	-1.18 q < 0.000428	-1.16 q < 0.807	-1.07 q < 0.916	-1.17 q < 0.381	-1.41 q < 0.0098	-1.13 q < 1
LYPLAL1	lysophospholipase-like 1	Ssc.19905.1.S1_at	1.19 q < 0.000436	1.16 q < 0.809	1.13 q < 0.749	1.38 q < 0.0161	1.17 q < 1	1.12 q < 1
UGCG	UDP-glucose ceramide glucosyltransferase	Ssc.13473.1.A1_at	1.15 q < 0.00044	-1.06 q < 1	1.49 q < 0.0000163	1.13 q < 0.398	1.04 q < 1	1.2 q < 0.253
TOM1L2	target of myb1-like 2 (chicken)	Ssc.5014.1.S1_at	-1.19 q < 0.000443	-1.18 q < 0.712	-1.84 q < 0.0000000482	-1.14 q < 0.508	-1.05 q < 1	1.07 q < 1
TOM1L2	target of myb1-like 2 (chicken)	Ssc.11264.1.A1_at	-1.13 q < 0.00973	-1.19 q < 0.527	-1.09 q < 0.864	-1.03 q < 0.907	-1.17 q < 1	-1.21 q < 0.397
MEP1B	meprin A, beta	Ssc.12240.1.S1_at	1.54 q < 0.000459	1.16 q < 1	1.25 q < 0.877	2.43 q < 0.00514	1.66 q < 0.694	1.47 q < 0.869
MEP1B	meprin A, beta	Ssc.13230.1.S1_at	1.16 q < 0.0522	-1.01 q < 1	1.07 q < 0.94	1.48 q < 0.0251	1.15 q < 1	1.15 q < 1
CCDC99	coiled-coil domain containing 99	Ssc.25461.1.S1_at	1.19 q < 0.000462	1.04 q < 1	1.24 q < 0.239	1.77 q < 0.000000145	-1 q < 1	1.03 q < 1
CCDC99	coiled-coil domain containing 99	Ssc.22545.1.A1_at	1.05 q < 0.577	1.43 q < 0.000735	1.01 q < 0.996	-1.01 q < 0.966	-1.03 q < 1	-1.08 q < 1
SULT4A1	sulfotransferase family 4A, member 1	Ssc.1936.1.A1_at	-1.23 q < 0.000462	-1.02 q < 1	-1.29 q < 0.276	-1.22 q < 0.354	-1.46 q < 0.0437	-1.19 q < 0.934
MYEOV2	myeloma overexpressed 2	Ssc.16493.1.S1_s_at	-1.14 q < 0.000467	1.1 q < 0.987	-1.29 q < 0.0125	-1.2 q < 0.126	-1.15 q < 0.997	-1.19 q < 0.242
GNRHR2	gonadotropin-releasing hormone (type 2) receptor 2	Ssc.26339.1.S1_a_at	1.18 q < 0.000481	1.08 q < 1	1.15 q < 0.647	1.4 q < 0.00413	1.14 q < 1	1.13 q < 1
PDCD6IP	programmed cell death 6 interacting protein	Ssc.17478.1.A1_at	-1.23 q < 0.000488	-1.1 q < 1	-1.28 q < 0.316	-1.48 q < 0.0134	-1.25 q < 0.917	-1.08 q < 1
TLE1	transducin-like enhancer of split 1 (E(sp1) homolog, Drosophila)	Ssc.18454.1.A1_at	-1.17 q < 0.000498	-1.35 q < 0.0146	-1.14 q < 0.649	-1.1 q < 0.606	-1.22 q < 0.501	-1.05 q < 1
PNPO	pyridoxamine 5'-phosphate oxidase	Ssc.6484.1.S1_at	1.24 q < 0.000499	1.31 q < 0.373	1.28 q < 0.392	1.43 q < 0.0476	1.21 q < 1	1.03 q < 1
O95333	-	Ssc.5784.1.S1_at	-1.35 q < 0.000516	-1.22 q < 1	-1.8 q < 0.0125	-1.43 q < 0.209	-1.17 q < 1	-1.22 q < 1
SH3PXD2A	SH3 and PX domains 2A	Ssc.16978.1.A1_at	-1.25 q < 0.000527	-1.51 q < 0.0244	-1.17 q < 0.764	-1.31 q < 0.19	-1.17 q < 1	-1.11 q < 1
SH3PXD2A	SH3 and PX domains 2A	Ssc.16978.2.S1_at	-1.22 q < 0.00122	-1.46 q < 0.0309	-1.17 q < 0.726	-1.24 q < 0.32	-1.12 q < 1	-1.13 q < 1
IL21R	interleukin 21 receptor	Ssc.19416.1.A1_at	-1.43 q < 0.000537	-1.17 q < 1	-2.17 q < 0.00393	-1.33 q < 0.481	-1.74 q < 0.186	-1.02 q < 1
FAP	fibroblast activation protein, alpha	Ssc.4543.1.S1_at	-1.38 q < 0.000565	-2.92 q < 0.000000164	-1.3 q < 0.68	-1.18 q < 0.67	1.03 q < 1	-1.13 q < 1
RPL3	ribosomal protein L3	Ssc.11099.1.S1_at	-1.13 q < 0.000565	1.01 q < 1	-1.17 q < 0.234	-1.09 q < 0.497	-1.11 q < 1	-1.29 q < 0.00401
C4orf45	chromosome 4 open reading frame 45	Ssc.5870.3.S1_at	1.16 q < 0.00057	1.05 q < 1	-1.05 q < 0.94	1.27 q < 0.0616	1.22 q < 0.474	1.37 q < 0.0067
C14orf1	chromosome 14 open reading frame 1	Ssc.11458.1.S1_at	1.33 q < 0.000602	1.11 q < 1	1.21 q < 0.802	1.72 q < 0.0162	1.44 q < 0.575	1.27 q < 0.991

Gene Name	Gene Description	Probe Set ID	Breed MS-WC	Day 25 MS-WC	Day 45 MS-WC	Day 65 MS-WC	Day 85 MS-WC	Day 105 MS-WC
GLE1	GLE1 RNA export mediator homolog (yeast)	Ssc.1572.1.S1_at	-1.14 q < 0.000667	-1.04 q < 1	-1.32 q < 0.0079	-1.15 q < 0.334	-1.12 q < 1	-1.11 q < 1
LRRC8C	leucine rich repeat containing 8 family, member C	Ssc.13736.1.A1_at	-1.29 q < 0.000675	-1.02 q < 1	-1.4 q < 0.213	-1.19 q < 0.554	-1.64 q < 0.0233	-1.26 q < 0.87
RPS6KC1	ribosomal protein S6 kinase, 52kDa, polypeptide 1	Ssc.29354.1.A1_at	-1.26 q < 0.000681	-1.18 q < 1	-1.64 q < 0.00558	-1.04 q < 0.911	-1.32 q < 0.696	-1.2 q < 1
TYBN	-	Ssc.26266.1.S1_at	-1.25 q < 0.00072	-1.69 q < 0.00135	-1.15 q < 0.828	-1.11 q < 0.72	-1.19 q < 1	-1.18 q < 1
UMPS	uridine monophosphate synthetase	Ssc.1601.1.S1_at	1.17 q < 0.000724	1.12 q < 1	1.34 q < 0.0311	1.08 q < 0.715	1.24 q < 0.472	1.11 q < 1
ADAMTS3	ADAM metallopeptidase with thrombospondin type 1 motif, 3	Ssc.30575.1.A1_at	1.18 q < 0.000755	1.01 q < 1	1.16 q < 0.602	1.6 q < 0.0000295	1.12 q < 1	1.08 q < 1
PRDX3	peroxiredoxin 3	Ssc.1490.1.S1_at	-1.41 q < 0.000767	1.1 q < 1	-1.52 q < 0.332	-2.5 q < 0.000141	-1.54 q < 0.626	-1.05 q < 1
GUSB	glucuronidase, beta	Ssc.18585.1.S1_at	-1.31 q < 0.000772	-1.76 q < 0.00959	1.01 q < 0.997	-1.51 q < 0.089	-1.32 q < 1	-1.12 q < 1
LRP1B	low density lipoprotein receptor-related protein 1B	Ssc.8009.1.A1_at	-1.21 q < 0.00078	1.19 q < 0.827	-1.31 q < 0.19	-1.32 q < 0.121	-1.23 q < 1	-1.47 q < 0.013
HLA-DRB1	major histocompatibility complex, class II, DR beta 1	Ssc.16213.1.A1_x_at	1.25 q < 0.000792	-1.07 q < 1	1.03 q < 0.982	3.08 q < 0	-1.03 q < 1	1.08 q < 1
OXCT1	3-oxoacid CoA transferase 1	Ssc.11096.1.S1_at	1.19 q < 0.000797	1.63 q < 0.0000531	-1.05 q < 0.948	1.31 q < 0.0818	1.04 q < 1	1.12 q < 1
OXCT1	3-oxoacid CoA transferase 1	Ssc.27309.1.S1_at	1.17 q < 0.0089	1.64 q < 0.000213	-1.08 q < 0.911	1.31 q < 0.12	1.01 q < 1	1.08 q < 1
OXCT1	3-oxoacid CoA transferase 1	Ssc.3086.1.S1_at	1.14 q < 0.0126	1.3 q < 0.137	-1.06 q < 0.93	1.27 q < 0.126	1.08 q < 1	1.17 q < 0.848
KPNA5	karyopherin alpha 5 (importin alpha 6)	Ssc.15827.1.S1_at	1.15 q < 0.000798	1.07 q < 1	1.22 q < 0.171	1.32 q < 0.00768	1.05 q < 1	1.09 q < 1
ACAD8	acyl-CoA dehydrogenase family, member 8	Ssc.21283.1.S1_at	-1.66 q < 0.000802	-1.2 q < 1	-2.66 q < 0.0203	-1.97 q < 0.159	-1.49 q < 1	-1.35 q < 1
ACAD8	acyl-CoA dehydrogenase family, member 8	Ssc.21283.2.S1_at	-1.25 q < 0.0146	-1.14 q < 1	-1.51 q < 0.161	-1.27 q < 0.451	-1.11 q < 1	-1.25 q < 1
PLEC1	plectin	Ssc.28415.1.S1_at	-1.25 q < 0.000811	1.03 q < 1	-1.48 q < 0.0473	-1.21 q < 0.451	-1.36 q < 0.42	-1.28 q < 0.596
PTOV1	prostate tumor overexpressed 1	Ssc.18853.2.S1_a_at	1.21 q < 0.000821	1.3 q < 0.249	1.04 q < 0.958	1.53 q < 0.00287	1.04 q < 1	1.2 q < 0.855
Q8WTR8	-	Ssc.29810.1.A1_at	-1.14 q < 0.000824	1.01 q < 1	-1.36 q < 0.00136	-1.15 q < 0.288	-1.2 q < 0.396	-1.03 q < 1
RASSF2	Ras association (RalGDS/AF-6) domain family member 2	Ssc.11787.2.A1_at	-1.26 q < 0.000835	-1.98 q < 0.0000139	-1.15 q < 0.844	-1.15 q < 0.606	-1.06 q < 1	-1.13 q < 1
RASSF2	Ras association (RalGDS/AF-6) domain family member 2	Ssc.11787.1.S1_at	-1.12 q < 0.0554	-1.47 q < 0.00446	-1.08 q < 0.899	-1 q < 0.988	-1.05 q < 1	-1.06 q < 1
OGT	O-linked N-acetylglucosamine (GlcNAc) transferase (UDP-N-acetylglucosamine:polypeptide-N-acetylglucosaminyl transferase)	Ssc.8000.1.A1_at	-1.18 q < 0.000847	-1.08 q < 1	-1.36 q < 0.0343	-1.15 q < 0.475	-1.23 q < 0.688	-1.12 q < 1
OGT	O-linked N-acetylglucosamine (GlcNAc) transferase (UDP-N-acetylglucosamine:polypeptide-N-acetylglucosaminyl transferase)	Ssc.27417.1.S1_at	1.12 q < 0.108	1.21 q < 0.676	1.02 q < 0.99	1.39 q < 0.0354	-1.09 q < 1	1.12 q < 1
PLS3	plastin 3	Ssc.12172.1.A1_at	-1.15 q < 0.000847	-1.18 q < 0.453	-1.06 q < 0.907	-1.01 q < 0.957	-1.2 q < 0.519	-1.31 q < 0.02
TTYH2	tweety homolog 2 (Drosophila)	Ssc.2531.1.S1_at	1.22 q < 0.000863	1.01 q < 1	1.13 q < 0.844	1.51 q < 0.00813	1.21 q < 1	1.31 q < 0.317
HPS5	Hermansky-Pudlak syndrome 5	Ssc.24670.1.S1_at	-1.19 q < 0.000879	-1.16 q < 0.887	-1.5 q < 0.00252	-1.08 q < 0.746	-1.12 q < 1	-1.14 q < 1
HPS5	Hermansky-Pudlak syndrome 5	Ssc.24670.2.S1_at	-1.2 q < 0.00266	-1.21 q < 0.795	-1.51 q < 0.0108	1.01 q < 0.983	-1.14 q < 1	-1.23 q < 0.73
MEG3	maternally expressed 3 (non-protein coding)	Ssc.6192.1.A1_at	-1.61 q < 0.00088	-2.5 q < 0.0274	-1.39 q < 0.802	-1.12 q < 0.865	-1.43 q < 1	-1.96 q < 0.245
MEG3	maternally expressed 3 (non-protein coding)	Ssc.5159.2.S1_at	-1.43 q < 0.00233	-2.12 q < 0.0212	-1.29 q < 0.819	1.01 q < 0.983	-1.34 q < 1	-1.66 q < 0.325
DGCR8	DiGeorge syndrome critical region gene 8	Ssc.28251.1.A1_at	1.18 q < 0.000915	1.17 q < 0.822	1.06 q < 0.93	1.37 q < 0.0234	1.15 q < 1	1.19 q < 0.731
HMGCR	3-hydroxy-3-methylglutaryl-CoA reductase	Ssc.16088.1.S1_at	1.38 q < 0.000923	1.71 q < 0.104	-1.07 q < 0.96	1.76 q < 0.0415	1.47 q < 0.777	1.24 q < 1
FUS	fused in sarcoma	Ssc.11135.1.A1_at	-1.16 q < 0.000946	1.1 q < 1	-1.21 q < 0.31	-1.19 q < 0.271	-1.17 q < 1	-1.39 q < 0.00512
Q8N4P4	-	Ssc.1600.1.A1_a_at	1.25 q < 0.000969	1.07 q < 1	1.19 q < 0.724	1.64 q < 0.00311	1.17 q < 1	1.23 q < 0.862
Q8N4P4	-	Ssc.1600.3.S1_at	1.32 q < 0.000983	1.2 q < 1	1.21 q < 0.792	1.88 q < 0.00266	1.44 q < 0.554	1.01 q < 1
Q8N4P4	-	Ssc.1600.1.A1_at	1.24 q < 0.00443	-1.01 q < 1	1.16 q < 0.843	1.68 q < 0.00432	1.26 q < 1	1.2 q < 1
TRAPPC9	trafficking protein particle complex 9	Ssc.7549.1.A1_at	1.16 q < 0.000987	-1.03 q < 1	1.15 q < 0.597	1.33 q < 0.0151	1.15 q < 1	1.22 q < 0.297
CYP4B1	cytochrome P450, family 4, subfamily B, polypeptide 1	Ssc.19211.1.S1_at	1.48 q < 0.001	1.1 q < 1	1.87 q < 0.107	-1.15 q < 0.786	1.93 q < 0.145	2.08 q < 0.0353
ABCA5	ATP-binding cassette, sub-family A (ABC1), member 5	Ssc.30026.1.A1_at	1.17 q < 0.00102	1.09 q < 1	1.06 q < 0.933	1.48 q < 0.000904	1.18 q < 1	1.11 q < 1
DNAH9	dynein, axonemal, heavy chain 9	Ssc.22302.1.A1_at	1.18 q < 0.00103	-1 q < 1	1.14 q < 0.707	1.21 q < 0.267	1.17 q < 1	1.4 q < 0.0128
EBP	emopamil binding protein (sterol isomerase)	Ssc.5045.1.S1_at	1.32 q < 0.00103	1.87 q < 0.0042	-1.14 q < 0.898	1.39 q < 0.25	1.32 q < 1	1.34 q < 0.706
EBP	SH3 domain containing 19	Ssc.5045.1.S1_at	1.32 q < 0.00103	1.87 q < 0.0042	-1.14 q < 0.898	1.39 q < 0.25	1.32 q < 1	1.34 q < 0.706
EBP	galactosidase, beta 1	Ssc.5045.1.S1_at	1.32 q < 0.00103	1.87 q < 0.0042	-1.14 q < 0.898	1.39 q < 0.25	1.32 q < 1	1.34 q < 0.706
PHCA	alkaline ceramidase 3	Ssc.10292.1.S1_at	1.24 q < 0.00104	-1.12 q < 1	1.11 q < 0.9	1.73 q < 0.000495	1.26 q < 1	1.36 q < 0.231
PDGFD	platelet derived growth factor D	Ssc.10303.1.A1_at	-1.39 q < 0.00104	-2.16 q < 0.00216	-1.61 q < 0.168	-1.32 q < 0.466	-1.08 q < 1	-1.03 q < 1

Gene Name	Gene Description	Probe Set ID	Breed MS-WC	Day 25 MS-WC	Day 45 MS-WC	Day 65 MS-WC	Day 85 MS-WC	Day 105 MS-WC
PAPOLA	poly(A) polymerase alpha	Ssc.21300.1.S1_at	-1.21 q < 0.00106	1.15 q < 1	-1.44 q < 0.0223	-1.34 q < 0.0922	-1.2 q < 1	-1.27 q < 0.404
PHYH	phytanoyl-CoA 2-hydroxylase	Ssc.8139.1.S1_at	-1.26 q < 0.00107	1.01 q < 1	-1.52 q < 0.0407	-1.3 q < 0.288	-1.26 q < 1	-1.28 q < 0.673
C1QA	complement component 1, q subcomponent, A chain	Ssc.833.1.S1_at	-1.64 q < 0.00108	-1.04 q < 1	-1.27 q < 0.898	-1.3 q < 0.673	-1.17 q < 1	-5.92 q < 0.0000000502
GABARAPL1	GABA(A) receptor-associated protein like 1	Ssc.22588.1.S1_at	-1.19 q < 0.00112	-1.05 q < 1	-1.32 q < 0.106	-1.42 q < 0.0117	-1.25 q < 0.585	1.04 q < 1
GABARAPL1	GABA(A) receptor-associated protein like 1	Ssc.22588.2.S1_at	-1.13 q < 0.0345	-1.04 q < 1	-1.16 q < 0.642	-1.21 q < 0.269	-1.14 q < 1	-1.1 q < 1
CYBA	cytochrome b-245, alpha polypeptide	Ssc.16296.1.S1_at	-1.26 q < 0.00113	-1.23 q < 0.857	-1.22 q < 0.673	-1.19 q < 0.537	-1.09 q < 1	-1.63 q < 0.00944
RPL4	ribosomal protein L4	Ssc.12277.1.S1_at	-1.16 q < 0.00117	-1.09 q < 1	-1.22 q < 0.259	-1.06 q < 0.761	-1.14 q < 1	-1.31 q < 0.0478
PTHLH	parathyroid hormone-like hormone	Ssc.9991.1.S1_at	1.19 q < 0.00127	1.07 q < 1	1.32 q < 0.102	1.51 q < 0.00148	1.04 q < 1	1.06 q < 1
GAPDH	glyceraldehyde-3-phosphate dehydrogenase	AFFX-Ssc-gapdh-3_at	1.17 q < 0.00129	1.24 q < 0.302	1.06 q < 0.934	1.43 q < 0.00346	1.16 q < 1	1.01 q < 1
GAPDH	glyceraldehyde-3-phosphate dehydrogenase	Ssc.14942.1.S1_at	1.13 q < 0.0221	1.2 q < 0.522	1.02 q < 0.989	1.35 q < 0.0197	1.12 q < 1	-1.01 q < 1
ATMIN	ATM interactor	Ssc.6346.1.S1_at	1.24 q < 0.00133	1.18 q < 1	1.08 q < 0.93	1.54 q < 0.0153	1.29 q < 0.823	1.16 q < 1
AKAP2	A kinase (PRKA) anchor protein 2	Ssc.1407.3.S1_at	-1.27 q < 0.00134	-1.01 q < 1	-1.62 q < 0.0184	-1.27 q < 0.386	-1.39 q < 0.519	-1.15 q < 1
AKAP2	A kinase (PRKA) anchor protein 2	Ssc.1407.2.S1_at	-1.24 q < 0.0179	-1.05 q < 1	-1.63 q < 0.0424	-1.21 q < 0.549	-1.34 q < 1	-1.05 q < 1
CCR5	chemokine (C-C motif) receptor 5	Ssc.26328.1.S1_at	-1.18 q < 0.00138	-1.08 q < 1	-1.13 q < 0.8	-1.1 q < 0.655	-1.12 q < 1	-1.53 q < 0.000838
SYNJ2	synaptojanin 2	Ssc.14132.1.A1_at	-1.16 q < 0.00142	-1.08 q < 1	-1.35 q < 0.0232	-1.05 q < 0.801	-1.1 q < 1	-1.26 q < 0.194
PDHA2	pyruvate dehydrogenase (lipoamide) alpha 2	Ssc.25479.1.S1_at	1.12 q < 0.00144	-1 q < 1	1.07 q < 0.865	1.28 q < 0.00923	1.13 q < 1	1.17 q < 0.377
CLIC4	chloride intracellular channel 4	Ssc.14592.2.A1_at	1.42 q < 0.00157	1.88 q < 0.0698	-1.13 q < 0.937	2.34 q < 0.00187	1.29 q < 1	1.15 q < 1
CLIC4	chloride intracellular channel 4	Ssc.26192.1.S1_at	1.13 q < 0.0622	1.08 q < 1	-1.07 q < 0.921	1.51 q < 0.00202	-1.02 q < 1	1.22 q < 0.6
CDR2	cerebellar degeneration-related protein 2, 62kDa	Ssc.3394.3.A1_at	1.23 q < 0.00159	1.08 q < 1	1.33 q < 0.239	1.14 q < 0.609	1.13 q < 1	1.51 q < 0.025
DNAJC1	DnaJ (Hsp40) homolog, subfamily C, member 1	Ssc.27322.1.S1_at	1.22 q < 0.00162	-1.15 q < 1	1.16 q < 0.776	1.54 q < 0.00682	1.33 q < 0.423	1.3 q < 0.391
GKP3	glycerol kinase 3 pseudogene	Ssc.22221.1.S1_a_at	1.25 q < 0.00169	1.31 q < 0.491	-1 q < 1	1.83 q < 0.000203	1.08 q < 1	1.17 q < 1
LOX	lysyl oxidase	Ssc.8224.1.S1_at	-1.46 q < 0.00173	-1.52 q < 0.62	-1.37 q < 0.726	-1.2 q < 0.714	-1.25 q < 1	-2.11 q < 0.0287
LOX	lysyl oxidase	Ssc.10386.1.S1_at	-1.38 q < 0.0123	-2.07 q < 0.0463	-1.27 q < 0.845	-1.1 q < 0.859	-1.21 q < 1	-1.43 q < 0.926
LIMD1	LIM domains containing 1	Ssc.30639.1.S1_at	-1.24 q < 0.00173	-1.04 q < 1	-1.65 q < 0.00407	-1.3 q < 0.256	-1.14 q < 1	-1.15 q < 1
TCEA2	transcription elongation factor A (SII), 2	Ssc.15645.1.S1_at	-1.2 q < 0.00174	-1.25 q < 0.469	-1.48 q < 0.0111	-1.24 q < 0.261	-1.03 q < 1	-1.05 q < 1
CYP26B1	cytochrome P450, family 26, subfamily B, polypeptide 1	Ssc.22436.1.S1_at	1.58 q < 0.00175	1.24 q < 1	2.12 q < 0.107	2.24 q < 0.0485	1.21 q < 1	1.37 q < 1
ABCC1	ATP-binding cassette, sub-family C (CFTR/MRP), member 1	Ssc.19612.1.S1_at	1.18 q < 0.00188	1.03 q < 1	1.13 q < 0.792	1.08 q < 0.736	1.14 q < 1	1.63 q < 0.000105
ABCC1	ATP-binding cassette, sub-family C (CFTR/MRP), member 1	Ssc.14287.1.S1_at	1.12 q < 0.0652	-1.11 q < 1	1.08 q < 0.908	1.16 q < 0.444	1.08 q < 1	1.45 q < 0.00807
HSD17B14	hydroxysteroid (17-beta) dehydrogenase 14	Ssc.19146.1.S1_at	1.26 q < 0.00192	1.34 q < 0.453	-1.24 q < 0.628	1.54 q < 0.0335	1.28 q < 1	1.49 q < 0.0958
GPC5	glypican 5	Ssc.20047.1.S1_at	1.17 q < 0.00192	1.51 q < 0.00124	1.09 q < 0.894	1.17 q < 0.401	1.02 q < 1	1.14 q < 1
GPC5	glypican 5	Ssc.9154.1.A1_at	-1.16 q < 0.00333	-1.08 q < 1	-1.2 q < 0.448	-1.29 q < 0.0946	-1.2 q < 0.913	-1.06 q < 1
GSTA4	glutathione S-transferase alpha 4	Ssc.5008.1.A1_at	-1.24 q < 0.00195	-1.08 q < 1	-1.28 q < 0.464	-1.29 q < 0.288	-1.06 q < 1	-1.57 q < 0.0207
COL3A1	collagen, type III, alpha 1	Ssc.11302.1.S1_at	-1.38 q < 0.00197	-1.02 q < 1	-1.44 q < 0.462	-1.08 q < 0.879	-1.19 q < 1	-2.65 q < 0.0000303
COL3A1	collagen, type III, alpha 1	Ssc.11302.1.S2_at	-1.46 q < 0.00512	-1.04 q < 1	-1.48 q < 0.622	-1.25 q < 0.682	-1.13 q < 1	-3.02 q < 0.000334
WARS	tryptophanyl-tRNA synthetase	Ssc.6139.1.S1_at	-1.18 q < 0.00203	-1.12 q < 1	-1.28 q < 0.181	1.01 q < 0.972	-1.16 q < 1	-1.38 q < 0.0335
GEMIN7	gem (nuclear organelle) associated protein 7	Ssc.15453.3.S1_a_at	-1.16 q < 0.00204	-1.12 q < 0.996	-1.32 q < 0.0387	-1.07 q < 0.718	-1.07 q < 1	-1.21 q < 0.42
C3orf59	chromosome 3 open reading frame 59	Ssc.8346.1.A1_at	1.27 q < 0.00206	-1.01 q < 1	1.07 q < 0.953	1.77 q < 0.00269	1.26 q < 1	1.4 q < 0.316
UBA6	ubiquitin-like modifier activating enzyme 6	Ssc.13681.1.S1_at	1.16 q < 0.00206	1.11 q < 1	1.05 q < 0.943	1.59 q < 0.00000927	1.03 q < 1	1.08 q < 1
ELMO2	engulfment and cell motility 2	Ssc.2298.1.S1_at	-1.16 q < 0.00211	-1.09 q < 1	-1.16 q < 0.583	1.02 q < 0.948	-1.22 q < 0.63	-1.39 q < 0.011
TTC29	tetratricopeptide repeat domain 29	Ssc.28065.1.A1_at	-1.14 q < 0.00213	-1.02 q < 1	-1.03 q < 0.958	-1.22 q < 0.107	-1.47 q < 0.0000812	-1.01 q < 1
RASSF1	Ras association (RalGDS/AF-6) domain family member 1	Ssc.2077.1.S1_at	1.16 q < 0.00222	1.32 q < 0.0589	-1.14 q < 0.691	1.84 q < 0.00000000491	1.1 q < 1	-1.11 q < 1
ZBP1	Z-DNA binding protein 1	Ssc.26005.1.S1_at	1.15 q < 0.00224	1.07 q < 1	1.19 q < 0.384	1.3 q < 0.041	1.28 q < 0.158	-1.04 q < 1
PTGFRN	prostaglandin F2 receptor negative regulator	Ssc.11942.1.S1_at	-1.18 q < 0.00225	-1.52 q < 0.00175	-1.04 q < 0.958	1.04 q < 0.874	-1.18 q < 1	-1.28 q < 0.247
PTGFRN	prostaglandin F2 receptor negative regulator	Ssc.12024.1.A1_at	-1.14 q < 0.0103	-1.37 q < 0.0144	-1.07 q < 0.902	1.01 q < 0.979	-1.12 q < 1	-1.16 q < 0.835
PTGFRN	prostaglandin F2 receptor negative regulator	Ssc.12992.1.S1_at	-1.23 q < 0.02	-1.32 q < 0.684	-1.07 q < 0.955	-1.1 q < 0.8	-1.29 q < 1	-1.43 q < 0.338
Q96I09	-	Ssc.6206.1.S1_at	-1.23 q < 0.00226	-1.1 q < 1	-1.22 q < 0.617	-1.47 q < 0.0325	-1.13 q < 1	-1.23 q < 0.849
Q5TF57	-	Ssc.21179.1.S1_at	1.39 q < 0.00227	1.04 q < 1	1.46 q < 0.457	1.93 q < 0.0215	1.18 q < 1	1.5 q < 0.539

Gene Name	Gene Description	Probe Set ID	Breed MS-WC	Day 25 MS-WC	Day 45 MS-WC	Day 65 MS-WC	Day 85 MS-WC	Day 105 MS-WC
KIAA2022	KIAA2022	Ssc.15351.1.A1_at	-1.2 q < 0.00228	1.04 q < 1	-1.31 q < 0.216	-1.47 q < 0.0147	-1.3 q < 0.516	-1.05 q < 1
CRYM	crystallin, mu	Ssc.30236.1.A1_at	1.27 q < 0.00229	1.3 q < 0.658	-1.02 q < 0.992	-1.03 q < 0.931	1.56 q < 0.0898	1.7 q < 0.00958
SLC6A6	solute carrier family 6 (neurotransmitter transporter, taurine), member 6	Ssc.6154.1.S1_at	-1.15 q < 0.00236	1.02 q < 1	-1.25 q < 0.147	-1.33 q < 0.0178	-1.07 q < 1	-1.15 q < 0.852
FAM49A	family with sequence similarity 49, member A	Ssc.8340.1.A1_at	-1.2 q < 0.00245	-1.13 q < 1	-1.46 q < 0.0232	-1.24 q < 0.3	-1.08 q < 1	-1.13 q < 1
GLRX5	glutaredoxin 5	Ssc.11338.1.S1_a_at	-1.33 q < 0.0025	-1.01 q < 1	-1.75 q < 0.0373	-1.48 q < 0.197	-1.56 q < 0.348	-1.03 q < 1
GLRX5	glutaredoxin 5	Ssc.6703.1.S1_at	-1.16 q < 0.000879	1.05 q < 1	-1.23 q < 0.241	-1.13 q < 0.491	-1.27 q < 0.199	-1.27 q < 0.124
IDH2	isocitrate dehydrogenase 2 (NADP+), mitochondrial	Ssc.2449.1.S1_at	-1.23 q < 0.0025	1.18 q < 1	-1.18 q < 0.763	-1.3 q < 0.246	-1.44 q < 0.155	-1.5 q < 0.0351
PDE4D	phosphodiesterase 4D, cAMP-specific	Ssc.29848.1.A1_at	1.27 q < 0.0025	1.07 q < 1	1.06 q < 0.959	1.67 q < 0.0115	1.31 q < 1	1.33 q < 0.627
RPL31	ribosomal protein L31	Ssc.3747.1.A1_x_at	-1.15 q < 0.0025	1.04 q < 1	-1.2 q < 0.354	-1.08 q < 0.705	-1.12 q < 1	-1.45 q < 0.00081
RPL31	ribosomal protein L31	Ssc.3747.1.A1_at	-1.13 q < 0.0189	1.05 q < 1	-1.18 q < 0.521	-1.05 q < 0.84	-1.1 q < 1	-1.45 q < 0.00253
RPL31	ribosomal protein L31	Ssc.30785.1.S1_at	1.14 q < 0.0159	1.11 q < 1	1.05 q < 0.94	1.23 q < 0.209	1.08 q < 1	1.24 q < 0.321
SLC5A1	solute carrier family 5 (sodium/glucose cotransporter), member 1	Ssc.22229.1.A1_at	-1.15 q < 0.0025	-1.46 q < 0.000441	-1.11 q < 0.796	-1.1 q < 0.587	-1.09 q < 1	-1.02 q < 1
DAB2	disabled homolog 2, mitogen-responsive phosphoprotein (Drosophila)	Ssc.9118.1.S1_at	-1.37 q < 0.00251	-1.38 q < 0.769	-1.17 q < 0.899	-2.28 q < 0.000784	-1.19 q < 1	-1.09 q < 1
DAB2	disabled homolog 2, mitogen-responsive phosphoprotein (Drosophila)	Ssc.2807.1.A1_at	-1.36 q < 0.0339	-1.26 q < 1	-1.22 q < 0.899	-2.22 q < 0.0212	-1.21 q < 1	-1.13 q < 1
IL17RD	interleukin 17 receptor D	Ssc.30983.1.A1_at	-1.16 q < 0.00251	-1.03 q < 1	-1.18 q < 0.517	1.03 q < 0.885	-1.52 q < 0.000487	-1.18 q < 0.714
SGCG	sarcoglycan, gamma (35kDa dystrophin-associated glycoprotein)	Ssc.21763.1.A1_at	-1.13 q < 0.00251	-1.11 q < 0.987	-1.31 q < 0.0125	1.03 q < 0.868	-1.12 q < 1	-1.17 q < 0.48
ELOVL1	elongation of very long chain fatty acids (FEN1/Elo2, SUR4/Elo3, yeast)-like 1	Ssc.5650.1.S1_at	1.26 q < 0.00253	1.04 q < 1	1.12 q < 0.903	1.53 q < 0.0481	1.31 q < 1	1.37 q < 0.406
BECN1	beclin 1, autophagy related	Ssc.4845.1.S1_at	1.18 q < 0.00255	1.22 q < 0.574	1.01 q < 0.997	1.52 q < 0.00191	1.05 q < 1	1.19 q < 0.86
ZNF277	zinc finger protein 277	Ssc.1012.1.S1_at	-1.21 q < 0.00262	-1.04 q < 1	-1.46 q < 0.036	-1.08 q < 0.773	-1.23 q < 1	-1.29 q < 0.442
ABHD6	abhydrolase domain containing 6	Ssc.6276.1.S1_at	-1.23 q < 0.00264	1.06 q < 1	-1.27 q < 0.48	-1.19 q < 0.521	-1.33 q < 0.611	-1.5 q < 0.0496
ADI1	acireductone dioxygenase 1	Ssc.14003.4.S1_at	-1.27 q < 0.00273	1.02 q < 1	-1.94 q < 0.000367	-1.08 q < 0.834	-1.28 q < 1	-1.24 q < 1
ABCB11	ATP-binding cassette, sub-family B (MDR/TAP), member 11	Ssc.16256.1.S1_at	1.33 q < 0.00275	1.06 q < 1	1.29 q < 0.695	1.93 q < 0.00595	1.24 q < 1	1.28 q < 1
MAGED2	melanoma antigen family D, 2	Ssc.11376.1.A1_a_at	-1.18 q < 0.00277	1.01 q < 1	-1.4 q < 0.0347	-1.29 q < 0.132	-1.12 q < 1	-1.16 q < 1
TFAP2B	transcription factor AP-2 beta (activating enhancer binding protein 2 beta)	Ssc.17350.1.A1_at	1.13 q < 0.00285	-1.04 q < 1	1.08 q < 0.842	1.35 q < 0.00165	1.12 q < 1	1.16 q < 0.601
EFNB2	ephrin-B2	Ssc.27799.1.S1_at	-1.18 q < 0.0029	-1.11 q < 1	-1.5 q < 0.00342	-1.05 q < 0.855	-1.15 q < 1	-1.13 q < 1
CAMK1G	calcium/calmodulin-dependent protein kinase IG	Ssc.27351.1.S1_at	1.16 q < 0.00305	1.17 q < 0.733	1.15 q < 0.664	1.31 q < 0.045	1.07 q < 1	1.11 q < 1
DHDH	dihydrodiol dehydrogenase (dimeric)	Ssc.16041.1.S1_at	-1.17 q < 0.00312	1.03 q < 1	-1.47 q < 0.00307	-1.2 q < 0.31	-1.18 q < 1	-1.07 q < 1
WFDC1	WAP four-disulfide core domain 1	Ssc.9108.1.S1_at	1.22 q < 0.00312	1.16 q < 1	1.2 q < 0.69	1.58 q < 0.00594	1.04 q < 1	1.17 q < 1
COL1A2	collagen, type I, alpha 2	Ssc.21011.1.S1_at	-1.47 q < 0.00317	-1.05 q < 1	-1.74 q < 0.254	-1.24 q < 0.682	-1.2 q < 1	-2.5 q < 0.00564
COL1A2	collagen, type I, alpha 2	Ssc.24975.1.S1_at	-1.47 q < 0.00579	-1.21 q < 1	-1.79 q < 0.26	-1.28 q < 0.65	-1.06 q < 1	-2.34 q < 0.0257
POLR3A	polymerase (RNA) III (DNA directed) polypeptide A, 155kDa	Ssc.9561.1.A1_at	-1.28 q < 0.00317	-1.18 q < 1	-1.19 q < 0.829	-1.14 q < 0.703	1.01 q < 1	-2.14 q < 0.0000386
PIH1D1	PIH1 domain containing 1	Ssc.12478.1.S1_at	-1.14 q < 0.00319	1.04 q < 1	-1.29 q < 0.0331	-1.21 q < 0.147	-1.08 q < 1	-1.15 q < 0.731
CYB5R3	cytochrome b5 reductase 3	Ssc.16727.1.A1_at	-1.2 q < 0.00321	1.23 q < 0.671	-1.59 q < 0.00248	-1.21 q < 0.38	-1.32 q < 0.416	-1.2 q < 0.912
GLT8D1	glycosyltransferase 8 domain containing 1	Ssc.11574.1.A1_at	-1.2 q < 0.00328	-1.58 q < 0.00284	-1.23 q < 0.514	-1.04 q < 0.887	-1.03 q < 1	-1.2 q < 0.92
CF365427	-	Ssc.22094.1.A1_at	-1.15 q < 0.00329	-1.12 q < 1	-1.06 q < 0.918	-1.23 q < 0.158	-1.37 q < 0.0233	-1.02 q < 1
TNFRSF21	tumor necrosis factor receptor superfamily, member 21	Ssc.1509.1.S1_at	-1.26 q < 0.00329	-1.64 q < 0.0184	-1.27 q < 0.572	-1.21 q < 0.525	-1.07 q < 1	-1.16 q < 1
BRD3	bromodomain containing 3	Ssc.4207.1.S1_at	-1.19 q < 0.00336	-1.06 q < 1	-1.45 q < 0.0212	-1.06 q < 0.833	-1.25 q < 0.828	-1.18 q < 0.955
CYP4A11	cytochrome P450, family 4, subfamily A, polypeptide 11	Ssc.15895.1.S1_s_at	1.54 q < 0.00337	1.37 q < 1	1.22 q < 0.913	1.33 q < 0.626	2.82 q < 0.00838	1.38 q < 1
STX8	syntaxin 8	Ssc.8421.1.S1_at	-1.18 q < 0.00339	-1.49 q < 0.00408	1 q < 1	-1.21 q < 0.327	1 q < 1	-1.25 q < 0.402
STX8	syntaxin 8	Ssc.17842.2.S1_a_at	-1.14 q < 0.0052	1.03 q < 1	-1.07 q < 0.896	-1.2 q < 0.212	-1.25 q < 0.303	-1.22 q < 0.301
C1orf135	chromosome 1 open reading frame 135	Ssc.25735.1.S1_at	1.11 q < 0.00347	1.1 q < 0.901	1.04 q < 0.932	1.25 q < 0.0184	1.08 q < 1	1.1 q < 1
RXRG	retinoid X receptor, gamma	Ssc.19608.1.S1_at	1.15 q < 0.00352	1.03 q < 1	1.1 q < 0.844	1.4 q < 0.00333	1.15 q < 1	1.1 q < 1
SLK	STE20-like kinase	Ssc.8732.1.A1_at	-1.18 q < 0.00355	-1.12 q < 1	-1.47 q < 0.0108	1.03 q < 0.924	-1.23 q < 0.925	-1.17 q < 0.971
SLK	FYN oncogene related to SRC, FGR, YES	Ssc.8732.1.A1_at	-1.18 q < 0.00355	-1.12 q < 1	-1.47 q < 0.0108	1.03 q < 0.924	-1.23 q < 0.925	-1.17 q < 0.971

Gene Name	Gene Description	Probe Set ID	Breed MS-WC	Day 25 MS-WC	Day 45 MS-WC	Day 65 MS-WC	Day 85 MS-WC	Day 105 MS-WC
ENPP1	ectonucleotide pyrophosphatase/phosphodiesterase 1	Ssc.4913.1.A1_at	-1.24 q < 0.00359	-1.09 q < 1	-1.53 q < 0.0474	-1.17 q < 0.595	-1.13 q < 1	-1.35 q < 0.433
PEPD	peptidase D	Ssc.776.1.S1_a_at	-1.25 q < 0.00378	1 q < 1	-1.39 q < 0.251	-1.21 q < 0.523	-1.14 q < 1	-1.61 q < 0.0254
LYVE1	lymphatic vessel endothelial hyaluronan receptor 1	Ssc.5047.1.A1_at	-1.46 q < 0.00387	-1.16 q < 1	-1.33 q < 0.812	-1.31 q < 0.596	-1.51 q < 1	-2.18 q < 0.034
LYVE1	lymphatic vessel endothelial hyaluronan receptor 1	Ssc.17343.1.S1_at	-1.33 q < 0.00762	-1.11 q < 1	-1.25 q < 0.814	-1.17 q < 0.714	-1.37 q < 1	-1.87 q < 0.0335
GLRXL	glutaredoxin (thioltransferase) pseudogene 3	Ssc.709.1.S1_at	-1.48 q < 0.00399	-1.1 q < 1	-1.29 q < 0.855	-2.18 q < 0.0325	-1.56 q < 1	-1.47 q < 0.947
HMGB3	high-mobility group box 3	Ssc.11434.1.S1_at	1.26 q < 0.00406	1.06 q < 1	-1.01 q < 0.997	1.76 q < 0.00346	1.27 q < 1	1.33 q < 0.594
HMGB3	high-mobility group box 3	Ssc.27311.1.S1_at	1.21 q < 0.00831	1.18 q < 1	1 q < 1	1.57 q < 0.0126	1.24 q < 1	1.14 q < 1
EXOSC3	exosome component 3	Ssc.24317.2.S1_at	1.14 q < 0.00407	1.15 q < 0.777	1.1 q < 0.809	1.3 q < 0.031	1.09 q < 1	1.07 q < 1
NIT1	nitrilase 1	Ssc.1294.3.S1_at	1.16 q < 0.00407	1.04 q < 1	1.07 q < 0.921	1.38 q < 0.0185	1.22 q < 0.772	1.13 q < 1
SIDT1	SID1 transmembrane family, member 1	Ssc.6634.1.A1_at	2.55 q < 0.00407	2.12 q < 1	2.86 q < 0.529	5.81 q < 0.0486	1.59 q < 1	1.91 q < 1
SIDT1	SID1 transmembrane family, member 1	Ssc.26734.1.A1_at	-1.17 q < 0.145	-1.69 q < 0.0247	-1.11 q < 0.917	-1.07 q < 0.853	-1.09 q < 1	1.01 q < 1
ZNF716	zinc finger protein 716	Ssc.27062.1.A1_at	-1.21 q < 0.00408	1.01 q < 1	-1.27 q < 0.403	-1.12 q < 0.666	-1.2 q < 1	-1.51 q < 0.018
ACLY	ATP citrate lyase	Ssc.30707.1.S1_at	1.21 q < 0.00408	1.59 q < 0.0059	-1.06 q < 0.943	1.3 q < 0.209	1.24 q < 1	1.07 q < 1
PTX3	pentraxin 3, long	Ssc.8162.2.S1_at	-1.41 q < 0.00419	-1.52 q < 0.581	-2.04 q < 0.0309	-1.45 q < 0.382	-1.14 q < 1	-1.09 q < 1
PTX3	pentraxin 3, long	Ssc.8162.1.S1_at	-1.42 q < 0.0174	-1.69 q < 0.494	-2.01 q < 0.119	-1.42 q < 0.506	-1.29 q < 1	1.07 q < 1
KIRREL3	kin of IRRE like 3 (Drosophila)	Ssc.19197.2.A1_at	1.25 q < 0.00421	1 q < 1	1.21 q < 0.741	1.85 q < 0.000672	-1.02 q < 1	1.38 q < 0.365
NUDT9	nudix (nucleoside diphosphate linked moiety X)-type motif 9	Ssc.23132.1.A1_at	1.2 q < 0.00422	1.29 q < 0.36	1.01 q < 0.995	1.46 q < 0.0222	1.08 q < 1	1.19 q < 0.937
PRKAR2B	protein kinase, cAMP-dependent, regulatory, type II, beta	Ssc.1056.1.A1_at	-1.2 q < 0.00422	-1.46 q < 0.0348	-1.08 q < 0.919	-1.18 q < 0.469	-1.22 q < 1	-1.08 q < 1
ACSF2	acyl-CoA synthetase family member 2	Ssc.12161.1.S1_at	-1.19 q < 0.00433	1.05 q < 1	-1.42 q < 0.0367	-1.22 q < 0.345	-1.07 q < 1	-1.33 q < 0.181
PLSCR4	phospholipid scramblase 4	Ssc.5708.1.A1_at	1.14 q < 0.00433	1.12 q < 1	1.06 q < 0.924	1.14 q < 0.452	-1.02 q < 1	1.48 q < 0.000253
F13A1	coagulation factor XIII, A1 polypeptide	Ssc.26345.1.S1_at	-1.49 q < 0.00436	-1.03 q < 1	-1.4 q < 0.749	1.07 q < 0.923	-1.41 q < 1	-3.83 q < 0.0000104
F13A1	coagulation factor XIII, A1 polypeptide	Ssc.26345.3.S1_at	-1.18 q < 0.00473	-1.13 q < 1	-1.09 q < 0.903	1.1 q < 0.677	-1.22 q < 1	-1.66 q < 0.0000747
CDK8	cyclin-dependent kinase 8	Ssc.7933.1.A1_at	-1.21 q < 0.00451	-1 q < 1	-1.48 q < 0.0315	-1.1 q < 0.736	-1.37 q < 0.272	-1.14 q < 1
PLA2G7	phospholipase A2, group VII (platelet-activating factor acetylhydrolase, plasma)	Ssc.19691.1.S1_at	-1.2 q < 0.00459	1.02 q < 1	-1.11 q < 0.89	-1.07 q < 0.822	-1.51 q < 0.0251	-1.43 q < 0.0597
GBAS	glioblastoma amplified sequence	Ssc.10949.1.S1_at	-1.22 q < 0.00464	-1.16 q < 1	-1.52 q < 0.0243	-1.16 q < 0.571	-1.15 q < 1	-1.13 q < 1
SLC5A6	solute carrier family 5 (sodium-dependent vitamin transporter), member 6	Ssc.2516.3.S1_a_at	1.62 q < 0.00464	1.34 q < 1	1.72 q < 0.536	2.51 q < 0.0465	1.1 q < 1	1.74 q < 0.732
SLC5A6	solute carrier family 5 (sodium-dependent vitamin transporter), member 6	Ssc.2516.2.S1_a_at	1.36 q < 0.0491	1.23 q < 1	1.29 q < 0.856	1.9 q < 0.117	1.01 q < 1	1.51 q < 0.875
PSMA4	proteasome (prosome, macropain) subunit, alpha type, 4	Ssc.30.1.S1_at	1.16 q < 0.00464	1.24 q < 0.358	1 q < 0.998	1.35 q < 0.0382	1.23 q < 0.694	1.03 q < 1
CTR9	Ctr9, Paf1/RNA polymerase II complex component, homolog (S. cerevisiae)	Ssc.30790.1.A1_at	1.2 q < 0.00478	1.2 q < 0.857	-1.03 q < 0.982	1.46 q < 0.0244	1.34 q < 0.409	1.09 q < 1
CTR9	Ctr9, Paf1/RNA polymerase II complex component, homolog (S. cerevisiae)	Ssc.22181.1.S1_at	1.18 q < 0.0000152	1.14 q < 0.772	1.17 q < 0.396	1.23 q < 0.0866	1.15 q < 1	1.23 q < 0.139
S100PBP	S100P binding protein	Ssc.27465.1.S1_at	-1.22 q < 0.00478	-1.17 q < 1	-1.31 q < 0.355	1.43 q < 0.0773	-1.86 q < 0.000191	-1.37 q < 0.288
S100PBP	S100P binding protein	Ssc.9376.1.A1_at	-1.05 q < 0.715	-1.08 q < 1	-1.21 q < 0.57	1.55 q < 0.00359	-1.36 q < 0.252	-1.14 q < 1
ARID1B	AT rich interactive domain 1B (SWI1-like)	Ssc.27166.1.S1_at	-1.26 q < 0.00487	-1.19 q < 1	-1.08 q < 0.943	-1.56 q < 0.0463	-1.17 q < 1	-1.36 q < 0.512
ARID1B	AT rich interactive domain 1B (SWI1-like)	SscAffx.15.1.S1_s_at	1.16 q < 0.00435	1.05 q < 1	1.01 q < 0.99	1.31 q < 0.0718	1.2 q < 1	1.27 q < 0.247
XIAP	X-linked inhibitor of apoptosis	Ssc.8950.1.A1_at	1.09 q < 0.00493	1.04 q < 1	1.07 q < 0.791	1.21 q < 0.0135	1.1 q < 1	1.04 q < 1
XIAP	X-linked inhibitor of apoptosis	Ssc.10175.1.S1_at	-1.15 q < 0.0176	-1.09 q < 1	-1.41 q < 0.0287	-1.02 q < 0.94	-1.18 q < 1	-1.1 q < 1
RAMP3	receptor (G protein-coupled) activity modifying protein 3	Ssc.15751.1.S1_a_at	1.16 q < 0.00506	1.08 q < 1	-1.02 q < 0.987	1.12 q < 0.589	1.14 q < 1	1.56 q < 0.000227
C4orf33	chromosome 4 open reading frame 33	Ssc.20478.1.S1_at	1.15 q < 0.00509	1.52 q < 0.000383	1.19 q < 0.488	1.11 q < 0.583	1.03 q < 1	-1.01 q < 1
GPATCH3	G patch domain containing 3	Ssc.27126.1.A1_at	1.13 q < 0.00514	1.05 q < 1	1.06 q < 0.911	1.31 q < 0.0148	1.12 q < 1	1.13 q < 0.99
ATP5J	ATP synthase, H+ transporting, mitochondrial Fo complex, subunit F6	Ssc.16536.1.S1_at	-1.24 q < 0.00524	1 q < 1	-1.65 q < 0.0143	-1.24 q < 0.455	-1.14 q < 1	-1.29 q < 0.752
TMEM144	transmembrane protein 144	Ssc.23305.1.S1_at	1.28 q < 0.00524	1.12 q < 1	-1.09 q < 0.943	1.44 q < 0.194	1.34 q < 1	1.74 q < 0.0213
C12orf35	chromosome 12 open reading frame 35	Ssc.25441.2.S1_a_at	1.2 q < 0.00524	1.19 q < 0.901	-1.13 q < 0.849	1.54 q < 0.0089	1.24 q < 1	1.25 q < 0.669
SMYD2	SET and MYND domain containing 2	Ssc.13656.1.S1_at	1.18 q < 0.00526	-1.1 q < 1	1.02 q < 0.99	1.2 q < 0.383	1.41 q < 0.0727	1.47 q < 0.0131

Gene Name	Gene Description	Probe Set ID	Breed MS-WC	Day 25 MS-WC	Day 45 MS-WC	Day 65 MS-WC	Day 85 MS-WC	Day 105 MS-WC
ZNF662	zinc finger protein 662	Ssc.24347.1.S1_at	1.14 q < 0.00547	1.16 q < 0.787	1.08 q < 0.885	1.31 q < 0.0353	1.06 q < 1	1.13 q < 1
AQP5	aquaporin 5	Ssc.25999.1.S1_at	-1.3 q < 0.00548	-1.26 q < 0.986	-1.71 q < 0.0422	-1.02 q < 0.967	-1.47 q < 0.59	-1.15 q < 1
BLMH	bleomycin hydrolase	Ssc.11484.1.A1_at	-1.14 q < 0.00559	1.03 q < 1	-1.32 q < 0.0403	-1.04 q < 0.872	-1.21 q < 0.636	-1.22 q < 0.394
FGL1	fibrinogen-like 1	Ssc.20442.1.S1_at	1.34 q < 0.00571	-1.03 q < 1	1.26 q < 0.807	1.81 q < 0.034	1.49 q < 0.744	1.3 q < 1
KLHL13	kelch-like 13 (Drosophila)	Ssc.19801.1.S1_at	1.31 q < 0.00604	1.4 q < 0.579	1.06 q < 0.965	1.87 q < 0.0107	1.13 q < 1	1.22 q < 1
GOLPH3L	golgi phosphoprotein 3-like	Ssc.16613.1.A1_at	1.17 q < 0.00616	1.17 q < 0.872	1.12 q < 0.841	1.4 q < 0.0227	1.08 q < 1	1.1 q < 1
MMP16	matrix metallopeptidase 16 (membrane-inserted)	Ssc.13435.1.A1_at	-1.3 q < 0.00628	-2.38 q < 0.0000408	-1.2 q < 0.843	-1.13 q < 0.76	-1.01 q < 1	-1.12 q < 1
MMP16	matrix metallopeptidase 16 (membrane-inserted)	Ssc.30826.1.A1_at	-1.12 q < 0.0692	-1.56 q < 0.00025	-1 q < 1	-1.06 q < 0.796	-1.03 q < 1	-1.02 q < 1
VTI1A	vesicle transport through interaction with t-SNAREs homolog 1A (yeast)	Ssc.19072.1.A1_at	1.13 q < 0.00629	1.02 q < 1	1.06 q < 0.907	1.28 q < 0.0371	1.18 q < 0.751	1.12 q < 1
C19orf54	chromosome 19 open reading frame 54	Ssc.21879.1.S1_at	1.17 q < 0.00636	1.18 q < 0.8	-1.02 q < 0.983	1.49 q < 0.00267	1.1 q < 1	1.13 q < 1
FCN2	ficolin (collagen/fibrinogen domain containing lectin) 2 (hucolin)	Ssc.137.1.S1_at	-1.18 q < 0.00636	-1.01 q < 1	-1 q < 1	-1.09 q < 0.705	-1.15 q < 1	-1.77 q < 0.00000906
GJA1	gap junction protein, alpha 1, 43kDa	Ssc.15912.1.S1_at	-1.23 q < 0.00651	-1.87 q < 0.000448	-1.27 q < 0.546	-1.1 q < 0.767	1.03 q < 1	-1.13 q < 1
MCC	mutated in colorectal cancers	Ssc.18085.1.A1_at	1.23 q < 0.00657	1.12 q < 1	1.17 q < 0.817	1.58 q < 0.0154	1.12 q < 1	1.19 q < 1
PRKAA2	protein kinase, AMP-activated, alpha 2 catalytic subunit	Ssc.16257.1.S1_at	1.2 q < 0.00662	1.06 q < 1	1.15 q < 0.834	1.45 q < 0.0406	1.06 q < 1	1.34 q < 0.288
RCAN1	regulator of calcineurin 1	Ssc.3189.1.A1_at	1.32 q < 0.00683	1.07 q < 1	1.69 q < 0.0863	2.15 q < 0.00125	1.15 q < 1	-1.13 q < 1
RCAN1	regulator of calcineurin 1	Ssc.9607.1.A1_at	1.19 q < 0.0929	1.04 q < 1	1.27 q < 0.687	1.76 q < 0.0112	1.13 q < 1	-1.09 q < 1
TBC1D4	TBC1 domain family, member 4	Ssc.13357.1.A1_at	1.21 q < 0.00702	1.62 q < 0.00859	1.1 q < 0.913	1.18 q < 0.54	1.05 q < 1	1.2 q < 1
SPG20	spastic paraplegia 20 (Troyer syndrome)	Ssc.23990.1.A1_at	-1.21 q < 0.00708	-1.1 q < 1	-1.49 q < 0.0481	-1.01 q < 0.984	-1.24 q < 1	-1.3 q < 0.565
SPG20	spastic paraplegia 20 (Troyer syndrome)	Ssc.9916.1.S1_at	-1.23 q < 0.0109	-1.17 q < 1	-1.7 q < 0.00816	-1.03 q < 0.929	-1.25 q < 1	-1.08 q < 1
SPG20	spastic paraplegia 20 (Troyer syndrome)	Ssc.14083.1.A1_at	1.12 q < 0.354	-1.21 q < 1	1.02 q < 0.99	1.73 q < 0.00748	1.09 q < 1	1.13 q < 1
THNSL2	threonine synthase-like 2 (S. cerevisiae)	Ssc.4665.1.S1_at	1.11 q < 0.00711	1.11 q < 0.926	1.01 q < 0.987	1.24 q < 0.0433	1.09 q < 1	1.13 q < 0.854
PMP22	peripheral myelin protein 22	Ssc.11108.1.A1_at	-1.19 q < 0.00737	1.26 q < 0.526	-1.24 q < 0.489	-1.68 q < 0.00051	-1.18 q < 1	-1.24 q < 0.706
PMP22	peroxisomal membrane protein 2, 22kDa	Ssc.11108.1.A1_at	-1.19 q < 0.00737	1.26 q < 0.526	-1.24 q < 0.489	-1.68 q < 0.00051	-1.18 q < 1	-1.24 q < 0.706
NFIL3	nuclear factor, interleukin 3 regulated	Ssc.16882.1.A1_at	1.17 q < 0.00741	1.44 q < 0.0202	1.12 q < 0.841	1.19 q < 0.412	1.01 q < 1	1.14 q < 1
NFIL3	nuclear factor, interleukin 3 regulated	Ssc.29480.1.A1_at	1.11 q < 0.0337	1.03 q < 1	1.13 q < 0.726	1.24 q < 0.13	1.03 q < 1	1.17 q < 0.728
CD163	CD163 molecule	Ssc.11991.1.A1_at	-1.19 q < 0.00743	-1.21 q < 0.817	-1.07 q < 0.934	-1.05 q < 0.855	-1.16 q < 1	-1.53 q < 0.0116
CD163	CD163 molecule	Ssc.5053.1.S1_at	-1.26 q < 0.0741	-1.1 q < 1	-1.22 q < 0.876	-1.12 q < 0.817	-1.08 q < 1	-1.99 q < 0.0274
PCSK1	proprotein convertase subtilisin/kexin type 1	Ssc.141.1.S1_at	-1.43 q < 0.00748	1.04 q < 1	1 q < 0.999	-1.35 q < 0.547	-2.11 q < 0.0809	-2.18 q < 0.034
PDGFRL	platelet-derived growth factor receptor-like	Ssc.1161.1.A1_at	-1.4 q < 0.00757	-2.4 q < 0.00461	-1.38 q < 0.708	-1.15 q < 0.796	-1.06 q < 1	-1.34 q < 1
PDGFRL	platelet-derived growth factor receptor-like	Ssc.1161.2.S1_at	-1.14 q < 0.0994	-1.66 q < 0.00109	-1.15 q < 0.804	1.12 q < 0.647	-1.03 q < 1	-1.08 q < 1
LCP1	lymphocyte cytosolic protein 1 (L-plastin)	Ssc.9330.1.A1_at	-1.17 q < 0.00791	-1.11 q < 1	-1 q < 0.997	-1.1 q < 0.667	-1.15 q < 1	-1.52 q < 0.00243
LCP1	lymphocyte cytosolic protein 1 (L-plastin)	Ssc.23774.3.S1_at	-1.15 q < 0.0352	1 q < 1	-1.11 q < 0.881	-1.05 q < 0.843	-1.11 q < 1	-1.55 q < 0.00334
LCP1	TOX high mobility group box family member 4	Ssc.9330.1.A1_at	-1.17 q < 0.00791	-1.11 q < 1	-1 q < 0.997	-1.1 q < 0.667	-1.15 q < 1	-1.52 q < 0.00243
LCP1	TOX high mobility group box family member 4	Ssc.23774.3.S1_at	-1.15 q < 0.0352	1 q < 1	-1.11 q < 0.881	-1.05 q < 0.843	-1.11 q < 1	-1.55 q < 0.00334
CLIC5	chloride intracellular channel 5	Ssc.21139.2.S1_at	-1.23 q < 0.00798	-1.03 q < 1	-1.08 q < 0.94	-1.53 q < 0.0378	-1.38 q < 0.519	-1.19 q < 1
CLIC5	chloride intracellular channel 5	Ssc.21139.1.S1_at	-1.15 q < 0.00582	-1.01 q < 1	-1.03 q < 0.96	-1.26 q < 0.122	-1.23 q < 0.584	-1.24 q < 0.325
CLIC5	chloride intracellular channel 6	Ssc.21139.2.S1_at	-1.23 q < 0.00798	-1.03 q < 1	-1.08 q < 0.94	-1.53 q < 0.0378	-1.38 q < 0.519	-1.19 q < 1
CLIC5	chloride intracellular channel 6	Ssc.21139.1.S1_at	-1.15 q < 0.00582	-1.01 q < 1	-1.03 q < 0.96	-1.26 q < 0.122	-1.23 q < 0.584	-1.24 q < 0.325
SLC25A1	solute carrier family 25 (mitochondrial carrier; citrate transporter), member 1	Ssc.17264.1.S1_at	1.23 q < 0.00802	1.95 q < 0.000131	-1.25 q < 0.629	1.34 q < 0.244	1.25 q < 1	1.07 q < 1
OLFM3	olfactomedin 3	Ssc.8494.1.A1_at	-1.26 q < 0.0081	-1.67 q < 0.036	-1.14 q < 0.898	-1.27 q < 0.439	-1.08 q < 1	-1.21 q < 1
SEPHS2	selenophosphate synthetase 2	Ssc.15219.1.S1_at	-1.25 q < 0.00818	1.22 q < 1	-1.55 q < 0.0829	-1.16 q < 0.649	-1.23 q < 1	-1.7 q < 0.016
SEPHS2	selenophosphate synthetase 2	Ssc.4475.1.S1_at	-1.24 q < 0.028	1.21 q < 1	-1.52 q < 0.163	-1.08 q < 0.842	-1.33 q < 1	-1.61 q < 0.09
SS18L2	synovial sarcoma translocation gene on chromosome 18-like 2	Ssc.5794.1.S1_at	1.18 q < 0.00831	1.02 q < 1	1.05 q < 0.956	1.49 q < 0.0105	1.25 q < 0.884	1.15 q < 1
RHD	Rh blood group, D antigen	Ssc.14472.1.S1_at	1.17 q < 0.0084	1.8 q < 0.00000863	1.03 q < 0.976	1.09 q < 0.708	1.04 q < 1	1.06 q < 1
ROGDI	rogdi homolog (Drosophila)	Ssc.2167.3.A1_at	1.17 q < 0.00858	1.01 q < 1	1.18 q < 0.659	1.43 q < 0.0201	1.12 q < 1	1.16 q < 1
FER	fer (fps/fes related) tyrosine kinase	Ssc.12515.1.S1_at	-1.09 q < 0.00874	-1.01 q < 1	-1.18 q < 0.127	-1.02 q < 0.91	-1.03 q < 1	-1.26 q < 0.0069

Gene Name	Gene Description	Probe Set ID	Breed MS-WC	Day 25 MS-WC	Day 45 MS-WC	Day 65 MS-WC	Day 85 MS-WC	Day 105 MS-WC
PLA1A	phospholipase A1 member A	Ssc.11422.1.A1_at	-1.21 q < 0.00875	1.12 q < 1	-1.51 q < 0.0338	-1.34 q < 0.179	-1.34 q < 0.531	-1.05 q < 1
KDSR	3-ketodihydrosphingosine reductase	Ssc.18314.2.A1_at	1.26 q < 0.00878	1.04 q < 1	1.23 q < 0.762	1.95 q < 0.000797	1.05 q < 1	1.2 q < 1
NAGA	N-acetylgalactosaminidase, alpha-	Ssc.8873.1.A1_at	1.35 q < 0.00886	1.23 q < 1	1.39 q < 0.615	1.01 q < 0.983	1.25 q < 1	2.08 q < 0.013
BCAS2	breast carcinoma amplified sequence 2	Ssc.6999.1.S1_at	-1.15 q < 0.00896	-1.1 q < 1	-1.39 q < 0.0176	1.03 q < 0.91	-1.12 q < 1	-1.2 q < 0.608
PHLDA2	pleckstrin homology-like domain, family A, member 2	Ssc.9796.1.A1_at	1.23 q < 0.00896	1.16 q < 1	1.16 q < 0.845	1.73 q < 0.00313	1.13 q < 1	1.06 q < 1
KDELR2	KDEL (Lys-Asp-Glu-Leu) endoplasmic reticulum protein retention receptor 2	Ssc.997.3.A1_at	1.29 q < 0.009	1.28 q < 0.924	1.09 q < 0.946	1.74 q < 0.0262	1.37 q < 1	1.07 q < 1
RPL28	ribosomal protein L28	Ssc.1500.1.S1_a_at	-1.16 q < 0.00908	-1.04 q < 1	-1.21 q < 0.468	-1.12 q < 0.585	-1.03 q < 1	-1.43 q < 0.013
SLC35A3	solute carrier family 35 (UDP-N-acetylglucosamine (UDP-GlcNAc) transporter), member A3	Ssc.24284.1.S1_at	-1.32 q < 0.00924	-1.24 q < 1	-1.82 q < 0.0422	1.07 q < 0.888	-1.38 q < 1	-1.38 q < 0.844
SLC13A4	solute carrier family 13 (sodium/sulfate symporters), member 4	Ssc.5486.1.S1_at	1.55 q < 0.00932	1.58 q < 0.867	1.08 q < 0.98	2.92 q < 0.00968	1.09 q < 1	1.66 q < 0.826
SLC13A4	solute carrier family 13 (sodium/sulfate symporters), member 4	Ssc.5486.2.A1_at	1.4 q < 0.0304	1.62 q < 0.648	1.06 q < 0.982	2.07 q < 0.0758	1.03 q < 1	1.48 q < 0.998
ACSL6	acyl-CoA synthetase long-chain family member 6	Ssc.11255.1.A1_at	1.13 q < 0.00936	1.08 q < 1	1.09 q < 0.849	1.03 q < 0.896	-1.02 q < 1	1.55 q < 0.0000144
ACOT4	acyl-CoA thioesterase 4	Ssc.18475.3.A1_at	-1.26 q < 0.00937	-1.28 q < 0.837	-1.65 q < 0.0367	-1.15 q < 0.693	1.07 q < 1	-1.39 q < 0.491
NAP1L5	nucleosome assembly protein 1-like 5	Ssc.5726.1.S1_at	1.27 q < 0.0095	1.08 q < 1	1.02 q < 0.99	1.64 q < 0.0404	1.25 q < 1	1.45 q < 0.363
C10orf57	chromosome 10 open reading frame 57	Ssc.8441.1.A1_at	-1.25 q < 0.00962	1.06 q < 1	-1.79 q < 0.00629	-1.18 q < 0.617	-1.26 q < 1	-1.22 q < 1
C10orf57	chromosome 10 open reading frame 57	Ssc.8099.1.S1_at	1.6 q < 0.025	1.56 q < 1	1.85 q < 0.553	2.41 q < 0.135	1.03 q < 1	1.48 q < 1
NCOA1	nuclear receptor coactivator 1	Ssc.24299.1.S1_at	1.17 q < 0.00967	1.04 q < 1	1.04 q < 0.958	1.68 q < 0.000066	1.07 q < 1	1.12 q < 1
FAM12B	epididymal protein 3B	Ssc.350.1.S1_at	-1.29 q < 0.00971	1.08 q < 1	-1.94 q < 0.00703	-1.02 q < 0.976	-1.42 q < 0.906	-1.39 q < 0.663
NBEA	neurobeachin	Ssc.13921.1.S1_at	-1.27 q < 0.00998	-1.14 q < 1	-1.35 q < 0.5	-1.64 q < 0.0446	-1.12 q < 1	-1.17 q < 1
GGT6	gamma-glutamyltransferase 6	Ssc.25647.1.S1_at	1.29 q < 0.01	-1.08 q < 1	-1.28 q < 0.719	2.36 q < 0.0000664	1.38 q < 1	1.52 q < 0.308
NDUFA9	NADH dehydrogenase (ubiquinone) 1 alpha subcomplex, 9, 39kDa	Ssc.1239.1.A1_at	1.17 q < 0.0101	1.13 q < 1	1.06 q < 0.937	1.45 q < 0.0134	1.12 q < 1	1.12 q < 1
CTSS	cathepsin S	Ssc.17203.3.S1_at	-1.3 q < 0.0102	-1.15 q < 1	-1.18 q < 0.881	-1.06 q < 0.896	-1.16 q < 1	-2.25 q < 0.000598
CTSS	cathepsin S	Ssc.17203.1.A1_at	-1.34 q < 0.0396	-1.1 q < 1	-1.35 q < 0.769	-1.16 q < 0.782	-1.1 q < 1	-2.26 q < 0.0186
PITPNC1	phosphatidylinositol transfer protein, cytoplasmic 1	Ssc.21539.2.A1_at	1.19 q < 0.0103	1.11 q < 1	-1.01 q < 0.996	1.53 q < 0.0134	1.25 q < 1	1.16 q < 1
RPL36A	ribosomal protein L36a	Ssc.13585.7.S1_a_at	1.16 q < 0.0104	1.06 q < 1	1.14 q < 0.781	1.39 q < 0.0285	1.2 q < 1	1.04 q < 1
RPL36A	ribosomal protein L36a	Ssc.13585.8.S1_x_at	1.12 q < 0.0323	1.02 q < 1	1.13 q < 0.741	1.3 q < 0.0491	1.2 q < 0.821	-1.02 q < 1
RPL36A	ribosomal protein L36a-like	Ssc.13585.7.S1_a_at	1.16 q < 0.0104	1.06 q < 1	1.14 q < 0.781	1.39 q < 0.0285	1.2 q < 1	1.04 q < 1
RPL36A	ribosomal protein L36a-like	Ssc.13585.8.S1_x_at	1.12 q < 0.0323	1.02 q < 1	1.13 q < 0.741	1.3 q < 0.0491	1.2 q < 0.821	-1.02 q < 1
ZMYM3	zinc finger, MYM-type 3	Ssc.3316.1.A1_at	1.16 q < 0.0105	1.16 q < 0.927	-1.16 q < 0.713	1.43 q < 0.0156	1.25 q < 0.744	1.19 q < 0.832
CNTLN	centlein, centrosomal protein	Ssc.25950.1.S1_at	-1.11 q < 0.0106	-1.29 q < 0.0228	-1.05 q < 0.925	-1.04 q < 0.834	-1.11 q < 1	-1.09 q < 1
MSRB2	methionine sulfoxide reductase B2	Ssc.11012.1.A1_at	-1.16 q < 0.0107	-1.04 q < 1	-1.44 q < 0.0168	-1.15 q < 0.507	-1.13 q < 1	-1.09 q < 1
MPDU1	mannose-P-dolichol utilization defect 1	Ssc.1400.1.A1_at	1.17 q < 0.0109	1.28 q < 0.348	-1.03 q < 0.974	1.39 q < 0.0425	1.17 q < 1	1.08 q < 1
CXXC5	CXXC finger 5	Ssc.24142.1.S1_at	1.15 q < 0.0111	1.07 q < 1	1.09 q < 0.892	1.38 q < 0.0169	1.1 q < 1	1.13 q < 1
INPPL1	inositol polyphosphate phosphatase-like 1	Ssc.2346.1.S1_at	-1.1 q < 0.0111	-1.24 q < 0.0332	-1.08 q < 0.804	-1.1 q < 0.489	-1.08 q < 1	-1 q < 1
PCOLCE2	procollagen C-endopeptidase enhancer 2	Ssc.8758.1.A1_at	-1.32 q < 0.0112	-1.87 q < 0.0408	-1.57 q < 0.241	-1.29 q < 0.525	-1.16 q < 1	1.1 q < 1
PECI	peroxisomal D3,D2-enoyl-CoA isomerase	Ssc.10932.1.S1_at	-1.22 q < 0.0117	1.1 q < 1	-1.77 q < 0.00246	-1.26 q < 0.41	-1.23 q < 1	-1.1 q < 1
PABPC1	poly(A) binding protein, cytoplasmic 1	Ssc.1158.1.A1_at	-1.13 q < 0.0119	1.01 q < 1	-1.13 q < 0.67	-1.31 q < 0.0243	-1.07 q < 1	-1.16 q < 0.768
TMEM181	transmembrane protein 181	Ssc.24633.1.A1_at	1.18 q < 0.0119	1.05 q < 1	1.32 q < 0.22	1.5 q < 0.0111	1.04 q < 1	1.05 q < 1
EGR1	early growth response 1	Ssc.19629.1.A1_at	1.44 q < 0.012	1.32 q < 1	1.61 q < 0.475	2.53 q < 0.00714	-1.01 q < 1	1.15 q < 1
EGR1	early growth response 1	Ssc.16460.1.S1_at	-1.28 q < 0.00901	-1.63 q < 0.0912	-1.29 q < 0.659	1.04 q < 0.927	-1.29 q < 1	-1.31 q < 0.895
GPT2	glutamic pyruvate transaminase (alanine aminotransferase) 2	Ssc.7458.1.A1_at	-1.27 q < 0.0121	-2.41 q < 0.0000117	1.01 q < 0.997	-1.28 q < 0.468	-1.13 q < 1	1.06 q < 1
GPT2	glutamic pyruvate transaminase (alanine aminotransferase) 2	Ssc.27024.1.S1_at	-1.05 q < 0.799	-1.51 q < 0.0457	1.05 q < 0.962	1.07 q < 0.816	1 q < 1	1.04 q < 1
ABCG2	ATP-binding cassette, sub-family G (WHITE), member 2	Ssc.25103.1.S1_at	1.16 q < 0.0124	1.07 q < 1	1.44 q < 0.0163	1.13 q < 0.581	1.05 q < 1	1.14 q < 1
TMEM77	DNA-damage regulated autophagy modulator 2	Ssc.25450.1.S1_at	-1.14 q < 0.0125	-1.16 q < 0.796	-1.37 q < 0.0232	1.12 q < 0.551	-1.2 q < 0.935	-1.14 q < 1
CDC14A	CDC14 cell division cycle 14 homolog A (S. cerevisiae)	Ssc.13784.1.A1_at	1.12 q < 0.0126	-1.01 q < 1	1.1 q < 0.812	1.3 q < 0.0278	1.11 q < 1	1.14 q < 0.896

Gene Name	Gene Description	Probe Set ID	Breed MS-WC	Day 25 MS-WC	Day 45 MS-WC	Day 65 MS-WC	Day 85 MS-WC	Day 105 MS-WC
PDLIM5	PDZ and LIM domain 5	Ssc.29166.1.A1_at	1.15 q < 0.0128	1.05 q < 1	1.14 q < 0.752	1.35 q < 0.0473	1.22 q < 0.921	1.03 q < 1
PDLIM5	PDZ and LIM domain 5	Ssc.3783.1.S1_at	-1.25 q < 0.0149	-1.25 q < 0.927	-1.37 q < 0.403	-1.07 q < 0.868	-1.26 q < 1	-1.3 q < 0.862
CCND2	cyclin D2	Ssc.15749.1.S1_at	-1.24 q < 0.013	1.16 q < 1	-1.71 q < 0.0131	-1.4 q < 0.197	-1.22 q < 1	-1.16 q < 1
MTHFD1L	methylenetetrahydrofolate dehydrogenase (NADP+ dependent) 1-like	Ssc.9594.1.A1_at	-1.14 q < 0.0131	-1.38 q < 0.0309	-1.16 q < 0.65	-1.04 q < 0.86	-1.04 q < 1	-1.13 q < 1
DPP10	dipeptidyl-peptidase 10 (non-functional)	Ssc.13053.1.A1_at	1.19 q < 0.0132	1.1 q < 1	1.14 q < 0.843	1.68 q < 0.000966	1.44 q < 0.114	-1.28 q < 0.559
ORC3L	origin recognition complex, subunit 3-like (yeast)	Ssc.24532.1.S1_at	1.18 q < 0.0132	1.01 q < 1	1.04 q < 0.965	1.47 q < 0.0266	1.17 q < 1	1.29 q < 0.456
RPS27	ribosomal protein S27	Ssc.4805.1.S1_at	1.2 q < 0.0133	1.09 q < 1	-1.04 q < 0.977	1.48 q < 0.0433	1.17 q < 1	1.37 q < 0.269
C1orf77	chromosome 1 open reading frame 77	Ssc.7483.1.A1_at	-1.16 q < 0.0135	-1 q < 1	-1.5 q < 0.0053	-1.11 q < 0.655	-1.05 q < 1	-1.2 q < 0.767
JAK1	Janus kinase 1	Ssc.328.1.A1_at	-1.15 q < 0.0137	-1.16 q < 0.89	-1.39 q < 0.0304	-1.08 q < 0.734	-1.11 q < 1	-1.05 q < 1
SPG7	spastic paraplegia 7 (pure and complicated autosomal recessive)	Ssc.1399.1.S1_at	1.14 q < 0.0138	1 q < 1	-1.02 q < 0.987	1.39 q < 0.0111	1.17 q < 1	1.2 q < 0.6
PSMD9	proteasome (prosome, macropain) 26S subunit, non-ATPase, 9	Ssc.3258.1.S1_at	-1.13 q < 0.014	-1.16 q < 0.681	-1.33 q < 0.0211	-1.03 q < 0.878	-1.1 q < 1	-1.03 q < 1
CFP	complement factor properdin	Ssc.27474.1.S1_at	-1.17 q < 0.0141	-1.15 q < 1	-1.13 q < 0.846	-1.04 q < 0.898	-1.09 q < 1	-1.5 q < 0.0122
ATG4A	ATG4 autophagy related 4 homolog A (S. cerevisiae)	Ssc.9516.1.A1_at	1.17 q < 0.0142	1.06 q < 1	-1.01 q < 0.996	1.63 q < 0.000797	1.07 q < 1	1.21 q < 0.844
MAGEB3	melanoma antigen family B, 3	Ssc.25444.1.S1_at	1.15 q < 0.0145	-1.07 q < 1	1.16 q < 0.648	1.44 q < 0.005	1.13 q < 1	1.11 q < 1
MVP	major vault protein	Ssc.21214.1.A1_at	-1.15 q < 0.0149	-1.41 q < 0.0332	-1.08 q < 0.915	-1.02 q < 0.957	-1.14 q < 1	-1.17 q < 0.949
C7orf42	chromosome 7 open reading frame 42	Ssc.18341.1.A1_at	-1.13 q < 0.0151	-1.18 q < 0.64	-1.41 q < 0.00485	1.01 q < 0.955	-1.05 q < 1	-1.07 q < 1
TNFAIP1	tumor necrosis factor, alpha-induced protein 1 (endothelial)	Ssc.6549.1.S1_at	1.27 q < 0.0151	1.47 q < 0.36	-1.07 q < 0.958	1.28 q < 0.485	1.85 q < 0.0216	1.02 q < 1
ARID4B	AT rich interactive domain 4B (RBP1-like)	Ssc.2575.1.S1_at	1.1 q < 0.0158	1.03 q < 1	1.06 q < 0.901	1.14 q < 0.321	1.04 q < 1	1.25 q < 0.0317
LONP1	lon peptidase 1, mitochondrial	Ssc.1154.1.A1_at	-1.1 q < 0.0159	-1.05 q < 1	-1.23 q < 0.0469	-1.01 q < 0.979	-1.02 q < 1	-1.19 q < 0.155
IKBKG	inhibitor of kappa light polypeptide gene enhancer in B-cells, kinase gamma	Ssc.25274.1.A1_at	-1.13 q < 0.0161	1.02 q < 1	-1.36 q < 0.0175	1.05 q < 0.812	-1.2 q < 0.823	-1.21 q < 0.437
FAM81B	family with sequence similarity 81, member B	Ssc.30843.1.A1_at	-1.2 q < 0.0165	1.04 q < 1	1 q < 1	-1.25 q < 0.361	-1.02 q < 1	-1.99 q < 0.0000107
CD320	CD320 molecule	Ssc.12524.1.S1_at	1.17 q < 0.0167	1.15 q < 1	1.07 q < 0.936	-1.09 q < 0.732	1.13 q < 1	1.69 q < 0.000142
MARCKS	myristoylated alanine-rich protein kinase C substrate	Ssc.1228.1.S1_at	-1.19 q < 0.017	-1.06 q < 1	-1.55 q < 0.0188	-1.07 q < 0.827	1 q < 1	-1.36 q < 0.259
TMEM68	transmembrane protein 68	Ssc.6834.1.A1_at	1.21 q < 0.017	1.05 q < 1	1.08 q < 0.94	1.54 q < 0.0321	1.13 q < 1	1.31 q < 0.598
DLG5	discs, large homolog 5 (Drosophila)	Ssc.12059.1.A1_at	1.23 q < 0.0174	1.02 q < 1	1.08 q < 0.941	1.57 q < 0.0433	1.33 q < 1	1.21 q < 1
C8orf4	chromosome 8 open reading frame 4	Ssc.13156.2.S1_at	1.25 q < 0.0178	1.23 q < 1	1.26 q < 0.711	1.97 q < 0.00148	-1.06 q < 1	1.06 q < 1
OPRS1	sigma non-opioid intracellular receptor 1	Ssc.16410.1.A1_at	1.21 q < 0.0182	1.45 q < 0.151	-1.18 q < 0.802	1.54 q < 0.0308	1.25 q < 1	1.07 q < 1
CACNA1B	calcium channel, voltage-dependent, N type, alpha 1B subunit	Ssc.19324.2.S1_at	1.12 q < 0.0193	1.02 q < 1	1.11 q < 0.777	1.39 q < 0.00273	1.04 q < 1	1.08 q < 1
NF1	neurofibromin 1	Ssc.5446.1.S1_at	-1.12 q < 0.0194	-1.04 q < 1	-1.37 q < 0.00418	-1.02 q < 0.913	-1.11 q < 1	-1.07 q < 1
PTPN21	protein tyrosine phosphatase, non-receptor type 21	Ssc.4953.1.A1_at	-1.21 q < 0.0194	-1.05 q < 1	1.19 q < 0.792	-1.39 q < 0.163	-1.28 q < 1	-1.67 q < 0.0117
CYP19A1	cytochrome P450, family 19, subfamily A, polypeptide 1	Ssc.16184.1.S1_at	1.14 q < 0.0199	1.02 q < 1	1.27 q < 0.171	1.33 q < 0.0456	1.01 q < 1	1.09 q < 1
CYP19A1	cytochrome P450, family 19, subfamily A, polypeptide 1	Ssc.4548.1.A1_at	1.15 q < 0.41	1 q < 1	1.12 q < 0.934	1.8 q < 0.0324	-1.04 q < 1	1.02 q < 1
MYOZ1	myozenin 1	Ssc.10961.1.S1_at	1.1 q < 0.0199	1.02 q < 1	1.07 q < 0.878	1.25 q < 0.0317	1.12 q < 1	1.06 q < 1
FER1L3	myoferlin	Ssc.3692.1.A1_at	-1.21 q < 0.0199	-1.1 q < 1	-1.26 q < 0.617	-1.03 q < 0.932	-1.18 q < 1	-1.57 q < 0.0491
MGLL	monoglyceride lipase	Ssc.8436.1.S1_at	1.23 q < 0.02	-1.02 q < 1	-1.08 q < 0.944	2.28 q < 0.00000741	1.06 q < 1	1.29 q < 0.871
SREBF2	sterol regulatory element binding transcription factor 2	Ssc.16976.1.S1_at	1.18 q < 0.0208	1.2 q < 0.891	-1.13 q < 0.865	1.5 q < 0.0201	1.13 q < 1	1.26 q < 0.659
RPLP0P2	ribosomal protein, large, P0 pseudogene 2	Ssc.929.2.S1_at	-1.08 q < 0.0208	-1.01 q < 1	-1.17 q < 0.13	-1.1 q < 0.395	1.09 q < 1	-1.26 q < 0.00629
GRK7	G protein-coupled receptor kinase 7	Ssc.29960.1.A1_at	1.13 q < 0.021	1.01 q < 1	1.22 q < 0.31	1.4 q < 0.00617	1.02 q < 1	1.05 q < 1
FCGR3B	Fc fragment of IgG, low affinity IIIb, receptor (CD16b)	Ssc.167.2.S1_a_at	-1.26 q < 0.0218	1 q < 1	-1.09 q < 0.943	-1.18 q < 0.66	1.01 q < 1	-2.51 q < 0.00000952
C6orf106	chromosome 6 open reading frame 106	Ssc.4073.1.S1_at	1.1 q < 0.0226	1.07 q < 1	-1.04 q < 0.932	1.37 q < 0.000283	1.05 q < 1	1.08 q < 1
RPL13	ribosomal protein L13	Ssc.769.1.S1_a_at	-1.1 q < 0.0228	1.05 q < 1	-1.15 q < 0.443	-1.11 q < 0.475	-1.05 q < 1	-1.25 q < 0.0401
RPL13	mitochondrial ribosomal protein L13	Ssc.769.1.S1_a_at	-1.1 q < 0.0228	1.05 q < 1	-1.15 q < 0.443	-1.11 q < 0.475	-1.05 q < 1	-1.25 q < 0.0401
ECH1	enoyl CoA hydratase 1, peroxisomal	Ssc.5099.1.S1_at	1.16 q < 0.0228	1.07 q < 1	1 q < 0.997	1.41 q < 0.0336	1.3 q < 0.519	1.06 q < 1
HBD	hemoglobin, delta	Ssc.7157.1.A1_at	1.19 q < 0.0228	2.61 q < 0.0000000000471	-1.29 q < 0.436	-1.02 q < 0.967	1.03 q < 1	1.16 q < 1

Gene Name	Gene Description	Probe Set ID	Breed MS-WC	Day 25 MS-WC	Day 45 MS-WC	Day 65 MS-WC	Day 85 MS-WC	Day 105 MS-WC
HBD	keratin 121 pseudogene	Ssc.7157.1.A1_at	1.19 q < 0.0228	2.61 q < 0.0000000000471	-1.29 q < 0.436	-1.02 q < 0.967	1.03 q < 1	1.16 q < 1
HBD	hemoglobin, beta	Ssc.7157.1.A1_at	1.19 q < 0.0228	2.61 q < 0.0000000000471	-1.29 q < 0.436	-1.02 q < 0.967	1.03 q < 1	1.16 q < 1
SPR	sepiapterin reductase (7,8-dihydrobiopterin:NADP+ oxidoreductase)	Ssc.11252.1.S1_at	-1.24 q < 0.0239	1.27 q < 0.899	-1.28 q < 0.654	-1.09 q < 0.822	-1.4 q < 0.75	-1.89 q < 0.00412
SPR	tachykinin receptor 1	Ssc.11252.1.S1_at	-1.24 q < 0.0239	1.27 q < 0.899	-1.28 q < 0.654	-1.09 q < 0.822	-1.4 q < 0.75	-1.89 q < 0.00412
ZFY	zinc finger protein, Y-linked	Ssc.25078.1.S1_at	1.19 q < 0.0239	1.11 q < 1	1.03 q < 0.982	1.64 q < 0.00397	1.04 q < 1	1.21 q < 1
BRF1	BRF1 homolog, subunit of RNA polymerase III transcription initiation factor IIIB (S. cerevisiae)	Ssc.11687.1.A1_at	1.09 q < 0.0243	-1.01 q < 1	1.02 q < 0.975	1.31 q < 0.000995	1.05 q < 1	1.11 q < 0.925
BRF1	zinc finger protein 36, C3H type-like 1	Ssc.11687.1.A1_at	1.09 q < 0.0243	-1.01 q < 1	1.02 q < 0.975	1.31 q < 0.000995	1.05 q < 1	1.11 q < 0.925
GSTA1	glutathione S-transferase alpha 1	Ssc.8516.1.A1_at	-1.2 q < 0.0247	-1.04 q < 1	1.07 q < 0.946	-1.16 q < 0.624	-1.22 q < 1	-1.83 q < 0.000847
ADRB1	adrenergic, beta-1-, receptor	Ssc.16075.1.S1_at	1.13 q < 0.0249	-1.04 q < 1	-1.02 q < 0.982	1.23 q < 0.194	1.17 q < 1	1.36 q < 0.0322
MAGOHB	mago-nashi homolog B (Drosophila)	Ssc.21714.1.S1_at	1.11 q < 0.025	1.48 q < 0.0000358	1.02 q < 0.981	1.08 q < 0.666	1.01 q < 1	1.01 q < 1
MED19	mediator complex subunit 19	Ssc.26132.1.S1_at	1.13 q < 0.0251	1.01 q < 1	1.04 q < 0.946	1.42 q < 0.00411	1.13 q < 1	1.07 q < 1
CCL2	chemokine (C-C motif) ligand 2	Ssc.657.1.A1_at	-1.27 q < 0.0257	-1.09 q < 1	-1.04 q < 0.981	-1.03 q < 0.96	-1.02 q < 1	-2.76 q < 0.00000167
ACOT11	acyl-CoA thioesterase 11	Ssc.9743.1.A1_at	-1.26 q < 0.0259	-1.23 q < 1	-1.03 q < 0.988	1.07 q < 0.876	1.22 q < 1	-3.21 q < 0.00000000244
ZFX	zinc finger protein, X-linked	Ssc.15967.1.A1_at	1.12 q < 0.0277	1.05 q < 1	1.06 q < 0.918	1.31 q < 0.0456	1.04 q < 1	1.18 q < 0.681
BF711022	-	Ssc.9600.1.A1_at	1.14 q < 0.0278	1.07 q < 1	1.03 q < 0.976	1.35 q < 0.0472	1.08 q < 1	1.2 q < 0.742
VSIG4	V-set and immunoglobulin domain containing 4	Ssc.16640.1.A1_at	-1.22 q < 0.0278	-1.04 q < 1	-1.12 q < 0.911	-1.11 q < 0.769	-1.07 q < 1	-1.93 q < 0.000805
AMIGO2	adhesion molecule with Ig-like domain 2	Ssc.30916.1.A1_at	1.2 q < 0.0281	1.15 q < 1	-1 q < 0.997	1.02 q < 0.962	1.27 q < 1	1.68 q < 0.00838
PPP1R12A	protein phosphatase 1, regulatory (inhibitor) subunit 12A	Ssc.6525.2.A1_at	1.24 q < 0.0283	1.84 q < 0.00872	1.11 q < 0.928	1.18 q < 0.647	1.12 q < 1	1.08 q < 1
SLC25A38	solute carrier family 25, member 38	Ssc.6273.1.A1_at	-1.14 q < 0.0283	-1.15 q < 0.943	-1.42 q < 0.0125	1.11 q < 0.632	-1.05 q < 1	-1.22 q < 0.557
SLC25A43	solute carrier family 25, member 43	Ssc.12402.1.A1_at	1.2 q < 0.0283	1.03 q < 1	1.26 q < 0.622	1.54 q < 0.0463	1.2 q < 1	1.06 q < 1
CYP4A22	cytochrome P450, family 4, subfamily A, polypeptide 22	Ssc.190.1.S1_at	1.13 q < 0.0285	1.09 q < 1	1.09 q < 0.885	-1.02 q < 0.924	1.46 q < 0.00304	1.07 q < 1
PALLD	palladin, cytoskeletal associated protein	Ssc.7463.1.A1_at	-1.22 q < 0.0285	-1.02 q < 1	-1.51 q < 0.118	-1.67 q < 0.0153	-1.21 q < 1	1.17 q < 1
CPOX	coproporphyrinogen oxidase	Ssc.3633.1.S1_at	1.33 q < 0.0288	1.57 q < 0.503	1 q < 1	1.97 q < 0.0396	1.18 q < 1	1.15 q < 1
SKP2	S-phase kinase-associated protein 2 (p45)	Ssc.23085.1.S1_at	1.14 q < 0.0288	1.24 q < 0.437	-1.03 q < 0.977	1.56 q < 0.000444	1.02 q < 1	-1 q < 1
TMEM98	transmembrane protein 98	Ssc.11555.1.S1_at	1.19 q < 0.0291	2.01 q < 0.0000167	1.03 q < 0.981	1.07 q < 0.836	1.01 q < 1	1.05 q < 1
COL1A1	collagen, type I, alpha 1	Ssc.1091.1.S1_at	-1.52 q < 0.0294	-1.04 q < 1	-1.75 q < 0.556	1.22 q < 0.793	-1.39 q < 1	-3.96 q < 0.00125
COL1A1	collagen, type I, alpha 1	Ssc.1091.2.S1_at	-1.5 q < 0.0484	-1.28 q < 1	-2 q < 0.37	-1.03 q < 0.977	-1.09 q < 1	-2.63 q < 0.091
C11orf59	chromosome 11 open reading frame 59	Ssc.3302.1.S1_at	-1.14 q < 0.0295	-1.17 q < 0.843	-1.38 q < 0.0306	-1.02 q < 0.95	-1.04 q < 1	-1.11 q < 1
HLA-DMA	major histocompatibility complex, class II, DM alpha	Ssc.13777.1.S1_at	-1.14 q < 0.0304	1 q < 1	-1.03 q < 0.975	-1.07 q < 0.77	-1.04 q < 1	-1.71 q < 0.00002
SLC16A9	solute carrier family 16, member 9 (monocarboxylic acid transporter 9)	Ssc.3909.1.A1_at	1.24 q < 0.0307	1.1 q < 1	1.22 q < 0.808	-1.34 q < 0.371	1.28 q < 1	2.28 q < 0.0000573
SLC16A9	solute carrier family 16, member 9 (monocarboxylic acid transporter 9)	Ssc.24682.1.S1_at	1.04 q < 0.709	-1.04 q < 1	-1.02 q < 0.977	-1.02 q < 0.932	-1 q < 1	1.31 q < 0.0243
TMEM87A	transmembrane protein 87A	Ssc.1852.1.S1_at	1.14 q < 0.0308	1.14 q < 1	-1.08 q < 0.911	1.21 q < 0.344	1.11 q < 1	1.39 q < 0.0491
MGST2	microsomal glutathione S-transferase 2	Ssc.21635.1.A1_at	1.28 q < 0.0308	1.19 q < 1	1.01 q < 0.996	2.06 q < 0.00582	1.29 q < 1	1.08 q < 1
PFN1	profilin 1	Ssc.835.1.S1_at	-1.28 q < 0.0316	1.03 q < 1	-1.73 q < 0.0948	-1.63 q < 0.125	1.5 q < 0.747	-1.9 q < 0.0288
LBR	lamin B receptor	Ssc.22874.1.S1_at	-1.18 q < 0.032	1.15 q < 1	-1.66 q < 0.00462	1.14 q < 0.637	-1.31 q < 0.813	-1.4 q < 0.202
IRF5	interferon regulatory factor 5	Ssc.5480.1.S1_at	-1.1 q < 0.0325	-1.01 q < 1	-1.11 q < 0.724	1.04 q < 0.843	1.01 q < 1	-1.49 q < 0.0000051
PTBP2	polypyrimidine tract binding protein 2	Ssc.18855.1.A1_at	-1.13 q < 0.0329	1.2 q < 0.542	-1.5 q < 0.000875	-1.15 q < 0.45	-1.09 q < 1	-1.15 q < 0.956
ETS2	v-ets erythroblastosis virus E26 oncogene homolog 2 (avian)	Ssc.18456.2.S1_at	1.17 q < 0.0331	1.19 q < 0.95	-1.05 q < 0.958	1.53 q < 0.0169	1.17 q < 1	1.09 q < 1
ETS2	v-ets erythroblastosis virus E26 oncogene homolog 2 (avian)	Ssc.6163.1.A1_at	1.15 q < 0.148	1.07 q < 1	1.05 q < 0.959	1.65 q < 0.00888	1.02 q < 1	1.05 q < 1
CDK2	cyclin-dependent kinase 2	Ssc.16532.1.S1_at	1.11 q < 0.0337	1.15 q < 0.787	-1.05 q < 0.93	1.34 q < 0.0137	1.13 q < 1	1.04 q < 1
LAMB2	laminin, beta 2 (laminin S)	Ssc.5067.1.S1_at	-1.15 q < 0.0337	-1.1 q < 1	-1.45 q < 0.0295	-1.1 q < 0.706	-1.17 q < 1	1 q < 1
LAMB2	laminin, gamma 1 (formerly LAMB2)	Ssc.5067.1.S1_at	-1.15 q < 0.0337	-1.1 q < 1	-1.45 q < 0.0295	-1.1 q < 0.706	-1.17 q < 1	1 q < 1
DNMT1	DNA (cytosine-5-)-methyltransferase 1	Ssc.28025.1.A1_at	1.12 q < 0.034	1.05 q < 1	1.13 q < 0.701	1.3 q < 0.045	1.1 q < 1	1.03 q < 1

Gene Name	Gene Description	Probe Set ID	Breed MS-WC	Day 25 MS-WC	Day 45 MS-WC	Day 65 MS-WC	Day 85 MS-WC	Day 105 MS-WC
GSTO1	glutathione S-transferase omega 1	Ssc.183.1.S1_at	-1.19 q < 0.034	1.48 q < 0.117	-1.13 q < 0.895	-1.51 q < 0.0476	-1.37 q < 0.574	-1.53 q < 0.0585
PCBD2	pterin-4 alpha-carbinolamine dehydratase/dimerization cofactor of hepatocyte nuclear factor 1 alpha (TCF1) 2	Ssc.6520.1.A1_at	1.15 q < 0.0354	1.47 q < 0.0152	-1.05 q < 0.952	1.11 q < 0.666	1.2 q < 1	1.06 q < 1
STIM1	stromal interaction molecule 1	Ssc.23482.1.S1_at	1.16 q < 0.0355	-1.02 q < 1	1.02 q < 0.989	1.51 q < 0.00896	1.24 q < 1	1.1 q < 1
REM1	RAS (RAD and GEM)-like GTP-binding 1	Ssc.17853.1.A1_at	-1.14 q < 0.0356	-1.73 q < 0.0000216	-1.25 q < 0.36	1.01 q < 0.965	-1.07 q < 1	1.17 q < 1
BI400739	-	Ssc.10356.1.A1_at	1.13 q < 0.0357	-1.03 q < 1	1.21 q < 0.447	1.36 q < 0.0391	1.05 q < 1	1.11 q < 1
IGF2	insulin-like growth factor 2 (somatomedin A)	Ssc.9365.6.S1_x_at	-1.12 q < 0.0366	-1.36 q < 0.0365	-1.26 q < 0.181	1.04 q < 0.855	-1.02 q < 1	-1.07 q < 1
IGF2	insulin-like growth factor 2 (somatomedin A)	Ssc.9365.1.S1_at	-1.05 q < 0.751	-1.25 q < 0.547	-1.21 q < 0.607	1.56 q < 0.00413	-1.3 q < 0.554	-1.02 q < 1
STC1	stanniocalcin 1	Ssc.2464.1.S1_at	1.12 q < 0.0386	1.05 q < 1	-1.15 q < 0.663	1.05 q < 0.803	1.1 q < 1	1.66 q < 0.00000194
PTK2	PTK2 protein tyrosine kinase 2	Ssc.9581.1.A1_at	1.1 q < 0.0392	-1 q < 1	1.02 q < 0.976	1.31 q < 0.01	1.06 q < 1	1.13 q < 0.867
SPARC	secreted protein, acidic, cysteine-rich (osteonectin)	Ssc.1458.3.A1_a_at	-1.13 q < 0.0392	-1.02 q < 1	-1.51 q < 0.000939	-1.07 q < 0.764	-1.07 q < 1	-1.03 q < 1
INSIG1	insulin induced gene 1	Ssc.15598.1.S1_at	1.23 q < 0.0394	1.34 q < 0.723	-1.11 q < 0.929	1.67 q < 0.0406	1.2 q < 1	1.18 q < 1
CMTM4	CKLF-like MARVEL transmembrane domain containing 4	Ssc.19203.2.S1_at	1.16 q < 0.0403	-1.02 q < 1	-1.03 q < 0.982	1.51 q < 0.0119	1.21 q < 1	1.19 q < 1
NPDC1	neural proliferation, differentiation and control, 1	Ssc.21971.2.S1_at	-1.13 q < 0.0409	-1.03 q < 1	-1.39 q < 0.0178	-1.12 q < 0.569	-1.27 q < 0.396	1.13 q < 1
ZC3H13	zinc finger CCCH-type containing 13	Ssc.10054.1.S1_at	-1.11 q < 0.0429	1.03 q < 1	-1.31 q < 0.0416	-1.05 q < 0.803	-1.09 q < 1	-1.17 q < 0.685
UBA5	ubiquitin-like modifier activating enzyme 5	Ssc.25021.1.S1_at	1.12 q < 0.0437	1.14 q < 0.984	1.05 q < 0.946	1.35 q < 0.032	1.02 q < 1	1.1 q < 1
BLVRB	biliverdin reductase B (flavin reductase (NADPH))	Ssc.1338.1.S1_at	1.21 q < 0.0438	1.98 q < 0.000598	-1.02 q < 0.991	1.14 q < 0.7	-1 q < 1	1.17 q < 1
UCP2	uncoupling protein 2 (mitochondrial, proton carrier)	Ssc.16350.1.S1_at	-1.12 q < 0.0456	-1.11 q < 1	-1.02 q < 0.982	1.05 q < 0.83	-1.14 q < 1	-1.4 q < 0.0067
CSRP2	cysteine and glycine-rich protein 2	Ssc.11530.1.S1_at	1.22 q < 0.0457	1.16 q < 1	1.07 q < 0.958	2.08 q < 0.000325	1.05 q < 1	-1 q < 1
PROM2	prominin 2	Ssc.27157.2.S1_at	1.26 q < 0.0471	1.09 q < 1	-1.11 q < 0.94	1.81 q < 0.0371	1.26 q < 1	1.45 q < 0.639
GULP1	GULP, engulfment adaptor PTB domain containing 1	Ssc.19175.3.A1_at	1.17 q < 0.0481	1.03 q < 1	1.08 q < 0.939	1.52 q < 0.0316	1.11 q < 1	1.19 q < 1
TTC3	tetratricopeptide repeat domain 3	Ssc.27325.1.S1_at	-1.1 q < 0.0484	-1.33 q < 0.0157	-1.11 q < 0.749	-1.18 q < 0.251	1.01 q < 1	1.06 q < 1
SPEF1	sperm flagellar 1	Ssc.30895.2.A1_at	-1.1 q < 0.0498	-1.3 q < 0.0386	-1 q < 0.997	-1.1 q < 0.555	-1.04 q < 1	-1.08 q < 1
ABCC5	ATP-binding cassette, sub-family C (CFTR/MRP), member 5	Ssc.1657.1.S1_at	1.18 q < 0.167	-1.13 q < 1	-1.29 q < 0.67	1.72 q < 0.0324	1.14 q < 1	1.72 q < 0.0463
ABCC5	ATP-binding cassette, sub-family C (CFTR/MRP), member 5	Ssc.6425.1.A1_at	1.2 q < 0.124	-1.1 q < 1	-1.15 q < 0.91	1.6 q < 0.0931	1.09 q < 1	1.79 q < 0.0285
Q8WUC5	-	Ssc.11616.1.S1_at	-1.11 q < 0.285	-1.02 q < 1	-1.55 q < 0.0125	1.55 q < 0.00954	-1.35 q < 0.42	-1.21 q < 0.937
VAV3	vav 3 guanine nucleotide exchange factor	Ssc.12682.1.A1_at	-1.07 q < 0.572	-1.49 q < 0.00959	-1.44 q < 0.0254	1.05 q < 0.851	1.22 q < 1	1.21 q < 0.769
ERAF	alpha hemoglobin stabilizing protein	Ssc.1354.1.S1_at	1.07 q < 0.809	1.75 q < 0.0431	-1.79 q < 0.0232	-1.12 q < 0.771	1.23 q < 1	1.29 q < 1
ARHGAP12	Rho GTPase activating protein 12	Ssc.19040.1.A1_at	-1.02 q < 0.899	-1.07 q < 1	-1.14 q < 0.529	-1.43 q < 0.0000664	1.23 q < 0.204	1.31 q < 0.0108
ARHGAP12	Rho GTPase activating protein 12	Ssc.11354.1.A1_at	-1.21 q < 0.0242	-1.33 q < 0.539	-1.19 q < 0.812	-1.13 q < 0.694	-1.16 q < 1	-1.25 q < 0.931
NPL	N-acetylneuraminate pyruvate lyase (dihydrodipicolinate synthase)	Ssc.23640.1.S1_at	-1.01 q < 0.96	-1 q < 1	-1.09 q < 0.885	-1.35 q < 0.0321	-1.04 q < 1	1.46 q < 0.00365
FAM162B	family with sequence similarity 162, member B	Ssc.6162.2.S1_at	-1.01 q < 0.965	1.06 q < 1	-1.52 q < 0.0227	1.04 q < 0.902	-1.18 q < 1	1.52 q < 0.0243
FAM162B	family with sequence similarity 162, member B	Ssc.6162.1.A1_at	1.04 q < 0.844	1.06 q < 1	-1.41 q < 0.0703	1.15 q < 0.579	-1.15 q < 1	1.62 q < 0.00184
ASB11	ankyrin repeat and SOCS box-containing 11	Ssc.12849.1.A1_at	1.02 q < 0.97	-1.07 q < 1	-3.47 q < 0.0000706	2.92 q < 0.000985	1.02 q < 1	1.4 q < 1
TMEM16C	anoctamin 3	Ssc.8291.1.A1_at	-1.01 q < 0.978	-1.51 q < 0.00393	-1.06 q < 0.94	-1.02 q < 0.958	-1.04 q < 1	1.62 q < 0.000257
CNIH4	cornichon homolog 4 (Drosophila)	Ssc.11342.1.A1_at	-1.38 q < 0.000000274	-1.43 q < 0.117	-1.33 q < 0.267	-1.28 q < 0.288	-1.51 q < 0.0521	-1.33 q < 0.367
DSCR3	Down syndrome critical region gene 3	Ssc.5989.1.S1_at	-1.25 q < 0.000000632	-1.2 q < 0.512	-1.32 q < 0.0551	-1.27 q < 0.0959	-1.22 q < 0.636	-1.24 q < 0.328
PTGES3	prostaglandin E synthase 3 (cytosolic)	Ssc.25850.1.A1_at	2.1 q < 0.000000748	2.09 q < 0.265	2.37 q < 0.0932	2.07 q < 0.152	2.17 q < 0.356	1.85 q < 0.56
STAG2	stromal antigen 2	Ssc.30423.1.A1_at	-1.32 q < 0.00000133	-1.28 q < 0.425	-1.39 q < 0.0932	-1.3 q < 0.177	-1.39 q < 0.17	-1.24 q < 0.709
TMEM69	transmembrane protein 69	Ssc.9306.1.A1_at	1.45 q < 0.00000296	1.53 q < 0.159	1.28 q < 0.646	1.51 q < 0.11	1.54 q < 0.229	1.39 q < 0.505
TOR1B	torsin family 1, member B (torsin B)	Ssc.6881.1.A1_s_at	-1.2 q < 0.00000336	-1.26 q < 0.108	-1.23 q < 0.147	-1.08 q < 0.654	-1.25 q < 0.2	-1.21 q < 0.325
DSCAM	Down syndrome cell adhesion molecule	Ssc.4500.1.S1_at	-1.21 q < 0.00000423	-1.29 q < 0.0691	-1.18 q < 0.434	-1.19 q < 0.237	-1.17 q < 1	-1.24 q < 0.228
NAV1	neuron navigator 1	Ssc.4867.1.S1_at	-1.29 q < 0.00000444	-1.18 q < 0.91	-1.31 q < 0.232	-1.37 q < 0.0663	-1.34 q < 0.311	-1.28 q < 0.434
LYSMD3	LysM, putative peptidoglycan-binding, domain containing 3	Ssc.10088.1.A1_at	-1.32 q < 0.00000843	-1.45 q < 0.0752	-1.21 q < 0.648	-1.16 q < 0.581	-1.49 q < 0.0593	-1.31 q < 0.416
CDC5L	CDC5 cell division cycle 5-like (S. pombe)	Ssc.19068.1.A1_at	1.29 q < 0.00000918	1.33 q < 0.257	1.22 q < 0.545	1.41 q < 0.0519	1.33 q < 0.409	1.19 q < 0.971
ZDHHC7	zinc finger, DHHC-type containing 7	Ssc.3617.2.S1_at	-1.25 q < 0.000013	-1.22 q < 0.539	-1.23 q < 0.372	-1.21 q < 0.303	-1.31 q < 0.281	-1.27 q < 0.313
EPYC	epiphycan	Ssc.9725.1.A1_at	-1.4 q < 0.0000169	-1.09 q < 1	-1.57 q < 0.0726	-1.37 q < 0.265	-1.48 q < 0.358	-1.53 q < 0.126

Gene Name	Gene Description	Probe Set ID	Breed MS-WC	Day 25 MS-WC	Day 45 MS-WC	Day 65 MS-WC	Day 85 MS-WC	Day 105 MS-WC
C18orf19	chromosome 18 open reading frame 19	Ssc.3529.1.A1_at	-1.19 q < 0.0000208	1.01 q < 1	-1.26 q < 0.0745	-1.23 q < 0.103	-1.25 q < 0.185	-1.22 q < 0.264
UBE2G2	ubiquitin-conjugating enzyme E2G 2 (UBC7 homolog, yeast)	Ssc.5115.1.S1_at	-1.24 q < 0.0000216	-1.2 q < 0.664	-1.34 q < 0.0722	-1.16 q < 0.454	-1.26 q < 0.542	-1.24 q < 0.453
RRP12	ribosomal RNA processing 12 homolog (S. cerevisiae)	Ssc.24912.1.S1_at	-1.24 q < 0.0000244	-1.17 q < 0.837	-1.33 q < 0.0932	-1.33 q < 0.0714	-1.29 q < 0.366	-1.1 q < 1
PTK7	PTK7 protein tyrosine kinase 7	Ssc.28680.1.S1_at	-1.26 q < 0.0000249	-1.41 q < 0.0526	-1.35 q < 0.11	-1.18 q < 0.468	-1.17 q < 1	-1.22 q < 0.717
PLAA	phospholipase A2-activating protein	Ssc.16422.2.A1_at	-1.22 q < 0.0000348	-1.32 q < 0.0784	-1.28 q < 0.142	-1.11 q < 0.612	-1.36 q < 0.0587	-1.05 q < 1
MACF1	microtubule-actin crosslinking factor 1	Ssc.5639.2.S1_at	-1.22 q < 0.0000377	-1.22 q < 0.486	-1.3 q < 0.124	-1.19 q < 0.353	-1.24 q < 0.604	-1.18 q < 0.848
SLC30A9	solute carrier family 30 (zinc transporter), member 9	Ssc.7040.2.A1_at	-1.29 q < 0.0000439	-1.37 q < 0.189	-1.4 q < 0.105	-1.17 q < 0.524	-1.31 q < 0.621	-1.19 q < 1
COMMD7	COMM domain containing 7	Ssc.11716.1.S1_a_at	1.32 q < 0.0000483	1.44 q < 0.161	1.21 q < 0.712	1.29 q < 0.317	1.27 q < 1	1.42 q < 0.196
EXDL1	exonuclease 3'-5' domain containing 1	Ssc.10798.1.A1_at	-1.18 q < 0.0000503	-1.04 q < 1	-1.22 q < 0.184	-1.18 q < 0.255	-1.28 q < 0.079	-1.18 q < 0.513
HTR3B	5-hydroxytryptamine (serotonin) receptor 3B	Ssc.26752.1.S1_at	-1.21 q < 0.0000503	-1.1 q < 1	-1.31 q < 0.0796	-1.3 q < 0.072	-1.23 q < 0.679	-1.14 q < 1
LFNG	LFNG O-fucosylpeptide 3-beta-N-acetylglucosaminyltransferase	Ssc.19531.1.A1_at	-1.22 q < 0.0000616	-1.23 q < 0.432	-1.2 q < 0.457	-1.12 q < 0.563	-1.27 q < 0.366	-1.26 q < 0.277
ZCCHC24	zinc finger, CCHC domain containing 24	Ssc.12014.2.A1_at	-1.25 q < 0.000062	-1.39 q < 0.0766	-1.29 q < 0.243	-1.31 q < 0.136	-1.14 q < 1	-1.14 q < 1
ZCCHC24	zinc finger, CCHC domain containing 24	Ssc.16810.1.S1_at	-1.23 q < 0.00246	-1.16 q < 1	-1.4 q < 0.127	-1.27 q < 0.304	-1.14 q < 1	-1.19 q < 1
EGFR	epidermal growth factor receptor	Ssc.55.1.S1_at	-1.37 q < 0.0000817	-1.59 q < 0.0794	-1.38 q < 0.372	-1.18 q < 0.612	-1.37 q < 0.848	-1.35 q < 0.646
ACACA	acetyl-CoA carboxylase alpha	Ssc.17769.1.S1_at	1.22 q < 0.0000838	1.16 q < 0.855	1.06 q < 0.93	1.32 q < 0.0634	1.26 q < 0.498	1.3 q < 0.152
FAU	Finkel-Biskis-Reilly murine sarcoma virus (FBR-MuSV) ubiquitously expressed	Ssc.11087.1.S1_at	-1.15 q < 0.0000838	-1.01 q < 1	-1.24 q < 0.051	-1.19 q < 0.129	-1.1 q < 1	-1.23 q < 0.0899
AIFM2	apoptosis-inducing factor, mitochondrion-associated, 2	Ssc.2487.1.S1_at	1.24 q < 0.0000866	1 q < 1	1.23 q < 0.439	1.32 q < 0.111	1.42 q < 0.0556	1.27 q < 0.39
AIFM2	apoptosis-inducing factor, mitochondrion-associated, 2	Ssc.2487.2.S1_at	1.2 q < 0.00858	1.01 q < 1	1.24 q < 0.527	1.31 q < 0.196	1.35 q < 0.415	1.1 q < 1
RNF12	ring finger protein, LIM domain interacting	Ssc.9094.1.A1_at	-1.23 q < 0.0000892	-1.21 q < 0.671	-1.23 q < 0.429	-1.22 q < 0.317	-1.28 q < 0.472	-1.23 q < 0.587
RGS2	regulator of G-protein signaling 2, 24kDa	Ssc.3139.1.A1_at	-1.46 q < 0.0001	-1.67 q < 0.153	-1.3 q < 0.73	-1.21 q < 0.644	-1.4 q < 1	-1.82 q < 0.0502
CYP51A1	cytochrome P450, family 51, subfamily A, polypeptide 1	Ssc.5712.1.S1_at	1.38 q < 0.000109	1.51 q < 0.217	1.09 q < 0.942	1.52 q < 0.115	1.4 q < 0.774	1.44 q < 0.394
HOXB6	homeobox B6	Ssc.13232.1.A1_at	1.21 q < 0.000118	1.24 q < 0.41	1.09 q < 0.884	1.28 q < 0.111	1.24 q < 0.664	1.22 q < 0.533
OLFML3	olfactomedin-like 3	Ssc.15592.1.S1_at	-1.36 q < 0.00012	-1.61 q < 0.0712	-1.39 q < 0.36	-1.55 q < 0.0706	-1.07 q < 1	-1.27 q < 0.971
BAG3	BCL2-associated athanogene 3	Ssc.13649.1.S1_at	-1.35 q < 0.000126	-1.28 q < 0.819	-1.52 q < 0.11	-1.32 q < 0.344	-1.45 q < 0.444	-1.22 q < 1
Q9H3B5	-	Ssc.16863.1.S1_at	-1.22 q < 0.000136	-1.18 q < 0.756	-1.32 q < 0.101	-1.19 q < 0.381	-1.19 q < 1	-1.2 q < 0.698
C6orf98	spectrin repeat containing, nuclear envelope 1	Ssc.6365.1.A1_at	-1.24 q < 0.000141	-1.15 q < 1	-1.3 q < 0.208	-1.37 q < 0.0527	-1.39 q < 0.0955	-1.01 q < 1
NASP	nuclear autoantigenic sperm protein (histone-binding)	Ssc.7153.1.A1_at	-1.19 q < 0.000144	-1.07 q < 1	-1.3 q < 0.0681	-1.17 q < 0.344	-1.19 q < 0.899	-1.22 q < 0.402
NASP	nuclear autoantigenic sperm protein (histone-binding)	Ssc.25115.1.A1_at	1.13 q < 0.0765	1.47 q < 0.0162	-1.01 q < 0.996	1.1 q < 0.691	1.1 q < 1	1.05 q < 1
ANKFY1	ankyrin repeat and FYVE domain containing 1	Ssc.5685.1.S1_at	-1.2 q < 0.00015	-1.15 q < 0.901	-1.32 q < 0.0714	-1.13 q < 0.545	-1.19 q < 1	-1.23 q < 0.401
SLC25A33	solute carrier family 25, member 33	Ssc.23233.1.S1_a_at	1.27 q < 0.00015	1.24 q < 0.727	1.24 q < 0.556	1.33 q < 0.176	1.3 q < 0.73	1.26 q < 0.665
RFK	riboflavin kinase	Ssc.7099.2.A1_a_at	1.31 q < 0.000156	-1.02 q < 1	1.5 q < 0.0709	1.49 q < 0.0615	1.41 q < 0.396	1.25 q < 0.917
RFK	riboflavin kinase	Ssc.7099.1.S1_at	1.21 q < 0.0269	1.06 q < 1	1.14 q < 0.891	1.37 q < 0.225	1.28 q < 1	1.25 q < 0.998
TSC1	tuberous sclerosis 1	Ssc.30016.3.A1_at	1.17 q < 0.000181	1.19 q < 0.489	1.16 q < 0.515	1.22 q < 0.151	1.13 q < 1	1.18 q < 0.621
SFXN4	sideroflexin 4	Ssc.3798.1.A1_at	-1.22 q < 0.00019	-1.13 q < 1	-1.23 q < 0.443	-1.35 q < 0.0599	-1.22 q < 1	-1.21 q < 0.717
ZFP64	zinc finger protein 64 homolog (mouse)	Ssc.23811.2.S1_at	1.19 q < 0.00019	1.16 q < 0.812	1.25 q < 0.187	1.18 q < 0.343	1.08 q < 1	1.3 q < 0.0998
MEIS2	Meis homeobox 2	Ssc.23820.1.A1_a_at	-1.17 q < 0.0002	-1.24 q < 0.188	-1.17 q < 0.484	-1.17 q < 0.317	-1.25 q < 0.252	-1.05 q < 1
MEIS2	Meis homeobox 2	Ssc.10110.1.A1_at	-1.19 q < 0.012	-1.05 q < 1	-1.35 q < 0.196	-1.36 q < 0.125	-1.18 q < 1	-1.05 q < 1
WDR68	DDB1 and CUL4 associated factor 7	Ssc.24219.1.S1_at	-1.22 q < 0.000203	-1.07 q < 1	-1.37 q < 0.0522	-1.19 q < 0.384	-1.28 q < 0.434	-1.2 q < 0.771
PTPMT1	protein tyrosine phosphatase, mitochondrial 1	Ssc.9174.2.A1_a_at	1.26 q < 0.000207	1.19 q < 0.906	1.37 q < 0.143	1.37 q < 0.0989	1.28 q < 0.844	1.11 q < 1
COL4A2	collagen, type IV, alpha 2	Ssc.9939.1.A1_at	-1.66 q < 0.000208	-1.65 q < 0.605	-1.98 q < 0.155	-1.31 q < 0.639	-1.77 q < 0.696	-1.68 q < 0.613
ABCF1	ATP-binding cassette, sub-family F (GCN20), member 1	Ssc.1504.2.S1_at	-1.23 q < 0.000241	-1.19 q < 0.848	-1.34 q < 0.134	-1.17 q < 0.497	-1.23 q < 1	-1.25 q < 0.544
SIPA1L1	signal-induced proliferation-associated 1 like 1	Ssc.24345.1.S1_at	1.23 q < 0.000244	1.2 q < 0.787	1.14 q < 0.806	1.32 q < 0.126	1.32 q < 0.354	1.19 q < 0.901
PKP2	plakophilin 2	Ssc.7352.1.S1_at	-1.25 q < 0.000245	-1.29 q < 0.382	-1.19 q < 0.68	-1.2 q < 0.418	-1.32 q < 0.454	-1.24 q < 0.728
PKP2	plakophilin 2	Ssc.10743.1.A1_at	-1.33 q < 0.00275	-1.13 q < 1	-1.14 q < 0.913	-1.55 q < 0.133	-1.38 q < 1	-1.55 q < 0.257
MORN4	MORN repeat containing 4	Ssc.2958.1.S1_at	-1.17 q < 0.000247	-1.11 q < 1	-1.21 q < 0.26	-1.19 q < 0.249	-1.25 q < 0.216	-1.09 q < 1
DIAPH2	diaphanous homolog 2 (Drosophila)	Ssc.14280.1.S1_at	-1.2 q < 0.000255	-1.23 q < 0.458	-1.3 q < 0.132	-1.14 q < 0.521	-1.28 q < 0.351	-1.09 q < 1
DIAPH2	diaphanous homolog 2 (Drosophila)	Ssc.24473.1.A1_at	-1.11 q < 0.0438	-1.14 q < 0.901	-1.15 q < 0.623	-1.15 q < 0.432	-1.07 q < 1	-1.07 q < 1

Gene Name	Gene Description	Probe Set ID	Breed MS-WC	Day 25 MS-WC	Day 45 MS-WC	Day 65 MS-WC	Day 85 MS-WC	Day 105 MS-WC
MAPT	microtubule-associated protein tau	Ssc.4282.1.S1_at	-1.33 q < 0.000261	-1.54 q < 0.104	-1.48 q < 0.144	-1.13 q < 0.704	-1.18 q < 1	-1.35 q < 0.56
ICOSLG	inducible T-cell co-stimulator ligand	Ssc.11718.1.S1_at	-1.31 q < 0.000262	-1.07 q < 1	-1.54 q < 0.0588	-1.34 q < 0.253	-1.32 q < 0.927	-1.32 q < 0.596
KIF21B	kinesin family member 21B	Ssc.8424.1.A1_at	-1.27 q < 0.000284	-1.39 q < 0.18	-1.15 q < 0.829	-1.32 q < 0.199	-1.18 q < 1	-1.32 q < 0.434
SEC16B	SEC16 homolog B (S. cerevisiae)	Ssc.2554.1.S1_at	-1.31 q < 0.000284	-1.32 q < 0.547	-1.28 q < 0.552	-1.3 q < 0.334	-1.21 q < 1	-1.45 q < 0.19
SNRPN	small nuclear ribonucleoprotein polypeptide N	Ssc.26460.1.A1_at	1.22 q < 0.000294	1.24 q < 0.512	1.07 q < 0.92	1.28 q < 0.166	1.22 q < 1	1.3 q < 0.241
SNRPN	small nuclear ribonucleoprotein polypeptide N	Ssc.3875.1.S1_a_at	1.2 q < 0.000755	1.16 q < 0.897	1.04 q < 0.963	1.24 q < 0.233	1.23 q < 0.799	1.34 q < 0.0983
SNRPN	small nuclear ribonucleoprotein polypeptide N	Ssc.3875.2.A1_at	1.17 q < 0.00226	1.14 q < 0.968	-1.01 q < 0.99	1.3 q < 0.0815	1.18 q < 1	1.28 q < 0.197
FBXW11	F-box and WD repeat domain containing 11	Ssc.12819.1.A1_at	1.18 q < 0.000311	1.28 q < 0.134	1.23 q < 0.249	1.23 q < 0.164	1.13 q < 1	1.05 q < 1
INPP5F	inositol polyphosphate-5-phosphatase F	Ssc.17070.1.A1_s_at	-1.19 q < 0.000355	-1.18 q < 0.679	-1.14 q < 0.736	-1.21 q < 0.25	-1.21 q < 0.769	-1.2 q < 0.577
EAPP	E2F-associated phosphoprotein	Ssc.23226.1.S1_at	-1.26 q < 0.000356	-1.22 q < 0.796	-1.33 q < 0.257	-1.13 q < 0.647	-1.22 q < 1	-1.4 q < 0.13
DNAJA2	DnaJ (Hsp40) homolog, subfamily A, member 2	Ssc.1752.1.S1_at	1.28 q < 0.000419	1.44 q < 0.128	1.1 q < 0.913	1.31 q < 0.265	1.4 q < 0.361	1.17 q < 1
MYLE	Dexi homolog (mouse)	Ssc.28510.1.S1_at	-1.16 q < 0.000431	-1.17 q < 0.557	-1.26 q < 0.0711	-1.12 q < 0.474	-1.2 q < 0.522	-1.04 q < 1
RCN3	reticulocalbin 3, EF-hand calcium binding domain	Ssc.24393.1.S1_at	-1.36 q < 0.000433	-1.63 q < 0.104	-1.41 q < 0.384	-1.37 q < 0.323	-1.04 q < 1	-1.44 q < 0.428
ZSCAN29	zinc finger and SCAN domain containing 29	Ssc.5058.1.A1_at	1.2 q < 0.000433	1.16 q < 0.913	1.19 q < 0.56	1.34 q < 0.0568	1.17 q < 1	1.17 q < 0.937
CKAP4	cytoskeleton-associated protein 4	Ssc.2147.2.A1_at	-1.37 q < 0.000436	-1.62 q < 0.128	-1.52 q < 0.212	-1.11 q < 0.794	-1.34 q < 1	-1.34 q < 0.845
CKAP4	cytoskeleton-associated protein 4	Ssc.2147.1.A1_at	-1.24 q < 0.0108	-1.13 q < 1	-1.26 q < 0.635	-1.29 q < 0.382	-1.09 q < 1	-1.47 q < 0.202
HNRNPR	heterogeneous nuclear ribonucleoprotein R	Ssc.7683.1.A1_at	-1.18 q < 0.00045	-1.09 q < 1	-1.28 q < 0.11	-1.16 q < 0.409	-1.16 q < 1	-1.22 q < 0.422
DMRT1	doublesex and mab-3 related transcription factor 1	Ssc.4503.1.A1_at	-1.45 q < 0.000465	-1.42 q < 0.733	-1.52 q < 0.387	-1.56 q < 0.211	-1.36 q < 1	-1.42 q < 0.816
HSD17B10	hydroxysteroid (17-beta) dehydrogenase 10	Ssc.1301.1.S1_at	1.29 q < 0.000469	1.25 q < 0.795	1.15 q < 0.854	1.47 q < 0.0723	1.4 q < 0.449	1.19 q < 1
TMEM219	transmembrane protein 219	Ssc.11229.1.S1_at	-1.17 q < 0.000521	-1.08 q < 1	-1.3 q < 0.0682	-1.12 q < 0.528	-1.14 q < 1	-1.24 q < 0.279
TMEM59	transmembrane protein 59	Ssc.9592.1.A1_at	-1.31 q < 0.000527	-1.41 q < 0.315	-1.54 q < 0.0703	-1.03 q < 0.945	-1.34 q < 0.863	-1.26 q < 0.919
OPALIN	oligodendrocytic myelin paranodal and inner loop protein	Ssc.3497.1.A1_at	-1.16 q < 0.000535	-1.15 q < 0.732	-1.09 q < 0.856	-1.14 q < 0.436	-1.27 q < 0.163	-1.17 q < 0.651
CCDC144B	coiled-coil domain containing 144B	Ssc.1902.1.A1_at	-1.34 q < 0.000571	-1.48 q < 0.258	-1.56 q < 0.11	-1.48 q < 0.144	-1.07 q < 1	-1.18 q < 1
LRRC58	leucine rich repeat containing 58	Ssc.12280.1.A1_at	1.16 q < 0.0006	1.11 q < 1	1.16 q < 0.524	1.26 q < 0.079	1.17 q < 1	1.12 q < 1
MEST	mesoderm specific transcript homolog (mouse)	Ssc.6883.1.A1_at	-1.44 q < 0.000629	-1.42 q < 0.735	-1.44 q < 0.524	-1.57 q < 0.207	-1.21 q < 1	-1.62 q < 0.319
TRIM2	tripartite motif-containing 2	Ssc.7210.1.A1_at	1.2 q < 0.000634	1.24 q < 0.384	1.14 q < 0.765	1.18 q < 0.382	1.09 q < 1	1.34 q < 0.0828
TRIM2	tripartite motif-containing 2	Ssc.9717.1.A1_at	1.16 q < 0.00487	1.27 q < 0.263	1.16 q < 0.641	1.03 q < 0.921	1.08 q < 1	1.3 q < 0.158
GTF2A1	general transcription factor IIA, 1, 19/37kDa	Ssc.22615.1.A1_at	-1.21 q < 0.000638	-1.33 q < 0.149	-1.2 q < 0.555	-1.1 q < 0.681	-1.29 q < 0.438	-1.14 q < 1
RNF31	ring finger protein 31	Ssc.1625.1.S1_at	-1.18 q < 0.000638	-1.2 q < 0.53	-1.31 q < 0.0641	-1.22 q < 0.196	1.04 q < 1	-1.22 q < 0.402
PAQR3	progestin and adipoQ receptor family member III	Ssc.28652.1.A1_at	-1.36 q < 0.000665	-1.26 q < 0.972	-1.61 q < 0.106	-1.22 q < 0.584	-1.29 q < 1	-1.44 q < 0.456
TATDN1	TatD DNase domain containing 1	Ssc.26438.1.S1_at	-1.18 q < 0.000665	-1.2 q < 0.522	-1.23 q < 0.27	-1.1 q < 0.621	-1.22 q < 0.688	-1.14 q < 1
PEX19	peroxisomal biogenesis factor 19	Ssc.4292.1.S1_a_at	-1.22 q < 0.000667	-1.12 q < 1	-1.28 q < 0.298	-1.14 q < 0.579	-1.19 q < 1	-1.4 q < 0.0693
ATAD2B	ATPase family, AAA domain containing 2B	Ssc.30465.1.A1_at	-1.17 q < 0.000677	-1.22 q < 0.355	-1.17 q < 0.515	-1.07 q < 0.738	-1.25 q < 0.389	-1.16 q < 0.871
C9orf19	GLI pathogenesis-related 2	Ssc.1850.1.A1_at	-1.22 q < 0.000711	-1.36 q < 0.159	-1.13 q < 0.832	-1.25 q < 0.273	-1.05 q < 1	-1.35 q < 0.158
XPO1	exportin 1 (CRM1 homolog, yeast)	Ssc.22357.1.A1_at	1.24 q < 0.000729	1.25 q < 0.621	1.21 q < 0.641	1.35 q < 0.128	1.15 q < 1	1.24 q < 0.731
VMO1	vitelline membrane outer layer 1 homolog (chicken)	Ssc.19013.2.A1_at	1.23 q < 0.000737	1.26 q < 0.541	1.05 q < 0.957	1.34 q < 0.127	1.29 q < 0.699	1.25 q < 0.671
ADAM12	ADAM metallopeptidase domain 12	Ssc.1692.1.A1_at	-1.18 q < 0.000766	-1.11 q < 1	-1.3 q < 0.0783	-1.12 q < 0.548	-1.2 q < 0.83	-1.15 q < 0.976
BRP44L	brain protein 44-like	Ssc.8972.1.S1_at	1.33 q < 0.00077	1.48 q < 0.263	1.1 q < 0.933	1.21 q < 0.584	1.61 q < 0.133	1.33 q < 0.784
BTF3	basic transcription factor 3	Ssc.11125.1.S1_at	-1.33 q < 0.000781	-1.05 q < 1	-1.6 q < 0.074	-1.29 q < 0.414	-1.26 q < 1	-1.51 q < 0.202
BTF3	butyrophilin, subfamily 3, member A3	Ssc.11125.1.S1_at	-1.33 q < 0.000781	-1.05 q < 1	-1.6 q < 0.074	-1.29 q < 0.414	-1.26 q < 1	-1.51 q < 0.202
ATRX	alpha thalassemia/mental retardation syndrome X-linked	Ssc.24244.2.A1_at	-1.17 q < 0.000802	-1.12 q < 1	-1.22 q < 0.287	-1.14 q < 0.439	-1.26 q < 0.263	-1.1 q < 1
ATRX	alpha thalassemia/mental retardation syndrome X-linked	Ssc.23856.1.A1_at	-1.16 q < 0.00138	-1.12 q < 0.993	-1.16 q < 0.545	-1.19 q < 0.254	-1.16 q < 1	-1.16 q < 0.815
ATRX	alpha thalassemia/mental retardation syndrome X-linked	Ssc.30753.1.S1_at	-1.18 q < 0.00356	-1.14 q < 1	-1.27 q < 0.273	-1.14 q < 0.545	-1.19 q < 1	-1.17 q < 0.984
LCP2	lymphocyte cytosolic protein 2 (SH2 domain containing leukocyte protein of 76kDa)	Ssc.15565.1.S1_at	-1.24 q < 0.000814	-1.14 q < 1	-1.31 q < 0.282	-1.17 q < 0.53	-1.18 q < 1	-1.41 q < 0.111
RPS20	ribosomal protein S20	Ssc.20036.1.S1_at	-1.14 q < 0.000847	-1.1 q < 0.995	-1.21 q < 0.133	-1.09 q < 0.55	-1.07 q < 1	-1.21 q < 0.174
COX7C	cytochrome c oxidase subunit VIIc	Ssc.1461.1.S1_at	1.34 q < 0.000864	1.42 q < 0.441	1.1 q < 0.939	1.52 q < 0.123	1.4 q < 0.848	1.3 q < 0.923

Gene Name	Gene Description	Probe Set ID	Breed MS-WC	Day 25 MS-WC	Day 45 MS-WC	Day 65 MS-WC	Day 85 MS-WC	Day 105 MS-WC
SERPINE2	serpin peptidase inhibitor, clade E (nexin, plasminogen activator inhibitor type 1), member 2	Ssc.16342.1.A1_at	-1.51 q < 0.000888	-1.67 q < 0.399	-1.78 q < 0.193	-1.84 q < 0.106	-1.15 q < 1	-1.25 q < 1
SERPINE2	serpin peptidase inhibitor, clade E (nexin, plasminogen activator inhibitor type 1), member 2	Ssc.8736.1.A1_at	1.1 q < 0.23	-1 q < 1	1.07 q < 0.93	1.39 q < 0.0325	-1.02 q < 1	1.1 q < 1
MRPS2	mitochondrial ribosomal protein S2	Ssc.1787.1.A1_at	-1.17 q < 0.000917	-1.11 q < 1	-1.3 q < 0.0714	-1.1 q < 0.604	-1.24 q < 0.42	-1.1 q < 1
LOH12CR1	loss of heterozygosity, 12, chromosomal region 1	Ssc.21514.1.S1_at	-1.13 q < 0.000932	-1.07 q < 1	-1.16 q < 0.354	-1.11 q < 0.477	-1.15 q < 0.813	-1.16 q < 0.456
MMP2	matrix metallopeptidase 2 (gelatinase A, 72kDa gelatinase, 72kDa type IV collagenase)	Ssc.5713.1.S1_at	-1.42 q < 0.000944	-1.37 q < 0.829	-1.48 q < 0.43	-1.75 q < 0.0706	-1.13 q < 1	-1.43 q < 0.731
C13orf31	chromosome 13 open reading frame 31	Ssc.27190.1.S1_at	1.15 q < 0.000981	1.17 q < 0.567	1.15 q < 0.552	1.2 q < 0.179	1.2 q < 0.567	1.04 q < 1
ETF1	eukaryotic translation termination factor 1	Ssc.6041.2.A1_at	-1.26 q < 0.00102	-1.25 q < 0.783	-1.27 q < 0.513	-1.17 q < 0.565	-1.35 q < 0.559	-1.26 q < 0.785
C4orf30	DDB1 and CUL4 associated factor 16	Ssc.22469.1.A1_at	1.16 q < 0.00103	1.2 q < 0.441	1.14 q < 0.667	1.17 q < 0.332	1.11 q < 1	1.2 q < 0.493
RNF13	ring finger protein 13	Ssc.8275.1.A1_at	-1.32 q < 0.00105	-1.33 q < 0.671	-1.27 q < 0.65	-1.13 q < 0.736	-1.57 q < 0.173	-1.32 q < 0.779
GSN	gelsolin	Ssc.14246.1.S1_at	-1.37 q < 0.00106	-1.35 q < 0.785	-1.28 q < 0.737	-1.34 q < 0.403	-1.23 q < 1	-1.67 q < 0.105
CYP2U1	cytochrome P450, family 2, subfamily U, polypeptide 1	Ssc.28083.1.A1_at	-1.17 q < 0.00108	-1.15 q < 0.89	-1.15 q < 0.673	-1.18 q < 0.35	-1.25 q < 0.42	-1.14 q < 1
SLC4A5	solute carrier family 4, sodium bicarbonate cotransporter, member 5	Ssc.14147.1.A1_at	-1.28 q < 0.00113	-1.57 q < 0.0574	-1.44 q < 0.176	-1.14 q < 0.669	-1.17 q < 1	-1.15 q < 1
SLC4A5	solute carrier family 4, sodium bicarbonate cotransporter, member 4	Ssc.14147.1.A1_at	-1.28 q < 0.00113	-1.57 q < 0.0574	-1.44 q < 0.176	-1.14 q < 0.669	-1.17 q < 1	-1.15 q < 1
OGFOD1	2-oxoglutarate and iron-dependent oxygenase domain containing 1	Ssc.3417.1.A1_at	-1.14 q < 0.00117	-1.14 q < 0.671	-1.14 q < 0.514	-1.1 q < 0.522	-1.2 q < 0.415	-1.1 q < 1
TUFM	Tu translation elongation factor, mitochondrial	Ssc.5285.1.S1_at	-1.21 q < 0.00117	-1.07 q < 1	-1.37 q < 0.0874	-1.15 q < 0.549	-1.19 q < 1	-1.3 q < 0.314
CXCL16	chemokine (C-X-C motif) ligand 16	Ssc.6583.1.S1_at	-1.43 q < 0.00119	-1.51 q < 0.526	-1.81 q < 0.0899	-1.3 q < 0.549	-1.29 q < 1	-1.31 q < 1
SERPINF1	serpin peptidase inhibitor, clade F (alpha-2 antiplasmin, pigment epithelium derived factor), member 1	Ssc.10451.1.S1_at	-1.5 q < 0.00123	-1.44 q < 0.851	-1.93 q < 0.105	-1.35 q < 0.546	-1.07 q < 1	-1.9 q < 0.139
POLR2I	polymerase (RNA) II (DNA directed) polypeptide I, 14.5kDa	Ssc.18382.1.S1_at	-1.18 q < 0.00125	-1.02 q < 1	-1.31 q < 0.1	-1.23 q < 0.226	-1.23 q < 0.758	-1.15 q < 1
C2orf79	chromosome 2 open reading frame 79	Ssc.6448.1.S1_at	1.2 q < 0.00126	1.16 q < 0.951	1.19 q < 0.611	1.28 q < 0.177	1.32 q < 0.302	1.07 q < 1
EXT1	exostosin 1	Ssc.3325.2.S1_a_at	-1.25 q < 0.00129	-1.35 q < 0.355	-1.36 q < 0.256	-1.21 q < 0.478	-1.16 q < 1	-1.2 q < 1
EXT1	exostosin 1	Ssc.3325.3.S1_at	-1.17 q < 0.0212	-1.32 q < 0.315	-1.29 q < 0.361	-1.03 q < 0.932	-1.1 q < 1	-1.17 q < 1
EXT1	exostosin 1	Ssc.7562.1.A1_at	-1.21 q < 0.0277	-1.44 q < 0.266	-1.24 q < 0.688	-1.31 q < 0.344	-1.11 q < 1	-1.02 q < 1
CANX	calnexin	Ssc.21107.1.S1_at	1.13 q < 0.0013	1.09 q < 1	1.15 q < 0.466	1.03 q < 0.865	1.16 q < 0.813	1.25 q < 0.0571
P4HA2	prolyl 4-hydroxylase, alpha polypeptide II	Ssc.16984.1.S1_at	-1.27 q < 0.00131	-1.39 q < 0.281	-1.24 q < 0.654	-1.15 q < 0.638	-1.23 q < 1	-1.35 q < 0.456
APOF	apolipoprotein F	Ssc.23221.1.S1_at	1.24 q < 0.00136	1.22 q < 0.838	1.15 q < 0.831	1.3 q < 0.248	1.4 q < 0.293	1.16 q < 1
C14orf100	JNK1/MAPK8-associated membrane protein	Ssc.5740.1.S1_at	1.2 q < 0.00136	1.25 q < 0.45	1.03 q < 0.976	1.28 q < 0.18	1.24 q < 0.844	1.22 q < 0.687
CYP2D6	cytochrome P450, family 2, subfamily D, polypeptide 6	Ssc.4617.1.S1_at	-1.2 q < 0.00138	-1.11 q < 1	-1.09 q < 0.902	-1.29 q < 0.159	-1.14 q < 1	-1.39 q < 0.0504
YKT6	YKT6 v-SNARE homolog (S. cerevisiae)	Ssc.1537.1.S1_at	-1.21 q < 0.00145	-1.16 q < 0.996	-1.35 q < 0.138	-1.16 q < 0.53	-1.11 q < 1	-1.3 q < 0.335
C16orf45	chromosome 16 open reading frame 45	Ssc.26539.1.S1_at	-1.44 q < 0.00145	-1.66 q < 0.304	-1.46 q < 0.526	-1.1 q < 0.847	-1.22 q < 1	-1.9 q < 0.0727
NUDT15	nudix (nucleoside diphosphate linked moiety X)-type motif 15	Ssc.13453.2.S1_at	-1.19 q < 0.00146	-1.08 q < 1	-1.35 q < 0.0752	-1.07 q < 0.782	-1.32 q < 0.227	-1.16 q < 1
NUDT15	nudix (nucleoside diphosphate linked moiety X)-type motif 15	Ssc.13453.3.A1_at	-1.13 q < 0.0323	1.01 q < 1	-1.28 q < 0.153	1.04 q < 0.874	-1.33 q < 0.0993	-1.13 q < 1
NSF	N-ethylmaleimide-sensitive factor	Ssc.9739.1.A1_at	-1.19 q < 0.00151	-1.14 q < 1	-1.03 q < 0.97	-1.26 q < 0.188	-1.34 q < 0.141	-1.19 q < 0.844
KATNAL2	katanin p60 subunit A-like 2	Ssc.29574.1.A1_at	1.22 q < 0.00152	-1.06 q < 1	1.14 q < 0.841	1.38 q < 0.0745	1.32 q < 0.524	1.38 q < 0.133
VEGFB	vascular endothelial growth factor B	Ssc.2095.1.S1_at	-1.19 q < 0.00153	-1.18 q < 0.8	-1.33 q < 0.114	-1.1 q < 0.689	-1.21 q < 1	-1.16 q < 1
HLA-DRB4	major histocompatibility complex, class II, DR beta 4	Ssc.210.7.S1_x_at	1.34 q < 0.00154	1.1 q < 1	1.49 q < 0.26	1.27 q < 0.495	1.75 q < 0.0587	1.18 q < 1
HLA-DRB4	major histocompatibility complex, class II, DR beta 4	Ssc.16169.1.S1_x_at	-1.22 q < 0.216	-1.07 q < 1	1.04 q < 0.987	1.01 q < 0.993	-2.33 q < 0.00753	-1.13 q < 1
ZMIZ1	zinc finger, MIZ-type containing 1	Ssc.2259.1.A1_at	-1.26 q < 0.00159	-1.3 q < 0.576	-1.33 q < 0.364	-1.29 q < 0.317	-1.25 q < 1	-1.14 q < 1
TRPS1	trichorhinophalangeal syndrome I	Ssc.30243.1.A1_at	-1.15 q < 0.00161	-1.17 q < 0.577	-1.09 q < 0.842	-1.19 q < 0.232	-1.12 q < 1	-1.18 q < 0.585
C7orf24	gamma-glutamylcyclotransferase	Ssc.19205.1.A1_at	-1.45 q < 0.00163	-1.02 q < 1	-1.67 q < 0.249	-1.23 q < 0.663	-1.74 q < 0.37	-1.76 q < 0.201
C7orf24	gamma-glutamylcyclotransferase	Ssc.19205.2.S1_at	-1.3 q < 0.0292	1.08 q < 1	-1.51 q < 0.393	-1.27 q < 0.579	-1.44 q < 1	-1.45 q < 0.698
ENTPD4	ectonucleoside triphosphate diphosphohydrolase 4	Ssc.8305.1.A1_at	-1.28 q < 0.00163	-1.34 q < 0.527	-1.35 q < 0.4	1.01 q < 0.984	-1.44 q < 0.397	-1.34 q < 0.567
RBM18	RNA binding motif protein 18	Ssc.9676.1.S1_at	1.18 q < 0.00166	1.1 q < 1	1.3 q < 0.147	1.22 q < 0.288	1.14 q < 1	1.18 q < 0.893

Gene Name	Gene Description	Probe Set ID	Breed MS-WC	Day 25 MS-WC	Day 45 MS-WC	Day 65 MS-WC	Day 85 MS-WC	Day 105 MS-WC
NCOA5	nuclear receptor coactivator 5	Ssc.12924.1.A1_at	1.18 q < 0.00166	1.1 q < 1	1.15 q < 0.738	1.28 q < 0.131	1.16 q < 1	1.24 q < 0.429
ICK	intestinal cell (MAK-like) kinase	Ssc.29658.1.A1_at	-1.18 q < 0.00167	-1.24 q < 0.408	-1.16 q < 0.671	-1.13 q < 0.558	-1.27 q < 0.495	-1.13 q < 1
CYB5D2	cytochrome b5 domain containing 2	Ssc.2472.1.S1_at	-1.2 q < 0.00169	-1.19 q < 0.819	-1.29 q < 0.232	-1.17 q < 0.487	-1.03 q < 1	-1.35 q < 0.128
HARSL	histidyl-tRNA synthetase 2, mitochondrial (putative)	Ssc.12954.1.S1_at	-1.21 q < 0.00174	-1.12 q < 1	-1.36 q < 0.125	-1.09 q < 0.747	-1.22 q < 1	-1.28 q < 0.416
DMPK	dystrophia myotonica-protein kinase	Ssc.28696.1.S1_at	-1.22 q < 0.00177	-1.38 q < 0.152	-1.39 q < 0.0992	-1.04 q < 0.883	-1.1 q < 1	-1.22 q < 0.855
DSE	dermatan sulfate epimerase	Ssc.29146.1.A1_at	-1.3 q < 0.00177	-1.06 q < 1	-1.52 q < 0.128	-1.48 q < 0.128	-1.2 q < 1	-1.3 q < 0.852
IFT88	intraflagellar transport 88 homolog (Chlamydomonas)	Ssc.25960.1.S1_at	-1.16 q < 0.00178	-1.01 q < 1	-1.28 q < 0.0933	-1.01 q < 0.968	-1.22 q < 0.602	-1.31 q < 0.053
C21orf100	non-protein coding RNA 161	Ssc.15439.1.A1_at	-1.27 q < 0.00187	-1.32 q < 0.557	-1.12 q < 0.902	-1.5 q < 0.0643	-1.16 q < 1	-1.29 q < 0.774
SLC2A8	solute carrier family 2 (facilitated glucose transporter), member 8	Ssc.12400.1.S1_at	1.23 q < 0.00188	1.12 q < 1	1.32 q < 0.271	1.35 q < 0.142	1.17 q < 1	1.18 q < 1
MTERFD1	MTERF domain containing 1	Ssc.27515.1.S1_at	1.19 q < 0.00189	1.18 q < 0.867	1.14 q < 0.799	1.26 q < 0.212	1.19 q < 1	1.21 q < 0.761
KIAA1598	KIAA1598	Ssc.8371.1.A1_at	1.15 q < 0.00192	1.29 q < 0.0541	1.04 q < 0.942	1.15 q < 0.362	1.21 q < 0.558	1.05 q < 1
TMED5	transmembrane emp24 protein transport domain containing 5	Ssc.9167.1.A1_at	1.17 q < 0.00192	1.16 q < 0.864	1.12 q < 0.821	1.17 q < 0.396	1.22 q < 0.804	1.21 q < 0.623
CCDC24	coiled-coil domain containing 24	Ssc.1926.1.A1_at	-1.15 q < 0.00195	-1.15 q < 0.777	-1.25 q < 0.142	1.03 q < 0.873	-1.24 q < 0.4	-1.18 q < 0.623
GPR98	G protein-coupled receptor 98	Ssc.30004.1.A1_at	-1.17 q < 0.00209	-1.01 q < 1	-1.23 q < 0.289	-1.16 q < 0.436	-1.34 q < 0.081	-1.13 q < 1
AP1S2	adaptor-related protein complex 1, sigma 2 subunit	Ssc.4135.2.A1_at	1.44 q < 0.0021	1.25 q < 1	1.61 q < 0.315	1.85 q < 0.0683	1.23 q < 1	1.33 q < 1
AP1S2	adaptor-related protein complex 1, sigma 2 subunit	Ssc.4135.1.S1_at	1.12 q < 0.00573	-1.01 q < 1	1.22 q < 0.124	1.22 q < 0.0831	1.04 q < 1	1.14 q < 0.723
HSPA12A	heat shock 70kDa protein 12A	Ssc.14293.1.A1_at	1.15 q < 0.00213	1.24 q < 0.199	1.23 q < 0.168	1.24 q < 0.105	-1.03 q < 1	1.08 q < 1
PRKX	protein kinase, X-linked	Ssc.2560.1.S1_at	1.26 q < 0.00215	1.33 q < 0.485	1.28 q < 0.508	1.28 q < 0.354	1.24 q < 1	1.16 q < 1
KCNMA1	potassium large conductance calcium-activated channel, subfamily M, alpha member 1	Ssc.8148.2.S1_at	-1.29 q < 0.00222	-1.4 q < 0.432	-1.58 q < 0.0762	-1.22 q < 0.547	-1.12 q < 1	-1.21 q < 1
ISLR	immunoglobulin superfamily containing leucine-rich repeat	Ssc.1064.1.A1_at	-1.24 q < 0.00227	-1.28 q < 0.613	-1.38 q < 0.192	-1.27 q < 0.332	-1.13 q < 1	-1.16 q < 1
GAS1	growth arrest-specific 1	Ssc.21626.1.A1_at	-1.38 q < 0.00229	-1.35 q < 0.85	-1.76 q < 0.0838	-1.62 q < 0.14	-1.16 q < 1	-1.12 q < 1
GAS1	growth arrest-specific 1	Ssc.21626.2.S1_at	-1.26 q < 0.0333	-1.25 q < 1	-1.51 q < 0.279	-1.41 q < 0.308	-1.06 q < 1	-1.12 q < 1
COL18A1	collagen, type XVIII, alpha 1	Ssc.4892.1.S1_at	-1.28 q < 0.00234	-1.5 q < 0.153	-1.56 q < 0.0711	-1.16 q < 0.645	-1.28 q < 1	1.01 q < 1
C1orf59	chromosome 1 open reading frame 59	Ssc.14081.1.A1_at	-1.38 q < 0.00234	-1.13 q < 1	-1.63 q < 0.182	-1.52 q < 0.223	-1.21 q < 1	-1.48 q < 0.58
PRKG1	protein kinase, cGMP-dependent, type I	Ssc.26918.1.A1_at	1.14 q < 0.00236	1.07 q < 1	1.11 q < 0.766	1.17 q < 0.276	1.09 q < 1	1.28 q < 0.0605
ZMYND8	zinc finger, MYND-type containing 8	Ssc.5447.1.A1_at	-1.16 q < 0.00245	-1.01 q < 1	-1.3 q < 0.0621	-1.1 q < 0.626	-1.19 q < 0.907	-1.2 q < 0.536
PDZK1IP1	PDZK1 interacting protein 1	Ssc.1743.1.S1_at	1.2 q < 0.00246	1.26 q < 0.484	1.02 q < 0.984	1.11 q < 0.67	1.24 q < 1	1.42 q < 0.0549
LTV1	LTV1 homolog (S. cerevisiae)	Ssc.24930.1.S1_at	-1.21 q < 0.00249	-1.18 q < 0.919	-1.24 q < 0.481	-1.17 q < 0.5	-1.17 q < 1	-1.28 q < 0.473
RPS8	ribosomal protein S8	Ssc.12202.1.A1_at	-1.09 q < 0.00255	-1.01 q < 1	-1.1 q < 0.494	-1.11 q < 0.223	-1.06 q < 1	-1.15 q < 0.0951
TOB2	transducer of ERBB2, 2	Ssc.2933.1.S1_at	-1.24 q < 0.00256	-1.36 q < 0.32	-1.5 q < 0.0521	-1.12 q < 0.696	-1.16 q < 1	-1.11 q < 1
FYN	FYN oncogene related to SRC, FGR, YES	Ssc.2714.1.S1_a_at	-1.23 q < 0.00272	-1.05 q < 1	-1.33 q < 0.297	-1.37 q < 0.131	-1.15 q < 1	-1.28 q < 0.643
F3	coagulation factor III (thromboplastin, tissue factor)	Ssc.19907.1.S1_at	-1.43 q < 0.00277	-1.58 q < 0.454	-1.73 q < 0.186	-1.11 q < 0.841	-1.55 q < 0.868	-1.25 q < 1
F3	contactin 1	Ssc.19907.1.S1_at	-1.43 q < 0.00277	-1.58 q < 0.454	-1.73 q < 0.186	-1.11 q < 0.841	-1.55 q < 0.868	-1.25 q < 1
KCMF1	potassium channel modulatory factor 1	Ssc.25963.1.A1_at	1.19 q < 0.00286	1.22 q < 0.66	1.14 q < 0.789	1.25 q < 0.267	1.11 q < 1	1.25 q < 0.544
SLC25A5	solute carrier family 25 (mitochondrial carrier; adenine nucleotide translocator), member 5	Ssc.5999.1.A1_at	1.32 q < 0.00287	1.48 q < 0.351	1.02 q < 0.99	1.62 q < 0.0716	1.5 q < 0.495	1.1 q < 1
RABEPK	Rab9 effector protein with kelch motifs	Ssc.30706.1.A1_at	-1.21 q < 0.00295	-1.08 q < 1	-1.41 q < 0.0716	-1.14 q < 0.593	-1.23 q < 1	-1.21 q < 0.912
SLC25A23	solute carrier family 25 (mitochondrial carrier; phosphate carrier), member 23	Ssc.17440.1.S1_at	-1.19 q < 0.00301	-1.29 q < 0.351	-1.18 q < 0.677	-1.13 q < 0.59	-1.29 q < 0.524	-1.1 q < 1
FBLN1	fibulin 1	Ssc.20870.1.S1_at	-1.39 q < 0.00312	-1.79 q < 0.107	-1.35 q < 0.677	-1.67 q < 0.123	-1.08 q < 1	-1.18 q < 1
LY96	lymphocyte antigen 96	Ssc.25550.1.S1_at	-1.23 q < 0.00312	-1.3 q < 0.487	-1.07 q < 0.943	-1.2 q < 0.474	-1.13 q < 1	-1.48 q < 0.0597
KIAA1949	KIAA1949	Ssc.29821.1.A1_at	-1.15 q < 0.00315	-1.17 q < 0.68	-1.2 q < 0.409	-1.03 q < 0.912	-1.13 q < 1	-1.26 q < 0.205
TMEM106A	transmembrane protein 106A	Ssc.9906.2.A1_at	-1.16 q < 0.00316	-1.12 q < 1	-1.16 q < 0.641	-1.1 q < 0.626	-1.26 q < 0.423	-1.18 q < 0.747
DNAH11	dynein, axonemal, heavy chain 11	Ssc.29936.1.A1_at	-1.19 q < 0.00325	-1.17 q < 0.899	-1.22 q < 0.507	-1.24 q < 0.279	-1.16 q < 1	-1.16 q < 1
LYPD6	LY6/PLAUR domain containing 6	Ssc.24669.1.A1_at	1.29 q < 0.00329	1.4 q < 0.441	1.32 q < 0.529	1.39 q < 0.242	1.21 q < 1	1.13 q < 1
C6orf162	chromosome 6 open reading frame 162	Ssc.17900.1.S1_at	-1.17 q < 0.0034	-1.06 q < 1	-1.21 q < 0.445	-1.24 q < 0.215	-1.11 q < 1	-1.25 q < 0.404
SBF2	SET binding factor 2	Ssc.16986.1.S1_at	-1.36 q < 0.00346	-1.07 q < 1	-1.72 q < 0.0932	-1.1 q < 0.842	-1.36 q < 1	-1.68 q < 0.144

Gene Name	Gene Description	Probe Set ID	Breed MS-WC	Day 25 MS-WC	Day 45 MS-WC	Day 65 MS-WC	Day 85 MS-WC	Day 105 MS-WC
FRMD6	FERM domain containing 6	Ssc.11215.1.S1_at	-1.29 q < 0.00347	-1.37 q < 0.527	-1.35 q < 0.45	-1.21 q < 0.553	-1.09 q < 1	-1.43 q < 0.359
SEMA3E	sema domain, immunoglobulin domain (Ig), short basic domain, secreted, (semaphorin) 3E	Ssc.17824.1.A1_at	-1.22 q < 0.00347	-1.41 q < 0.146	-1.26 q < 0.498	-1.35 q < 0.149	-1.28 q < 0.899	1.13 q < 1
SEMA3E	sema domain, immunoglobulin domain (Ig), short basic domain, secreted, (semaphorin) 3E	Ssc.17824.2.A1_at	-1.19 q < 0.00832	-1.21 q < 0.809	-1.4 q < 0.0854	-1.3 q < 0.198	-1.15 q < 1	1.06 q < 1
MAN2A2	mannosidase, alpha, class 2A, member 2	Ssc.1529.1.A1_at	-1.33 q < 0.0035	-1.42 q < 0.557	-1.49 q < 0.31	-1.27 q < 0.528	-1.38 q < 1	-1.14 q < 1
CNNM2	cyclin M2	Ssc.1808.1.S1_at	-1.15 q < 0.0035	-1.12 q < 1	-1.28 q < 0.111	1 q < 0.991	-1.12 q < 1	-1.28 q < 0.128
TNXA	tenascin XA pseudogene	Ssc.19638.1.S1_at	-1.54 q < 0.00363	-1.33 q < 1	-1.65 q < 0.494	-1.42 q < 0.537	-1.22 q < 1	-2.27 q < 0.0651
TNXA	tenascin XA pseudogene	Ssc.27043.1.A1_at	1.19 q < 0.0193	1.01 q < 1	1.12 q < 0.885	1.32 q < 0.212	1.24 q < 1	1.29 q < 0.596
ABO	ABO blood group (transferase A, alpha 1-3-N-acetylgalactosaminyltransferase; transferase B, alpha 1-3-galactosyltransferase)	Ssc.17029.1.A1_at	-1.19 q < 0.00368	-1.03 q < 1	-1.38 q < 0.0758	-1.19 q < 0.43	-1.12 q < 1	-1.28 q < 0.394
RNF38	ring finger protein 38	Ssc.30608.1.A1_at	-1.2 q < 0.00369	-1.24 q < 0.667	-1.42 q < 0.0676	-1.09 q < 0.755	-1.19 q < 1	-1.12 q < 1
NFKBIA	nuclear factor of kappa light polypeptide gene enhancer in B-cells inhibitor, alpha	Ssc.4759.1.S1_at	-1.16 q < 0.00381	-1.09 q < 1	-1.24 q < 0.274	-1.05 q < 0.827	-1.21 q < 0.884	-1.24 q < 0.42
CDH2	cadherin 2, type 1, N-cadherin (neuronal)	Ssc.31099.1.A1_at	1.14 q < 0.00398	1.11 q < 1	1.09 q < 0.861	1.16 q < 0.35	1.09 q < 1	1.27 q < 0.113
LPHN2	latrophilin 2	Ssc.15595.1.S1_at	1.14 q < 0.00406	1.19 q < 0.432	1.06 q < 0.913	1.17 q < 0.322	1.17 q < 1	1.11 q < 1
NKTR	natural killer-tumor recognition sequence	Ssc.30611.1.A1_at	1.17 q < 0.00406	1.14 q < 1	1.07 q < 0.913	1.28 q < 0.124	1.14 q < 1	1.21 q < 0.608
C2orf34	chromosome 2 open reading frame 34	Ssc.13318.1.A1_at	1.16 q < 0.00407	1.15 q < 0.849	1.09 q < 0.878	1.18 q < 0.345	1.11 q < 1	1.25 q < 0.257
IL3RA	interleukin 3 receptor, alpha (low affinity)	Ssc.29769.1.A1_at	1.19 q < 0.0041	1.16 q < 1	1.07 q < 0.936	1.17 q < 0.503	1.22 q < 1	1.38 q < 0.103
PLD3	phospholipase D family, member 3	Ssc.2571.1.S1_at	-1.3 q < 0.00417	-1.44 q < 0.382	-1.49 q < 0.216	-1.13 q < 0.739	-1.27 q < 1	-1.18 q < 1
SUSD1	sushi domain containing 1	Ssc.24293.1.A1_a_at	-1.16 q < 0.00417	-1.18 q < 0.679	-1.12 q < 0.783	-1.15 q < 0.469	-1.18 q < 1	-1.16 q < 0.909
MRPL44	mitochondrial ribosomal protein L44	Ssc.11581.1.S1_at	-1.21 q < 0.00422	-1.2 q < 0.897	-1.31 q < 0.31	-1.01 q < 0.987	-1.24 q < 1	-1.34 q < 0.296
INTS3	integrator complex subunit 3	Ssc.2238.1.S1_at	-1.18 q < 0.00427	-1.17 q < 0.91	-1.32 q < 0.145	-1.05 q < 0.845	-1.28 q < 0.542	-1.11 q < 1
INTS3	integrator complex subunit 3	Ssc.13701.1.S1_at	-1.13 q < 0.0325	-1.09 q < 1	-1.28 q < 0.142	-1.06 q < 0.805	-1.16 q < 1	-1.06 q < 1
PACSIN2	protein kinase C and casein kinase substrate in neurons 2	Ssc.16537.1.S1_at	1.21 q < 0.00433	1.1 q < 1	1.04 q < 0.968	1.41 q < 0.0658	1.12 q < 1	1.43 q < 0.0741
CUTA	cutA divalent cation tolerance homolog (E. coli)	Ssc.3128.2.S1_at	-1.27 q < 0.00455	-1.22 q < 1	-1.5 q < 0.147	-1.29 q < 0.404	-1.11 q < 1	-1.28 q < 0.921
CCNA1	cyclin A1	Ssc.24262.1.A1_at	-1.35 q < 0.00461	-1.04 q < 1	-1.58 q < 0.227	-1.23 q < 0.612	-1.51 q < 0.743	-1.47 q < 0.58
SLC2A5	solute carrier family 2 (facilitated glucose/fructose transporter), member 5	Ssc.16926.1.S1_at	1.67 q < 0.00464	1.2 q < 1	1.96 q < 0.4	2.36 q < 0.109	1.46 q < 1	1.63 q < 1
DONSON	downstream neighbor of SON	Ssc.26428.1.S1_at	1.18 q < 0.00468	1.23 q < 0.607	1.11 q < 0.872	1.27 q < 0.208	1.29 q < 0.495	1.04 q < 1
RNF7	ring finger protein 7	Ssc.18436.3.A1_a_at	-1.22 q < 0.0047	-1.33 q < 0.36	-1.48 q < 0.0574	-1.18 q < 0.524	-1.07 q < 1	-1.09 q < 1
CXCL14	chemokine (C-X-C motif) ligand 14	Ssc.20578.1.S1_at	-1.39 q < 0.00471	1.06 q < 1	-1.63 q < 0.251	1.06 q < 0.919	-1.88 q < 0.117	-1.88 q < 0.0714
CXCL14	chemokine (C-X-C motif) ligand 14	Ssc.4984.1.S1_at	-1.46 q < 0.0278	1.3 q < 1	-2.04 q < 0.196	1.15 q < 0.842	-2.31 q < 0.156	-2.1 q < 0.206
C6orf145	chromosome 6 open reading frame 145	Ssc.15664.1.S1_at	-1.27 q < 0.00473	-1.32 q < 0.652	-1.36 q < 0.406	-1 q < 0.998	-1.31 q < 1	-1.39 q < 0.44
HERC1	hect (homologous to the E6-AP (UBE3A) carboxyl terminus) domain and RCC1 (CHC1)-like domain (RLD) 1	Ssc.6629.1.S1_at	-1.16 q < 0.00478	-1.01 q < 1	-1.29 q < 0.129	-1.14 q < 0.512	-1.26 q < 0.411	-1.12 q < 1
Q96EJ7	-	Ssc.15958.1.S1_at	1.23 q < 0.00478	1.17 q < 1	1.23 q < 0.658	1.42 q < 0.102	1.24 q < 1	1.13 q < 1
TUSC3	tumor suppressor candidate 3	Ssc.6817.1.A1_a_at	1.4 q < 0.00478	1.33 q < 1	1.27 q < 0.842	1.72 q < 0.131	1.16 q < 1	1.6 q < 0.433
APOBEC4	apolipoprotein B mRNA editing enzyme, catalytic polypeptide-like 4 (putative)	Ssc.4154.1.A1_at	-1.15 q < 0.00486	-1.23 q < 0.36	-1.19 q < 0.459	-1.06 q < 0.761	-1.13 q < 1	-1.15 q < 0.936
CHCHD8	coiled-coil-helix-coiled-coil-helix domain containing 8	Ssc.12501.1.S1_at	1.15 q < 0.00486	1.18 q < 0.628	1.06 q < 0.918	1.19 q < 0.304	1.18 q < 1	1.14 q < 1
ACBD6	acyl-CoA binding domain containing 6	Ssc.12913.1.A1_at	-1.14 q < 0.00487	-1.14 q < 0.851	-1.23 q < 0.216	-1.19 q < 0.251	-1.14 q < 1	-1.01 q < 1
EGLN1	egl nine homolog 1 (C. elegans)	Ssc.5854.1.S1_at	1.2 q < 0.00487	1.14 q < 1	1.06 q < 0.943	1.3 q < 0.197	1.18 q < 1	1.32 q < 0.283
CD44	CD44 molecule (Indian blood group)	Ssc.15556.1.S1_at	1.43 q < 0.00494	1.91 q < 0.118	1.14 q < 0.936	1.76 q < 0.138	1.25 q < 1	1.24 q < 1
RHOBTB1	Rho-related BTB domain containing 1	Ssc.18959.1.A1_at	1.16 q < 0.00497	1.11 q < 1	1.02 q < 0.981	1.31 q < 0.0594	1.04 q < 1	1.33 q < 0.0572
ABCB7	ATP-binding cassette, sub-family B (MDR/TAP), member 7	Ssc.21338.1.S1_at	-1.18 q < 0.00501	-1.03 q < 1	-1.32 q < 0.163	-1 q < 0.992	-1.42 q < 0.0642	-1.2 q < 0.864
COPA	coatomer protein complex, subunit alpha	Ssc.13207.1.A1_at	-1.21 q < 0.00515	-1.23 q < 0.773	-1.36 q < 0.195	-1.11 q < 0.693	-1.09 q < 1	-1.28 q < 0.59
PPP1R3E	protein phosphatase 1, regulatory (inhibitor) subunit 3E	Ssc.6745.2.A1_a_at	-1.13 q < 0.00518	-1.01 q < 1	-1.16 q < 0.493	-1.11 q < 0.521	-1.26 q < 0.142	-1.13 q < 0.984
Q8NHG7	-	Ssc.29609.2.A1_at	1.21 q < 0.00518	1.03 q < 1	1.31 q < 0.32	1.43 q < 0.0571	1.12 q < 1	1.2 q < 0.997

Gene Name	Gene Description	Probe Set ID	Breed MS-WC	Day 25 MS-WC	Day 45 MS-WC	Day 65 MS-WC	Day 85 MS-WC	Day 105 MS-WC
PARC	cullin 9	Ssc.19484.1.A1_at	-1.11 q < 0.00521	-1.12 q < 0.81	-1.05 q < 0.923	-1.13 q < 0.379	-1.2 q < 0.318	-1.08 q < 1
PARC	chemokine (C-C motif) ligand 18 (pulmonary and activation-regulated)	Ssc.19484.1.A1_at	-1.11 q < 0.00521	-1.12 q < 0.81	-1.05 q < 0.923	-1.13 q < 0.379	-1.2 q < 0.318	-1.08 q < 1
C4orf27	chromosome 4 open reading frame 27	Ssc.1956.1.S1_a_at	-1.16 q < 0.00524	1.04 q < 1	-1.28 q < 0.181	-1.2 q < 0.325	-1.17 q < 1	-1.24 q < 0.42
TAGLN2	transgelin 2	Ssc.17300.1.S1_at	-1.48 q < 0.00528	-1.25 q < 1	-1.62 q < 0.47	-1.47 q < 0.457	-1.21 q < 1	-1.98 q < 0.15
ATP6V1D	ATPase, H+ transporting, lysosomal 34kDa, V1 subunit D	Ssc.14416.1.A1_at	-1.31 q < 0.00532	-1.34 q < 0.787	-1.36 q < 0.532	-1.31 q < 0.444	-1.12 q < 1	-1.45 q < 0.498
FAM114A2	family with sequence similarity 114, member A2	Ssc.30685.1.A1_at	1.22 q < 0.00539	1.02 q < 1	1.26 q < 0.539	1.33 q < 0.223	1.27 q < 1	1.28 q < 0.681
SERF1B	small EDRK-rich factor 1B (centromeric)	Ssc.25510.1.S1_at	-1.16 q < 0.00545	-1.13 q < 1	-1.24 q < 0.282	-1.04 q < 0.86	-1.25 q < 0.469	-1.13 q < 1
PAIP2	poly(A) binding protein interacting protein 2	Ssc.10336.1.S1_at	-1.18 q < 0.00547	-1.08 q < 1	-1.37 q < 0.0625	-1.13 q < 0.593	-1.18 q < 1	-1.14 q < 1
CCDC115	coiled-coil domain containing 115	Ssc.4903.1.A1_at	1.28 q < 0.00548	1.2 q < 1	1.29 q < 0.626	1.37 q < 0.281	1.4 q < 0.758	1.15 q < 1
RSRC2	arginine/serine-rich coiled-coil 2	Ssc.19083.2.A1_at	1.15 q < 0.0055	1.04 q < 1	1.16 q < 0.629	1.17 q < 0.376	1.21 q < 0.823	1.19 q < 0.66
MRPL22	mitochondrial ribosomal protein L22	Ssc.2756.1.A1_at	-1.22 q < 0.00558	-1.18 q < 1	-1.29 q < 0.412	-1.24 q < 0.393	-1.16 q < 1	-1.21 q < 0.99
FAM111B	family with sequence similarity 111, member B	Ssc.24695.1.A1_at	-1.14 q < 0.0056	-1.15 q < 0.741	-1.11 q < 0.803	-1.11 q < 0.533	-1.09 q < 1	-1.22 q < 0.277
HNRNPC	heterogeneous nuclear ribonucleoprotein C (C1/C2)	Ssc.24754.1.A1_at	1.18 q < 0.00563	1.31 q < 0.259	1.16 q < 0.738	1.23 q < 0.327	1.11 q < 1	1.12 q < 1
COL27A1	collagen, type XXVII, alpha 1	Ssc.3861.1.S1_at	-1.14 q < 0.00568	-1.03 q < 1	-1.2 q < 0.337	-1.15 q < 0.395	-1.18 q < 1	-1.14 q < 0.929
COPZ2	coatomer protein complex, subunit zeta 2	Ssc.4221.1.S1_at	-1.27 q < 0.00571	-1.17 q < 1	-1.5 q < 0.136	-1.11 q < 0.758	-1.22 q < 1	-1.36 q < 0.592
GMPR2	guanosine monophosphate reductase 2	Ssc.11000.1.S1_at	-1.23 q < 0.00571	-1.1 q < 1	-1.45 q < 0.109	-1.15 q < 0.639	-1.24 q < 1	-1.24 q < 0.912
ANXA11	annexin A11	Ssc.2918.1.S1_at	-1.21 q < 0.00572	-1.17 q < 1	-1.3 q < 0.384	-1.19 q < 0.515	-1.19 q < 1	-1.22 q < 0.921
GPC6	glypican 6	Ssc.2834.1.A1_at	-1.19 q < 0.00574	-1.36 q < 0.136	-1.27 q < 0.342	-1.22 q < 0.344	-1.06 q < 1	-1.05 q < 1
CCDC93	coiled-coil domain containing 93	Ssc.18505.1.A1_at	-1.14 q < 0.00604	-1.13 q < 0.929	-1.25 q < 0.166	1.02 q < 0.935	-1.19 q < 0.892	-1.18 q < 0.676
CCDC93	coiled-coil domain containing 93	Ssc.27636.1.S1_at	-1.12 q < 0.0261	-1.22 q < 0.308	-1.17 q < 0.503	-1.01 q < 0.962	-1.15 q < 1	-1.05 q < 1
DDX19A	DEAD (Asp-Glu-Ala-As) box polypeptide 19A	Ssc.30461.1.A1_at	-1.16 q < 0.00604	-1.08 q < 1	-1 q < 0.997	-1.29 q < 0.111	-1.26 q < 0.489	-1.2 q < 0.676
NCRNA00084	nuclear paraspeckle assembly transcript 1 (non-protein coding)	Ssc.10822.1.S1_at	-1.47 q < 0.00606	-1.61 q < 0.613	-1.15 q < 0.94	-1.12 q < 0.852	-1.67 q < 0.853	-2 q < 0.137
PSD4	pleckstrin and Sec7 domain containing 4	Ssc.26669.1.A1_at	-1.27 q < 0.00607	-1.4 q < 0.449	-1.36 q < 0.443	-1.22 q < 0.553	-1.41 q < 0.694	-1.03 q < 1
SAPS3	protein phosphatase 6, regulatory subunit 3	Ssc.12021.1.S1_at	1.1 q < 0.00611	1.11 q < 0.82	1 q < 0.997	1.11 q < 0.413	1.09 q < 1	1.21 q < 0.0725
APOOL	apolipoprotein O-like	Ssc.8779.1.A1_at	-1.17 q < 0.00613	-1.06 q < 1	-1.29 q < 0.187	-1.14 q < 0.548	-1.23 q < 0.851	-1.14 q < 1
CAMLG	calcium modulating ligand	Ssc.4324.2.A1_at	-1.15 q < 0.00617	-1.26 q < 0.227	-1.22 q < 0.323	-1.09 q < 0.669	-1.08 q < 1	-1.11 q < 1
Q7Z7L8	-	Ssc.17312.1.A1_at	-1.26 q < 0.00644	-1.28 q < 0.796	-1.41 q < 0.287	-1.37 q < 0.248	-1.45 q < 0.423	1.13 q < 1
B3GNTL1	UDP-GlcNAc:betaGal beta-1,3-N-acetylglucosaminyltransferase-like 1	Ssc.19604.1.S1_at	1.12 q < 0.00654	1.19 q < 0.349	1 q < 0.997	1.2 q < 0.152	1.13 q < 1	1.1 q < 1
ZNF652	zinc finger protein 652	Ssc.30952.1.A1_at	1.15 q < 0.00666	1.04 q < 1	1.15 q < 0.629	1.3 q < 0.0603	1.12 q < 1	1.14 q < 1
CK465633	-	Ssc.20531.1.S1_at	-1.19 q < 0.00669	-1.17 q < 0.97	-1.34 q < 0.149	-1.22 q < 0.366	-1.17 q < 1	-1.05 q < 1
COX7B	cytochrome c oxidase subunit VIIb	Ssc.986.1.A1_at	-1.22 q < 0.00676	-1.17 q < 1	-1.35 q < 0.269	-1.24 q < 0.394	-1.08 q < 1	-1.26 q < 0.756
GRHL2	grainyhead-like 2 (Drosophila)	Ssc.18618.1.A1_at	1.15 q < 0.00696	-1.02 q < 1	1.28 q < 0.136	1.2 q < 0.272	1.12 q < 1	1.19 q < 0.686
HLA-A	major histocompatibility complex, class I, A	Ssc.13780.9.S1_a_at	1.21 q < 0.007	1.48 q < 0.0605	1.3 q < 0.372	1.03 q < 0.932	-1.08 q < 1	1.41 q < 0.131
HLA-A	major histocompatibility complex, class I, A	Ssc.13780.3.S1_at	1.14 q < 0.00982	1.32 q < 0.0614	1.04 q < 0.956	1.24 q < 0.156	1.1 q < 1	1.03 q < 1
HLA-A	major histocompatibility complex, class I, A	Ssc.13780.11.S1_x_at	1.16 q < 0.766	1.06 q < 1	-1.46 q < 0.842	4.21 q < 0.00154	-1.2 q < 1	-1.22 q < 1
OXR1	oxidation resistance 1	Ssc.10917.1.A1_at	-1.3 q < 0.007	-1.34 q < 0.767	-1.35 q < 0.556	-1.32 q < 0.416	-1.36 q < 1	-1.14 q < 1
PDGFC	platelet derived growth factor C	Ssc.25067.1.S1_at	-1.25 q < 0.00706	-1.17 q < 1	-1.2 q < 0.802	-1.12 q < 0.744	-1.39 q < 0.607	-1.4 q < 0.372
C18orf18	chromosome 18 open reading frame 18	Ssc.30604.1.S1_at	-1.13 q < 0.0071	-1.26 q < 0.138	-1.16 q < 0.52	-1.14 q < 0.433	-1.17 q < 1	1.04 q < 1
GPX7	glutathione peroxidase 7	Ssc.11374.1.S1_at	-1.21 q < 0.0071	-1.44 q < 0.114	-1.43 q < 0.108	-1.01 q < 0.988	-1.05 q < 1	-1.21 q < 1
HLA-C	major histocompatibility complex, class I, C	Ssc.16756.1.S1_at	-1.2 q < 0.00724	-1.18 q < 0.985	-1.3 q < 0.329	1.03 q < 0.94	-1.26 q < 1	-1.32 q < 0.354
TMEM214	transmembrane protein 214	Ssc.4888.1.S1_at	-1.18 q < 0.00726	-1.29 q < 0.36	-1.39 q < 0.0788	1.03 q < 0.919	-1.18 q < 1	-1.14 q < 1
ZRANB2	zinc finger, RAN-binding domain containing 2	Ssc.23838.1.A1_at	1.21 q < 0.00752	-1.02 q < 1	1.24 q < 0.579	1.24 q < 0.409	1.18 q < 1	1.49 q < 0.0571
FUNDC1	FUN14 domain containing 1	Ssc.5383.1.S1_at	-1.14 q < 0.00753	-1.07 q < 1	-1.15 q < 0.617	-1.08 q < 0.7	-1.18 q < 0.925	-1.21 q < 0.41
CAPN9	calpain 9	Ssc.9446.1.A1_at	-1.16 q < 0.00763	-1.16 q < 0.907	-1.3 q < 0.133	-1.17 q < 0.413	-1.17 q < 1	-1.02 q < 1
ADAMTS1	ADAM metallopeptidase with thrombospondin type 1 motif, 1	Ssc.12365.1.A1_at	-1.37 q < 0.00791	-1.73 q < 0.185	-1.44 q < 0.555	-1.3 q < 0.557	-1.39 q < 1	-1.07 q < 1
ELOVL7	ELOVL family member 7, elongation of long chain fatty acids (yeast)	Ssc.17942.1.A1_at	1.28 q < 0.00793	1.21 q < 1	1.54 q < 0.138	1.54 q < 0.0981	-1.07 q < 1	1.25 q < 1

Gene Name	Gene Description	Probe Set ID	Breed MS-WC	Day 25 MS-WC	Day 45 MS-WC	Day 65 MS-WC	Day 85 MS-WC	Day 105 MS-WC
MMS19	MMS19 nucleotide excision repair homolog (S. cerevisiae)	Ssc.27378.1.S1_at	1.16 q < 0.00798	1.21 q < 0.598	-1.06 q < 0.935	1.33 q < 0.0565	1.24 q < 0.688	1.11 q < 1
DUT	deoxyuridine triphosphatase	Ssc.19928.1.S1_a_at	1.18 q < 0.00804	1.35 q < 0.161	1.16 q < 0.764	1.17 q < 0.486	1.21 q < 1	1.04 q < 1
DNAH14	dynein, axonemal, heavy chain 14	Ssc.10174.1.A1_at	1.16 q < 0.00806	-1 q < 1	1.11 q < 0.859	1.18 q < 0.389	1.24 q < 0.67	1.29 q < 0.199
DNAH14	dynein, axonemal, heavy chain 14	Ssc.30965.1.A1_at	-1.2 q < 0.0213	-1.3 q < 0.598	-1.45 q < 0.135	-1.3 q < 0.323	-1.03 q < 1	-1.01 q < 1
DNAH14	dynein, axonemal, heavy chain 14	Ssc.21309.1.A1_at	1.16 q < 0.128	1.77 q < 0.00542	1.02 q < 0.989	1.45 q < 0.131	-1.2 q < 1	-1.02 q < 1
SGPL1	sphingosine-1-phosphate lyase 1	Ssc.7716.1.A1_at	-1.14 q < 0.0081	-1.28 q < 0.132	-1.02 q < 0.983	-1.14 q < 0.477	-1.28 q < 0.192	-1.02 q < 1
SLC25A34	solute carrier family 25, member 34	Ssc.29286.1.A1_at	1.12 q < 0.0081	1.02 q < 1	1.2 q < 0.253	1.17 q < 0.281	1.16 q < 1	1.08 q < 1
KCTD1	potassium channel tetramerisation domain containing 1	Ssc.11801.1.A1_at	-1.16 q < 0.00821	-1.2 q < 0.671	-1.29 q < 0.171	-1.11 q < 0.622	-1.16 q < 1	-1.06 q < 1
MCCC2	methylcrotonoyl-CoA carboxylase 2 (beta)	Ssc.27572.1.S1_at	1.16 q < 0.00823	1.16 q < 0.921	1.17 q < 0.641	1.26 q < 0.187	1.13 q < 1	1.11 q < 1
SIAE	sialic acid acetylesterase	Ssc.27298.1.A1_at	-1.18 q < 0.00823	-1.22 q < 0.677	-1.21 q < 0.552	-1.31 q < 0.146	-1.15 q < 1	-1.02 q < 1
FASN	fatty acid synthase	Ssc.18175.1.A1_at	1.27 q < 0.00831	1.65 q < 0.0577	-1.12 q < 0.913	1.52 q < 0.11	1.2 q < 1	1.23 q < 1
PAK1	p21 protein (Cdc42/Rac)-activated kinase 1	Ssc.11929.1.A1_at	-1.12 q < 0.00831	-1.13 q < 0.812	-1.23 q < 0.109	-1.03 q < 0.868	-1.15 q < 1	-1.06 q < 1
PAK1	protein kinase N1	Ssc.11929.1.A1_at	-1.12 q < 0.00831	-1.13 q < 0.812	-1.23 q < 0.109	-1.03 q < 0.868	-1.15 q < 1	-1.06 q < 1
Q9H7X0	-	Ssc.5727.1.S1_at	-1.15 q < 0.00832	-1.14 q < 0.995	-1.33 q < 0.0695	-1.04 q < 0.864	-1.16 q < 1	-1.12 q < 1
HADH	hydroxyacyl-CoA dehydrogenase	Ssc.12507.1.S1_at	-1.22 q < 0.00844	-1.11 q < 1	-1.26 q < 0.572	-1.35 q < 0.217	-1.2 q < 1	-1.22 q < 1
GPR37	G protein-coupled receptor 37 (endothelin receptor type B-like)	Ssc.27084.1.S1_at	-1.49 q < 0.00862	-1.41 q < 1	-1.88 q < 0.254	-1.57 q < 0.399	-1.44 q < 1	-1.24 q < 1
IGFBP3	insulin-like growth factor binding protein 3	Ssc.15588.1.S2_at	1.87 q < 0.00866	1.98 q < 0.812	2.55 q < 0.31	3.26 q < 0.0756	1.17 q < 1	1.2 q < 1
CPNE4	copine IV	Ssc.11953.1.A1_at	-1.08 q < 0.0089	-1.08 q < 0.893	-1.05 q < 0.898	-1.14 q < 0.162	-1.08 q < 1	-1.08 q < 1
C5orf34	chromosome 5 open reading frame 34	Ssc.22208.1.A1_at	-1.14 q < 0.00896	-1.21 q < 0.418	-1.12 q < 0.777	-1.09 q < 0.667	-1.21 q < 0.706	-1.08 q < 1
PDCL	phosducin-like	Ssc.5703.1.A1_at	-1.22 q < 0.00896	-1.26 q < 0.736	-1.17 q < 0.819	-1.15 q < 0.643	-1.31 q < 0.861	-1.22 q < 1
GTF3C5	general transcription factor IIIC, polypeptide 5, 63kDa	Ssc.17447.1.A1_at	-1.1 q < 0.00922	-1.08 q < 1	-1.12 q < 0.55	-1.07 q < 0.612	-1.1 q < 1	-1.14 q < 0.622
CD28	CD28 molecule	Ssc.13463.2.S1_at	-1.17 q < 0.00925	-1.03 q < 1	-1.38 q < 0.0582	-1.27 q < 0.197	-1.05 q < 1	-1.15 q < 1
CD28	cytotoxic T-lymphocyte-associated protein 4	Ssc.13463.2.S1_at	-1.17 q < 0.00925	-1.03 q < 1	-1.38 q < 0.0582	-1.27 q < 0.197	-1.05 q < 1	-1.15 q < 1
PLOD1	procollagen-lysine 1, 2-oxoglutarate 5-dioxygenase 1	Ssc.23519.2.S1_at	-1.26 q < 0.00928	-1.35 q < 0.598	-1.57 q < 0.0824	-1.15 q < 0.685	-1.06 q < 1	-1.23 q < 1
RGS11	regulator of G-protein signaling 11	Ssc.5588.1.A1_at	1.11 q < 0.00928	1.09 q < 1	1.05 q < 0.917	1.13 q < 0.368	1.14 q < 1	1.13 q < 0.729
GNPDA1	glucosamine-6-phosphate deaminase 1	Ssc.20238.2.S1_a_at	-1.21 q < 0.00932	-1.08 q < 1	-1.47 q < 0.0721	-1.16 q < 0.585	-1.11 q < 1	-1.27 q < 0.728
PSCD1	cytohesin 1	Ssc.13662.1.A1_at	1.13 q < 0.00938	1.07 q < 1	1.14 q < 0.647	1.12 q < 0.521	1.2 q < 0.685	1.13 q < 1
STBD1	starch binding domain 1	Ssc.13948.1.S1_at	-1.18 q < 0.00944	-1.13 q < 1	-1.21 q < 0.583	-1.21 q < 0.404	-1.21 q < 1	-1.16 q < 1
KITLG	KIT ligand	Ssc.4707.1.A1_at	-1.2 q < 0.00949	-1.05 q < 1	-1.39 q < 0.15	-1.13 q < 0.66	-1.24 q < 1	-1.23 q < 0.872
PSMG4	proteasome (prosome, macropain) assembly chaperone 4	Ssc.15590.1.S1_at	-1.12 q < 0.0095	1.02 q < 1	-1.2 q < 0.265	-1.06 q < 0.769	-1.22 q < 0.415	-1.18 q < 0.49
QSOX1	quiescin Q6 sulfhydryl oxidase 1	Ssc.3249.1.S1_at	-1.33 q < 0.00952	-1.22 q < 1	-1.61 q < 0.196	-1.49 q < 0.251	-1.18 q < 1	-1.19 q < 1
COL12A1	collagen, type XII, alpha 1	Ssc.1049.1.S1_at	-1.47 q < 0.00973	-1.26 q < 1	-1.6 q < 0.522	-1.54 q < 0.406	-1.13 q < 1	-1.95 q < 0.218
C3orf10	chromosome 3 open reading frame 10	Ssc.17314.1.S1_at	-1.15 q < 0.00988	1.02 q < 1	-1.31 q < 0.0947	-1.12 q < 0.552	-1.15 q < 1	-1.2 q < 0.655
THY1	Thy-1 cell surface antigen	Ssc.20133.1.A1_at	2.02 q < 0.0099	1.39 q < 1	2.27 q < 0.561	3.94 q < 0.0643	1.45 q < 1	1.84 q < 1
RNF213	ring finger protein 213	Ssc.30869.1.S1_at	-1.17 q < 0.00995	-1.11 q < 1	-1.33 q < 0.134	-1.14 q < 0.567	-1.04 q < 1	-1.24 q < 0.544
CHD4	chromodomain helicase DNA binding protein 4	Ssc.16672.1.S1_at	-1.11 q < 0.00998	-1.06 q < 1	-1.23 q < 0.103	-1.13 q < 0.395	-1.05 q < 1	-1.11 q < 1
XPO6	exportin 6	Ssc.21575.1.S1_at	-1.15 q < 0.01	-1.05 q < 1	-1.28 q < 0.152	-1.09 q < 0.69	-1.13 q < 1	-1.21 q < 0.549
ZFP1	zinc finger protein 1 homolog (mouse)	Ssc.30576.1.A1_at	1.13 q < 0.0101	1.27 q < 0.0928	1.01 q < 0.988	1.16 q < 0.346	1.1 q < 1	1.1 q < 1
TTC7A	tetratricopeptide repeat domain 7A	Ssc.5883.1.S1_at	-1.13 q < 0.0101	-1.15 q < 0.786	-1 q < 0.997	-1.22 q < 0.179	-1.11 q < 1	-1.19 q < 0.515
ITGA2	integrin, alpha 2 (CD49B, alpha 2 subunit of VLA-2 receptor)	Ssc.16218.1.S1_at	1.2 q < 0.0102	-1.07 q < 1	1.37 q < 0.202	1.3 q < 0.264	1.2 q < 1	1.28 q < 0.623
ITGA2	integrin, alpha 2 (CD49B, alpha 2 subunit of VLA-2 receptor)	Ssc.26342.1.A1_at	1.04 q < 0.717	-1.13 q < 1	-1.04 q < 0.954	1.35 q < 0.0242	1.1 q < 1	-1.01 q < 1
ARHGAP28	Rho GTPase activating protein 28	Ssc.11844.1.A1_at	1.16 q < 0.0102	1.29 q < 0.257	1.26 q < 0.31	1.2 q < 0.381	1.08 q < 1	1.02 q < 1
ARHGAP28	Rho GTPase activating protein 28	Ssc.30073.1.A1_at	1.07 q < 0.423	-1.04 q < 1	1.11 q < 0.843	1.32 q < 0.045	-1.04 q < 1	1.03 q < 1
CYP1B1	cytochrome P450, family 1, subfamily B, polypeptide 1	Ssc.8767.1.A1_at	1.24 q < 0.0104	1.15 q < 1	1.26 q < 0.632	1.52 q < 0.0637	1.29 q < 1	1.02 q < 1
CABLES2	Cdk5 and Abl enzyme substrate 2	Ssc.28609.1.S1_at	1.25 q < 0.0104	1.36 q < 0.515	1.35 q < 0.427	1.26 q < 0.452	1.2 q < 1	1.08 q < 1
SCPEP1	serine carboxypeptidase 1	Ssc.3037.1.S1_at	-1.59 q < 0.0104	-1.74 q < 0.725	-1.38 q < 0.859	1.32 q < 0.698	-2.85 q < 0.0549	-1.95 q < 0.498
PTN	pleiotrophin	Ssc.12975.1.S1_at	-1.58 q < 0.0104	-1.6 q < 0.89	-1.25 q < 0.917	-1.67 q < 0.41	-1.24 q < 1	-2.39 q < 0.132

Gene Name	Gene Description	Probe Set ID	Breed MS-WC	Day 25 MS-WC	Day 45 MS-WC	Day 65 MS-WC	Day 85 MS-WC	Day 105 MS-WC
AQP1	aquaporin 1 (Colton blood group)	Ssc.27596.1.S1_at	-1.3 q < 0.0105	1.09 q < 1	-1.52 q < 0.251	-1.52 q < 0.166	-1.23 q < 1	-1.4 q < 0.682
TJP2	tight junction protein 2 (zona occludens 2)	Ssc.23465.1.S1_at	-1.15 q < 0.0105	-1.07 q < 1	-1.11 q < 0.842	-1.15 q < 0.477	-1.36 q < 0.0642	-1.08 q < 1
KLHDC2	kelch domain containing 2	Ssc.11035.3.A1_at	1.13 q < 0.0106	1.05 q < 1	1.18 q < 0.464	1.23 q < 0.145	1.12 q < 1	1.1 q < 1
SELI	ethanolaminephosphotransferase 1 (CDP-ethanolamine-specific)	Ssc.27288.1.S1_at	1.17 q < 0.0106	1.16 q < 0.936	-1.04 q < 0.967	1.37 q < 0.0545	1.26 q < 0.714	1.12 q < 1
TRIM26	tripartite motif-containing 26	Ssc.20734.2.S1_at	1.13 q < 0.0108	1.03 q < 1	1.05 q < 0.938	1.23 q < 0.124	1.23 q < 0.409	1.11 q < 1
CPD	carboxypeptidase D	Ssc.1116.1.S1_at	-1.21 q < 0.0112	-1.38 q < 0.284	-1.29 q < 0.477	-1.1 q < 0.755	-1.2 q < 1	-1.13 q < 1
CPD	homer homolog 2 (Drosophila)	Ssc.1116.1.S1_at	-1.21 q < 0.0112	-1.38 q < 0.284	-1.29 q < 0.477	-1.1 q < 0.755	-1.2 q < 1	-1.13 q < 1
C1orf31	chromosome 1 open reading frame 31	Ssc.26398.2.S1_a_at	1.14 q < 0.0112	1.32 q < 0.0523	1.07 q < 0.911	1.01 q < 0.979	1.15 q < 1	1.16 q < 0.894
GLTSCR2	glioma tumor suppressor candidate region gene 2	Ssc.20716.1.S1_at	-1.12 q < 0.0113	-1.06 q < 1	-1.14 q < 0.584	-1.19 q < 0.199	-1.07 q < 1	-1.14 q < 0.81
ISCA1	iron-sulfur cluster assembly 1 homolog (S. cerevisiae)	Ssc.1940.1.S1_a_at	1.21 q < 0.0113	1.48 q < 0.104	1.1 q < 0.913	1.18 q < 0.554	1.16 q < 1	1.19 q < 1
ZC3H12D	zinc finger CCCH-type containing 12D	Ssc.30602.1.A1_at	1.21 q < 0.0115	1.09 q < 1	1.48 q < 0.0693	1.45 q < 0.0644	1.11 q < 1	-1.01 q < 1
AHCTF1P	AT hook containing transcription factor 1 pseudogene 1	Ssc.27942.1.A1_at	-1.14 q < 0.0117	-1.08 q < 1	-1.26 q < 0.147	-1.03 q < 0.904	-1.16 q < 1	-1.17 q < 0.753
CACNA2D4	calcium channel, voltage-dependent, alpha 2/delta subunit 4	Ssc.28045.1.A1_at	-1.13 q < 0.0117	-1.08 q < 1	-1.24 q < 0.177	-1.22 q < 0.17	-1.14 q < 1	1.01 q < 1
PEG3	paternally expressed 3	Ssc.22623.2.S1_at	-1.33 q < 0.0118	-1.15 q < 1	-1.49 q < 0.411	-1.64 q < 0.131	-1.18 q < 1	-1.26 q < 1
PEG3	paternally expressed 3	Ssc.10530.1.A1_at	-1.33 q < 0.0235	-1.17 q < 1	-1.51 q < 0.448	-1.61 q < 0.2	-1.24 q < 1	-1.18 q < 1
SDC3	syndecan 3	Ssc.6589.1.S1_a_at	-1.18 q < 0.0119	-1.24 q < 0.664	-1.13 q < 0.855	-1.17 q < 0.509	-1.04 q < 1	-1.35 q < 0.201
DNAL4	dynein, axonemal, light chain 4	Ssc.5303.1.S1_at	-1.14 q < 0.012	-1.12 q < 1	-1.2 q < 0.391	-1.06 q < 0.766	-1.18 q < 1	-1.12 q < 1
C15orf33	chromosome 15 open reading frame 33	Ssc.26783.1.S1_at	-1.12 q < 0.012	-1.18 q < 0.527	-1.16 q < 0.5	-1.07 q < 0.712	-1.11 q < 1	-1.11 q < 1
RIMBP2	RIMS binding protein 2	Ssc.28484.1.S1_at	-1.15 q < 0.012	-1.18 q < 0.715	-1.11 q < 0.84	-1.05 q < 0.846	-1.22 q < 0.724	-1.17 q < 0.843
Q7L3Y3	-	Ssc.21934.1.A1_at	-1.12 q < 0.0122	-1.14 q < 0.72	-1.11 q < 0.763	-1.07 q < 0.67	-1.14 q < 1	-1.12 q < 1
ZADH2	zinc binding alcohol dehydrogenase domain containing 2	Ssc.11794.1.A1_at	-1.16 q < 0.0123	-1.07 q < 1	-1.18 q < 0.661	-1.22 q < 0.313	-1.31 q < 0.348	-1.06 q < 1
SNORD107	small nucleolar RNA, C/D box 107	Ssc.7020.1.A1_at	1.18 q < 0.0124	1.22 q < 0.727	-1.05 q < 0.953	1.3 q < 0.187	1.13 q < 1	1.33 q < 0.25
SLC44A2	solute carrier family 44, member 2	Ssc.26850.1.S1_at	1.14 q < 0.0124	1.14 q < 0.949	1.13 q < 0.766	1.15 q < 0.458	1.18 q < 1	1.12 q < 1
FSHB	follicle stimulating hormone, beta polypeptide	Ssc.5833.1.A1_at	1.13 q < 0.0125	1.16 q < 0.767	1.11 q < 0.811	1.14 q < 0.426	1.11 q < 1	1.13 q < 1
C14orf65	entry withdrawn	Ssc.26910.1.S1_at	1.12 q < 0.0128	1.22 q < 0.252	1.06 q < 0.92	1.12 q < 0.507	1.02 q < 1	1.2 q < 0.366
TEX2	testis expressed 2	Ssc.11043.1.A1_at	1.39 q < 0.0129	1.61 q < 0.502	1.41 q < 0.69	-1.06 q < 0.931	2.12 q < 0.063	1.15 q < 1
TEX2	testis expressed 2	Ssc.11043.2.A1_at	1.16 q < 0.0191	1.18 q < 0.893	1.14 q < 0.808	-1 q < 0.999	1.37 q < 0.183	1.16 q < 1
SDCCAG10	CWC27 spliceosome-associated protein homolog (S. cerevisiae)	Ssc.10698.1.S1_at	-1.09 q < 0.0131	-1.04 q < 1	-1.15 q < 0.265	-1.08 q < 0.557	-1.09 q < 1	-1.11 q < 0.852
RBMS2P	RNA binding motif, single stranded interacting protein 2 pseudogene 1	Ssc.21723.1.S1_at	-1.14 q < 0.0132	-1.29 q < 0.144	-1.15 q < 0.656	-1.07 q < 0.755	-1.04 q < 1	-1.16 q < 0.875
EIF4B	eukaryotic translation initiation factor 4B	Ssc.18323.1.S1_at	-1.1 q < 0.0133	-1.07 q < 1	-1.2 q < 0.0897	-1 q < 0.985	-1.13 q < 1	-1.09 q < 1
XKRX	XK, Kell blood group complex subunit-related, X-linked	Ssc.3376.1.S1_at	-1.25 q < 0.0133	1.09 q < 1	-1.55 q < 0.0983	-1.2 q < 0.587	-1.39 q < 0.75	-1.28 q < 0.926
DGKI	diacylglycerol kinase, iota	Ssc.30272.1.A1_at	-1.18 q < 0.0134	-1.03 q < 1	-1.17 q < 0.749	-1.26 q < 0.288	-1.35 q < 0.331	-1.11 q < 1
FAM127A	family with sequence similarity 127, member A	Ssc.4896.1.A1_at	-1.17 q < 0.0134	-1.2 q < 0.787	-1.4 q < 0.0637	-1.02 q < 0.945	-1.17 q < 1	-1.09 q < 1
GNPTAB	N-acetylglucosamine-1-phosphate transferase, alpha and beta subunits	Ssc.10082.1.A1_at	-1.22 q < 0.0135	-1.08 q < 1	-1.16 q < 0.846	-1.25 q < 0.438	-1.41 q < 0.454	-1.24 q < 0.971
CYP17A1	cytochrome P450, family 17, subfamily A, polypeptide 1	Ssc.15060.1.S1_at	1.5 q < 0.0136	1.26 q < 1	-1.01 q < 0.997	1.52 q < 0.476	1.68 q < 1	2.41 q < 0.0631
MMRN1	multimerin 1	Ssc.14368.1.A1_at	-1.4 q < 0.0136	-1.02 q < 1	-1.54 q < 0.525	-1.7 q < 0.209	-1.84 q < 0.358	-1.11 q < 1
TTC31	tetratricopeptide repeat domain 31	Ssc.29637.1.A1_at	-1.11 q < 0.0137	-1.07 q < 1	-1.22 q < 0.148	1.01 q < 0.971	-1.16 q < 0.882	-1.14 q < 0.844
GPR177	wntless homolog (Drosophila)	Ssc.9713.1.A1_at	1.16 q < 0.0138	1.08 q < 1	1.29 q < 0.208	1.17 q < 0.45	1.06 q < 1	1.21 q < 0.695
ZNF783	zinc finger family member 783	Ssc.18236.1.S1_at	-1.12 q < 0.0138	-1.06 q < 1	-1.23 q < 0.166	-1.08 q < 0.647	-1.13 q < 1	-1.11 q < 1
TNNC1	troponin C type 1 (slow)	Ssc.16889.1.S1_at	-1.27 q < 0.0139	-1.23 q < 1	-1.46 q < 0.298	-1.56 q < 0.113	-1.04 q < 1	-1.15 q < 1
TNNC1	troponin I type 3 (cardiac)	Ssc.16889.1.S1_at	-1.27 q < 0.0139	-1.23 q < 1	-1.46 q < 0.298	-1.56 q < 0.113	-1.04 q < 1	-1.15 q < 1
NP_056245	-	Ssc.15541.1.A1_at	-1.24 q < 0.014	-1.22 q < 1	-1.15 q < 0.885	-1.16 q < 0.663	-1.15 q < 1	-1.59 q < 0.0709
SLC16A10	solute carrier family 16, member 10 (aromatic amino acid transporter)	Ssc.26633.1.A1_at	-1.21 q < 0.0141	-1.54 q < 0.0501	-1.36 q < 0.287	1.27 q < 0.365	-1.45 q < 0.239	-1.09 q < 1
MRC1L1	mannose receptor, C type 1-like 1	Ssc.9229.1.S1_at	-1.45 q < 0.0142	-1 q < 1	-1.5 q < 0.662	-1.35 q < 0.596	-1.42 q < 1	-2.26 q < 0.0594
FBXO22	F-box protein 22	Ssc.20801.1.S1_at	-1.13 q < 0.0142	-1.17 q < 0.74	-1.27 q < 0.129	-1.2 q < 0.269	-1.02 q < 1	-1.04 q < 1

Gene Name	Gene Description	Probe Set ID	Breed MS-WC	Day 25 MS-WC	Day 45 MS-WC	Day 65 MS-WC	Day 85 MS-WC	Day 105 MS-WC
MBNL2	muscleblind-like 2 (Drosophila)	Ssc.8655.1.A1_at	-1.18 q < 0.0145	-1.32 q < 0.325	-1.36 q < 0.159	1.01 q < 0.981	-1.17 q < 1	-1.11 q < 1
SH3BGRL3	SH3 domain binding glutamic acid-rich protein like 3	Ssc.1403.1.S1_at	-1.19 q < 0.0146	-1.04 q < 1	-1.4 q < 0.12	-1.19 q < 0.496	-1.09 q < 1	-1.27 q < 0.665
COQ6	coenzyme Q6 homolog, monooxygenase (S. cerevisiae)	Ssc.20196.2.S1_at	1.15 q < 0.0146	1.03 q < 1	1.14 q < 0.73	1.33 q < 0.054	1.12 q < 1	1.13 q < 1
COQ6	coenzyme Q6 homolog, monooxygenase (S. cerevisiae)	Ssc.2294.1.S1_a_at	1.15 q < 0.0283	1.04 q < 1	1.13 q < 0.828	1.26 q < 0.246	1.2 q < 1	1.14 q < 1
LGALS1	lectin, galactoside-binding, soluble, 1	Ssc.1320.1.A1_at	-1.3 q < 0.0146	-1.16 q < 1	-1.18 q < 0.886	-1.33 q < 0.449	-1.2 q < 1	-1.7 q < 0.111
ZNF622	zinc finger protein 622	Ssc.19538.1.A1_at	1.16 q < 0.0147	1.32 q < 0.177	1.01 q < 0.997	1.26 q < 0.212	1.07 q < 1	1.17 q < 0.979
CEP250	centrosomal protein 250kDa	Ssc.27568.1.S1_at	-1.12 q < 0.0147	-1.01 q < 1	-1.25 q < 0.096	-1.18 q < 0.258	-1.06 q < 1	-1.11 q < 1
FAM114A1	family with sequence similarity 114, member A1	Ssc.19323.1.S1_at	-1.15 q < 0.0147	-1.09 q < 1	-1.21 q < 0.447	-1.1 q < 0.65	-1.1 q < 1	-1.24 q < 0.433
FAM114A1	family with sequence similarity 114, member A1	Ssc.19323.2.S1_at	-1.14 q < 0.0199	-1.22 q < 0.503	-1.24 q < 0.308	-1.07 q < 0.76	-1.06 q < 1	-1.13 q < 1
ATP2B2	ATPase, Ca++ transporting, plasma membrane 2	Ssc.25241.1.S1_at	1.23 q < 0.0147	1.1 q < 1	1.23 q < 0.741	1.38 q < 0.231	1.15 q < 1	1.34 q < 0.608
ATP2B2	ATPase, Ca++ transporting, plasma membrane 2	Ssc.10127.1.A1_at	1.18 q < 0.034	1.07 q < 1	1.19 q < 0.747	1.22 q < 0.449	1.1 q < 1	1.34 q < 0.406
ATP2B2	ATPase, Ca++ transporting, plasma membrane 4	Ssc.25241.1.S1_at	1.23 q < 0.0147	1.1 q < 1	1.23 q < 0.741	1.38 q < 0.231	1.15 q < 1	1.34 q < 0.608
ATP2B2	ATPase, Ca++ transporting, plasma membrane 4	Ssc.10127.1.A1_at	1.18 q < 0.034	1.07 q < 1	1.19 q < 0.747	1.22 q < 0.449	1.1 q < 1	1.34 q < 0.406
AGA	aspartylglucosaminidase	Ssc.27438.1.S1_at	-1.28 q < 0.0149	-1.14 q < 1	-1.28 q < 0.708	-1.11 q < 0.804	-1.44 q < 0.785	-1.46 q < 0.434
BACE1	beta-site APP-cleaving enzyme 1	Ssc.880.1.A1_at	-1.1 q < 0.0149	-1.22 q < 0.121	-1.07 q < 0.878	-1.08 q < 0.585	-1.16 q < 0.73	1 q < 1
AP3B1	adaptor-related protein complex 3, beta 1 subunit	Ssc.4666.1.A1_at	1.16 q < 0.015	1.29 q < 0.264	1 q < 0.999	1.23 q < 0.288	1.15 q < 1	1.14 q < 1
PPP1CA	protein phosphatase 1, catalytic subunit, alpha isozyme	Ssc.21716.1.A1_at	1.27 q < 0.015	1.25 q < 1	1.27 q < 0.702	1.62 q < 0.0635	1.2 q < 1	1.05 q < 1
SOX5	SRY (sex determining region Y)-box 5	Ssc.22778.1.S1_at	1.15 q < 0.0151	1.19 q < 0.76	1.05 q < 0.947	1.29 q < 0.126	1.2 q < 1	1.05 q < 1
ITPK1	inositol 1,3,4-triphosphate 5/6 kinase	Ssc.24396.1.S1_at	1.14 q < 0.0151	1.18 q < 0.731	1.12 q < 0.816	1.27 q < 0.122	1.1 q < 1	1.05 q < 1
ARL4A	ADP-ribosylation factor-like 4A	Ssc.30613.1.A1_at	-1.15 q < 0.0151	-1.34 q < 0.104	-1.16 q < 0.704	-1.18 q < 0.429	-1.06 q < 1	-1.05 q < 1
C10orf68	chromosome 10 open reading frame 68	Ssc.4035.3.S1_at	1.14 q < 0.0151	1.35 q < 0.0501	-1.05 q < 0.943	1.3 q < 0.0714	1.18 q < 1	-1.03 q < 1
HSF4	heat shock transcription factor 4	Ssc.9258.1.A1_at	-1.12 q < 0.0151	-1.11 q < 1	-1.18 q < 0.403	-1.04 q < 0.834	-1.31 q < 0.0501	1.01 q < 1
SUV420H1	suppressor of variegation 4-20 homolog 1 (Drosophila)	Ssc.5835.1.S1_at	-1.12 q < 0.0151	1.02 q < 1	-1.21 q < 0.245	-1.18 q < 0.259	-1.15 q < 1	-1.09 q < 1
MEGF11	multiple EGF-like-domains 11	Ssc.12456.1.A1_at	1.29 q < 0.0153	1.68 q < 0.113	-1.04 q < 0.982	1.69 q < 0.0635	-1 q < 1	1.31 q < 0.991
C8orf33	chromosome 8 open reading frame 33	Ssc.3829.1.S1_a_at	-1.13 q < 0.0154	-1 q < 1	-1.23 q < 0.216	-1.11 q < 0.566	-1.11 q < 1	-1.2 q < 0.509
CDH11	cadherin 11, type 2, OB-cadherin (osteoblast)	Ssc.8698.1.S1_at	-1.32 q < 0.0157	-1.1 q < 1	-1.57 q < 0.28	-1.49 q < 0.276	-1.13 q < 1	-1.38 q < 0.901
GNL2	guanine nucleotide binding protein-like 2 (nucleolar)	Ssc.25247.1.S1_at	-1.13 q < 0.0158	-1.11 q < 1	-1.1 q < 0.822	-1.16 q < 0.365	-1.16 q < 1	-1.1 q < 1
RPL13A	ribosomal protein L13a	Ssc.17024.1.A1_at	-1.09 q < 0.0158	1.02 q < 1	-1.22 q < 0.0516	-1.06 q < 0.687	-1.04 q < 1	-1.18 q < 0.18
NT5DC2	5'-nucleotidase domain containing 2	Ssc.5735.1.A1_at	-1.36 q < 0.0158	-1.14 q < 1	-1.48 q < 0.535	-1.48 q < 0.357	-1.17 q < 1	-1.6 q < 0.479
FSTL4	follistatin-like 4	Ssc.11743.1.S1_at	-1.2 q < 0.0162	-1.09 q < 1	-1.09 q < 0.93	-1.07 q < 0.827	-1.34 q < 0.641	-1.48 q < 0.0843
SKAP1	src kinase associated phosphoprotein 1	Ssc.4835.1.S1_at	-1.13 q < 0.0162	-1.21 q < 0.464	-1.23 q < 0.273	1.02 q < 0.951	-1.08 q < 1	-1.18 q < 0.683
NUP93	nucleoporin 93kDa	Ssc.5852.1.A1_at	1.21 q < 0.0164	1.21 q < 0.991	1.01 q < 0.997	1.43 q < 0.114	1.26 q < 1	1.2 q < 1
GLUL	glutamate-ammonia ligase	Ssc.9637.1.S1_at	1.36 q < 0.0164	1.96 q < 0.0698	-1.08 q < 0.967	-1.01 q < 0.991	1.83 q < 0.231	1.4 q < 0.964
XRCC6	X-ray repair complementing defective repair in Chinese hamster cells 6	Ssc.15663.1.S1_at	-1.12 q < 0.0168	-1.08 q < 1	-1.24 q < 0.148	-1.04 q < 0.842	-1.08 q < 1	-1.17 q < 0.618
ACOT1	acyl-CoA thioesterase 1	Ssc.18475.2.S1_at	1.19 q < 0.0168	1.47 q < 0.0617	1.07 q < 0.94	1.23 q < 0.387	1.08 q < 1	1.13 q < 1
Q9HCM6	-	Ssc.24513.1.S1_at	1.17 q < 0.017	1.09 q < 1	1.2 q < 0.607	1.15 q < 0.539	1.15 q < 1	1.24 q < 0.609
Q9HCM6	-	Ssc.1118.1.A1_at	1.18 q < 0.0327	1.04 q < 1	1.09 q < 0.917	1.38 q < 0.122	1.13 q < 1	1.28 q < 0.655
TMTC3	transmembrane and tetratricopeptide repeat containing 3	Ssc.11289.1.A1_at	-1.12 q < 0.017	-1.17 q < 0.596	-1.07 q < 0.908	-1.04 q < 0.831	-1.21 q < 0.544	-1.12 q < 1
C18orf32	chromosome 18 open reading frame 32	Ssc.9968.1.S1_at	1.22 q < 0.017	1.25 q < 0.829	-1.01 q < 0.994	1.5 q < 0.0646	1.26 q < 1	1.14 q < 1
BIN2	bridging integrator 2	Ssc.4283.1.S1_at	-1.2 q < 0.0171	-1.09 q < 1	-1.15 q < 0.849	-1.38 q < 0.127	-1.15 q < 1	-1.23 q < 0.917
ST6GALNAC2	ST6 (alpha-N-acetyl-neuraminyl-2,3-beta-galactosyl-1,3)-N-acetylgalactosaminide alpha-2,6-sialyltransferase 2	Ssc.7269.1.A1_at	-1.14 q < 0.0172	-1.3 q < 0.118	-1.15 q < 0.696	1.04 q < 0.852	-1.15 q < 1	-1.16 q < 0.958
PIK3C2A	phosphoinositide-3-kinase, class 2, alpha polypeptide	Ssc.13442.1.S1_at	-1.2 q < 0.0174	-1.33 q < 0.416	-1.23 q < 0.645	-1.06 q < 0.846	-1.23 q < 1	-1.16 q < 1
MXRA7	matrix-remodelling associated 7	Ssc.19282.1.S1_at	-1.18 q < 0.0176	-1.2 q < 0.854	-1.21 q < 0.626	-1.08 q < 0.78	-1.23 q < 1	-1.17 q < 1
FABP4	fatty acid binding protein 4, adipocyte	Ssc.1089.1.S1_at	-1.31 q < 0.0177	-1.03 q < 1	-1.11 q < 0.942	-1.71 q < 0.0847	-1.28 q < 1	-1.56 q < 0.389
HK1	hexokinase 1	Ssc.5615.1.S1_at	1.18 q < 0.0178	1.16 q < 1	1.15 q < 0.81	1.13 q < 0.645	1.32 q < 0.551	1.15 q < 1
HK1	potassium voltage-gated channel, shaker-related subfamily, member 4	Ssc.5615.1.S1_at	1.18 q < 0.0178	1.16 q < 1	1.15 q < 0.81	1.13 q < 0.645	1.32 q < 0.551	1.15 q < 1

Gene Name	Gene Description	Probe Set ID	Breed MS-WC	Day 25 MS-WC	Day 45 MS-WC	Day 65 MS-WC	Day 85 MS-WC	Day 105 MS-WC
HK1	hook homolog 1 (Drosophila)	Ssc.5615.1.S1_at	1.18 q < 0.0178	1.16 q < 1	1.15 q < 0.81	1.13 q < 0.645	1.32 q < 0.551	1.15 q < 1
UTP3	UTP3, small subunit (SSU) processome component, homolog (S. cerevisiae)	Ssc.9528.1.S1_at	-1.13 q < 0.0179	-1.13 q < 0.972	-1.18 q < 0.465	-1.15 q < 0.421	-1.08 q < 1	-1.11 q < 1
EXOC7	exocyst complex component 7	Ssc.22063.1.S1_at	-1.19 q < 0.0179	-1.06 q < 1	-1.24 q < 0.57	-1.19 q < 0.497	-1.33 q < 0.607	-1.15 q < 1
PPAP2B	phosphatidic acid phosphatase type 2B	Ssc.6233.1.S1_at	-1.15 q < 0.0179	-1.26 q < 0.369	-1.28 q < 0.22	-1.04 q < 0.87	-1.16 q < 1	-1.04 q < 1
RRAGD	Ras-related GTP binding D	Ssc.25575.1.S1_at	-1.16 q < 0.0184	-1.07 q < 1	-1.23 q < 0.447	-1.24 q < 0.277	-1.29 q < 0.454	1.01 q < 1
RRAGD	Ras-related GTP binding D	Ssc.15466.1.S1_at	-1.18 q < 0.028	-1.02 q < 1	-1.36 q < 0.204	-1.21 q < 0.457	-1.36 q < 0.423	1.01 q < 1
AOX1	aldehyde oxidase 1	Ssc.18611.1.A1_at	-1.14 q < 0.0187	-1.14 q < 0.991	-1.33 q < 0.0682	-1.09 q < 0.698	-1.11 q < 1	-1.06 q < 1
CRADD	CASP2 and RIPK1 domain containing adaptor with death domain	Ssc.17839.2.A1_at	-1.13 q < 0.0188	-1.13 q < 0.941	-1.05 q < 0.943	-1.11 q < 0.586	-1.25 q < 0.389	-1.12 q < 1
ERCC5	excision repair cross-complementing rodent repair deficiency, complementation group 5	Ssc.6879.1.A1_at	1.18 q < 0.0189	1.01 q < 1	-1.03 q < 0.977	1.36 q < 0.116	1.19 q < 1	1.43 q < 0.0744
PREX	-	Ssc.10373.1.S1_at	-1.2 q < 0.0189	-1.16 q < 1	-1.16 q < 0.842	-1.19 q < 0.526	-1.2 q < 1	-1.3 q < 0.6
EIF2AK1	eukaryotic translation initiation factor 2-alpha kinase 1	Ssc.9900.1.A1_at	-1.13 q < 0.0191	-1.06 q < 1	-1.24 q < 0.195	-1.06 q < 0.759	-1.17 q < 1	-1.11 q < 1
EIF2AK1	eukaryotic translation initiation factor 2-alpha kinase 2	Ssc.9900.1.A1_at	-1.13 q < 0.0191	-1.06 q < 1	-1.24 q < 0.195	-1.06 q < 0.759	-1.17 q < 1	-1.11 q < 1
C15orf29	chromosome 15 open reading frame 29	Ssc.14780.1.S1_at	-1.16 q < 0.0192	-1.32 q < 0.221	-1.26 q < 0.332	1.01 q < 0.985	-1.2 q < 1	-1.05 q < 1
CDC10	septin 7	Ssc.1837.1.A1_at	-1.17 q < 0.0192	-1.08 q < 1	-1.29 q < 0.314	-1.15 q < 0.548	-1.16 q < 1	-1.17 q < 1
SIRT5	sirtuin 5	Ssc.15393.1.S1_at	-1.15 q < 0.0192	-1.07 q < 1	-1.34 q < 0.0786	-1 q < 0.999	-1.17 q < 1	-1.2 q < 0.768
DOLPP1	dolichyl pyrophosphate phosphatase 1	Ssc.19574.1.A1_at	1.11 q < 0.0193	1.16 q < 0.537	-1.01 q < 0.988	1.22 q < 0.0913	1.11 q < 1	1.07 q < 1
MYH10	myosin, heavy chain 10, non-muscle	Ssc.20017.1.A1_at	-1.12 q < 0.0193	1.03 q < 1	-1.11 q < 0.752	-1.2 q < 0.176	-1.08 q < 1	-1.24 q < 0.151
NRG3	neuregulin 3	Ssc.13323.1.A1_at	1.17 q < 0.0193	1.32 q < 0.273	-1.02 q < 0.99	1.26 q < 0.278	1.09 q < 1	1.23 q < 0.747
PRRX2	paired related homeobox 2	Ssc.17912.1.A1_s_at	-1.13 q < 0.0194	-1.19 q < 0.62	-1.06 q < 0.931	-1.15 q < 0.46	-1.01 q < 1	-1.28 q < 0.138
FZD5	frizzled homolog 5 (Drosophila)	Ssc.29483.1.A1_at	1.25 q < 0.0195	1.44 q < 0.389	1.16 q < 0.885	1.28 q < 0.469	1.03 q < 1	1.4 q < 0.544
CNOT1	CCR4-NOT transcription complex, subunit 1	Ssc.4409.1.A1_at	1.14 q < 0.0195	1.08 q < 1	1.08 q < 0.911	1.25 q < 0.187	1.15 q < 1	1.17 q < 0.912
IFNA8	interferon, alpha 8	Ssc.4815.1.A1_at	-1.14 q < 0.0195	-1.05 q < 1	-1.16 q < 0.665	-1.22 q < 0.268	-1.16 q < 1	-1.14 q < 1
C6orf29	solute carrier family 44, member 4	Ssc.4668.1.A1_at	1.34 q < 0.0196	1.58 q < 0.446	1.43 q < 0.583	1.24 q < 0.647	1.19 q < 1	1.3 q < 1
UGT2A1	UDP glucuronosyltransferase 2 family, polypeptide A1	Ssc.21161.1.S1_at	-1.16 q < 0.0198	-1.09 q < 1	-1.23 q < 0.459	-1 q < 0.993	-1.42 q < 0.0756	-1.11 q < 1
USP10	ubiquitin specific peptidase 10	Ssc.10775.1.A1_at	1.12 q < 0.0199	1.22 q < 0.281	1.08 q < 0.861	1.14 q < 0.393	1 q < 1	1.14 q < 0.876
USP10	ubiquitin specific peptidase 10	Ssc.14327.1.A1_at	1.07 q < 0.426	1.05 q < 1	-1.14 q < 0.662	1.15 q < 0.429	-1.06 q < 1	1.38 q < 0.0114
USP10	ubiquitin specific peptidase 32	Ssc.10775.1.A1_at	1.12 q < 0.0199	1.22 q < 0.281	1.08 q < 0.861	1.14 q < 0.393	1 q < 1	1.14 q < 0.876
USP10	ubiquitin specific peptidase 32	Ssc.14327.1.A1_at	1.07 q < 0.426	1.05 q < 1	-1.14 q < 0.662	1.15 q < 0.429	-1.06 q < 1	1.38 q < 0.0114
OR52I2	olfactory receptor, family 52, subfamily I, member 2	Ssc.24960.1.S1_at	-1.11 q < 0.02	-1.12 q < 0.925	-1.06 q < 0.903	-1.04 q < 0.831	-1.14 q < 1	-1.19 q < 0.352
FAM84B	family with sequence similarity 84, member B	Ssc.1831.2.S1_at	-1.26 q < 0.0201	-1.18 q < 1	-1.28 q < 0.705	-1.24 q < 0.546	-1.37 q < 1	-1.23 q < 1
TRAPPC4	trafficking protein particle complex 4	Ssc.16983.1.S1_at	1.14 q < 0.0202	1.17 q < 0.829	1.03 q < 0.971	1.29 q < 0.122	1.22 q < 0.862	1.03 q < 1
SFRS11	serine/arginine-rich splicing factor 11	Ssc.8959.1.A1_at	1.13 q < 0.0202	1 q < 1	1.07 q < 0.918	1.28 q < 0.102	1.07 q < 1	1.29 q < 0.136
TOMM40L	translocase of outer mitochondrial membrane 40 homolog (yeast)-like	Ssc.18758.1.S1_at	1.11 q < 0.0204	1.12 q < 0.901	1.09 q < 0.844	1.17 q < 0.251	1.06 q < 1	1.12 q < 1
KIAA0368	KIAA0368	Ssc.18221.1.S1_at	-1.15 q < 0.0204	-1.07 q < 1	-1.23 q < 0.366	-1.12 q < 0.612	-1.24 q < 0.716	-1.08 q < 1
NQO1	NAD(P)H dehydrogenase, quinone 1	Ssc.1142.1.S1_at	1.18 q < 0.0205	1.47 q < 0.0529	1.12 q < 0.883	1.14 q < 0.611	1.04 q < 1	1.17 q < 1
CCNK	cyclin K	Ssc.5131.2.A1_at	1.14 q < 0.0205	1.1 q < 1	1.08 q < 0.907	1.3 q < 0.0864	1.11 q < 1	1.13 q < 1
Q6ZW51	-	Ssc.2492.2.S1_at	-1.14 q < 0.0205	-1.12 q < 1	-1.18 q < 0.584	-1.05 q < 0.825	-1.2 q < 1	-1.16 q < 0.969
Q6ZW51	-	Ssc.2492.1.A1_at	-1.18 q < 0.0306	-1.01 q < 1	-1.25 q < 0.554	1.02 q < 0.954	-1.42 q < 0.223	-1.3 q < 0.529
RPL36AL	ribosomal protein L36a-like	Ssc.2707.1.S1_at	-1.13 q < 0.0205	1.04 q < 1	-1.31 q < 0.0705	-1.01 q < 0.973	-1.12 q < 1	-1.29 q < 0.116
TAX1BP1	Tax1 (human T-cell leukemia virus type I) binding protein 1	Ssc.15643.1.S1_at	-1.15 q < 0.0208	-1.14 q < 1	-1.2 q < 0.529	-1.11 q < 0.632	-1.19 q < 1	-1.11 q < 1
TMEM209	transmembrane protein 209	Ssc.11253.1.A1_at	1.14 q < 0.0208	1.1 q < 1	1.08 q < 0.898	1.3 q < 0.0847	1.17 q < 1	1.05 q < 1
PLXNC1	plexin C1	Ssc.25153.1.A1_at	1.13 q < 0.021	1.25 q < 0.247	-1.02 q < 0.977	1.29 q < 0.0658	-1.01 q < 1	1.17 q < 0.732
PLXNC1	plexin C1	Ssc.3879.1.S1_at	-1.12 q < 0.408	-1.07 q < 1	-1.07 q < 0.956	1.05 q < 0.891	1.02 q < 1	-1.66 q < 0.0322
Q8TAL6	-	Ssc.2104.1.S1_at	-1.38 q < 0.021	-1.04 q < 1	-2.01 q < 0.0807	-1.28 q < 0.634	-1.28 q < 1	-1.47 q < 0.92
Q9P273	-	Ssc.27124.1.A1_at	-1.19 q < 0.021	-1.39 q < 0.243	-1.01 q < 0.997	-1.1 q < 0.75	-1.09 q < 1	-1.45 q < 0.111
ZNF146	zinc finger protein 146	Ssc.6436.2.S1_at	-1.15 q < 0.021	-1.19 q < 0.78	-1.26 q < 0.29	-1.13 q < 0.569	-1.05 q < 1	-1.13 q < 1
MAN2A1	mannosidase, alpha, class 2A, member 1	Ssc.10976.1.S1_at	1.09 q < 0.021	-1.01 q < 1	1.08 q < 0.81	1.07 q < 0.633	1.23 q < 0.0593	1.1 q < 1

Gene Name	Gene Description	Probe Set ID	Breed MS-WC	Day 25 MS-WC	Day 45 MS-WC	Day 65 MS-WC	Day 85 MS-WC	Day 105 MS-WC
FOXN3	forkhead box N3	Ssc.24976.1.S1_at	-1.18 q < 0.0211	-1.12 q < 1	-1.32 q < 0.296	-1.11 q < 0.696	-1.27 q < 0.948	-1.1 q < 1
NAP1L1	nucleosome assembly protein 1-like 1	Ssc.1429.1.A1_at	-1.24 q < 0.0212	-1.39 q < 0.471	-1.19 q < 0.83	-1.14 q < 0.71	-1.2 q < 1	-1.28 q < 0.956
NAP1L1	nucleosome assembly protein 1-like 1	Ssc.3802.1.S1_at	-1.23 q < 0.0311	-1.05 q < 1	-1.34 q < 0.495	-1.17 q < 0.651	-1.36 q < 0.964	-1.24 q < 1
TFE3	transcription factor binding to IGHM enhancer 3	Ssc.4131.1.S1_at	-1.13 q < 0.0212	-1.16 q < 0.784	-1.16 q < 0.597	-1.02 q < 0.951	-1.17 q < 1	-1.15 q < 0.955
MAF	v-maf musculoaponeurotic fibrosarcoma oncogene homolog (avian)	Ssc.15325.1.S1_at	-1.23 q < 0.0212	-1.08 q < 1	-1.22 q < 0.767	-1.3 q < 0.38	-1.25 q < 1	-1.3 q < 0.785
LEPRE1	leucine proline-enriched proteoglycan (leprecan) 1	Ssc.3670.1.S1_a_at	-1.24 q < 0.0212	-1.41 q < 0.409	-1.39 q < 0.392	-1.16 q < 0.665	-1.06 q < 1	-1.21 q < 1
ZKSCAN1	zinc finger with KRAB and SCAN domains 1	Ssc.22060.1.A1_at	1.12 q < 0.0213	1.01 q < 1	1.22 q < 0.251	1.08 q < 0.664	1.11 q < 1	1.19 q < 0.485
MPI	mannose phosphate isomerase	Ssc.8311.1.A1_at	1.11 q < 0.0213	1.1 q < 1	1.02 q < 0.973	1.19 q < 0.206	1.14 q < 1	1.1 q < 1
AKAP13	A kinase (PRKA) anchor protein 13	Ssc.17348.1.S1_at	-1.16 q < 0.0213	-1.07 q < 1	-1.23 q < 0.447	-1.05 q < 0.868	-1.22 q < 1	-1.23 q < 0.659
HAND2	heart and neural crest derivatives expressed 2	Ssc.31068.1.A1_at	-1.31 q < 0.0213	-1.72 q < 0.147	-1.28 q < 0.791	-1.44 q < 0.337	-1.02 q < 1	-1.19 q < 1
LSG1	large subunit GTPase 1 homolog (S. cerevisiae)	Ssc.5261.1.A1_at	-1.12 q < 0.0214	-1.15 q < 0.808	-1.29 q < 0.0704	-1.28 q < 0.0669	-1.01 q < 1	1.07 q < 1
SYS1	SYS1 Golgi-localized integral membrane protein homolog (S. cerevisiae)	Ssc.817.2.A1_at	1.14 q < 0.0214	1.04 q < 1	1.1 q < 0.868	1.25 q < 0.182	1.07 q < 1	1.27 q < 0.288
CLEC4M	C-type lectin domain family 4, member M	Ssc.29642.1.A1_at	-1.12 q < 0.0214	-1.19 q < 0.436	-1.14 q < 0.647	-1.11 q < 0.539	-1.17 q < 0.951	1.02 q < 1
RASIP1	Ras interacting protein 1	Ssc.26694.1.S1_at	-1.13 q < 0.0215	-1.16 q < 0.783	-1.21 q < 0.347	-1.04 q < 0.876	-1.1 q < 1	-1.14 q < 1
SCHIP1	schwannomin interacting protein 1	Ssc.6666.1.A1_at	-1.18 q < 0.0215	1.04 q < 1	-1.26 q < 0.497	-1.04 q < 0.9	-1.3 q < 0.744	-1.42 q < 0.127
C1QTNF6	C1q and tumor necrosis factor related protein 6	Ssc.27811.1.S1_at	-1.25 q < 0.0216	-1.61 q < 0.107	-1.34 q < 0.54	-1.28 q < 0.468	1 q < 1	-1.11 q < 1
ATXN10	ataxin 10	Ssc.12724.1.S1_at	-1.16 q < 0.0221	-1.14 q < 1	-1.22 q < 0.515	-1.18 q < 0.445	-1.09 q < 1	-1.15 q < 1
GMPR	guanosine monophosphate reductase	Ssc.3433.2.S1_at	-1.14 q < 0.0222	-1.04 q < 1	-1.26 q < 0.239	-1.19 q < 0.356	-1.16 q < 1	-1.06 q < 1
GMPR	guanosine monophosphate reductase	Ssc.3433.1.S1_at	-1.14 q < 0.0389	1.01 q < 1	-1.29 q < 0.225	-1.19 q < 0.405	-1.11 q < 1	-1.15 q < 1
SCCPDH	saccharopine dehydrogenase (putative)	Ssc.6810.1.A1_at	1.2 q < 0.0222	1.27 q < 0.732	1.03 q < 0.982	1.24 q < 0.439	1.28 q < 1	1.21 q < 1
C16orf73	chromosome 16 open reading frame 73	Ssc.11467.1.A1_at	-1.16 q < 0.0224	1.04 q < 1	-1.27 q < 0.31	-1.26 q < 0.244	-1.14 q < 1	-1.18 q < 0.937
NSDHL	NAD(P) dependent steroid dehydrogenase-like	Ssc.28560.1.A1_at	1.14 q < 0.0227	1.12 q < 1	-1.02 q < 0.981	1.24 q < 0.218	1.11 q < 1	1.28 q < 0.247
NSDHL	NAD(P) dependent steroid dehydrogenase-like	Ssc.3253.1.S1_at	1.16 q < 0.0352	1.38 q < 0.165	-1.1 q < 0.898	1.24 q < 0.349	1.18 q < 1	1.17 q < 1
TRAPPC3	trafficking protein particle complex 3	Ssc.1247.1.S1_at	-1.16 q < 0.0231	-1.07 q < 1	-1.35 q < 0.118	-1.14 q < 0.569	-1.08 q < 1	-1.16 q < 1
BRF2	BRF2, subunit of RNA polymerase III transcription initiation factor, BRF1-like	Ssc.21555.2.A1_at	-1.27 q < 0.0232	-1.38 q < 0.679	-1.27 q < 0.754	-1.1 q < 0.818	-1.57 q < 0.378	-1.09 q < 1
BRF2	zinc finger protein 36, C3H type-like 2	Ssc.21555.2.A1_at	-1.27 q < 0.0232	-1.38 q < 0.679	-1.27 q < 0.754	-1.1 q < 0.818	-1.57 q < 0.378	-1.09 q < 1
SERINC3	serine incorporator 3	Ssc.7222.1.S1_at	-1.15 q < 0.0233	-1.1 q < 1	-1.3 q < 0.172	-1.02 q < 0.957	-1.24 q < 0.777	-1.1 q < 1
PIBF1	progesterone immunomodulatory binding factor 1	Ssc.15394.2.S1_at	-1.1 q < 0.0235	-1.08 q < 1	-1.15 q < 0.452	-1.06 q < 0.736	-1.15 q < 1	-1.08 q < 1
SETX	senataxin	Ssc.26227.1.S1_at	-1.1 q < 0.0237	-1.09 q < 1	-1.13 q < 0.555	-1.09 q < 0.569	-1.13 q < 1	-1.06 q < 1
ALKBH1	alkB, alkylation repair homolog 1 (E. coli)	Ssc.14160.1.S1_at	-1.12 q < 0.0238	-1.09 q < 1	-1.21 q < 0.273	-1.03 q < 0.894	-1.17 q < 0.991	-1.09 q < 1
TNFAIP2	tumor necrosis factor, alpha-induced protein 2	Ssc.28739.1.S1_at	1.12 q < 0.0238	1.11 q < 1	1.16 q < 0.513	1.17 q < 0.3	1.06 q < 1	1.08 q < 1
YEATS2	YEATS domain containing 2	Ssc.6857.1.A1_at	-1.1 q < 0.0239	-1.01 q < 1	-1.23 q < 0.0923	1.05 q < 0.779	-1.21 q < 0.286	-1.12 q < 0.903
ELOVL2	elongation of very long chain fatty acids (FEN1/Elo2, SUR4/Elo3, yeast)-like 2	Ssc.30467.1.A1_at	1.48 q < 0.0242	1.43 q < 1	1.73 q < 0.492	1.96 q < 0.189	1.03 q < 1	1.43 q < 1
FBXO18	F-box protein, helicase, 18	Ssc.21961.1.A1_at	1.14 q < 0.0243	1.17 q < 0.795	-1 q < 0.997	1.24 q < 0.196	1.18 q < 1	1.11 q < 1
EGFL6	EGF-like-domain, multiple 6	Ssc.27626.1.S1_at	1.32 q < 0.0243	1.44 q < 0.732	1 q < 1	1.42 q < 0.406	1.21 q < 1	1.65 q < 0.317
Q8N812	-	Ssc.1197.1.A1_at	-1.12 q < 0.0243	-1.05 q < 1	-1.15 q < 0.606	-1.07 q < 0.745	-1.19 q < 0.97	-1.17 q < 0.732
CD97	CD97 molecule	Ssc.3348.1.S1_at	-1.11 q < 0.0244	-1.03 q < 1	-1.16 q < 0.448	-1.1 q < 0.575	-1.03 q < 1	-1.24 q < 0.124
GGA1	golgi-associated, gamma adaptin ear containing, ARF binding protein 1	Ssc.16891.1.S1_at	-1.14 q < 0.0244	-1.08 q < 1	-1.32 q < 0.127	-1.03 q < 0.926	-1.2 q < 1	-1.12 q < 1
STOM	stomatin	Ssc.5834.1.A1_at	-1.25 q < 0.0247	1.06 q < 1	-1.48 q < 0.243	-1.3 q < 0.435	-1.46 q < 0.583	-1.14 q < 1
PTPRS	protein tyrosine phosphatase, receptor type, S	Ssc.21137.1.A1_at	-1.16 q < 0.0248	-1.24 q < 0.535	-1.23 q < 0.468	-1.14 q < 0.563	-1.17 q < 1	-1.01 q < 1
RELL1	RELT-like 1	Ssc.13523.1.A1_at	-1.11 q < 0.0249	-1.02 q < 1	-1.26 q < 0.0796	-1.16 q < 0.317	-1.1 q < 1	-1.03 q < 1
EHD2	EH-domain containing 2	Ssc.5576.1.A1_at	-1.22 q < 0.025	-1.03 q < 1	-1.39 q < 0.306	-1.21 q < 0.539	-1.26 q < 1	-1.22 q < 1
RPS3	ribosomal protein S3	Ssc.4042.1.S1_at	-1.1 q < 0.0254	1.07 q < 1	-1.23 q < 0.108	-1.17 q < 0.234	-1.04 q < 1	-1.17 q < 0.494
KLF9	Kruppel-like factor 9	Ssc.16664.1.A1_at	1.16 q < 0.0258	-1.04 q < 1	1.16 q < 0.771	1.12 q < 0.65	1.23 q < 1	1.37 q < 0.116
ANKRD27	ankyrin repeat domain 27 (VPS9 domain)	Ssc.7196.1.A1_at	-1.18 q < 0.0258	1.04 q < 1	-1.28 q < 0.444	-1.15 q < 0.607	-1.52 q < 0.0509	-1.07 q < 1
ANGPTL2	angiopoietin-like 2	Ssc.29929.1.S1_at	-1.29 q < 0.0259	-1.84 q < 0.0559	-1.24 q < 0.843	-1.37 q < 0.418	1.08 q < 1	-1.25 q < 1

Gene Name	Gene Description	Probe Set ID	Breed MS-WC	Day 25 MS-WC	Day 45 MS-WC	Day 65 MS-WC	Day 85 MS-WC	Day 105 MS-WC
NEK7	NIMA (never in mitosis gene a)-related kinase 7	Ssc.21023.1.S1_at	-1.24 q < 0.0259	-1 q < 1	-1.32 q < 0.552	-1.16 q < 0.685	-1.35 q < 1	-1.4 q < 0.505
TMEM63A	transmembrane protein 63A	Ssc.5793.1.S1_at	-1.12 q < 0.026	-1.1 q < 1	-1.29 q < 0.0755	-1.15 q < 0.42	-1.01 q < 1	-1.07 q < 1
MAPK8IP3	mitogen-activated protein kinase 8 interacting protein 3	Ssc.18249.1.S1_at	-1.14 q < 0.026	-1.19 q < 0.682	-1.24 q < 0.314	-1.05 q < 0.841	-1.12 q < 1	-1.1 q < 1
BGN	biglycan	Ssc.5943.1.S1_at	-1.22 q < 0.026	-1.23 q < 0.984	-1.55 q < 0.0807	-1.13 q < 0.708	-1.12 q < 1	-1.11 q < 1
UBFD1	ubiquitin family domain containing 1	Ssc.27821.1.A1_at	1.12 q < 0.0261	1.08 q < 1	1.13 q < 0.703	1.14 q < 0.473	1.16 q < 1	1.1 q < 1
CSF1R	colony stimulating factor 1 receptor	Ssc.5826.1.A1_at	-1.34 q < 0.0261	-1.2 q < 1	-1.33 q < 0.77	-1.2 q < 0.72	-1.18 q < 1	-1.9 q < 0.09
MRPL11	mitochondrial ribosomal protein L11	Ssc.6370.1.S1_at	-1.12 q < 0.0269	1.16 q < 0.751	-1.28 q < 0.109	-1.14 q < 0.469	-1.14 q < 1	-1.25 q < 0.238
RPUSD1	RNA pseudouridylate synthase domain containing 1	Ssc.2915.1.S1_at	-1.1 q < 0.0269	-1.12 q < 0.803	-1.18 q < 0.249	-1 q < 0.994	-1.08 q < 1	-1.11 q < 1
TNRC6C	trinucleotide repeat containing 6C	Ssc.2114.1.S1_at	-1.13 q < 0.0271	-1.14 q < 0.927	-1.18 q < 0.52	-1.15 q < 0.444	-1.2 q < 0.875	1.02 q < 1
SUOX	sulfite oxidase	Ssc.30859.1.A1_at	1.17 q < 0.0272	1.05 q < 1	1.11 q < 0.884	1.4 q < 0.0644	1.19 q < 1	1.11 q < 1
CCDC80	coiled-coil domain containing 80	Ssc.3825.1.S1_at	-1.46 q < 0.0274	-1.16 q < 1	-1.95 q < 0.269	-1.62 q < 0.399	-1.05 q < 1	-1.73 q < 0.671
PEX7	peroxisomal biogenesis factor 7	Ssc.12804.1.S1_at	1.15 q < 0.0274	1.2 q < 0.727	-1.04 q < 0.967	1.25 q < 0.24	1.24 q < 0.831	1.11 q < 1
GRB2	growth factor receptor-bound protein 2	Ssc.26718.1.S1_at	-1.11 q < 0.0274	-1.21 q < 0.31	-1.13 q < 0.658	-1.09 q < 0.615	-1.08 q < 1	-1.06 q < 1
HSPA9	heat shock 70kDa protein 9 (mortalin)	Ssc.31057.1.A1_s_at	1.15 q < 0.0275	1.31 q < 0.225	-1.09 q < 0.899	1.22 q < 0.344	1.19 q < 1	1.17 q < 1
AGPAT3	1-acylglycerol-3-phosphate O-acyltransferase 3	Ssc.5622.1.A1_at	1.26 q < 0.0276	1.34 q < 0.792	1.48 q < 0.302	1.49 q < 0.194	1.02 q < 1	1.05 q < 1
CPXM2	carboxypeptidase X (M14 family), member 2	Ssc.26276.1.S1_at	-1.59 q < 0.0277	-1.59 q < 1	-1.31 q < 0.911	1.1 q < 0.917	-2.1 q < 0.779	-2.58 q < 0.164
MAGT1	magnesium transporter 1	Ssc.17326.1.A1_at	-1.24 q < 0.0278	-1.16 q < 1	-1.49 q < 0.216	-1.04 q < 0.925	-1.35 q < 1	-1.22 q < 1
ADAM18	ADAM metallopeptidase domain 18	Ssc.13121.1.A1_at	1.12 q < 0.0278	1.04 q < 1	1.13 q < 0.708	1.2 q < 0.233	1.15 q < 1	1.07 q < 1
CNIH	cornichon homolog (Drosophila)	Ssc.6495.1.S1_at	1.18 q < 0.0278	1.31 q < 0.432	-1.01 q < 0.993	1.27 q < 0.308	1.16 q < 1	1.19 q < 1
PARVG	parvin, gamma	Ssc.10710.1.A1_at	-1.16 q < 0.028	-1.09 q < 1	-1.05 q < 0.951	-1.17 q < 0.5	-1.32 q < 0.42	-1.18 q < 1
NKIRAS1	NFKB inhibitor interacting Ras-like 1	Ssc.12641.1.S1_at	-1.13 q < 0.0283	-1.16 q < 0.807	-1.26 q < 0.181	-1.06 q < 0.776	-1.06 q < 1	-1.11 q < 1
DUSP22	dual specificity phosphatase 22	Ssc.25180.1.S1_at	-1.13 q < 0.0285	-1 q < 1	-1.22 q < 0.317	-1.14 q < 0.489	-1.18 q < 1	-1.11 q < 1
MRPL28	mitochondrial ribosomal protein L28	Ssc.3916.1.A1_at	-1.12 q < 0.0287	-1.02 q < 1	-1.21 q < 0.289	-1.07 q < 0.736	-1.09 q < 1	-1.22 q < 0.325
LPIN1	lipin 1	Ssc.1942.1.S1_at	-1.14 q < 0.0288	-1.15 q < 0.938	-1.16 q < 0.662	-1.16 q < 0.477	-1.15 q < 1	-1.07 q < 1
SNX6	sorting nexin 6	Ssc.16529.1.S1_at	-1.18 q < 0.0294	1 q < 1	-1.18 q < 0.756	-1.11 q < 0.714	-1.22 q < 1	-1.42 q < 0.124
HOXD9	homeobox D9	Ssc.23551.1.S1_at	1.13 q < 0.0295	1.08 q < 1	1.08 q < 0.903	1.21 q < 0.249	1.13 q < 1	1.14 q < 1
DAD1	defender against cell death 1	Ssc.11184.1.S1_at	-1.19 q < 0.0297	-1.12 q < 1	-1.42 q < 0.147	1.02 q < 0.952	-1.13 q < 1	-1.36 q < 0.365
ASTN2	astrotactin 2	Ssc.30418.1.A1_at	-1.13 q < 0.0298	-1.31 q < 0.0818	-1.05 q < 0.937	-1.05 q < 0.81	-1.07 q < 1	-1.16 q < 0.893
MTMR9	myotubularin related protein 9	Ssc.14180.1.A1_at	-1.12 q < 0.0301	-1.2 q < 0.413	-1.13 q < 0.714	-1.07 q < 0.715	-1.12 q < 1	-1.06 q < 1
UST	uronyl-2-sulfotransferase	Ssc.18781.1.A1_at	-1.15 q < 0.0301	-1.06 q < 1	-1.08 q < 0.919	1.01 q < 0.983	-1.34 q < 0.215	-1.3 q < 0.234
UST	uronyl-2-sulfotransferase	Ssc.30640.1.S1_at	1.19 q < 0.0318	1.09 q < 1	1.15 q < 0.855	1.38 q < 0.156	1.22 q < 1	1.13 q < 1
TRRAP	transformation/transcription domain-associated protein	Ssc.4749.1.S1_at	1.17 q < 0.0302	1.03 q < 1	1.2 q < 0.662	1.39 q < 0.083	1.08 q < 1	1.17 q < 1
ABLIM3	actin binding LIM protein family, member 3	Ssc.5414.1.S1_at	-1.21 q < 0.0304	-1.22 q < 0.977	-1.44 q < 0.188	-1.2 q < 0.556	-1.15 q < 1	-1.06 q < 1
ABLIM3	actin binding LIM protein family, member 3	Ssc.27988.1.A1_at	1.1 q < 0.105	-1.04 q < 1	1.07 q < 0.91	1.5 q < 0.000304	1 q < 1	1.04 q < 1
SPOCK1	sparc/osteonectin, cwcv and kazal-like domains proteoglycan (testican) 1	Ssc.25458.1.S1_at	1.13 q < 0.0304	-1.01 q < 1	1.35 q < 0.0554	1.2 q < 0.329	1.13 q < 1	1.04 q < 1
COX8A	cytochrome c oxidase subunit VIIIA (ubiquitous)	Ssc.26496.1.A1_at	1.13 q < 0.0304	1.07 q < 1	1.11 q < 0.847	1.29 q < 0.0908	1.13 q < 1	1.07 q < 1
FMOD	fibromodulin	Ssc.11858.1.S1_at	-1.55 q < 0.0306	-1.47 q < 1	-1.65 q < 0.69	-1.64 q < 0.48	-1.2 q < 1	-1.9 q < 0.672
COQ4	coenzyme Q4 homolog (S. cerevisiae)	Ssc.4213.1.S1_at	1.13 q < 0.0306	1.08 q < 1	1 q < 1	1.21 q < 0.247	1.05 q < 1	1.32 q < 0.0745
SOX13	SRY (sex determining region Y)-box 13	Ssc.12263.1.A1_at	-1.13 q < 0.0308	-1.07 q < 1	-1.26 q < 0.195	-1.07 q < 0.747	-1.26 q < 0.42	-1.01 q < 1
CBLB	Cas-Br-M (murine) ecotropic retroviral transforming sequence b	Ssc.2567.2.A1_at	-1.16 q < 0.0308	-1.14 q < 1	-1.27 q < 0.375	-1.27 q < 0.256	-1.14 q < 1	1 q < 1
CBLB	methylmalonic aciduria (cobalamin deficiency) cblB type	Ssc.2567.2.A1_at	-1.16 q < 0.0308	-1.14 q < 1	-1.27 q < 0.375	-1.27 q < 0.256	-1.14 q < 1	1 q < 1
ECD	ecdysoneless homolog (Drosophila)	Ssc.13124.1.S1_at	1.13 q < 0.031	-1.01 q < 1	1.21 q < 0.398	1.25 q < 0.142	1.06 q < 1	1.13 q < 1
ECD	split hand/foot malformation (ectrodactyly) type 1	Ssc.13124.1.S1_at	1.13 q < 0.031	-1.01 q < 1	1.21 q < 0.398	1.25 q < 0.142	1.06 q < 1	1.13 q < 1
OBFC2B	oligonucleotide/oligosaccharide-binding fold containing 2B	Ssc.2422.1.S1_at	-1.14 q < 0.031	-1.16 q < 0.893	-1.25 q < 0.286	1.02 q < 0.958	-1.16 q < 1	-1.15 q < 1
FANCG	Fanconi anemia, complementation group G	Ssc.22197.1.S1_at	-1.15 q < 0.0311	-1.03 q < 1	-1.12 q < 0.86	-1.34 q < 0.085	-1.32 q < 0.348	1.02 q < 1
TCP11L1	t-complex 11 (mouse)-like 1	Ssc.7564.1.A1_at	-1.14 q < 0.0314	-1.19 q < 0.72	-1.25 q < 0.3	1.07 q < 0.754	-1.16 q < 1	-1.18 q < 0.838
TSPAN6	tetraspanin 6	Ssc.16583.1.S1_at	-1.12 q < 0.0315	-1.06 q < 1	-1.3 q < 0.0885	-1.14 q < 0.499	-1.09 q < 1	-1.06 q < 1

Gene Name	Gene Description	Probe Set ID	Breed MS-WC	Day 25 MS-WC	Day 45 MS-WC	Day 65 MS-WC	Day 85 MS-WC	Day 105 MS-WC
C2orf31	frizzled homolog 5 (Drosophila)	Ssc.13439.1.A1_at	1.31 q < 0.0318	1.59 q < 0.373	1.12 q < 0.94	1.42 q < 0.396	1.04 q < 1	1.45 q < 0.752
LAPTM5	lysosomal protein transmembrane 5	Ssc.22037.1.S1_at	-1.4 q < 0.0318	1.09 q < 1	-1.57 q < 0.554	-1.62 q < 0.329	-1.35 q < 1	-1.7 q < 0.559
PHF21A	PHD finger protein 21A	Ssc.29183.1.A1_at	1.1 q < 0.0323	1.03 q < 1	1.08 q < 0.852	1.2 q < 0.136	1.03 q < 1	1.17 q < 0.494
PITX2	paired-like homeodomain 2	Ssc.6994.1.S1_at	-1.28 q < 0.0323	-1.23 q < 1	-1.34 q < 0.647	-1.38 q < 0.393	-1.06 q < 1	-1.4 q < 0.759
RGN	regucalcin (senescence marker protein-30)	Ssc.13891.1.A1_at	-1.85 q < 0.0323	1.14 q < 1	-2.7 q < 0.375	-1.45 q < 0.731	-2.94 q < 0.604	-2.12 q < 0.931
ERC1	ELKS/RAB6-interacting/CAST family member 1	Ssc.3221.2.S1_at	-1.15 q < 0.0327	-1.04 q < 1	-1.3 q < 0.195	-1.11 q < 0.667	-1.15 q < 1	-1.15 q < 1
LACTB	lactamase, beta	Ssc.26736.1.A1_at	-1.13 q < 0.0328	1.01 q < 1	-1.18 q < 0.52	-1.16 q < 0.418	-1.16 q < 1	-1.15 q < 1
GPC4	glypican 4	Ssc.29046.1.S1_at	-1.3 q < 0.0329	-1.26 q < 1	-1.25 q < 0.843	-1.37 q < 0.446	-1.15 q < 1	-1.5 q < 0.608
QKI	quaking homolog, KH domain RNA binding (mouse)	Ssc.21958.1.S1_at	1.24 q < 0.0329	1.43 q < 0.418	1.21 q < 0.817	1.44 q < 0.213	1.06 q < 1	1.09 q < 1
QKI	quaking homolog, KH domain RNA binding (mouse)	Ssc.7659.1.A1_at	-1.14 q < 0.0432	-1.1 q < 1	-1.27 q < 0.253	-1.21 q < 0.32	-1.1 q < 1	-1.01 q < 1
Q8N9L7	-	Ssc.27396.1.A1_at	-1.12 q < 0.033	-1.08 q < 1	-1.18 q < 0.496	-1.03 q < 0.914	-1.29 q < 0.219	-1.06 q < 1
DLG2	discs, large homolog 2 (Drosophila)	Ssc.29215.1.A1_at	1.13 q < 0.0333	1.09 q < 1	1.14 q < 0.739	1.26 q < 0.156	1.08 q < 1	1.1 q < 1
DLG2	membrane protein, palmitoylated 2 (MAGUK p55 subfamily member 2)	Ssc.29215.1.A1_at	1.13 q < 0.0333	1.09 q < 1	1.14 q < 0.739	1.26 q < 0.156	1.08 q < 1	1.1 q < 1
SLC15A1	solute carrier family 15 (oligopeptide transporter), member 1	Ssc.17339.1.S1_at	-1.37 q < 0.0333	1.01 q < 1	1.17 q < 0.929	-1.82 q < 0.146	-2.07 q < 0.143	-1.53 q < 0.816
CRIM1	cysteine rich transmembrane BMP regulator 1 (chordin-like)	Ssc.4900.1.A1_at	1.12 q < 0.0334	1.03 q < 1	1.09 q < 0.878	1.2 q < 0.244	1.07 q < 1	1.21 q < 0.439
RPS6	ribosomal protein S6	Ssc.15665.1.S1_at	-1.08 q < 0.0337	1.02 q < 1	-1.15 q < 0.272	-1.1 q < 0.411	-1.03 q < 1	-1.15 q < 0.348
ZNF114	zinc finger protein 114	Ssc.25434.1.S1_at	-1.12 q < 0.0338	-1.18 q < 0.563	-1.17 q < 0.503	-1.01 q < 0.98	-1.09 q < 1	-1.14 q < 0.937
TNFRSF1B	tumor necrosis factor receptor superfamily, member 1B	Ssc.15242.1.S1_at	-1.14 q < 0.034	-1.08 q < 1	-1.18 q < 0.652	-1.08 q < 0.751	-1.09 q < 1	-1.31 q < 0.205
YIPF6	Yip1 domain family, member 6	Ssc.12989.1.A1_at	-1.12 q < 0.034	-1.32 q < 0.0785	-1.09 q < 0.885	-1.01 q < 0.966	-1.19 q < 1	-1.05 q < 1
FAM60A	family with sequence similarity 60, member A	Ssc.28499.1.S1_at	1.16 q < 0.0341	1.02 q < 1	1.27 q < 0.362	1.2 q < 0.417	-1.01 q < 1	1.35 q < 0.177
RPS16	ribosomal protein S16	Ssc.16651.1.S1_at	-1.1 q < 0.0341	1.01 q < 1	-1.23 q < 0.117	-1.14 q < 0.393	1.03 q < 1	-1.21 q < 0.238
HNRPLL	heterogeneous nuclear ribonucleoprotein L-like	Ssc.1672.1.A1_at	-1.12 q < 0.0346	1.03 q < 1	-1.2 q < 0.406	-1.15 q < 0.457	-1.17 q < 1	-1.14 q < 1
C10orf118	chromosome 10 open reading frame 118	Ssc.24426.1.S1_at	-1.11 q < 0.0346	-1.27 q < 0.0944	-1.12 q < 0.731	-1.06 q < 0.779	-1.1 q < 1	-1.02 q < 1
GPRC5C	G protein-coupled receptor, family C, group 5, member C	Ssc.4105.1.S1_at	1.19 q < 0.0351	1.02 q < 1	1.31 q < 0.421	1.29 q < 0.332	1.22 q < 1	1.14 q < 1
MTCH1	mitochondrial carrier homolog 1 (C. elegans)	Ssc.3750.1.S1_at	-1.11 q < 0.0352	1.02 q < 1	-1.19 q < 0.337	-1.07 q < 0.707	-1.1 q < 1	-1.23 q < 0.239
PCGF5	polycomb group ring finger 5	Ssc.11641.1.A1_at	-1.11 q < 0.0354	-1.23 q < 0.201	-1.13 q < 0.661	-1.03 q < 0.867	-1.11 q < 1	-1.05 q < 1
SRPK2	SRSF protein kinase 2	Ssc.17862.1.A1_at	-1.13 q < 0.0354	-1.19 q < 0.658	-1.3 q < 0.114	1.09 q < 0.694	-1.13 q < 1	-1.13 q < 1
HNMT	histamine N-methyltransferase	Ssc.28933.1.S1_at	1.11 q < 0.0357	-1.01 q < 1	1.25 q < 0.155	-1.03 q < 0.902	1.19 q < 0.821	1.2 q < 0.452
OSBPL9	oxysterol binding protein-like 9	Ssc.12121.1.S1_at	-1.26 q < 0.0357	-1.26 q < 1	-1.47 q < 0.34	-1.11 q < 0.807	-1.17 q < 1	-1.3 q < 1
TMEM87B	transmembrane protein 87B	Ssc.27891.1.S1_at	1.18 q < 0.0357	1.15 q < 1	1.09 q < 0.929	1.41 q < 0.11	1.16 q < 1	1.13 q < 1
SLC27A6	solute carrier family 27 (fatty acid transporter), member 6	Ssc.24721.1.A1_at	1.16 q < 0.0361	1.27 q < 0.487	1.22 q < 0.576	1.27 q < 0.264	1.06 q < 1	1 q < 1
TLK1	tousled-like kinase 1	Ssc.24888.1.S1_at	-1.12 q < 0.0363	-1.08 q < 1	-1.11 q < 0.814	-1.01 q < 0.955	-1.3 q < 0.133	-1.11 q < 1
ATP6V0D1	ATPase, H+ transporting, lysosomal 38kDa, V0 subunit d1	Ssc.12957.1.A1_at	-1.1 q < 0.0364	-1.03 q < 1	-1.16 q < 0.429	-1.19 q < 0.165	1.04 q < 1	-1.17 q < 0.491
CENTG2	ArfGAP with GTPase domain, ankyrin repeat and PH domain 1	Ssc.23897.1.S1_a_at	-1.11 q < 0.0365	-1.19 q < 0.479	-1.18 q < 0.467	-1.14 q < 0.435	-1.07 q < 1	-1 q < 1
WDR70	WD repeat domain 70	Ssc.31089.1.A1_at	1.18 q < 0.0366	1.42 q < 0.139	1.03 q < 0.979	1.24 q < 0.393	1.09 q < 1	1.13 q < 1
TCF21	transcription factor 21	Ssc.5396.1.S1_at	-1.32 q < 0.0368	-1.82 q < 0.152	-1.63 q < 0.301	-1.2 q < 0.718	-1.08 q < 1	-1.05 q < 1
CLEC3B	C-type lectin domain family 3, member B	Ssc.5704.1.S1_at	-1.15 q < 0.0368	1 q < 1	1.05 q < 0.956	-1.19 q < 0.439	-1.42 q < 0.0898	-1.27 q < 0.487
ZNF547	zinc finger protein 547	Ssc.8205.1.A1_at	-1.12 q < 0.0368	-1.06 q < 1	-1.17 q < 0.546	-1.14 q < 0.467	-1.16 q < 1	-1.07 q < 1
GLRB	glycine receptor, beta	Ssc.6613.1.A1_at	1.1 q < 0.037	1.01 q < 1	1.11 q < 0.7	-1.02 q < 0.938	1.16 q < 0.921	1.25 q < 0.0693
MYADM	myeloid-associated differentiation marker	Ssc.15508.1.S1_at	-1.26 q < 0.0371	1.1 q < 1	-1.34 q < 0.632	-1.45 q < 0.274	-1.19 q < 1	-1.53 q < 0.374
RPL18	ribosomal protein L18	Ssc.10553.1.A1_a_at	-1.11 q < 0.0371	1.03 q < 1	-1.22 q < 0.202	-1.16 q < 0.32	1.01 q < 1	-1.22 q < 0.252
SGK2	serum/glucocorticoid regulated kinase 2	Ssc.24957.1.S1_at	1.5 q < 0.0371	1.02 q < 1	1.57 q < 0.726	2.25 q < 0.127	1.32 q < 1	1.6 q < 1
SGK2	serum/glucocorticoid regulated kinase family, member 3	Ssc.24957.1.S1_at	1.5 q < 0.0371	1.02 q < 1	1.57 q < 0.726	2.25 q < 0.127	1.32 q < 1	1.6 q < 1
TMEM176B	transmembrane protein 176B	Ssc.11219.1.S1_at	-1.13 q < 0.0371	-1.1 q < 1	-1.11 q < 0.853	1.02 q < 0.939	-1.19 q < 1	-1.32 q < 0.128
CTGF	connective tissue growth factor	Ssc.8562.3.A1_at	-1.53 q < 0.0372	-1.16 q < 1	-1.37 q < 0.881	-2.09 q < 0.217	-1.82 q < 1	-1.41 q < 1
TSKU	tsukushi small leucine rich proteoglycan homolog (Xenopus laevis)	Ssc.21942.3.A1_a_at	-1.14 q < 0.0373	-1.29 q < 0.248	-1.14 q < 0.788	-1.1 q < 0.657	1.04 q < 1	-1.22 q < 0.62
COPG	coatomer protein complex, subunit gamma	Ssc.18388.1.A1_at	-1.28 q < 0.0374	-1.37 q < 0.829	-1.73 q < 0.108	-1.08 q < 0.867	-1.06 q < 1	-1.26 q < 1

Gene Name	Gene Description	Probe Set ID	Breed MS-WC	Day 25 MS-WC	Day 45 MS-WC	Day 65 MS-WC	Day 85 MS-WC	Day 105 MS-WC
SIL1	SIL1 homolog, endoplasmic reticulum chaperone (S. cerevisiae)	Ssc.21852.1.S1_at	1.11 q < 0.0374	1.09 q < 1	1.01 q < 0.989	1.18 q < 0.242	1.18 q < 0.84	1.08 q < 1
FKBP15	FK506 binding protein 15, 133kDa	Ssc.5052.1.S1_at	-1.16 q < 0.0376	-1.16 q < 1	-1.31 q < 0.256	-1.12 q < 0.644	-1.07 q < 1	-1.13 q < 1
MYO1D	myosin ID	Ssc.4520.1.S1_at	1.23 q < 0.0376	1.29 q < 0.869	1.15 q < 0.898	1.58 q < 0.0846	1.11 q < 1	1.1 q < 1
CASK	calcium/calmodulin-dependent serine protein kinase (MAGUK family)	Ssc.12901.2.A1_at	-1.16 q < 0.0376	-1.01 q < 1	-1.29 q < 0.363	-1.22 q < 0.401	-1.15 q < 1	-1.17 q < 1
NDUFC1	NADH dehydrogenase (ubiquinone) 1, subcomplex unknown, 1, 6kDa	Ssc.3869.1.A1_at	1.19 q < 0.0383	1.34 q < 0.471	1.09 q < 0.93	1.17 q < 0.612	1.24 q < 1	1.15 q < 1
CHD8	chromodomain helicase DNA binding protein 8	Ssc.29071.1.A1_at	-1.13 q < 0.0384	-1.16 q < 0.857	-1.13 q < 0.749	-1.06 q < 0.778	-1.12 q < 1	-1.15 q < 0.962
PAQR5	progestin and adipoQ receptor family member V	Ssc.18808.1.S1_at	1.18 q < 0.0384	-1.01 q < 1	1.35 q < 0.29	1.38 q < 0.145	1.11 q < 1	1.12 q < 1
NEK1	NIMA (never in mitosis gene a)-related kinase 1	Ssc.9260.1.A1_at	-1.15 q < 0.0385	-1.01 q < 1	-1.26 q < 0.337	-1.07 q < 0.776	-1.26 q < 0.749	-1.14 q < 1
NTRI	neurotrimin	Ssc.6392.1.S1_at	-1.11 q < 0.0386	-1.25 q < 0.194	1.01 q < 0.993	-1.11 q < 0.552	-1.1 q < 1	-1.13 q < 1
SRP72	signal recognition particle 72kDa	Ssc.12078.1.A1_at	-1.11 q < 0.0391	-1.08 q < 1	-1.24 q < 0.2	-1.02 q < 0.946	-1.04 q < 1	-1.21 q < 0.399
DDAH1	dimethylarginine dimethylaminohydrolase 1	Ssc.16839.1.S1_at	1.31 q < 0.0392	1.36 q < 0.933	1.48 q < 0.51	1.62 q < 0.197	1.02 q < 1	1.16 q < 1
HTATIP2	HIV-1 Tat interactive protein 2, 30kDa	Ssc.4271.1.S1_at	1.15 q < 0.0394	1.31 q < 0.265	1.03 q < 0.982	1.23 q < 0.332	1.17 q < 1	1.04 q < 1
FTH1	ferritin, heavy polypeptide 1	Ssc.12288.1.A1_a_at	-1.12 q < 0.0396	1.09 q < 1	-1.22 q < 0.269	-1.18 q < 0.324	-1.03 q < 1	-1.28 q < 0.131
GPR153	G protein-coupled receptor 153	Ssc.28878.1.S1_at	-1.14 q < 0.0396	-1.15 q < 0.968	-1.11 q < 0.849	-1.04 q < 0.88	-1.12 q < 1	-1.28 q < 0.306
IPO5	importin 5	Ssc.6727.1.S1_at	-1.16 q < 0.0396	1.03 q < 1	-1.27 q < 0.424	-1.21 q < 0.44	-1.08 q < 1	-1.32 q < 0.338
LOXL2	lysyl oxidase-like 2	Ssc.2598.1.S1_at	-1.39 q < 0.0396	-1.62 q < 0.673	-1.25 q < 0.898	-1.12 q < 0.863	-1.3 q < 1	-1.8 q < 0.419
MRPS26	mitochondrial ribosomal protein S26	Ssc.12554.1.A1_at	-1.11 q < 0.0396	1.03 q < 1	-1.21 q < 0.286	-1.12 q < 0.515	-1.05 q < 1	-1.22 q < 0.286
PPP2R2B	protein phosphatase 2, regulatory subunit B, beta	Ssc.21607.1.A1_at	-1.49 q < 0.0396	-1.11 q < 1	-1.34 q < 0.881	-1.92 q < 0.261	-1.18 q < 1	-2.17 q < 0.278
TNFAIP8L3	tumor necrosis factor, alpha-induced protein 8-like 3	Ssc.6503.1.A1_at	-1.13 q < 0.0397	1.01 q < 1	-1.34 q < 0.0652	-1.26 q < 0.173	1.08 q < 1	-1.2 q < 0.731
SMPD1	sphingomyelin phosphodiesterase 1, acid lysosomal	Ssc.2209.1.S1_at	-1.17 q < 0.04	-1.44 q < 0.0928	-1.23 q < 0.576	-1.25 q < 0.343	-1.03 q < 1	1.06 q < 1
TMEM65	transmembrane protein 65	Ssc.10752.1.A1_at	1.12 q < 0.0401	1.31 q < 0.077	1.03 q < 0.976	1.16 q < 0.405	-1.03 q < 1	1.16 q < 0.919
Q9NW24	-	Ssc.18306.1.A1_at	2.81 q < 0.0405	1.92 q < 1	1.83 q < 0.913	2.26 q < 0.65	5.48 q < 0.823	3.99 q < 0.856
Q9P145	-	Ssc.12395.1.A1_at	-1.11 q < 0.0405	-1.12 q < 1	-1.08 q < 0.878	-1.07 q < 0.721	-1.23 q < 0.467	-1.07 q < 1
O43303	-	Ssc.25147.1.A1_at	-1.1 q < 0.0409	1.02 q < 1	-1.12 q < 0.657	-1.07 q < 0.706	-1.21 q < 0.366	-1.12 q < 0.984
CNDP1	carnosine dipeptidase 1 (metallopeptidase M20 family)	Ssc.25136.2.A1_a_at	-1.18 q < 0.041	-1.03 q < 1	-1.25 q < 0.579	-1.18 q < 0.568	-1.44 q < 0.245	-1.06 q < 1
LMNA	lamin A/C	Ssc.5112.1.S1_at	-1.18 q < 0.0411	1.07 q < 1	-1.41 q < 0.135	1.05 q < 0.88	-1.21 q < 1	-1.47 q < 0.0858
ISG20L2	interferon stimulated exonuclease gene 20kDa-like 2	Ssc.10835.1.A1_at	-1.12 q < 0.0411	-1.12 q < 1	-1.26 q < 0.181	-1.06 q < 0.81	-1.1 q < 1	-1.08 q < 1
ADRM1	adhesion regulating molecule 1	Ssc.2655.1.A1_at	1.13 q < 0.0411	1.2 q < 0.647	1.12 q < 0.842	1.29 q < 0.108	1.03 q < 1	1.03 q < 1
PRCC	papillary renal cell carcinoma (translocation-associated)	Ssc.12110.1.S1_at	-1.1 q < 0.0412	-1.18 q < 0.457	-1.18 q < 0.371	-1.01 q < 0.948	-1.12 q < 1	-1.03 q < 1
SLC19A2	solute carrier family 19 (thiamine transporter), member 2	Ssc.22020.1.S1_at	1.15 q < 0.0412	1.22 q < 0.768	1.09 q < 0.91	1.23 q < 0.359	1.1 q < 1	1.14 q < 1
CCL4L	chemokine (C-C motif) ligand 4-like 1	Ssc.23797.1.S1_at	-1.19 q < 0.0413	-1.26 q < 0.778	-1.1 q < 0.918	-1.02 q < 0.951	-1.1 q < 1	-1.5 q < 0.0764
RTN4IP1	reticulon 4 interacting protein 1	Ssc.8754.1.A1_at	1.13 q < 0.0414	1.15 q < 0.932	1.12 q < 0.814	1.09 q < 0.667	1.09 q < 1	1.19 q < 0.742
CHRAC1	chromatin accessibility complex 1	Ssc.27571.1.S1_at	-1.14 q < 0.0417	-1.06 q < 1	-1.33 q < 0.142	-1.05 q < 0.846	-1.13 q < 1	-1.16 q < 1
ST8SIA4	ST8 alpha-N-acetyl-neuraminide alpha-2,8-sialyltransferase 4	Ssc.27896.1.S1_at	-1.12 q < 0.042	-1.04 q < 1	-1.13 q < 0.767	-1.11 q < 0.591	-1.09 q < 1	-1.25 q < 0.306
ST8SIA4	ST8 alpha-N-acetyl-neuraminide alpha-2,8-sialyltransferase 4	Ssc.11698.1.A1_at	1.04 q < 0.802	-1.34 q < 0.105	-1.06 q < 0.937	1.08 q < 0.745	1.14 q < 1	1.41 q < 0.0287
FAM134B	family with sequence similarity 134, member B	Ssc.5407.1.A1_at	-1.36 q < 0.042	-1.21 q < 1	-1.61 q < 0.448	-1.17 q < 0.782	-1.36 q < 1	-1.48 q < 0.92
SLC39A6	solute carrier family 39 (zinc transporter), member 6	Ssc.6670.1.A1_at	-1.16 q < 0.042	-1.24 q < 0.686	-1.21 q < 0.641	-1.01 q < 0.985	-1.15 q < 1	-1.22 q < 0.905
SMC5	structural maintenance of chromosomes 5	Ssc.6286.1.A1_at	1.15 q < 0.0425	-1.02 q < 1	-1.08 q < 0.923	1.35 q < 0.0941	1.24 q < 1	1.31 q < 0.276
AFF4	AF4/FMR2 family, member 4	Ssc.13568.1.A1_at	1.13 q < 0.0426	-1.01 q < 1	-1.06 q < 0.933	1.32 q < 0.082	1.23 q < 0.785	1.23 q < 0.515
UBASH3B	ubiquitin associated and SH3 domain containing B	Ssc.25139.2.S1_at	-1.16 q < 0.0428	1 q < 1	-1.37 q < 0.156	-1.15 q < 0.58	-1.08 q < 1	-1.24 q < 0.752
CDC42EP5	CDC42 effector protein (Rho GTPase binding) 5	Ssc.17268.1.A1_at	-1.14 q < 0.0429	-1.18 q < 0.858	-1.23 q < 0.444	-1.03 q < 0.914	-1.05 q < 1	-1.24 q < 0.593
SSR4	signal sequence receptor, delta (translocon-associated protein delta)	Ssc.2762.1.S1_at	-1.19 q < 0.043	-1.12 q < 1	-1.28 q < 0.547	-1.11 q < 0.745	-1.02 q < 1	-1.48 q < 0.128
DLG3	discs, large homolog 3 (Drosophila)	Ssc.4962.1.S1_at	-1.13 q < 0.0432	-1.06 q < 1	-1.27 q < 0.186	-1.1 q < 0.648	-1.04 q < 1	-1.17 q < 0.876
DLG3	membrane protein, palmitoylated 3 (MAGUK p55 subfamily member 3)	Ssc.4962.1.S1_at	-1.13 q < 0.0432	-1.06 q < 1	-1.27 q < 0.186	-1.1 q < 0.648	-1.04 q < 1	-1.17 q < 0.876

Gene Name	Gene Description	Probe Set ID	Breed MS-WC	Day 25 MS-WC	Day 45 MS-WC	Day 65 MS-WC	Day 85 MS-WC	Day 105 MS-WC
FHL1	four and a half LIM domains 1	Ssc.14463.1.S1_at	-1.22 q < 0.0432	1.05 q < 1	-1.24 q < 0.749	-1.2 q < 0.587	-1.18 q < 1	-1.6 q < 0.0901
FHL1	complement factor H	Ssc.14463.1.S1_at	-1.22 q < 0.0432	1.05 q < 1	-1.24 q < 0.749	-1.2 q < 0.587	-1.18 q < 1	-1.6 q < 0.0901
EPB41L4A	erythrocyte membrane protein band 4.1 like 4A	Ssc.30320.1.S1_at	1.2 q < 0.0433	-1 q < 1	1.25 q < 0.651	1.51 q < 0.0674	1.09 q < 1	1.18 q < 1
PLXDC2	plexin domain containing 2	Ssc.12603.1.A1_at	1.33 q < 0.0437	-1.52 q < 0.667	1.31 q < 0.819	1.83 q < 0.105	1.62 q < 0.758	1.62 q < 0.497
AES	amino-terminal enhancer of split	Ssc.1793.1.S1_a_at	-1.13 q < 0.0438	1.05 q < 1	-1.33 q < 0.0749	-1.03 q < 0.89	-1.2 q < 1	-1.16 q < 0.986
AES	amino-terminal enhancer of split	Ssc.1793.1.S1_at	-1.08 q < 0.479	1.19 q < 0.888	-1.44 q < 0.0422	-1.1 q < 0.724	-1.13 q < 1	1.02 q < 1
NUP133	nucleoporin 133kDa	Ssc.23133.1.A1_at	-1.12 q < 0.0438	-1.08 q < 1	-1.2 q < 0.45	-1.11 q < 0.609	-1.06 q < 1	-1.18 q < 0.786
SMARCA4	SWI/SNF related, matrix associated, actin dependent regulator of chromatin, subfamily a, member 4	Ssc.26993.1.S1_at	1.1 q < 0.0438	-1.03 q < 1	1.09 q < 0.829	1.17 q < 0.23	1.09 q < 1	1.17 q < 0.453
WDR82	WD repeat domain 82	Ssc.21588.1.S1_at	-1.08 q < 0.0438	-1.04 q < 1	-1.14 q < 0.338	-1.13 q < 0.315	-1.12 q < 1	1.02 q < 1
WDR82	WD repeat domain 82 pseudogene 1	Ssc.21588.1.S1_at	-1.08 q < 0.0438	-1.04 q < 1	-1.14 q < 0.338	-1.13 q < 0.315	-1.12 q < 1	1.02 q < 1
TRAF3IP2	TRAF3 interacting protein 2	Ssc.31132.1.A1_at	1.15 q < 0.0441	1.22 q < 0.754	1.17 q < 0.738	1.26 q < 0.28	1.02 q < 1	1.12 q < 1
TXNDC12	thioredoxin domain containing 12 (endoplasmic reticulum)	Ssc.5458.1.S1_at	1.14 q < 0.0444	1.12 q < 1	-1.09 q < 0.898	1.27 q < 0.2	1.25 q < 0.823	1.2 q < 0.857
TMEM41A	transmembrane protein 41A	Ssc.21284.1.S1_at	-1.14 q < 0.0446	-1.08 q < 1	-1.22 q < 0.498	-1.17 q < 0.489	-1.17 q < 1	-1.08 q < 1
C5orf13	chromosome 5 open reading frame 13	Ssc.20917.1.S1_at	-1.15 q < 0.0446	-1.25 q < 0.577	-1.18 q < 0.708	-1.07 q < 0.801	-1.26 q < 0.836	-1.02 q < 1
Q96S00	-	Ssc.30151.1.A1_at	1.11 q < 0.0446	-1.02 q < 1	1.19 q < 0.381	1.17 q < 0.321	1.11 q < 1	1.1 q < 1
NR5A2	nuclear receptor subfamily 5, group A, member 2	Ssc.29813.1.A1_at	1.13 q < 0.0446	1.19 q < 0.697	1.04 q < 0.958	1.15 q < 0.49	1.05 q < 1	1.23 q < 0.507
ADAM9	ADAM metallopeptidase domain 9	Ssc.16051.1.S1_at	-1.28 q < 0.0447	-1.35 q < 0.877	-1.41 q < 0.556	-1.11 q < 0.831	-1.02 q < 1	-1.58 q < 0.365
CNOT8	CCR4-NOT transcription complex, subunit 8	Ssc.5718.1.A1_at	1.14 q < 0.0447	1.21 q < 0.629	1.05 q < 0.953	1.14 q < 0.534	1.09 q < 1	1.2 q < 0.807
PABPC5	poly(A) binding protein, cytoplasmic 5	Ssc.6912.1.A1_at	1.1 q < 0.0448	1.05 q < 1	1.19 q < 0.283	1.04 q < 0.829	1.03 q < 1	1.2 q < 0.312
GEM	GTP binding protein overexpressed in skeletal muscle	Ssc.639.1.A1_at	-1.38 q < 0.0448	-1.27 q < 1	-1.24 q < 0.899	-1.08 q < 0.906	-1.71 q < 0.843	-1.72 q < 0.529
GEM	geminin, DNA replication inhibitor	Ssc.639.1.A1_at	-1.38 q < 0.0448	-1.27 q < 1	-1.24 q < 0.899	-1.08 q < 0.906	-1.71 q < 0.843	-1.72 q < 0.529
MINA	MYC induced nuclear antigen	Ssc.24089.1.S1_a_at	-1.16 q < 0.045	-1.09 q < 1	-1.33 q < 0.236	1.03 q < 0.922	-1.2 q < 1	-1.24 q < 0.768
RFFL	ring finger and FYVE-like domain containing 1	Ssc.28059.1.A1_at	1.16 q < 0.0451	-1.03 q < 1	1.17 q < 0.764	1.4 q < 0.0723	1.06 q < 1	1.25 q < 0.683
Q7Z322	-	Ssc.21860.3.A1_at	-1.21 q < 0.0453	-1.14 q < 1	-1.29 q < 0.609	-1.15 q < 0.695	-1.4 q < 0.723	-1.11 q < 1
PLTP	phospholipid transfer protein	Ssc.3517.1.S1_at	-1.3 q < 0.0456	-1.19 q < 1	-1.89 q < 0.0703	-1.26 q < 0.618	1.05 q < 1	-1.37 q < 1
KIAA1704	KIAA1704	Ssc.26671.1.A1_at	-1.1 q < 0.0457	-1.11 q < 0.964	-1.18 q < 0.383	-1.03 q < 0.859	-1.07 q < 1	-1.11 q < 1
TXNL4B	thioredoxin-like 4B	Ssc.27278.1.S1_at	-1.11 q < 0.0458	-1.09 q < 1	-1.25 q < 0.173	-1.06 q < 0.779	-1.08 q < 1	-1.1 q < 1
LARGE	like-glycosyltransferase	Ssc.4364.2.S1_at	1.13 q < 0.0466	1.22 q < 0.504	1.09 q < 0.883	1.23 q < 0.25	1.06 q < 1	1.05 q < 1
C17orf54	chromosome 17 open reading frame 54	Ssc.29537.1.A1_at	1.11 q < 0.0467	1.03 q < 1	1.2 q < 0.303	1.16 q < 0.354	1.05 q < 1	1.1 q < 1
NIN	ninein (GSK3B interacting protein)	Ssc.5092.1.S1_at	-1.16 q < 0.0469	-1.23 q < 0.773	-1.24 q < 0.552	-1.16 q < 0.578	-1.28 q < 0.901	1.06 q < 1
UROD	uroporphyrinogen decarboxylase	Ssc.6659.1.S1_at	1.18 q < 0.0469	1.4 q < 0.257	-1.07 q < 0.942	1.31 q < 0.288	1.3 q < 0.955	1.04 q < 1
C14orf132	chromosome 14 open reading frame 132	Ssc.8453.1.A1_at	-1.14 q < 0.0471	-1.2 q < 0.735	-1.09 q < 0.91	-1.29 q < 0.155	-1.06 q < 1	-1.07 q < 1
EBAG9	estrogen receptor binding site associated, antigen, 9	Ssc.1109.1.A1_at	1.17 q < 0.0471	1.07 q < 1	1.18 q < 0.777	1.25 q < 0.384	1.17 q < 1	1.2 q < 1
RALGDS	ral guanine nucleotide dissociation stimulator	Ssc.5618.2.S1_at	-1.12 q < 0.0471	-1.21 q < 0.551	-1.14 q < 0.738	-1.07 q < 0.758	-1.09 q < 1	-1.12 q < 1
COX6B1	cytochrome c oxidase subunit VIb polypeptide 1 (ubiquitous)	Ssc.5315.1.S1_at	1.17 q < 0.0471	1.35 q < 0.35	1.07 q < 0.94	1.04 q < 0.907	1.26 q < 1	1.17 q < 1
PLAT	plasminogen activator, tissue	Ssc.196.1.S1_at	-1.29 q < 0.0472	-1.52 q < 0.537	-1.43 q < 0.569	-1.44 q < 0.374	1.06 q < 1	-1.23 q < 1
SIGLECP3	sialic acid binding Ig-like lectin, pseudogene 3	Ssc.14336.1.A1_at	1.13 q < 0.0472	1.06 q < 1	1.07 q < 0.913	1.22 q < 0.259	1.07 q < 1	1.22 q < 0.56
B4GALT4	UDP-Gal:betaGlcNAc beta 1,4-galactosyltransferase, polypeptide 4	Ssc.17140.1.A1_at	1.18 q < 0.0475	1.44 q < 0.168	1.09 q < 0.933	-1 q < 0.991	1.28 q < 1	1.16 q < 1
ICA1	islet cell autoantigen 1, 69kDa	Ssc.15146.1.S1_at	1.14 q < 0.0475	1 q < 1	-1.08 q < 0.917	1.24 q < 0.267	1.3 q < 0.412	1.27 q < 0.377
Q8NAY9	-	Ssc.24083.1.A1_at	1.14 q < 0.0475	1.18 q < 0.881	1.13 q < 0.818	1.36 q < 0.0634	1.05 q < 1	1.01 q < 1
Q8NAY9	-	Ssc.20188.2.S1_at	-1.07 q < 0.595	-1.6 q < 0.00152	1.09 q < 0.91	1 q < 0.993	-1.02 q < 1	1.08 q < 1
CYP7B1	cytochrome P450, family 7, subfamily B, polypeptide 1	Ssc.24330.1.S1_at	-1.21 q < 0.0476	-1.44 q < 0.343	-1.17 q < 0.872	-1.3 q < 0.406	-1.09 q < 1	-1.11 q < 1
ISCA2	iron-sulfur cluster assembly 2 homolog (S. cerevisiae)	Ssc.28335.1.S1_at	1.13 q < 0.0476	1.06 q < 1	-1.03 q < 0.973	1.19 q < 0.352	1.36 q < 0.0873	1.09 q < 1
WFDC8	WAP four-disulfide core domain 8	Ssc.8061.1.A1_at	1.1 q < 0.0476	-1.05 q < 1	1.19 q < 0.295	1.24 q < 0.0818	1.04 q < 1	1.1 q < 1
CNP	2',3'-cyclic nucleotide 3' phosphodiesterase	Ssc.3522.2.S1_at	1.09 q < 0.0476	1.25 q < 0.0541	1.03 q < 0.958	1.1 q < 0.529	1.1 q < 1	-1.01 q < 1
CNP	natriuretic peptide precursor C	Ssc.3522.2.S1_at	1.09 q < 0.0476	1.25 q < 0.0541	1.03 q < 0.958	1.1 q < 0.529	1.1 q < 1	-1.01 q < 1

Gene Name	Gene Description	Probe Set ID	Breed MS-WC	Day 25 MS-WC	Day 45 MS-WC	Day 65 MS-WC	Day 85 MS-WC	Day 105 MS-WC
SPG21	spastic paraplegia 21 (autosomal recessive, Mast syndrome)	Ssc.14218.1.S1_at	1.14 q < 0.0477	1.07 q < 1	1.03 q < 0.981	1.2 q < 0.38	1.24 q < 0.821	1.16 q < 1
TSPAN15	tetraspanin 15	Ssc.19250.1.S1_at	1.14 q < 0.0487	1.19 q < 0.803	1.12 q < 0.854	1.23 q < 0.305	1.06 q < 1	1.11 q < 1
PPTC7	PTC7 protein phosphatase homolog (S. cerevisiae)	Ssc.24027.1.A1_at	1.14 q < 0.0487	-1.09 q < 1	1.14 q < 0.806	1.08 q < 0.743	1.27 q < 0.636	1.34 q < 0.137
STX4	syntaxin 4	Ssc.19737.1.S1_at	1.11 q < 0.0487	1.04 q < 1	1.01 q < 0.993	1.21 q < 0.199	1.27 q < 0.215	1.04 q < 1
GTPBP1	GTP binding protein 1	Ssc.24003.1.S1_at	1.11 q < 0.0493	1.13 q < 0.957	-1.05 q < 0.935	1.29 q < 0.0505	1.09 q < 1	1.12 q < 1
ROBO1	roundabout, axon guidance receptor, homolog 1 (Drosophila)	Ssc.12138.1.A1_at	-1.25 q < 0.0493	-1.74 q < 0.0818	-1.16 q < 0.908	-1.26 q < 0.548	-1.01 q < 1	-1.2 q < 1
YRDC	yrdC domain containing (E. coli)	Ssc.4998.1.A1_at	-1.11 q < 0.0493	-1.13 q < 0.967	-1.11 q < 0.78	-1.14 q < 0.431	-1.1 q < 1	-1.06 q < 1
KIF5C	kinesin family member 5C	Ssc.28310.1.S1_at	-1.21 q < 0.0496	1.07 q < 1	-1.44 q < 0.271	-1.26 q < 0.476	-1.4 q < 0.747	-1.11 q < 1
FBLN2	fibulin 2	Ssc.4479.1.S1_at	-1.21 q < 0.0497	-1.05 q < 1	-1.31 q < 0.552	-1.29 q < 0.395	-1.08 q < 1	-1.34 q < 0.672
O00172	-	Ssc.4658.1.A1_at	-1.1 q < 0.0497	-1.13 q < 0.886	-1.17 q < 0.402	1.1 q < 0.576	-1.29 q < 0.0601	-1.04 q < 1
QSOX2	quiescin Q6 sulfhydryl oxidase 2	Ssc.1614.1.A1_at	-1.12 q < 0.0498	-1.05 q < 1	-1.15 q < 0.658	-1.14 q < 0.488	-1.14 q < 1	-1.11 q < 1
SLC37A1	solute carrier family 37 (glycerol-3-phosphate transporter), member 1	Ssc.24911.1.S1_at	1.18 q < 0.05	-1.04 q < 1	1.11 q < 0.903	1.46 q < 0.071	1.27 q < 1	1.14 q < 1
MYH14	myosin, heavy chain 14, non-muscle	Ssc.12157.1.A1_at	1.21 q < 0.0506	-1.13 q < 1	1.07 q < 0.956	1.79 q < 0.00581	1.1 q < 1	1.37 q < 0.536
MYH14	myosin, heavy chain 7B, cardiac muscle, beta	Ssc.12157.1.A1_at	1.21 q < 0.0506	-1.13 q < 1	1.07 q < 0.956	1.79 q < 0.00581	1.1 q < 1	1.37 q < 0.536
TGFB2	transforming growth factor, beta 2	Ssc.9975.1.A1_at	1.13 q < 0.0509	1.12 q < 1	-1.01 q < 0.996	1.49 q < 0.00188	-1.19 q < 1	1.3 q < 0.178
FLNA	filamin A, alpha	Ssc.21758.1.S1_at	-1.12 q < 0.0517	1.1 q < 1	-1.36 q < 0.0367	-1.14 q < 0.493	-1.09 q < 1	-1.15 q < 1
NUFIP2	nuclear fragile X mental retardation protein interacting protein 2	Ssc.7387.1.A1_at	-1.13 q < 0.0521	-1.21 q < 0.644	-1.4 q < 0.0344	-1.05 q < 0.86	-1.05 q < 1	-1 q < 1
PLCZ1	phospholipase C, zeta 1	Ssc.17349.1.S1_at	1.1 q < 0.0527	-1.07 q < 1	1.08 q < 0.863	1.5 q < 0.0000401	-1.01 q < 1	1.08 q < 1
TSPAN12	tetraspanin 12	Ssc.6783.1.S1_at	1.25 q < 0.0533	1.35 q < 0.819	1.03 q < 0.989	1.83 q < 0.0239	1.07 q < 1	1.11 q < 1
HMBS	hydroxymethylbilane synthase	Ssc.11878.1.S1_at	1.18 q < 0.0534	1.61 q < 0.0185	1.02 q < 0.988	1.14 q < 0.669	1.22 q < 1	-1.01 q < 1
DENND1A	DENN/MADD domain containing 1A	Ssc.1929.1.S1_at	1.14 q < 0.0535	1.06 q < 1	1.12 q < 0.853	1.04 q < 0.874	1.09 q < 1	1.4 q < 0.0491
COG7	component of oligomeric golgi complex 7	Ssc.19030.1.S1_at	1.13 q < 0.0536	1.16 q < 0.927	-1.07 q < 0.923	1.42 q < 0.0165	1.16 q < 1	1.04 q < 1
RBL2	retinoblastoma-like 2 (p130)	Ssc.10727.1.A1_at	1.12 q < 0.0536	1.11 q < 1	1.01 q < 0.996	1.35 q < 0.0415	1.04 q < 1	1.15 q < 1
TPH2	tryptophan hydroxylase 2	Ssc.28909.3.A1_at	1.17 q < 0.0536	-1.17 q < 1	-1.01 q < 0.997	2.06 q < 0.0000068	1.02 q < 1	1.25 q < 0.855
TPH2	tryptophan 2,3-dioxygenase	Ssc.28909.3.A1_at	1.17 q < 0.0536	-1.17 q < 1	-1.01 q < 0.997	2.06 q < 0.0000068	1.02 q < 1	1.25 q < 0.855
SLC25A20	solute carrier family 25 (carnitine/acylcarnitine translocase), member 20	Ssc.19212.1.S1_at	1.16 q < 0.0553	1.27 q < 0.621	-1.11 q < 0.896	1.53 q < 0.0204	1.14 q < 1	1.07 q < 1
PCDH9	protocadherin 9	Ssc.19564.1.S1_at	-1.21 q < 0.0554	-1.7 q < 0.0431	-1.6 q < 0.0874	1.24 q < 0.524	-1.38 q < 0.921	1.14 q < 1
RPA1	replication protein A1, 70kDa	Ssc.4633.1.A1_at	-1.15 q < 0.0563	-1.12 q < 1	-1.44 q < 0.0387	-1.08 q < 0.782	-1.13 q < 1	-1 q < 1
RPA1	polymerase (RNA) I polypeptide A, 194kDa	Ssc.4633.1.A1_at	-1.15 q < 0.0563	-1.12 q < 1	-1.44 q < 0.0387	-1.08 q < 0.782	-1.13 q < 1	-1 q < 1
CYBB	cytochrome b-245, beta polypeptide	Ssc.151.1.S1_at	-1.17 q < 0.0567	-1.05 q < 1	-1.08 q < 0.938	1.09 q < 0.766	-1.02 q < 1	-2.04 q < 0.00000379
FCER1G	Fc fragment of IgE, high affinity I, receptor for; gamma polypeptide	Ssc.508.1.S1_at	-1.16 q < 0.0567	1.04 q < 1	-1.11 q < 0.9	1.01 q < 0.976	-1.1 q < 1	-1.84 q < 0.000141
BUB1B	budding uninhibited by benzimidazoles 1 homolog beta (yeast)	Ssc.7190.1.S1_at	1.13 q < 0.0571	1.59 q < 0.000441	1.05 q < 0.945	1.02 q < 0.941	1.09 q < 1	-1.02 q < 1
PTPRA	protein tyrosine phosphatase, receptor type, A	Ssc.6553.1.A1_at	1.11 q < 0.0573	-1.07 q < 1	-1.04 q < 0.954	1.18 q < 0.331	1.55 q < 0.000232	1.05 q < 1
PTPRA	protein tyrosine phosphatase, receptor type, A	Ssc.6553.2.S1_at	1.15 q < 0.06	1.01 q < 1	-1.04 q < 0.961	1.28 q < 0.234	1.52 q < 0.0169	1.05 q < 1
EAF2	ELL associated factor 2	Ssc.10498.1.A1_at	1.14 q < 0.0586	1.08 q < 1	1.02 q < 0.989	1.45 q < 0.0161	1.21 q < 1	-1.01 q < 1
EAF2	DNA methyltransferase 1 associated protein 1	Ssc.10498.1.A1_at	1.14 q < 0.0586	1.08 q < 1	1.02 q < 0.989	1.45 q < 0.0161	1.21 q < 1	-1.01 q < 1
OCA2	oculocutaneous albinism II	Ssc.15775.1.S1_at	1.13 q < 0.0598	1.46 q < 0.0149	1.02 q < 0.988	1.22 q < 0.317	-1.02 q < 1	1.04 q < 1
ARRDC4	arrestin domain containing 4	Ssc.17005.1.A1_at	-1.14 q < 0.0599	-1.63 q < 0.000952	-1.02 q < 0.989	-1.11 q < 0.678	1.13 q < 1	-1.2 q < 0.926
EDF1	endothelial differentiation-related factor 1	Ssc.4961.1.S1_at	-1.12 q < 0.0599	-1.02 q < 1	-1.45 q < 0.00489	-1.07 q < 0.755	1.01 q < 1	-1.12 q < 1
IGF1	insulin-like growth factor 1 (somatomedin C)	Ssc.12578.1.A1_at	-1.17 q < 0.061	-1.13 q < 1	-1.08 q < 0.937	-1 q < 0.993	-1.02 q < 1	-1.77 q < 0.0012
TYROBP	TYRO protein tyrosine kinase binding protein	Ssc.507.1.A1_at	-1.19 q < 0.064	1 q < 1	-1.13 q < 0.899	-1.15 q < 0.669	-1.05 q < 1	-1.74 q < 0.00852
SCD	stearoyl-CoA desaturase (delta-9-desaturase)	Ssc.16159.1.S1_at	1.31 q < 0.0652	2.11 q < 0.0451	-1.1 q < 0.958	1.57 q < 0.289	1.18 q < 1	1.07 q < 1
SCD	solute carrier family 22 (organic cation/carnitine transporter), member 5	Ssc.16159.1.S1_at	1.31 q < 0.0652	2.11 q < 0.0451	-1.1 q < 0.958	1.57 q < 0.289	1.18 q < 1	1.07 q < 1

Gene Name	Gene Description	Probe Set ID	Breed MS-WC	Day 25 MS-WC	Day 45 MS-WC	Day 65 MS-WC	Day 85 MS-WC	Day 105 MS-WC
CDS2	CDP-diacylglycerol synthase (phosphatidate cytidylyltransferase) 2	Ssc.26359.1.S1_a_at	1.11 q < 0.0661	1.27 q < 0.173	-1.1 q < 0.845	1.31 q < 0.0456	-1.06 q < 1	1.18 q < 0.706
IGKC	immunoglobulin kappa constant	Ssc.15778.1.S1_s_at	-1.14 q < 0.0663	-1.06 q < 1	1.04 q < 0.972	-1.05 q < 0.857	1.01 q < 1	-1.83 q < 0.0000167
SCARA5	scavenger receptor class A, member 5 (putative)	Ssc.6512.1.S1_at	-1.36 q < 0.0668	-3.04 q < 0.00137	-1.26 q < 0.885	-1.26 q < 0.689	1.12 q < 1	-1.05 q < 1
SCD5	stearoyl-CoA desaturase 5	Ssc.11708.1.A1_at	1.1 q < 0.0676	-1.03 q < 1	1.06 q < 0.913	1.3 q < 0.0266	1.05 q < 1	1.14 q < 0.937
LLGL2	lethal giant larvae homolog 2 (Drosophila)	Ssc.22143.1.S1_at	1.16 q < 0.0685	1.04 q < 1	1.04 q < 0.971	1.48 q < 0.0493	1.21 q < 1	1.11 q < 1
ADAMTS12	ADAM metallopeptidase with thrombospondin type 1 motif, 12	Ssc.18282.1.S1_at	-1.19 q < 0.0686	-1.73 q < 0.0131	-1.14 q < 0.898	1.11 q < 0.763	-1.08 q < 1	-1.26 q < 0.983
RERE	arginine-glutamic acid dipeptide (RE) repeats	Ssc.15512.1.S1_at	1.14 q < 0.0686	1.06 q < 1	-1.06 q < 0.948	1.47 q < 0.0212	1.09 q < 1	1.21 q < 0.855
MORF4L1	mortality factor 4 like 1	Ssc.1195.1.S1_at	-1.41 q < 0.0686	-2.63 q < 0.0439	-1.21 q < 0.93	1.06 q < 0.947	-1.17 q < 1	-1.59 q < 0.949
COX4NB	COX4 neighbor	Ssc.17265.1.S1_at	-1.13 q < 0.0691	1.03 q < 1	-1.42 q < 0.031	-1.13 q < 0.592	-1.04 q < 1	-1.14 q < 1
LPL	lipoprotein lipase	Ssc.1147.2.S1_at	1.21 q < 0.0699	1.04 q < 1	1.71 q < 0.0321	1.01 q < 0.991	1.2 q < 1	1.2 q < 1
SLC2A14	solute carrier family 2 (facilitated glucose transporter), member 14	Ssc.1674.1.A1_at	1.25 q < 0.0701	2.33 q < 0.000664	-1.09 q < 0.953	1.46 q < 0.278	-1.02 q < 1	-1.02 q < 1
KIAA1754	inositol 1,4,5-triphosphate receptor interacting protein	Ssc.18178.2.S1_at	1.1 q < 0.0701	1.17 q < 0.693	-1.04 q < 0.951	1.49 q < 0.000159	-1.07 q < 1	1.04 q < 1
PRLR	prolactin receptor	Ssc.24638.1.S1_at	-1.23 q < 0.0704	-1.04 q < 1	-1.1 q < 0.943	-1.04 q < 0.931	-1.83 q < 0.0488	-1.31 q < 1
SLC39A11	solute carrier family 39 (metal ion transporter), member 11	Ssc.24311.1.S1_at	-1.14 q < 0.0706	-1.03 q < 1	-1.76 q < 0.000054	-1.07 q < 0.814	-1.02 q < 1	1.03 q < 1
SNX24	sorting nexin 24	Ssc.8604.1.A1_at	-1.12 q < 0.0706	-1.25 q < 0.368	-1.49 q < 0.00306	1.06 q < 0.801	1.04 q < 1	-1.05 q < 1
PPM1H	protein phosphatase, Mg2+/Mn2+ dependent, 1H	Ssc.18826.1.A1_at	1.1 q < 0.0708	1.02 q < 1	1.08 q < 0.878	1.14 q < 0.43	-1.02 q < 1	1.32 q < 0.0334
LRRC4C	leucine rich repeat containing 4C	Ssc.18072.1.A1_at	-1.12 q < 0.0708	-1.47 q < 0.00481	-1.06 q < 0.933	-1.04 q < 0.871	-1.06 q < 1	-1.02 q < 1
LRRC4C	leucine rich repeat containing 4C	Ssc.25264.2.S1_a_at	1.13 q < 0.0857	-1.02 q < 1	1.12 q < 0.869	-1.04 q < 0.882	1.1 q < 1	1.64 q < 0.000877
CPT1A	carnitine palmitoyltransferase 1A (liver)	Ssc.8974.1.S1_at	1.2 q < 0.0713	1.08 q < 1	1.14 q < 0.903	1.67 q < 0.0321	1.17 q < 1	1.05 q < 1
PDAP1	PDGFA associated protein 1	Ssc.25607.1.S1_at	-1.11 q < 0.0714	-1 q < 1	-1.41 q < 0.00715	1.01 q < 0.979	-1.13 q < 1	-1.07 q < 1
FAM65B	family with sequence similarity 65, member B	Ssc.15335.1.S1_at	1.15 q < 0.0731	1.64 q < 0.00197	1.15 q < 0.804	1.05 q < 0.874	1.12 q < 1	-1.13 q < 1
BRCA1	breast cancer 1, early onset	Ssc.29471.1.A1_at	1.09 q < 0.0741	1.11 q < 0.994	1.04 q < 0.946	1.29 q < 0.0321	1.02 q < 1	1.04 q < 1
DDX46	DEAD (Asp-Glu-Ala-Asp) box polypeptide 46	Ssc.8127.1.A1_at	1.14 q < 0.0755	1.1 q < 1	1 q < 0.997	1.42 q < 0.0356	1.08 q < 1	1.12 q < 1
TNNI1	troponin I type 1 (skeletal, slow)	Ssc.2609.1.S1_at	1.11 q < 0.0764	1.45 q < 0.0018	1 q < 1	1.12 q < 0.557	-1.01 q < 1	1.04 q < 1
ZFP106	zinc finger protein 106 homolog (mouse)	Ssc.13616.2.S1_at	-1.12 q < 0.0768	-1.47 q < 0.00542	-1.13 q < 0.806	-1.06 q < 0.817	1 q < 1	-1 q < 1
STAT1	signal transducer and activator of transcription 1, 91kDa	Ssc.6025.2.A1_at	-1.21 q < 0.0775	1.08 q < 1	-1.38 q < 0.459	-1.03 q < 0.948	-1.14 q < 1	-1.7 q < 0.0445
NFIA	nuclear factor I/A	Ssc.30335.1.A1_at	1.1 q < 0.0778	-1 q < 1	1.11 q < 0.793	1.33 q < 0.0123	1.03 q < 1	1.05 q < 1
C17orf85	chromosome 17 open reading frame 85	Ssc.16988.1.S1_at	-1.09 q < 0.0823	1.01 q < 1	-1.22 q < 0.166	-1 q < 0.988	-1.32 q < 0.0193	1.03 q < 1
JMJD1A	lysine (K)-specific demethylase 3A	Ssc.30856.1.A1_s_at	1.1 q < 0.083	1.24 q < 0.252	-1.08 q < 0.893	1.36 q < 0.0124	-1.07 q < 1	1.12 q < 1
FAM46A	family with sequence similarity 46, member A	Ssc.10756.1.A1_at	-1.11 q < 0.0831	-1.38 q < 0.0285	1.04 q < 0.961	-1.07 q < 0.752	-1.15 q < 1	-1.05 q < 1
FAM46A	family with sequence similarity 46, member A	Ssc.15332.1.S1_at	-1.17 q < 0.279	-1.98 q < 0.00965	1.05 q < 0.975	-1.04 q < 0.938	-1.07 q < 1	-1.05 q < 1
DHCR7	7-dehydrocholesterol reductase	Ssc.5455.1.S1_at	-1.23 q < 0.0838	1.25 q < 1	-1.39 q < 0.516	-1.87 q < 0.0153	-1.1 q < 1	-1.21 q < 1
HM13	histocompatibility (minor) 13	Ssc.4188.1.S1_at	1.14 q < 0.0838	-1.16 q < 1	1.02 q < 0.988	1.77 q < 0.000104	1.14 q < 1	1.09 q < 1
ODZ1	odz, odd Oz/ten-m homolog 1(Drosophila)	Ssc.25498.1.S1_at	1.09 q < 0.0843	1.04 q < 1	1.05 q < 0.925	1.29 q < 0.015	1.06 q < 1	1.01 q < 1
C1orf26	chromosome 1 open reading frame 26	Ssc.8707.1.A1_at	1.12 q < 0.0846	1.04 q < 1	-1.01 q < 0.996	1.59 q < 0.00017	-1.02 q < 1	1.07 q < 1
STXBP3	syntaxin binding protein 3	Ssc.30767.1.A1_at	1.15 q < 0.0855	1.04 q < 1	-1.09 q < 0.917	1.55 q < 0.0126	1.13 q < 1	1.2 q < 1
LRP11	low density lipoprotein receptor-related protein 11	Ssc.14282.1.A1_at	-1.1 q < 0.0867	-1.11 q < 1	-1.36 q < 0.0174	1.03 q < 0.876	-1.03 q < 1	-1.08 q < 1
C6orf204	chromosome 6 open reading frame 204	Ssc.16987.1.A1_at	1.2 q < 0.0869	1.08 q < 1	-1.06 q < 0.969	1.07 q < 0.863	2.23 q < 0.000111	1 q < 1
HUNK	hormonally up-regulated Neu-associated kinase	Ssc.30233.1.A1_at	1.18 q < 0.0872	-1 q < 1	1.26 q < 0.662	1.05 q < 0.889	1.05 q < 1	1.66 q < 0.0252
HUNK	hormonally up-regulated Neu-associated kinase	Ssc.27274.1.S1_at	-1 q < 0.996	-1.07 q < 1	-1.49 q < 0.0461	1.44 q < 0.063	1.1 q < 1	-1.01 q < 1
AHNAK	AHNAK nucleoprotein	Ssc.27526.1.S1_at	-1.2 q < 0.0884	-1.11 q < 1	-1.44 q < 0.317	-1.73 q < 0.0219	1.34 q < 1	-1.21 q < 1
AHNAK	AHNAK nucleoprotein	Ssc.16645.1.S1_at	-1.13 q < 0.289	1.03 q < 1	-1.48 q < 0.127	-1.56 q < 0.0425	1.4 q < 0.541	-1.15 q < 1
C21orf33	chromosome 21 open reading frame 33	Ssc.21850.1.S1_at	-1.11 q < 0.0947	-1.04 q < 1	-1.37 q < 0.0202	-1 q < 0.988	-1.04 q < 1	-1.11 q < 1
STARD3	StAR-related lipid transfer (START) domain containing 3	Ssc.4484.1.S1_at	-1.1 q < 0.096	-1.03 q < 1	-1.35 q < 0.0311	1.07 q < 0.739	-1.12 q < 1	-1.13 q < 1
ALS2CR4	amyotrophic lateral sclerosis 2 (juvenile) chromosome region, candidate 4	Ssc.9901.1.A1_at	1.17 q < 0.096	1.14 q < 1	-1.31 q < 0.494	2.13 q < 0.0000246	1.02 q < 1	1.18 q < 1
DHRS12	dehydrogenase/reductase (SDR family) member 12	Ssc.21893.1.S1_at	-1.11 q < 0.0981	-1.14 q < 0.984	-1.45 q < 0.00515	1.01 q < 0.977	-1.02 q < 1	-1.01 q < 1

Gene Name	Gene Description	Probe Set ID	Breed MS-WC	Day 25 MS-WC	Day 45 MS-WC	Day 65 MS-WC	Day 85 MS-WC	Day 105 MS-WC
TMEM119	transmembrane protein 119	Ssc.1526.1.S1_at	-1.15 q < 0.0994	-1.57 q < 0.0216	-1.12 q < 0.894	-1.16 q < 0.601	1.13 q < 1	-1.13 q < 1
C6orf115	chromosome 6 open reading frame 115	Ssc.20225.1.A1_at	-1.2 q < 0.0995	1.03 q < 1	-1.21 q < 0.83	-1.12 q < 0.781	-1.08 q < 1	-1.77 q < 0.0274
CD81	CD81 molecule	Ssc.1641.1.S1_at	-1.17 q < 0.1	-1.12 q < 1	-1.58 q < 0.0469	-1.04 q < 0.914	-1.13 q < 1	-1.06 q < 1
TMEM47	transmembrane protein 47	Ssc.8464.1.A1_at	-1.15 q < 0.1	-1.72 q < 0.00146	-1.07 q < 0.94	-1.02 q < 0.964	-1.12 q < 1	1.05 q < 1
SLC36A2	solute carrier family 36 (proton/amino acid symporter), member 2	Ssc.4529.1.S1_at	1.24 q < 0.102	1.2 q < 1	2.34 q < 0.00114	1.32 q < 0.496	-1.24 q < 1	-1.03 q < 1
SLC36A2	solute carrier family 36 (proton/amino acid symporter), member 2	Ssc.14211.1.A1_at	1.11 q < 0.142	1.01 q < 1	1.8 q < 0.0000024	1.22 q < 0.322	-1.2 q < 1	-1.08 q < 1
CCL8	chemokine (C-C motif) ligand 8	Ssc.9957.1.A1_at	-1.23 q < 0.102	-1.13 q < 1	-1.06 q < 0.973	-1.02 q < 0.973	1.02 q < 1	-2.3 q < 0.000666
HPR	haptoglobin-related protein	Ssc.37.1.S1_at	-1.61 q < 0.104	1.1 q < 1	-1.02 q < 0.997	-1.2 q < 0.866	-2.17 q < 1	-4.49 q < 0.025
ASF1A	ASF1 anti-silencing function 1 homolog A (S. cerevisiae)	Ssc.8605.1.A1_s_at	1.11 q < 0.104	1.22 q < 0.468	-1.01 q < 0.995	1.36 q < 0.0233	1.02 q < 1	-1.01 q < 1
HPSE	heparanase	Ssc.7093.2.S1_at	1.17 q < 0.105	1.02 q < 1	1.08 q < 0.943	1.06 q < 0.865	1.64 q < 0.0379	1.14 q < 1
SPI1	spleen focus forming virus (SFFV) proviral integration oncogene spi1	Ssc.20177.1.S1_at	-1.14 q < 0.107	-1.05 q < 1	-1.09 q < 0.926	-1.04 q < 0.895	-1.07 q < 1	-1.54 q < 0.0227
SUCLA2	succinate-CoA ligase, ADP-forming, beta subunit	Ssc.68.1.A1_at	-1.19 q < 0.107	-1.01 q < 1	-1.69 q < 0.0414	1.07 q < 0.86	-1.19 q < 1	-1.28 q < 1
PPARD	peroxisome proliferator-activated receptor delta	Ssc.3251.1.A1_at	1.1 q < 0.108	-1.02 q < 1	1.14 q < 0.698	1.32 q < 0.0371	1.03 q < 1	1.06 q < 1
CXCR4	chemokine (C-X-C motif) receptor 4	Ssc.7176.1.A1_at	1.2 q < 0.109	1.02 q < 1	-1.15 q < 0.9	1.07 q < 0.883	1.13 q < 1	2.35 q < 0.0000536
AHCY	adenosylhomocysteinase	Ssc.25314.1.S1_a_at	1.14 q < 0.109	1.68 q < 0.00125	-1.04 q < 0.973	1.06 q < 0.852	1.16 q < 1	-1.04 q < 1
PIK3C2B	phosphoinositide-3-kinase, class 2, beta polypeptide	Ssc.3244.1.S1_at	-1.13 q < 0.11	1.05 q < 1	-1.23 q < 0.539	-1.42 q < 0.0493	-1.11 q < 1	-1.02 q < 1
SLC35F2	solute carrier family 35, member F2	Ssc.4329.1.S1_at	1.12 q < 0.11	1 q < 1	1.07 q < 0.933	1.51 q < 0.00482	1.02 q < 1	1.08 q < 1
EEF2	eukaryotic translation elongation factor 2	Ssc.965.1.S1_at	-1.08 q < 0.111	-1 q < 1	-1.27 q < 0.025	1.02 q < 0.913	-1.03 q < 1	-1.14 q < 0.734
COL21A1	collagen, type XXI, alpha 1	Ssc.24436.1.A1_at	1.09 q < 0.112	1.02 q < 1	1.1 q < 0.796	1.27 q < 0.0493	1.01 q < 1	1.05 q < 1
SLC19A1	solute carrier family 19 (folate transporter), member 1	Ssc.3044.1.S1_at	1.18 q < 0.113	1.07 q < 1	-1.17 q < 0.865	1.61 q < 0.0448	1.2 q < 1	1.31 q < 0.844
CDH13	cadherin 13, H-cadherin (heart)	Ssc.12939.1.S1_at	-1.13 q < 0.113	-1.12 q < 1	-1.48 q < 0.0274	-1.33 q < 0.141	-1.06 q < 1	1.25 q < 0.634
PDK4	pyruvate dehydrogenase kinase, isozyme 4	Ssc.1121.1.S1_at	-1.15 q < 0.115	-1.03 q < 1	-1.54 q < 0.0297	1.11 q < 0.731	-1.29 q < 1	-1.08 q < 1
UTRN	utrophin	Ssc.24741.1.A1_at	1.12 q < 0.115	1.57 q < 0.00108	-1.04 q < 0.969	1.09 q < 0.713	1.07 q < 1	-1.02 q < 1
FKBP8	FK506 binding protein 8, 38kDa	Ssc.16869.1.S1_at	1.09 q < 0.115	1.14 q < 0.82	-1.12 q < 0.739	1.31 q < 0.0241	1.15 q < 1	1.01 q < 1
EEF1A2	eukaryotic translation elongation factor 1 alpha 2	Ssc.17324.1.A1_at	-1.22 q < 0.117	-1 q < 1	1.08 q < 0.956	-1.29 q < 0.523	-1.2 q < 1	-1.89 q < 0.0257
OLFM1	olfactomedin 1	Ssc.17449.1.S1_at	1.09 q < 0.119	1.46 q < 0.000507	1.13 q < 0.708	1.01 q < 0.956	-1.02 q < 1	-1.05 q < 1
RAG1AP1	recombination activating gene 1 activating protein 1	Ssc.18227.1.S1_a_at	-1.09 q < 0.119	1.05 q < 1	-1.28 q < 0.0406	-1.06 q < 0.756	-1.06 q < 1	-1.09 q < 1
CN158086	-	Ssc.26710.1.S1_at	1.1 q < 0.12	-1.1 q < 1	1.02 q < 0.983	1.21 q < 0.269	-1.03 q < 1	1.5 q < 0.00103
PTHR1	parathyroid hormone 1 receptor	Ssc.14485.1.S1_at	1.11 q < 0.121	1.19 q < 0.767	-1.06 q < 0.938	-1.08 q < 0.754	1.13 q < 1	1.45 q < 0.0116
COG3	component of oligomeric golgi complex 3	Ssc.2241.1.A1_at	1.15 q < 0.121	-1.08 q < 1	1.13 q < 0.884	1.5 q < 0.0396	1.08 q < 1	1.18 q < 1
C9orf58	allograft inflammatory factor 1-like	Ssc.21247.2.S1_a_at	-1.13 q < 0.124	1 q < 1	-1.48 q < 0.0258	-1.31 q < 0.183	1.05 q < 1	1.01 q < 1
RAB8A	RAB8A, member RAS oncogene family	Ssc.24907.1.S1_at	-1.13 q < 0.126	-1.05 q < 1	-1.49 q < 0.0188	1.12 q < 0.659	-1.01 q < 1	-1.28 q < 0.479
DALRD3	DALR anticodon binding domain containing 3	Ssc.12434.2.S1_a_at	-1.12 q < 0.126	1.01 q < 1	-1.45 q < 0.0192	-1.12 q < 0.612	-1 q < 1	-1.08 q < 1
PLAUR	plasminogen activator, urokinase receptor	Ssc.10351.1.A1_at	1.15 q < 0.128	-1.11 q < 1	1.26 q < 0.537	1.57 q < 0.0153	1.12 q < 1	-1.01 q < 1
FAM78B	family with sequence similarity 78, member B	Ssc.9663.1.A1_at	1.1 q < 0.13	-1.08 q < 1	1.01 q < 0.997	1.38 q < 0.0222	1.08 q < 1	1.19 q < 0.753
LAP3	leucine aminopeptidase 3	Ssc.8775.3.S1_at	-1.14 q < 0.13	1.07 q < 1	-1.57 q < 0.0121	-1.02 q < 0.948	1.01 q < 1	-1.28 q < 0.619
LAP3	leucine aminopeptidase 3	Ssc.7713.1.A1_at	-1.1 q < 0.31	1.1 q < 1	-1.64 q < 0.00066	1.1 q < 0.708	1.02 q < 1	-1.19 q < 0.934
C4BPA	complement component 4 binding protein, alpha	Ssc.11162.1.S1_at	-1.11 q < 0.131	-1.06 q < 1	-1.01 q < 0.992	-1.03 q < 0.914	1.02 q < 1	-1.57 q < 0.000898
NDUFA2	NADH dehydrogenase (ubiquinone) 1 alpha subcomplex, 2, 8kDa	Ssc.8894.1.A1_at	1.09 q < 0.131	1.01 q < 1	-1.03 q < 0.973	1.39 q < 0.00463	1.03 q < 1	1.1 q < 1
SNF1LK2	salt-inducible kinase 2	Ssc.13078.1.A1_at	1.14 q < 0.133	1.2 q < 0.918	-1.13 q < 0.859	1.52 q < 0.0183	1.05 q < 1	1.12 q < 1
NRP2	neuropilin 2	Ssc.30598.1.A1_at	1.09 q < 0.134	1.4 q < 0.00462	-1.01 q < 0.996	1.03 q < 0.895	-1.01 q < 1	1.09 q < 1
NRP2	neuropilin 2	Ssc.24899.1.S1_at	1.09 q < 0.486	1.84 q < 0.000103	-1.13 q < 0.864	-1.14 q < 0.628	-1.06 q < 1	1.14 q < 1
NRP2	NEL-like 2 (chicken)	Ssc.30598.1.A1_at	1.09 q < 0.134	1.4 q < 0.00462	-1.01 q < 0.996	1.03 q < 0.895	-1.01 q < 1	1.09 q < 1
NRP2	NEL-like 2 (chicken)	Ssc.24899.1.S1_at	1.09 q < 0.486	1.84 q < 0.000103	-1.13 q < 0.864	-1.14 q < 0.628	-1.06 q < 1	1.14 q < 1
IER3	immediate early response 3	Ssc.5182.1.A1_at	1.1 q < 0.136	-1.03 q < 1	1.03 q < 0.965	1.36 q < 0.0283	1.03 q < 1	1.15 q < 0.973
HMGA1	high mobility group AT-hook 1	Ssc.1289.1.S1_at	1.19 q < 0.138	1.75 q < 0.0472	-1.17 q < 0.885	1.44 q < 0.249	1.08 q < 1	1.05 q < 1

Gene Name	Gene Description	Probe Set ID	Breed MS-WC	Day 25 MS-WC	Day 45 MS-WC	Day 65 MS-WC	Day 85 MS-WC	Day 105 MS-WC
MLH1	mutL homolog 1, colon cancer, nonpolyposis type 2 (E. coli)	Ssc.3701.1.S1_at	1.1 q < 0.142	1.15 q < 0.897	-1.07 q < 0.923	1.51 q < 0.000502	-1.02 q < 1	1 q < 1
BMP7	bone morphogenetic protein 7	Ssc.21774.1.S1_at	1.13 q < 0.142	1.05 q < 1	1.1 q < 0.91	1.43 q < 0.0438	1.06 q < 1	1.03 q < 1
TTLL7	tubulin tyrosine ligase-like family, member 7	Ssc.13381.1.A1_at	-1.09 q < 0.142	-1.35 q < 0.013	1.03 q < 0.958	-1.15 q < 0.398	-1.01 q < 1	1 q < 1
EEF1B2	eukaryotic translation elongation factor 1 beta 2	Ssc.6663.1.A1_at	-1.07 q < 0.143	1.04 q < 1	-1.16 q < 0.31	-1.02 q < 0.931	1.03 q < 1	-1.28 q < 0.0128
C12orf43	chromosome 12 open reading frame 43	Ssc.2821.1.A1_at	1.08 q < 0.143	1.01 q < 1	1 q < 0.997	1.36 q < 0.0011	1.04 q < 1	1.01 q < 1
TMEM123	transmembrane protein 123	Ssc.7785.1.S1_at	1.19 q < 0.148	1.35 q < 0.725	-1.18 q < 0.878	1.8 q < 0.0177	1.26 q < 1	-1.09 q < 1
C19orf63	chromosome 19 open reading frame 63	Ssc.23128.1.S1_at	1.1 q < 0.15	1.1 q < 1	-1.11 q < 0.829	1.32 q < 0.0433	1.11 q < 1	1.08 q < 1
DCLK1	doublecortin-like kinase 1	Ssc.29980.1.S1_at	-1.08 q < 0.152	1.01 q < 1	-1.02 q < 0.972	1.03 q < 0.858	-1.13 q < 1	-1.3 q < 0.0133
PRKACB	protein kinase, cAMP-dependent, catalytic, beta	Ssc.8658.1.A1_at	1.13 q < 0.152	1.12 q < 1	-1.1 q < 0.913	1.56 q < 0.0123	1.1 q < 1	1.07 q < 1
PRDM2	PR domain containing 2, with ZNF domain	Ssc.13143.1.S1_at	1.09 q < 0.154	1.04 q < 1	-1.1 q < 0.839	1.37 q < 0.011	1.09 q < 1	1.1 q < 1
COX15	COX15 homolog, cytochrome c oxidase assembly protein (yeast)	Ssc.28787.1.S1_at	1.1 q < 0.157	-1.01 q < 1	-1.06 q < 0.934	1.36 q < 0.0316	1.19 q < 1	1.06 q < 1
ERO1L	ERO1-like (S. cerevisiae)	Ssc.2709.1.S1_at	-1.13 q < 0.16	-1.6 q < 0.00758	-1.01 q < 0.996	-1.11 q < 0.722	1.01 q < 1	-1.04 q < 1
CLDN8	claudin 8	Ssc.12228.1.A1_at	1.23 q < 0.161	1.33 q < 1	1.17 q < 0.911	2.01 q < 0.0242	-1.04 q < 1	-1.06 q < 1
NANOS1	nanos homolog 1 (Drosophila)	Ssc.29246.1.A1_at	1.18 q < 0.161	-1.15 q < 1	1 q < 0.997	1.76 q < 0.0153	1.17 q < 1	1.25 q < 1
MAGI1	membrane associated guanylate kinase, WW and PDZ domain containing 1	Ssc.19060.1.A1_at	1.06 q < 0.164	1.14 q < 0.441	1.06 q < 0.878	1.02 q < 0.92	-1.11 q < 1	1.23 q < 0.0301
MAGI1	CNKSR family member 3	Ssc.19060.1.A1_at	1.06 q < 0.164	1.14 q < 0.441	1.06 q < 0.878	1.02 q < 0.92	-1.11 q < 1	1.23 q < 0.0301
HIST1H2AK	histone cluster 1, H2ak	Ssc.3070.1.S1_at	-1.12 q < 0.165	1.43 q < 0.0545	-1.12 q < 0.877	-1.37 q < 0.0832	1.01 q < 1	-1.65 q < 0.000666
MAPKBP1	mitogen-activated protein kinase binding protein 1	Ssc.2845.1.S1_at	1.14 q < 0.165	1.03 q < 1	-1.14 q < 0.872	1.7 q < 0.00269	1.07 q < 1	1.17 q < 1
MAPKBP1	mitogen-activated protein kinase binding protein 1	Ssc.2845.2.S1_at	1.04 q < 0.866	-1.01 q < 1	-1.15 q < 0.832	1.55 q < 0.0147	-1.11 q < 1	1.01 q < 1
TRAK2	trafficking protein, kinesin binding 2	Ssc.7277.1.A1_at	1.13 q < 0.166	1.15 q < 1	-1.12 q < 0.893	1.49 q < 0.0364	1.1 q < 1	1.11 q < 1
CSNK1D	casein kinase 1, delta	Ssc.9746.1.S1_a_at	1.09 q < 0.166	1.13 q < 0.941	-1.06 q < 0.917	1.32 q < 0.0242	1.11 q < 1	-1.03 q < 1
PPM1E	protein phosphatase, Mg2+/Mn2+ dependent, 1E	Ssc.18519.1.S1_at	-1.08 q < 0.167	1.02 q < 1	-1.42 q < 0.000323	-1.12 q < 0.48	-1.04 q < 1	1.12 q < 1
ALB	albumin	Ssc.10439.1.S1_at	-1.72 q < 0.17	1.01 q < 1	-1.03 q < 0.997	-1.88 q < 0.596	-1.08 q < 1	-7.26 q < 0.0154
CSDE1	cold shock domain containing E1, RNA-binding	Ssc.7628.1.A1_at	1.11 q < 0.17	1.17 q < 0.927	1.02 q < 0.989	1.39 q < 0.0487	-1.11 q < 1	1.14 q < 1
NENF	neuron derived neurotrophic factor	Ssc.11715.1.A1_at	-1.09 q < 0.173	1.05 q < 1	-1.38 q < 0.0162	-1.07 q < 0.759	-1.11 q < 1	-1 q < 1
PLEKHA5	pleckstrin homology domain containing, family A member 5	Ssc.29424.1.A1_at	1.17 q < 0.174	-1.06 q < 1	-1.05 q < 0.972	1.68 q < 0.0329	1.27 q < 1	1.15 q < 1
PUM1	pumilio homolog 1 (Drosophila)	Ssc.3364.2.S1_at	1.07 q < 0.177	1.01 q < 1	-1.14 q < 0.454	1.09 q < 0.56	1.07 q < 1	1.33 q < 0.00152
PRKRIP1	PRKR interacting protein 1 (IL11 inducible)	Ssc.18421.1.A1_at	1.09 q < 0.177	-1.04 q < 1	1.04 q < 0.946	1.31 q < 0.0448	1.06 q < 1	1.1 q < 1
UTY	ubiquitously transcribed tetratricopeptide repeat gene, Y-linked	Ssc.26959.1.S1_at	1.13 q < 0.178	1.07 q < 1	-1.05 q < 0.956	1.57 q < 0.00893	1.03 q < 1	1.11 q < 1
UNC5B	unc-5 homolog B (C. elegans)	Ssc.30844.1.A1_at	1.1 q < 0.178	-1.01 q < 1	-1.13 q < 0.766	1.43 q < 0.0067	1.03 q < 1	1.24 q < 0.391
HAPIP	kalirin, RhoGEF kinase	Ssc.7865.1.A1_at	-1.13 q < 0.178	-1.52 q < 0.0383	-1.03 q < 0.981	-1.02 q < 0.965	-1.12 q < 1	-1.04 q < 1
HSPB6	heat shock protein, alpha-crystallin-related, B6	Ssc.13823.1.A1_at	-1.11 q < 0.183	-1.24 q < 0.542	-1.42 q < 0.0398	1.33 q < 0.118	-1.05 q < 1	-1.21 q < 0.842
SPTB	spectrin, beta, erythrocytic	Ssc.25211.1.S1_at	1.11 q < 0.185	1.41 q < 0.0465	-1 q < 0.997	1.11 q < 0.651	1.01 q < 1	1.06 q < 1
SHROOM2	shroom family member 2	Ssc.21689.1.S1_at	1.07 q < 0.187	1.07 q < 1	-1.02 q < 0.985	1.1 q < 0.542	-1.06 q < 1	1.31 q < 0.0137
STAU2	staufen, RNA binding protein, homolog 2 (Drosophila)	Ssc.7242.1.A1_at	1.15 q < 0.188	-1.08 q < 1	1.06 q < 0.958	1.54 q < 0.0471	1.18 q < 1	1.11 q < 1
NME1	non-metastatic cells 1, protein (NM23A) expressed in	Ssc.19546.1.S1_at	-1.09 q < 0.188	1.34 q < 0.0592	-1.23 q < 0.284	-1.21 q < 0.272	1.01 q < 1	-1.42 q < 0.0076
NME1	RNA component of mitochondrial RNA processing endoribonuclease	Ssc.19546.1.S1_at	-1.09 q < 0.188	1.34 q < 0.0592	-1.23 q < 0.284	-1.21 q < 0.272	1.01 q < 1	-1.42 q < 0.0076
CDC7	cell division cycle 7 homolog (S. cerevisiae)	Ssc.26024.1.S1_at	1.13 q < 0.189	1.04 q < 1	-1.04 q < 0.974	1.51 q < 0.0233	1.06 q < 1	1.13 q < 1
SDS	serine dehydratase	Ssc.15246.1.S1_at	1.07 q < 0.189	-1.08 q < 1	1.09 q < 0.771	1.3 q < 0.00492	-1.01 q < 1	1.07 q < 1
SDS	Shwachman-Bodian-Diamond syndrome	Ssc.15246.1.S1_at	1.07 q < 0.189	-1.08 q < 1	1.09 q < 0.771	1.3 q < 0.00492	-1.01 q < 1	1.07 q < 1
STX2	syntaxin 2	Ssc.24069.1.S1_at	1.08 q < 0.19	1 q < 1	1.02 q < 0.975	1.02 q < 0.921	1.06 q < 1	1.3 q < 0.022
PAX5	paired box 5	Ssc.19194.1.A1_at	1.09 q < 0.196	1.21 q < 0.511	-1.2 q < 0.438	1.23 q < 0.198	-1.14 q < 1	1.42 q < 0.00747
JUN	jun proto-oncogene	Ssc.22550.1.A1_at	1.08 q < 0.197	-1.01 q < 1	1.06 q < 0.928	1.34 q < 0.0124	-1.01 q < 1	1.06 q < 1
TMEM2	transmembrane protein 2	Ssc.24637.1.A1_at	1.08 q < 0.197	1.05 q < 1	1.02 q < 0.979	1.33 q < 0.022	1.02 q < 1	1.03 q < 1
CYP4F3	cytochrome P450, family 4, subfamily F, polypeptide 3	Ssc.15069.1.S1_at	1.13 q < 0.2	1.07 q < 1	1.32 q < 0.34	-1.28 q < 0.308	1.05 q < 1	1.59 q < 0.0157
WDR47	WD repeat domain 47	Ssc.14429.1.A1_at	1.06 q < 0.201	1.07 q < 1	-1.09 q < 0.751	1.32 q < 0.000726	-1.06 q < 1	1.1 q < 0.994
KYNU	kynureninase (L-kynurenine hydrolase)	Ssc.7275.2.A1_at	-1.1 q < 0.202	1.11 q < 1	-1.06 q < 0.938	-1.01 q < 0.975	-1.11 q < 1	-1.48 q < 0.00355

Gene Name	Gene Description	Probe Set ID	Breed MS-WC	Day 25 MS-WC	Day 45 MS-WC	Day 65 MS-WC	Day 85 MS-WC	Day 105 MS-WC
HRSP12	heat-responsive protein 12	Ssc.8236.1.S1_at	1.18 q < 0.202	1.22 q < 1	-1.06 q < 0.969	1.74 q < 0.0404	1.04 q < 1	1.12 q < 1
POLR2E	polymerase (RNA) II (DNA directed) polypeptide E, 25kDa	Ssc.7464.1.S1_at	1.09 q < 0.204	1.63 q < 0.0000322	-1.11 q < 0.845	-1.03 q < 0.896	1.07 q < 1	1.02 q < 1
ELOVL5	ELOVL family member 5, elongation of long chain fatty acids (FEN1/Elo2, SUR4/Elo3-like, yeast)	Ssc.28365.1.A1_at	1.09 q < 0.205	-1.01 q < 1	1.07 q < 0.911	1.38 q < 0.00846	-1.03 q < 1	1.06 q < 1
NME6	non-metastatic cells 6, protein expressed in (nucleoside-diphosphate kinase)	Ssc.1496.1.S1_at	-1.08 q < 0.205	1.01 q < 1	-1.15 q < 0.579	1.09 q < 0.629	-1.4 q < 0.00643	-1.02 q < 1
RPL37	ribosomal protein L37	Ssc.925.1.S1_at	1.12 q < 0.205	-1.03 q < 1	1.07 q < 0.939	1.6 q < 0.00463	-1.04 q < 1	1.1 q < 1
ADAM5P	ADAM metallopeptidase domain 5, pseudogene	Ssc.8569.1.A1_at	-1.12 q < 0.205	1.15 q < 1	-1.15 q < 0.844	1.04 q < 0.905	-1.16 q < 1	-1.62 q < 0.00627
C1orf158	chromosome 1 open reading frame 158	Ssc.17976.1.A1_at	1.08 q < 0.207	-1.03 q < 1	1.03 q < 0.968	1.39 q < 0.00595	1.05 q < 1	1.04 q < 1
AKAP12	A kinase (PRKA) anchor protein 12	Ssc.7802.1.A1_at	1.18 q < 0.209	2.01 q < 0.00342	-1.07 q < 0.958	-1.14 q < 0.749	-1.03 q < 1	1.39 q < 0.674
Q8N799	-	Ssc.4986.2.S1_at	1.07 q < 0.21	1.04 q < 1	1.02 q < 0.967	1.24 q < 0.0453	1 q < 1	1.04 q < 1
NEB	nebulin	Ssc.14277.1.A1_at	-1.12 q < 0.214	-1.28 q < 0.493	-1.24 q < 0.529	1.28 q < 0.257	-1.61 q < 0.00643	1.15 q < 1
THBS1	thrombospondin 1	Ssc.10406.1.A1_at	-1.09 q < 0.214	-1.39 q < 0.0275	1.01 q < 0.996	-1.02 q < 0.94	-1.14 q < 1	1.03 q < 1
CPNE2	copine II	Ssc.5428.1.A1_at	-1.08 q < 0.215	-1.4 q < 0.00174	-1.07 q < 0.9	-1.05 q < 0.775	-1.06 q < 1	1.15 q < 0.768
IGHA2	immunoglobulin heavy constant alpha 2 (A2m marker)	Ssc.17718.1.S1_at	-1.11 q < 0.215	1.06 q < 1	-1.1 q < 0.905	1.06 q < 0.839	-1 q < 1	-1.73 q < 0.000133
PHLDA3	pleckstrin homology-like domain, family A, member 3	Ssc.3079.1.S1_at	-1.07 q < 0.221	-1.17 q < 0.465	-1.2 q < 0.212	1.33 q < 0.00532	-1.24 q < 0.147	-1.07 q < 1
NME2	non-metastatic cells 2, protein (NM23B) expressed in	Ssc.2257.1.S1_at	-1.11 q < 0.229	1.22 q < 0.71	-1.32 q < 0.22	-1.12 q < 0.658	1.04 q < 1	-1.45 q < 0.0359
SDCBP2	syndecan binding protein (syntenin) 2	Ssc.5938.1.A1_at	-1.16 q < 0.23	1.03 q < 1	1.04 q < 0.982	-1.87 q < 0.00465	-1.03 q < 1	-1.15 q < 1
SLC23A2	solute carrier family 23 (nucleobase transporters), member 2	Ssc.22104.1.A1_at	-1.14 q < 0.231	1.01 q < 1	-1.96 q < 0.000419	1.02 q < 0.968	-1.02 q < 1	1 q < 1
SLC23A2	solute carrier family 23 (nucleobase transporters), member 1	Ssc.22104.1.A1_at	-1.14 q < 0.231	1.01 q < 1	-1.96 q < 0.000419	1.02 q < 0.968	-1.02 q < 1	1 q < 1
HKE2	prefoldin subunit 6	Ssc.21182.1.S1_at	-1.07 q < 0.239	1.06 q < 1	-1.08 q < 0.869	-1.03 q < 0.878	1.03 q < 1	-1.37 q < 0.00107
HEBP1	heme binding protein 1	Ssc.2306.1.S1_at	1.18 q < 0.241	2.68 q < 0.0000125	-1.04 q < 0.987	1.02 q < 0.972	-1.02 q < 1	-1.13 q < 1
ATP10D	ATPase, class V, type 10D	Ssc.9238.1.A1_at	1.15 q < 0.243	-1.02 q < 1	-1.08 q < 0.951	1.81 q < 0.00717	-1.01 q < 1	1.24 q < 1
PPP1R12C	protein phosphatase 1, regulatory (inhibitor) subunit 12C	Ssc.6360.1.S1_at	1.07 q < 0.245	1.06 q < 1	-1.05 q < 0.928	1.26 q < 0.0486	1.01 q < 1	1.1 q < 1
PPP1R3D	protein phosphatase 1, regulatory (inhibitor) subunit 3D	Ssc.2361.1.A1_at	1.11 q < 0.247	1.1 q < 1	1 q < 0.997	1.51 q < 0.00984	-1.02 q < 1	1.02 q < 1
FAM26F	family with sequence similarity 26, member F	Ssc.19365.1.S1_at	-1.11 q < 0.249	-1.01 q < 1	1.02 q < 0.989	1.05 q < 0.873	1.02 q < 1	-1.85 q < 0.0000462
ALDH1B1	aldehyde dehydrogenase 1 family, member B1	Ssc.25096.1.A1_at	1.09 q < 0.249	1.41 q < 0.0228	-1.02 q < 0.988	1.11 q < 0.63	-1.02 q < 1	1.03 q < 1
ATP5L	ATP synthase, H+ transporting, mitochondrial Fo complex, subunit G	Ssc.2957.1.S2_at	1.1 q < 0.256	1.08 q < 1	1 q < 0.999	1.49 q < 0.0085	-1.05 q < 1	1.04 q < 1
AQP9	aquaporin 9	Ssc.7234.1.A1_at	1.08 q < 0.257	1.19 q < 0.492	1.1 q < 0.812	1.3 q < 0.0325	1 q < 1	-1.19 q < 0.53
AQP9	aquaporin 7	Ssc.7234.1.A1_at	1.08 q < 0.257	1.19 q < 0.492	1.1 q < 0.812	1.3 q < 0.0325	1 q < 1	-1.19 q < 0.53
SLMO2	slowmo homolog 2 (Drosophila)	Ssc.1913.1.A1_at	1.18 q < 0.258	1.21 q < 1	-1.11 q < 0.94	1.8 q < 0.0441	1.18 q < 1	1 q < 1
C12orf36	chromosome 12 open reading frame 36	Ssc.27503.1.A1_at	1.09 q < 0.258	1.01 q < 1	1.1 q < 0.878	1.39 q < 0.0222	-1.04 q < 1	1.03 q < 1
NP_957712	-	Ssc.8731.1.S1_at	1.1 q < 0.263	1.15 q < 1	-1.1 q < 0.9	1.39 q < 0.0487	1.09 q < 1	1.02 q < 1
WBP4	WW domain binding protein 4 (formin binding protein 21)	Ssc.11213.1.S1_at	1.05 q < 0.263	1.03 q < 1	-1.01 q < 0.982	1.27 q < 0.00202	-1.08 q < 1	1.09 q < 1
MBNL1	muscleblind-like (Drosophila)	Ssc.10532.1.A1_at	1.09 q < 0.264	1.01 q < 1	-1.03 q < 0.977	1.47 q < 0.00617	1.06 q < 1	1.02 q < 1
YWHAE	tyrosine 3-monooxygenase/tryptophan 5-monooxygenase activation protein, epsilon polypeptide	Ssc.21462.1.S1_at	-1.14 q < 0.266	-1.54 q < 0.124	-1.24 q < 0.722	-1.31 q < 0.361	1.71 q < 0.0289	-1.32 q < 0.724
TPK1	thiamin pyrophosphokinase 1	Ssc.24771.1.A1_at	-1.1 q < 0.266	-1.01 q < 1	1.29 q < 0.253	-1.34 q < 0.0908	-1.47 q < 0.0251	-1.03 q < 1
CCNB3	cyclin B3	Ssc.26568.1.A1_s_at	1.07 q < 0.267	1.3 q < 0.0457	1.04 q < 0.945	1.03 q < 0.872	-1.03 q < 1	1.04 q < 1
SCARB2	scavenger receptor class B, member 2	Ssc.23484.2.S1_at	1.08 q < 0.269	-1.08 q < 1	-1.05 q < 0.943	1.36 q < 0.0246	1.12 q < 1	1.11 q < 1
PSME1	proteasome (prosome, macropain) activator subunit 1 (PA28 alpha)	Ssc.1163.1.S1_at	-1.09 q < 0.269	1.24 q < 0.384	-1.42 q < 0.0143	-1.09 q < 0.685	1.04 q < 1	-1.27 q < 0.291
IFT57	intraflagellar transport 57 homolog (Chlamydomonas)	Ssc.13576.2.S1_at	-1.07 q < 0.271	-1.33 q < 0.0237	-1.03 q < 0.97	1.02 q < 0.942	-1.04 q < 1	-1.03 q < 1
FAM107B	family with sequence similarity 107, member B	Ssc.14418.2.A1_at	1.07 q < 0.272	-1.05 q < 1	1.03 q < 0.969	1.28 q < 0.034	1.05 q < 1	1.07 q < 1
TP53INP1	tumor protein p53 inducible nuclear protein 1	Ssc.29750.1.A1_at	-1.1 q < 0.273	-1.55 q < 0.00497	-1.2 q < 0.625	-1.11 q < 0.672	1.01 q < 1	1.27 q < 0.49
POR	P450 (cytochrome) oxidoreductase	Ssc.2887.1.S1_at	1.13 q < 0.276	1.14 q < 1	-1.24 q < 0.658	1.54 q < 0.0378	1.15 q < 1	1.12 q < 1
POR	porcupine homolog (Drosophila)	Ssc.2887.1.S1_at	1.13 q < 0.276	1.14 q < 1	-1.24 q < 0.658	1.54 q < 0.0378	1.15 q < 1	1.12 q < 1
FAAH	fatty acid amide hydrolase	Ssc.2698.1.S1_at	1.07 q < 0.281	1.05 q < 1	1.06 q < 0.922	-1.03 q < 0.908	-1 q < 1	1.32 q < 0.0322

Gene Name	Gene Description	Probe Set ID	Breed MS-WC	Day 25 MS-WC	Day 45 MS-WC	Day 65 MS-WC	Day 85 MS-WC	Day 105 MS-WC
FAAH	fatty acid 2-hydroxylase	Ssc.2698.1.S1_at	1.07 q < 0.281	1.05 q < 1	1.06 q < 0.922	-1.03 q < 0.908	-1 q < 1	1.32 q < 0.0322
APRT	adenine phosphoribosyltransferase	Ssc.2191.1.S1_at	-1.08 q < 0.281	1.08 q < 1	-1.34 q < 0.0225	1.1 q < 0.591	-1.02 q < 1	-1.25 q < 0.187
MAPKAP1	mitogen-activated protein kinase associated protein 1	Ssc.21536.1.A1_at	-1.17 q < 0.282	-1.04 q < 1	-1.8 q < 0.0424	-1.17 q < 0.714	-1.26 q < 1	1.25 q < 1
KSR2	kinase suppressor of ras 2	Ssc.5331.1.S1_at	-1.1 q < 0.288	-1.06 q < 1	-1.61 q < 0.00224	-1.02 q < 0.94	-1.24 q < 1	1.33 q < 0.252
KSR2	connector enhancer of kinase suppressor of Ras 2	Ssc.5331.1.S1_at	-1.1 q < 0.288	-1.06 q < 1	-1.61 q < 0.00224	-1.02 q < 0.94	-1.24 q < 1	1.33 q < 0.252
INPP5D	inositol polyphosphate-5-phosphatase, 145kDa	Ssc.19063.1.S1_at	1.11 q < 0.291	-1.07 q < 1	1.11 q < 0.893	1.53 q < 0.0153	1.06 q < 1	-1 q < 1
DOCK9	dedicator of cytokinesis 9	Ssc.8919.1.S1_at	1.07 q < 0.298	1.02 q < 1	1.03 q < 0.958	1.28 q < 0.0423	1.07 q < 1	-1.03 q < 1
CCDC15	coiled-coil domain containing 15	Ssc.21272.1.A1_at	-1.15 q < 0.298	-1.28 q < 0.921	-1.69 q < 0.0456	1.14 q < 0.736	-1.19 q < 1	1.13 q < 1
NUDCD2	NudC domain containing 2	Ssc.6107.1.S1_at	1.09 q < 0.301	1.04 q < 1	-1.02 q < 0.989	1.4 q < 0.0219	-1.02 q < 1	1.08 q < 1
SNORA32	small nucleolar RNA, H/ACA box 32	Ssc.489.1.A1_at	-1.12 q < 0.302	-2.59 q < 0.000000000692	1.09 q < 0.926	1.23 q < 0.461	-1.15 q < 1	1.28 q < 0.747
IGF2AS	insulin-like growth factor 2 antisense	Ssc.9365.6.A1_a_at	-1.07 q < 0.304	-1.25 q < 0.214	-1.33 q < 0.0315	1.18 q < 0.321	-1.04 q < 1	1.02 q < 1
STMN1	stathmin 1	Ssc.7701.1.A1_at	-1.13 q < 0.305	1.4 q < 0.333	-1.29 q < 0.529	-1.15 q < 0.648	-1.06 q < 1	-1.6 q < 0.0343
RARRES3	retinoic acid receptor responder (tazarotene induced) 3	Ssc.24766.1.S1_at	1.15 q < 0.307	1.08 q < 1	-1.33 q < 0.606	2.09 q < 0.000966	-1.04 q < 1	1.24 q < 1
SLC39A14	solute carrier family 39 (zinc transporter), member 14	Ssc.19427.1.S1_at	1.07 q < 0.308	-1.03 q < 1	1.01 q < 0.991	1.27 q < 0.0349	-1.01 q < 1	1.1 q < 1
FGFBP3	fibroblast growth factor binding protein 3	Ssc.25031.1.S1_at	1.09 q < 0.312	1.03 q < 1	-1 q < 0.999	1.09 q < 0.736	-1.02 q < 1	1.42 q < 0.0408
MARCO	macrophage receptor with collagenous structure	Ssc.19357.1.S1_at	-1.1 q < 0.317	-1.04 q < 1	1.01 q < 0.993	1.07 q < 0.796	-1.03 q < 1	-1.6 q < 0.00163
CCDC56	coiled-coil domain containing 56	Ssc.3838.1.S1_at	1.08 q < 0.318	1.53 q < 0.000735	-1.04 q < 0.961	-1.01 q < 0.977	1.06 q < 1	-1.05 q < 1
BTG3	BTG family, member 3	Ssc.9666.1.S1_at	1.11 q < 0.318	-1.03 q < 1	-1.29 q < 0.457	1.78 q < 0.000904	1.03 q < 1	1.26 q < 0.803
NP_694539	-	Ssc.13556.1.A1_at	1.09 q < 0.32	1.48 q < 0.0158	-1.23 q < 0.487	1.2 q < 0.415	-1.01 q < 1	1.09 q < 1
CCNG1	cyclin G1	Ssc.5070.1.A1_at	1.12 q < 0.321	1.04 q < 1	-1.13 q < 0.894	1.57 q < 0.0307	1.17 q < 1	1.04 q < 1
PI4K2B	phosphatidylinositol 4-kinase type 2 beta	Ssc.17355.1.A1_at	1.1 q < 0.324	-1.09 q < 1	1.02 q < 0.987	1.43 q < 0.0456	1.01 q < 1	1.19 q < 1
FOLR1	folate receptor 1 (adult)	Ssc.11779.1.S1_at	-1.16 q < 0.332	-1.11 q < 1	-1.16 q < 0.903	1.13 q < 0.778	1.21 q < 1	-2.21 q < 0.000881
CAMK2N1	calcium/calmodulin-dependent protein kinase II inhibitor 1	Ssc.6447.1.A1_at	-1.09 q < 0.334	1.13 q < 1	-1.55 q < 0.00216	1.08 q < 0.756	-1.14 q < 1	-1.05 q < 1
QDPR	quinoid dihydropteridine reductase	Ssc.17250.1.S1_a_at	1.11 q < 0.339	1.64 q < 0.00762	-1.08 q < 0.937	1.01 q < 0.979	-1.02 q < 1	1.11 q < 1
TNKS1BP1	tankyrase 1 binding protein 1, 182kDa	Ssc.3430.1.A1_at	1.08 q < 0.34	1 q < 1	-1.02 q < 0.981	1.41 q < 0.0147	1.08 q < 1	-1.01 q < 1
GPR175	transmembrane protein, adipocyte asscociated 1	Ssc.20244.1.S1_at	1.07 q < 0.344	-1.02 q < 1	-1.08 q < 0.885	1.32 q < 0.0212	1.01 q < 1	1.14 q < 0.925
MGMT	O-6-methylguanine-DNA methyltransferase	Ssc.19639.1.A1_at	-1.06 q < 0.344	-1.1 q < 1	-1.33 q < 0.0125	1 q < 0.985	1.01 q < 1	1.05 q < 1
KMO	kynurenine 3-monooxygenase (kynurenine 3-hydroxylase)	Ssc.283.1.S1_at	-1.09 q < 0.346	1 q < 1	1.06 q < 0.942	-1 q < 0.997	-1.08 q < 1	-1.52 q < 0.00787
EDC4	enhancer of mRNA decapping 4	Ssc.948.1.A1_at	1.06 q < 0.353	-1.01 q < 1	1.03 q < 0.961	1.26 q < 0.0496	-1 q < 1	1.06 q < 1
PPA2	pyrophosphatase (inorganic) 2	Ssc.30813.1.A1_at	-1.07 q < 0.353	1.03 q < 1	-1.33 q < 0.0468	-1.07 q < 0.735	-1.01 q < 1	-1.02 q < 1
LONRF3	LON peptidase N-terminal domain and ring finger 3	Ssc.10329.1.S1_at	1.08 q < 0.354	-1.09 q < 1	1.04 q < 0.949	1.13 q < 0.524	-1.05 q < 1	1.39 q < 0.0146
FLRT2	fibronectin leucine rich transmembrane protein 2	Ssc.9215.1.A1_at	-1.08 q < 0.354	-1.56 q < 0.00113	1.01 q < 0.996	1.02 q < 0.956	-1.02 q < 1	1.04 q < 1
CDKN1A	cyclin-dependent kinase inhibitor 1A (p21, Cip1)	Ssc.5737.1.S1_at	1.09 q < 0.357	-1.06 q < 1	1.01 q < 0.991	1.42 q < 0.0329	1.21 q < 1	-1.06 q < 1
AW437138	-	Ssc.20353.1.S1_at	-1.08 q < 0.357	-1.47 q < 0.00542	1.05 q < 0.946	-1.04 q < 0.873	-1.1 q < 1	1.09 q < 1
TCTN3	tectonic family member 3	Ssc.5488.1.S1_at	1.08 q < 0.358	1 q < 1	1.1 q < 0.866	1.45 q < 0.00478	-1.03 q < 1	-1.07 q < 1
IGFBP7	insulin-like growth factor binding protein 7	Ssc.17186.2.S2_at	-1.19 q < 0.36	1.5 q < 0.655	-2.4 q < 0.00407	-1.06 q < 0.926	-1.32 q < 1	-1.06 q < 1
FNBP4	formin binding protein 4	Ssc.12416.1.S1_a_at	1.08 q < 0.361	1 q < 1	-1.01 q < 0.994	1.41 q < 0.0191	-1.01 q < 1	1.07 q < 1
CD47	CD47 molecule	Ssc.17142.1.A1_s_at	1.12 q < 0.364	1.09 q < 1	-1.17 q < 0.844	1.59 q < 0.0325	-1.01 q < 1	1.2 q < 1
USF2	upstream transcription factor 2, c-fos interacting	Ssc.20441.2.S1_at	-1.07 q < 0.366	-1.01 q < 1	-1.33 q < 0.038	1.09 q < 0.664	-1.13 q < 1	-1.02 q < 1
EIF3E	eukaryotic translation initiation factor 3, subunit E	Ssc.15631.1.S1_at	-1.08 q < 0.369	1.11 q < 1	-1.42 q < 0.00971	1.03 q < 0.901	1.03 q < 1	-1.2 q < 0.646
SPDYA	speedy homolog A (Xenopus laevis)	Ssc.5232.2.A1_a_at	1.11 q < 0.369	1.05 q < 1	1 q < 1	1.51 q < 0.0372	1.08 q < 1	-1.03 q < 1
GJB3	gap junction protein, beta 3, 31kDa	Ssc.21830.1.A1_at	1.13 q < 0.381	-1.03 q < 1	1.2 q < 0.841	1.66 q < 0.0344	1.12 q < 1	-1.17 q < 1
ZC3HAV1	zinc finger CCCH-type, antiviral 1	Ssc.20242.1.A1_at	-1.07 q < 0.386	-1.15 q < 0.89	-1.38 q < 0.0217	1.03 q < 0.915	1.12 q < 1	-1.03 q < 1
MRPL52	mitochondrial ribosomal protein L52	Ssc.3811.1.S1_a_at	1.06 q < 0.387	1.01 q < 1	-1.04 q < 0.942	1.32 q < 0.00682	1.04 q < 1	1 q < 1
MRAP2	melanocortin 2 receptor accessory protein 2	Ssc.17375.1.S1_at	-1.13 q < 0.392	-1.89 q < 0.00722	-1.25 q < 0.761	1.1 q < 0.814	1.2 q < 1	-1.05 q < 1
PTGS2	prostaglandin-endoperoxide synthase 2 (prostaglandin G/H synthase and cyclooxygenase)	Ssc.7314.1.A1_at	1.18 q < 0.393	-1.08 q < 1	-1.03 q < 0.99	1.98 q < 0.0424	1.19 q < 1	1.1 q < 1
LMNB1	lamin B1	Ssc.21730.1.S1_at	1.08 q < 0.397	1.75 q < 0.000014	-1.07 q < 0.933	1.02 q < 0.937	-1.03 q < 1	-1.09 q < 1

Gene Name	Gene Description	Probe Set ID	Breed MS-WC	Day 25 MS-WC	Day 45 MS-WC	Day 65 MS-WC	Day 85 MS-WC	Day 105 MS-WC
VKORC1L1	vitamin K epoxide reductase complex, subunit 1-like 1	Ssc.21554.1.A1_at	1.08 q < 0.401	1.08 q < 1	-1.04 q < 0.958	1.39 q < 0.036	1.04 q < 1	-1.01 q < 1
SMYD3	SET and MYND domain containing 3	Ssc.28198.1.A1_at	1.07 q < 0.402	-1.02 q < 1	-1.02 q < 0.989	1.39 q < 0.0116	-1.01 q < 1	1.06 q < 1
IRF2BP2	interferon regulatory factor 2 binding protein 2	Ssc.10096.2.A1_at	1.08 q < 0.404	-1.09 q < 1	1.02 q < 0.985	1.45 q < 0.0106	-1.05 q < 1	1.13 q < 1
AGPS	alkylglycerone phosphate synthase	Ssc.25474.1.S1_at	1.06 q < 0.408	1.13 q < 0.91	-1.09 q < 0.844	1.34 q < 0.015	1.05 q < 1	-1.06 q < 1
HRB	ArfGAP with FG repeats 1	Ssc.24102.1.S1_at	-1.11 q < 0.414	1 q < 1	-1.58 q < 0.0398	-1 q < 0.994	-1.02 q < 1	-1.05 q < 1
FHDC1	FH2 domain containing 1	Ssc.16516.1.S1_at	1.06 q < 0.423	-1.03 q < 1	-1.09 q < 0.818	1.28 q < 0.0325	1.02 q < 1	1.15 q < 0.747
UCHL1	ubiquitin carboxyl-terminal esterase L1 (ubiquitin thiolesterase)	Ssc.2073.1.A1_at	1.14 q < 0.427	3.22 q < 0.0000000301	-1.03 q < 0.988	-1.28 q < 0.523	1.08 q < 1	-1.36 q < 0.833
YY1	YY1 transcription factor	Ssc.9948.1.S1_at	1.07 q < 0.427	1.07 q < 1	-1.09 q < 0.878	1.35 q < 0.034	1.02 q < 1	1.04 q < 1
HPRT1	hypoxanthine phosphoribosyltransferase 1	Ssc.4158.1.S1_at	-1.09 q < 0.43	1.19 q < 0.972	-1.18 q < 0.749	-1 q < 0.993	-1.05 q < 1	-1.5 q < 0.0394
PLOD2	procollagen-lysine, 2-oxoglutarate 5-dioxygenase 2	Ssc.25095.1.S1_at	-1.07 q < 0.431	-1.41 q < 0.00635	1.06 q < 0.932	1.07 q < 0.754	-1.09 q < 1	-1.01 q < 1
RNF144B	ring finger protein 144B	Ssc.22566.1.S1_at	1.06 q < 0.435	-1.05 q < 1	1.08 q < 0.859	1.28 q < 0.0336	-1.02 q < 1	1.02 q < 1
EMB	embigin	Ssc.4182.1.A1_at	1.11 q < 0.441	1.13 q < 1	-1.17 q < 0.854	1.61 q < 0.0336	1.14 q < 1	-1.04 q < 1
EMB	exportin 1 (CRM1 homolog, yeast)	Ssc.4182.1.A1_at	1.11 q < 0.441	1.13 q < 1	-1.17 q < 0.854	1.61 q < 0.0336	1.14 q < 1	-1.04 q < 1
PER1	period homolog 1 (Drosophila)	Ssc.16979.1.S1_a_at	1.06 q < 0.443	-1.17 q < 0.665	-1.05 q < 0.937	1.42 q < 0.00111	1.01 q < 1	1.15 q < 0.848
PER1	period homolog 1 (Drosophila)	Ssc.16979.3.S1_a_at	1.03 q < 0.789	-1.11 q < 0.998	-1.09 q < 0.829	1.27 q < 0.0261	-1.02 q < 1	1.12 q < 0.931
PER1	post-GPI attachment to proteins 3	Ssc.16979.1.S1_a_at	1.06 q < 0.443	-1.17 q < 0.665	-1.05 q < 0.937	1.42 q < 0.00111	1.01 q < 1	1.15 q < 0.848
PER1	post-GPI attachment to proteins 3	Ssc.16979.3.S1_a_at	1.03 q < 0.789	-1.11 q < 0.998	-1.09 q < 0.829	1.27 q < 0.0261	-1.02 q < 1	1.12 q < 0.931
Q9H373	-	Ssc.7256.1.A1_at	-1.12 q < 0.443	-1.01 q < 1	-1.01 q < 0.997	-1.02 q < 0.973	-1.03 q < 1	-1.63 q < 0.0478
RBP1	retinol binding protein 1, cellular	Ssc.5563.1.S1_at	-1.12 q < 0.443	-1.34 q < 0.66	-1.05 q < 0.976	-1.78 q < 0.00967	-1.02 q < 1	1.44 q < 0.36
RBP1	AT rich interactive domain 4A (RBP1-like)	Ssc.5563.1.S1_at	-1.12 q < 0.443	-1.34 q < 0.66	-1.05 q < 0.976	-1.78 q < 0.00967	-1.02 q < 1	1.44 q < 0.36
RGS10	regulator of G-protein signaling 10	Ssc.5274.1.S1_at	-1.07 q < 0.448	1.28 q < 0.145	-1.13 q < 0.756	-1.06 q < 0.782	-1.07 q < 1	-1.37 q < 0.018
GBP1	guanylate binding protein 1, interferon-inducible, 67kDa	Ssc.29054.1.A1_at	-1.11 q < 0.448	1.27 q < 0.85	1.09 q < 0.94	1.04 q < 0.931	1.13 q < 1	-2.77 q < 0.0000000066
GBP1	guanylate binding protein 1, interferon-inducible, 67kDa	Ssc.29054.3.S1_at	-1.05 q < 0.702	1.09 q < 1	1.04 q < 0.961	1.15 q < 0.521	-1 q < 1	-1.66 q < 0.000078
DKK2	dickkopf homolog 2 (Xenopus laevis)	Ssc.30925.1.A1_at	-1.14 q < 0.449	-1.91 q < 0.0234	-1.02 q < 0.994	1.01 q < 0.992	1.09 q < 1	-1.09 q < 1
DERL2	Der1-like domain family, member 2	Ssc.16776.1.S1_at	1.07 q < 0.449	-1.02 q < 1	-1.1 q < 0.885	1.4 q < 0.0201	1.13 q < 1	1.01 q < 1
TAF4B	TAF4b RNA polymerase II, TATA box binding protein (TBP)-associated factor, 105kDa	Ssc.13547.1.A1_at	-1.11 q < 0.449	-1.01 q < 1	-1.73 q < 0.00914	1.17 q < 0.634	-1.21 q < 1	1.08 q < 1
TAF4B	PAPPA antisense RNA (non-protein coding)	Ssc.13547.1.A1_at	-1.11 q < 0.449	-1.01 q < 1	-1.73 q < 0.00914	1.17 q < 0.634	-1.21 q < 1	1.08 q < 1
GOT1	glutamic-oxaloacetic transaminase 1, soluble (aspartate aminotransferase 1)	Ssc.3528.1.S1_at	1.18 q < 0.452	1.18 q < 1	-1.75 q < 0.232	2.08 q < 0.036	1.32 q < 1	1.23 q < 1
GOT1	golgi transport 1B	Ssc.3528.1.S1_at	1.18 q < 0.452	1.18 q < 1	-1.75 q < 0.232	2.08 q < 0.036	1.32 q < 1	1.23 q < 1
GOT1	golgi transport 1A	Ssc.3528.1.S1_at	1.18 q < 0.452	1.18 q < 1	-1.75 q < 0.232	2.08 q < 0.036	1.32 q < 1	1.23 q < 1
SIGLEC10	sialic acid binding Ig-like lectin 10	Ssc.27233.1.S1_at	1.07 q < 0.452	1.38 q < 0.0477	1.03 q < 0.972	-1.08 q < 0.751	1.02 q < 1	1.06 q < 1
SCYE1	aminoacyl tRNA synthetase complex-interacting multifunctional protein 1	Ssc.9246.1.S1_at	-1.07 q < 0.452	1.06 q < 1	-1.37 q < 0.0378	1.03 q < 0.899	-1.03 q < 1	-1.09 q < 1
APC	adenomatous polyposis coli	Ssc.22176.1.A1_at	1.06 q < 0.454	-1.08 q < 1	1.1 q < 0.799	1.36 q < 0.00421	-1.02 q < 1	-1.04 q < 1
PPP1R9A	protein phosphatase 1, regulatory (inhibitor) subunit 9A	Ssc.10642.1.S1_at	1.11 q < 0.454	1.09 q < 1	-1.18 q < 0.844	1.57 q < 0.0457	-1 q < 1	1.16 q < 1
MAN1C1	mannosidase, alpha, class 1C, member 1	Ssc.19360.1.S1_at	1.09 q < 0.457	-1.01 q < 1	-1.11 q < 0.894	1.58 q < 0.00764	-1.07 q < 1	1.17 q < 1
TSPO	translocator protein (18kDa)	Ssc.5936.1.A1_a_at	1.13 q < 0.459	1.11 q < 1	1 q < 1	1.73 q < 0.0433	1.2 q < 1	-1.23 q < 1
FAM129B	family with sequence similarity 129, member B	Ssc.4690.1.S1_at	1.08 q < 0.46	1.07 q < 1	-1.1 q < 0.885	1.51 q < 0.0067	1.03 q < 1	-1.02 q < 1
ZNF365	zinc finger protein 365	Ssc.29840.1.A1_at	1.07 q < 0.461	-1.11 q < 1	-1.04 q < 0.968	1.49 q < 0.00329	1.18 q < 1	-1.08 q < 1
VDAC3	voltage-dependent anion channel 3	Ssc.13817.1.A1_at	-1.07 q < 0.462	1.26 q < 0.308	-1.41 q < 0.0144	-1.11 q < 0.634	-1.06 q < 1	-1.06 q < 1
VDAC3	voltage-dependent anion channel 1 pseudogene 5	Ssc.13817.1.A1_at	-1.07 q < 0.462	1.26 q < 0.308	-1.41 q < 0.0144	-1.11 q < 0.634	-1.06 q < 1	-1.06 q < 1
CEBPD	CCAAT/enhancer binding protein (C/EBP), delta	Ssc.10025.3.S1_at	1.09 q < 0.466	-1.03 q < 1	-1.07 q < 0.94	1.64 q < 0.004	-1.1 q < 1	1.15 q < 1
DPEP2	dipeptidase 2	Ssc.12793.1.A1_at	1.06 q < 0.466	-1.05 q < 1	1.05 q < 0.943	1.32 q < 0.0248	1.02 q < 1	-1 q < 1
SLC22A15	solute carrier family 22, member 15	Ssc.25054.1.S1_at	-1.07 q < 0.466	-1.03 q < 1	-1.35 q < 0.0306	-1.16 q < 0.433	-1.08 q < 1	1.27 q < 0.179
TMEM88	transmembrane protein 88	Ssc.18336.1.S1_at	1.09 q < 0.466	1.62 q < 0.00864	-1.28 q < 0.468	1.09 q < 0.761	-1.07 q < 1	1.2 q < 1
ENPP2	ectonucleotide pyrophosphatase/phosphodiesterase 2	Ssc.5142.1.S1_at	1.1 q < 0.467	1.08 q < 1	-1.01 q < 0.996	-1.16 q < 0.607	1.08 q < 1	1.58 q < 0.0215
CREM	cAMP responsive element modulator	Ssc.2841.1.S1_at	1.08 q < 0.469	1.05 q < 1	-1.25 q < 0.444	1.53 q < 0.00602	-1.05 q < 1	1.21 q < 0.849

Gene Name	Gene Description	Probe Set ID	Breed MS-WC	Day 25 MS-WC	Day 45 MS-WC	Day 65 MS-WC	Day 85 MS-WC	Day 105 MS-WC
GPR158	G protein-coupled receptor 158	Ssc.9985.1.A1_at	-1.11 q < 0.473	-1.05 q < 1	-1.74 q < 0.0108	1.36 q < 0.268	-1.14 q < 1	-1.09 q < 1
NAAA	N-acylethanolamine acid amidase	Ssc.24984.1.S1_at	1.08 q < 0.475	1.06 q < 1	-1.17 q < 0.746	1.47 q < 0.0264	-1.06 q < 1	1.19 q < 1
Q8TAD7	-	Ssc.6028.1.S1_at	-1.24 q < 0.479	1.63 q < 0.839	-1.43 q < 0.823	1.03 q < 0.975	-3.29 q < 0.00967	-1.03 q < 1
CDKN1C	cyclin-dependent kinase inhibitor 1C (p57, Kip2)	Ssc.8871.1.S1_at	1.12 q < 0.481	-1.07 q < 1	-1.26 q < 0.731	2.26 q < 0.0000806	1.09 q < 1	-1.04 q < 1
PRDM6	PR domain containing 6	Ssc.5471.1.A1_at	1.06 q < 0.482	-1.07 q < 1	1.02 q < 0.98	1.05 q < 0.808	-1.04 q < 1	1.36 q < 0.00663
BANK1	B-cell scaffold protein with ankyrin repeats 1	Ssc.25700.1.S1_at	-1.08 q < 0.483	-1 q < 1	1.02 q < 0.989	-1.01 q < 0.984	-1.02 q < 1	-1.46 q < 0.0322
FRMD4A	FERM domain containing 4A	Ssc.5434.1.A1_at	-1.06 q < 0.484	-1.38 q < 0.0243	-1.09 q < 0.889	1.04 q < 0.85	1.03 q < 1	1.02 q < 1
HLA-DRB5	major histocompatibility complex, class II, DR beta 5	Ssc.210.6.S1_x_at	-1.09 q < 0.484	1.05 q < 1	-1.02 q < 0.989	1.21 q < 0.49	-1.06 q < 1	-1.81 q < 0.000457
RGS4	regulator of G-protein signaling 4	Ssc.6384.1.S1_at	1.09 q < 0.49	1.11 q < 1	-1.05 q < 0.962	1.04 q < 0.891	-1.09 q < 1	1.49 q < 0.0424
CDC42EP1	CDC42 effector protein (Rho GTPase binding) 1	Ssc.30932.1.S1_at	-1.06 q < 0.492	-1.04 q < 1	-1.33 q < 0.0486	1.02 q < 0.938	-1.02 q < 1	1.02 q < 1
SLC39A4	solute carrier family 39 (zinc transporter), member 4	Ssc.27155.1.S1_at	-1.07 q < 0.492	1.12 q < 1	-1.43 q < 0.0255	-1.05 q < 0.852	-1.08 q < 1	1.02 q < 1
CNN3	calponin 3, acidic	Ssc.4848.1.S1_at	-1.08 q < 0.492	1.05 q < 1	-1.43 q < 0.0424	1.01 q < 0.979	-1.12 q < 1	1.03 q < 1
MBTD1	mbt domain containing 1	Ssc.3152.1.A1_at	1.1 q < 0.494	-1.03 q < 1	-1.05 q < 0.971	1.59 q < 0.0371	1.03 q < 1	1.09 q < 1
ABLIM1	actin binding LIM protein 1	Ssc.19823.1.A1_at	1.15 q < 0.497	-1.12 q < 1	-1.33 q < 0.738	1.86 q < 0.045	1.34 q < 1	1.18 q < 1
ABLIM1	actin binding LIM protein 1	Ssc.10988.1.S1_at	1.08 q < 0.75	-1.13 q < 1	-1.41 q < 0.466	1.71 q < 0.0483	1.14 q < 1	1.22 q < 1
ZNF642	zinc finger protein 642	Ssc.14310.1.A1_at	1.05 q < 0.498	1.02 q < 1	-1.05 q < 0.918	1.28 q < 0.0103	-1.02 q < 1	1.04 q < 1
C6orf124	chromosome 6 open reading frame 124	Ssc.10978.1.S1_at	1.06 q < 0.498	1.04 q < 1	-1.04 q < 0.951	1.3 q < 0.0433	-1.02 q < 1	1.04 q < 1
CENPE	centromere protein E, 312kDa	Ssc.8766.1.A1_at	1.06 q < 0.5	1.45 q < 0.00216	-1.02 q < 0.987	-1 q < 0.989	1.06 q < 1	-1.12 q < 1
SOSTDC1	sclerostin domain containing 1	Ssc.4319.1.A1_at	-1.09 q < 0.5	-1.06 q < 1	-1.02 q < 0.992	1.01 q < 0.99	1.12 q < 1	-1.6 q < 0.0108
ABHD14B	abhydrolase domain containing 14B	Ssc.4942.1.S1_at	1.1 q < 0.505	-1.08 q < 1	-1.04 q < 0.974	1.59 q < 0.0362	1.06 q < 1	1.09 q < 1
RAB3GAP2	RAB3 GTPase activating protein subunit 2 (non-catalytic)	Ssc.6809.1.A1_at	1.07 q < 0.507	-1.05 q < 1	-1.08 q < 0.913	1.5 q < 0.00156	-1.05 q < 1	1.08 q < 1
ICAM1	intercellular adhesion molecule 1	Ssc.11187.1.S1_at	-1.06 q < 0.507	-1.07 q < 1	-1.43 q < 0.00515	1.18 q < 0.35	1.06 q < 1	-1.1 q < 1
C18orf24	spindle and kinetochore associated complex subunit 1	Ssc.27187.1.S1_at	1.05 q < 0.51	1.28 q < 0.0431	1.05 q < 0.932	-1.01 q < 0.955	1.02 q < 1	-1.06 q < 1
KIF11	kinesin family member 11	Ssc.7361.1.A1_at	1.06 q < 0.511	1.39 q < 0.0062	-1.01 q < 0.989	1.05 q < 0.812	-1.07 q < 1	-1.02 q < 1
NR4A3	nuclear receptor subfamily 4, group A, member 3	Ssc.761.1.S1_at	1.08 q < 0.518	-1.02 q < 1	1.02 q < 0.99	1.74 q < 0.000189	-1.2 q < 1	1.02 q < 1
FADS2	fatty acid desaturase 2	Ssc.29000.1.S1_at	1.08 q < 0.518	1.46 q < 0.0431	1.05 q < 0.958	1.06 q < 0.831	-1.14 q < 1	1.02 q < 1
FADS2	fatty acid desaturase 2	Ssc.3039.1.S1_at	1.07 q < 0.589	1.47 q < 0.0492	1.03 q < 0.982	-1 q < 0.999	-1.12 q < 1	1.06 q < 1
LNPEP	leucyl/cystinyl aminopeptidase	Ssc.24418.1.A1_at	-1.06 q < 0.521	1.01 q < 1	-1.05 q < 0.938	-1.31 q < 0.0442	-1.1 q < 1	1.13 q < 1
AMY2B	amylase, alpha 2B (pancreatic)	Ssc.6009.1.S1_a_at	-1.12 q < 0.527	-2.16 q < 0.000589	-1.39 q < 0.453	1.1 q < 0.815	1.44 q < 0.75	1.1 q < 1
AMY2B	amylase, alpha 2B (pancreatic)	Ssc.6009.1.S1_at	-1.06 q < 0.822	-1.94 q < 0.0062	-1.43 q < 0.366	1.04 q < 0.931	1.65 q < 0.146	1.19 q < 1
CWF19L2	CWF19-like 2, cell cycle control (S. pombe)	Ssc.18973.1.A1_at	1.06 q < 0.53	-1.05 q < 1	1.04 q < 0.958	1.35 q < 0.0288	-1.12 q < 1	1.12 q < 1
TAPT1	transmembrane anterior posterior transformation 1	Ssc.10298.1.A1_at	1.07 q < 0.532	-1.04 q < 1	-1.24 q < 0.415	1.44 q < 0.0164	1.31 q < 0.353	-1.04 q < 1
PARK2	Parkinson disease (autosomal recessive, juvenile) 2, parkin	Ssc.30566.1.A1_at	-1.06 q < 0.545	-1.43 q < 0.00291	-1.04 q < 0.958	1.11 q < 0.584	-1.02 q < 1	1.03 q < 1
GOLPH3	golgi phosphoprotein 3 (coat-protein)	Ssc.3795.1.A1_a_at	1.09 q < 0.545	-1.15 q < 1	-1 q < 0.999	1.58 q < 0.0316	-1.02 q < 1	1.16 q < 1
CASP4	caspase 4, apoptosis-related cysteine peptidase	Ssc.12446.1.A1_at	1.07 q < 0.547	1.03 q < 1	1.01 q < 0.997	1.49 q < 0.00714	1.21 q < 1	-1.34 q < 0.136
EDEM3	ER degradation enhancer, mannosidase alpha-like 3	Ssc.21755.1.A1_at	1.09 q < 0.554	-1.09 q < 1	-1.04 q < 0.973	1.52 q < 0.0486	1.03 q < 1	1.11 q < 1
GTF2A1L	general transcription factor IIA, 1-like	Ssc.21237.1.S1_at	1.07 q < 0.556	1.74 q < 0.000115	-1.16 q < 0.763	-1.01 q < 0.977	-1.11 q < 1	1.06 q < 1
SF3A1	splicing factor 3a, subunit 1, 120kDa	Ssc.6690.1.S1_at	1.07 q < 0.557	1.05 q < 1	-1.07 q < 0.933	1.41 q < 0.0336	1.03 q < 1	-1.01 q < 1
NCAPG	non-SMC condensin I complex, subunit G	Ssc.28512.1.S1_at	1.06 q < 0.56	1.39 q < 0.0431	-1.08 q < 0.911	1.07 q < 0.768	1.07 q < 1	-1.09 q < 1
EPHA4	EPH receptor A4	Ssc.22130.1.S1_at	1.11 q < 0.565	1.3 q < 0.849	-1.15 q < 0.903	1.71 q < 0.0324	-1.26 q < 1	1.09 q < 1
LAMA2	laminin, alpha 2	Ssc.10383.1.A1_at	1.04 q < 0.571	-1.03 q < 1	-1.07 q < 0.847	1.05 q < 0.731	1.04 q < 1	1.23 q < 0.0418
MICALL2	MICAL-like 2	Ssc.2688.1.S1_at	1.05 q < 0.572	-1.1 q < 1	-1.12 q < 0.682	1.35 q < 0.00188	1.08 q < 1	1.04 q < 1
ABI1	abl-interactor 1	Ssc.21567.2.S1_at	1.05 q < 0.584	-1.05 q < 1	-1.04 q < 0.951	1.28 q < 0.0364	-1 q < 1	1.08 q < 1
MTR	5-methyltetrahydrofolate-homocysteine methyltransferase	Ssc.19318.1.S1_at	1.06 q < 0.592	-1.12 q < 1	1.09 q < 0.91	1.39 q < 0.0362	-1 q < 1	1.01 q < 1
HIGD1B	HIG1 hypoxia inducible domain family, member 1B	Ssc.13089.1.S1_at	1.07 q < 0.593	1.02 q < 1	-1.31 q < 0.186	1.06 q < 0.815	-1.04 q < 1	1.73 q < 0.0000443
CASP1	caspase 1, apoptosis-related cysteine peptidase (interleukin 1, beta, convertase)	Ssc.16012.1.S1_at	-1.07 q < 0.594	1.02 q < 1	1.08 q < 0.926	1.07 q < 0.818	-1.04 q < 1	-1.6 q < 0.00402
CDKN3	cyclin-dependent kinase inhibitor 3	Ssc.7286.1.S1_at	1.05 q < 0.594	1.33 q < 0.0332	1.05 q < 0.943	1.02 q < 0.919	1.02 q < 1	-1.13 q < 1
ENDOD1	endonuclease domain containing 1	Ssc.12615.1.A1_at	-1.14 q < 0.594	-2.18 q < 0.0309	-1.1 q < 0.954	1.1 q < 0.87	-1.13 q < 1	1.26 q < 1

Gene Name	Gene Description	Probe Set ID	Breed MS-WC	Day 25 MS-WC	Day 45 MS-WC	Day 65 MS-WC	Day 85 MS-WC	Day 105 MS-WC
HNRNPH1	heterogeneous nuclear ribonucleoprotein H1 (H)	Ssc.13767.1.A1_at	1.1 q < 0.597	1.08 q < 1	-1.21 q < 0.799	1.61 q < 0.0463	-1.02 q < 1	1.13 q < 1
CRELD1	cysteine-rich with EGF-like domains 1	Ssc.1501.1.S1_at	1.06 q < 0.606	1.01 q < 1	-1.07 q < 0.918	1.4 q < 0.0216	-1.03 q < 1	1.04 q < 1
GSTT1	glutathione S-transferase theta 1	Ssc.15549.1.A1_at	1.05 q < 0.608	1.13 q < 0.888	-1.1 q < 0.811	1.39 q < 0.00141	-1.11 q < 1	-1.03 q < 1
HMOX2	heme oxygenase (decycling) 2	Ssc.2926.1.S1_at	1.06 q < 0.615	1.08 q < 1	-1.18 q < 0.561	1.45 q < 0.00581	1.05 q < 1	-1.05 q < 1
IFT52	intraflagellar transport 52 homolog (Chlamydomonas)	Ssc.21557.1.S1_at	1.06 q < 0.617	-1.08 q < 1	-1.07 q < 0.913	1.36 q < 0.0288	1.09 q < 1	1.03 q < 1
ANP32A	acidic (leucine-rich) nuclear phosphoprotein 32 family, member A	Ssc.2877.1.A1_at	1.04 q < 0.62	1.37 q < 0.00283	-1.06 q < 0.912	-1.07 q < 0.699	-1.01 q < 1	1.04 q < 1
PLEKHH1	pleckstrin homology domain containing, family H (with MyTH4 domain) member 1	Ssc.10413.1.A1_at	-1.07 q < 0.623	1.11 q < 1	-1.56 q < 0.00437	1.1 q < 0.693	-1.13 q < 1	1.05 q < 1
XPR1	xenotropic and polytropic retrovirus receptor 1	Ssc.13519.1.A1_at	1.05 q < 0.629	1.06 q < 1	1.03 q < 0.966	1.32 q < 0.0439	-1.05 q < 1	-1.07 q < 1
NID2	nidogen 2 (osteonidogen)	Ssc.1053.1.S1_at	-1.12 q < 0.631	-1.09 q < 1	-2.01 q < 0.0178	1.19 q < 0.695	-1.01 q < 1	1.06 q < 1
DPP7	dipeptidyl-peptidase 7	Ssc.1214.1.S1_at	1.06 q < 0.633	-1.28 q < 0.344	-1.1 q < 0.885	1.38 q < 0.0372	1.21 q < 1	1.11 q < 1
TMEM62	transmembrane protein 62	Ssc.1347.1.S1_at	-1.06 q < 0.635	1.01 q < 1	-1.48 q < 0.00672	-1.05 q < 0.839	1.06 q < 1	1.09 q < 1
NBEAL1	neurobeachin-like 1	Ssc.7238.1.A1_at	1.08 q < 0.638	-1.09 q < 1	-1.11 q < 0.913	1.52 q < 0.0487	1.01 q < 1	1.15 q < 1
ANLN	anillin, actin binding protein	Ssc.26757.1.A1_at	1.06 q < 0.643	1.44 q < 0.0489	-1.11 q < 0.878	1.17 q < 0.507	-1.02 q < 1	-1.09 q < 1
EIF3F	eukaryotic translation initiation factor 3, subunit F	Ssc.11792.1.A1_at	-1.05 q < 0.644	1.03 q < 1	-1.34 q < 0.0389	1.09 q < 0.671	1.03 q < 1	-1.1 q < 1
PPP5C	protein phosphatase 5, catalytic subunit	Ssc.18846.1.A1_at	-1.06 q < 0.646	-1.39 q < 0.0403	-1.03 q < 0.975	1.18 q < 0.402	-1.05 q < 1	-1.03 q < 1
SERPINB6	serpin peptidase inhibitor, clade B (ovalbumin), member 6	Ssc.8895.1.S1_at	-1.06 q < 0.647	1.37 q < 0.07	-1.38 q < 0.0398	-1.29 q < 0.134	-1.06 q < 1	1.05 q < 1
GSR	glutathione reductase	Ssc.6474.1.S1_at	1.05 q < 0.647	1.12 q < 1	-1.12 q < 0.796	1.44 q < 0.00397	-1.06 q < 1	-1.06 q < 1
PEBP1	phosphatidylethanolamine binding protein 1	Ssc.11032.1.S1_at	-1.08 q < 0.649	1.2 q < 1	-1.6 q < 0.0498	1.06 q < 0.889	1.04 q < 1	-1.23 q < 1
GMFG	glia maturation factor, gamma	Ssc.15370.1.S1_at	-1.07 q < 0.656	1.17 q < 1	-1.06 q < 0.954	1.01 q < 0.983	-1.02 q < 1	-1.54 q < 0.0241
CDCA3	cell division cycle associated 3	Ssc.18198.1.S1_at	1.06 q < 0.666	1.44 q < 0.0231	-1 q < 0.997	-1.04 q < 0.87	1.02 q < 1	-1.06 q < 1
RING1	ring finger protein 1	Ssc.5967.1.A1_at	1.05 q < 0.676	-1.06 q < 1	1.01 q < 0.994	1.32 q < 0.032	-1.02 q < 1	1.01 q < 1
GAS7	growth arrest-specific 7	Ssc.20467.1.S1_at	-1.05 q < 0.68	-1.43 q < 0.00241	1.01 q < 0.996	1.26 q < 0.107	1.05 q < 1	-1.16 q < 0.839
HLA-DMB	major histocompatibility complex, class II, DM beta	Ssc.11025.1.S1_at	-1.05 q < 0.693	-1.03 q < 1	-1.04 q < 0.956	1.06 q < 0.78	1.07 q < 1	-1.35 q < 0.0468
SAMD12	sterile alpha motif domain containing 12	Ssc.29319.1.A1_at	1.05 q < 0.694	-1.09 q < 1	1.05 q < 0.938	1.38 q < 0.0129	-1.07 q < 1	1.01 q < 1
CASP7	caspase 7, apoptosis-related cysteine peptidase	Ssc.3773.1.S1_at	-1.08 q < 0.696	-1.22 q < 1	-1.82 q < 0.0115	1.15 q < 0.712	1.36 q < 1	-1.06 q < 1
METTL9	methyltransferase like 9	Ssc.20160.1.S1_at	1.05 q < 0.697	1.04 q < 1	1.41 q < 0.0343	-1.28 q < 0.166	-1 q < 1	1.14 q < 1
DOCK1	dedicator of cytokinesis 1	Ssc.7408.1.A1_at	1.07 q < 0.698	-1.05 q < 1	-1.08 q < 0.94	1.58 q < 0.0232	-1.01 q < 1	1.02 q < 1
HPS3	Hermansky-Pudlak syndrome 3	Ssc.8819.1.A1_at	-1.05 q < 0.699	-1 q < 1	-1.44 q < 0.0223	1.12 q < 0.611	-1.05 q < 1	1.03 q < 1
ROR1	receptor tyrosine kinase-like orphan receptor 1	Ssc.29681.1.A1_at	1.05 q < 0.702	-1.18 q < 0.671	-1.01 q < 0.99	1.04 q < 0.851	1.06 q < 1	1.35 q < 0.0299
ROR1	RAR-related orphan receptor A	Ssc.29681.1.A1_at	1.05 q < 0.702	-1.18 q < 0.671	-1.01 q < 0.99	1.04 q < 0.851	1.06 q < 1	1.35 q < 0.0299
C13orf16	chromosome 13 open reading frame 16	Ssc.2130.1.S1_at	-1.04 q < 0.705	-1.05 q < 1	-1.35 q < 0.0175	1.06 q < 0.77	-1.04 q < 1	1.13 q < 1
ATIC	5-aminoimidazole-4-carboxamide ribonucleotide formyltransferase/IMP cyclohydrolase	Ssc.27454.1.S1_at	1.05 q < 0.706	1.46 q < 0.0214	1.02 q < 0.988	-1.11 q < 0.67	-1.01 q < 1	-1.02 q < 1
C11orf17	chromosome 11 open reading frame 17	Ssc.11695.1.S1_at	-1.05 q < 0.707	1.03 q < 1	-1.35 q < 0.0367	1.13 q < 0.529	-1.06 q < 1	-1.01 q < 1
C2	complement component 2	Ssc.19364.1.S1_at	-1.05 q < 0.711	1.07 q < 1	-1.12 q < 0.853	1.13 q < 0.595	1.04 q < 1	-1.45 q < 0.0191
WNK1	WNK lysine deficient protein kinase 1	Ssc.5955.1.A1_at	-1.04 q < 0.714	1.22 q < 0.139	-1.26 q < 0.0428	-1.05 q < 0.76	-1.08 q < 1	-1.02 q < 1
C16orf33	small nuclear ribonucleoprotein 25kDa (U11/U12)	Ssc.10863.1.A1_at	1.04 q < 0.715	1.42 q < 0.00191	-1.03 q < 0.967	-1.11 q < 0.546	1.06 q < 1	-1.08 q < 1
BF710262	-	Ssc.9331.1.A1_at	-1.05 q < 0.716	-1.03 q < 1	-1.03 q < 0.973	-1.49 q < 0.00269	-1.07 q < 1	1.35 q < 0.0727
HLA-DRB3	major histocompatibility complex, class II, DR beta 3	Ssc.30586.1.A1_at	1.04 q < 0.719	-1.02 q < 1	-1.08 q < 0.886	1.3 q < 0.0277	1 q < 1	1.02 q < 1
CD74	CD74 molecule, major histocompatibility complex, class II invariant chain	Ssc.6222.1.S1_a_at	-1.08 q < 0.721	1.44 q < 0.323	-1.21 q < 0.799	-1.01 q < 0.991	1.06 q < 1	-1.79 q < 0.0075
UBD	ubiquitin D	Ssc.21582.1.S1_at	-1.07 q < 0.724	-1.03 q < 1	1.11 q < 0.915	1.09 q < 0.817	-1 q < 1	-1.65 q < 0.0243
C13orf15	chromosome 13 open reading frame 15	Ssc.9553.1.A1_s_at	-1.04 q < 0.729	-1.04 q < 1	-1.09 q < 0.824	1.34 q < 0.004	-1.27 q < 0.0672	-1.1 q < 1
C13orf15	chromosome 13 open reading frame 15	Ssc.23963.1.S1_at	1.06 q < 0.833	1.41 q < 0.53	-1.17 q < 0.884	1.73 q < 0.0329	-1.45 q < 0.744	-1.07 q < 1
SRPX	sushi-repeat-containing protein, X-linked	Ssc.897.1.S1_at	1.07 q < 0.73	-1.6 q < 0.0738	1.67 q < 0.0307	1.32 q < 0.353	1.2 q < 1	-1.15 q < 1
UBE4A	ubiquitination factor E4A (UFD2 homolog, yeast)	Ssc.8329.1.A1_at	1.04 q < 0.734	1.01 q < 1	-1.18 q < 0.529	1.36 q < 0.0242	1.04 q < 1	1.02 q < 1

Gene Name	Gene Description	Probe Set ID	Breed MS-WC	Day 25 MS-WC	Day 45 MS-WC	Day 65 MS-WC	Day 85 MS-WC	Day 105 MS-WC
SLC3A1	solute carrier family 3 (cystine, dibasic and neutral amino acid transporters, activator of cystine, dibasic and neutral amino acid transport), member 1	Ssc.2441.2.A1_at	-1.05 q < 0.738	1.25 q < 0.579	-1.57 q < 0.00418	-1.01 q < 0.985	-1.25 q < 1	1.22 q < 0.814
CCDC120	coiled-coil domain containing 120	Ssc.17126.2.S1_at	1.05 q < 0.746	-1.12 q < 1	-1.05 q < 0.952	1.48 q < 0.0165	1.03 q < 1	-1 q < 1
MCM2	minichromosome maintenance complex component 2	Ssc.21350.1.S1_at	1.03 q < 0.746	1.33 q < 0.0091	-1.12 q < 0.703	1.03 q < 0.877	1.02 q < 1	-1.06 q < 1
MCM2	minichromosome maintenance complex component 2	Ssc.1408.2.S1_at	1.02 q < 0.908	1.37 q < 0.0152	-1.06 q < 0.928	-1.02 q < 0.925	1.03 q < 1	-1.18 q < 0.717
MCM2	minichromosome maintenance complex component 7	Ssc.21350.1.S1_at	1.03 q < 0.746	1.33 q < 0.0091	-1.12 q < 0.703	1.03 q < 0.877	1.02 q < 1	-1.06 q < 1
MCM2	minichromosome maintenance complex component 7	Ssc.1408.2.S1_at	1.02 q < 0.908	1.37 q < 0.0152	-1.06 q < 0.928	-1.02 q < 0.925	1.03 q < 1	-1.18 q < 0.717
BRE	brain and reproductive organ-expressed (TNFRSF1A modulator)	Ssc.9240.3.S1_a_at	1.05 q < 0.751	-1 q < 1	-1.08 q < 0.913	1.44 q < 0.0106	1 q < 1	-1.06 q < 1
UTX	lysine (K)-specific demethylase 6A	Ssc.8310.1.A1_at	1.05 q < 0.759	-1 q < 1	-1.16 q < 0.772	1.43 q < 0.034	-1.06 q < 1	1.1 q < 1
PPFIA2	protein tyrosine phosphatase, receptor type, f polypeptide (PTPRF), interacting protein (liprin), alpha 2	Ssc.5203.1.A1_at	1.04 q < 0.762	-1.06 q < 1	1.01 q < 0.989	1.35 q < 0.0219	-1.09 q < 1	1.02 q < 1
DLGAP5	discs, large (Drosophila) homolog-associated protein 5	Ssc.19504.1.A1_at	1.05 q < 0.765	1.57 q < 0.00283	-1.04 q < 0.958	-1.05 q < 0.86	-1.03 q < 1	-1.1 q < 1
RABEP1	rabaptin, RAB GTPase binding effector protein 1	Ssc.21275.1.A1_at	1.04 q < 0.777	-1.03 q < 1	-1.13 q < 0.719	1.31 q < 0.0424	1.03 q < 1	1.04 q < 1
EZH2	enhancer of zeste homolog 2 (Drosophila)	Ssc.5073.1.A1_at	1.04 q < 0.777	1.36 q < 0.0169	-1.04 q < 0.951	-1 q < 0.998	-1.03 q < 1	-1.06 q < 1
MRAS	muscle RAS oncogene homolog	Ssc.24201.1.A1_at	1.03 q < 0.779	-1.04 q < 1	-1.02 q < 0.975	1.25 q < 0.0481	-1.03 q < 1	1.02 q < 1
SH3BP5	SH3-domain binding protein 5 (BTK-associated)	Ssc.25282.1.S1_at	-1.06 q < 0.779	1.07 q < 1	-1.63 q < 0.0165	-1.06 q < 0.86	-1.08 q < 1	1.32 q < 0.592
TBCEL	tubulin folding cofactor E-like	Ssc.23894.1.A1_at	1.03 q < 0.78	-1.04 q < 1	1.08 q < 0.835	1.24 q < 0.0415	-1.11 q < 1	-1 q < 1
ACO1	aconitase 1, soluble	Ssc.26682.1.A1_at	-1.08 q < 0.78	1.27 q < 0.991	-1.83 q < 0.0232	1.23 q < 0.589	-1.02 q < 1	-1.21 q < 1
ZFAND2A	zinc finger, AN1-type domain 2A	Ssc.5082.1.A1_at	1.04 q < 0.789	-1.02 q < 1	-1.19 q < 0.586	1.42 q < 0.0218	-1.01 q < 1	1.08 q < 1
LRRTM4	leucine rich repeat transmembrane neuronal 4	Ssc.29330.1.S1_at	-1.04 q < 0.796	-1.4 q < 0.0114	1.07 q < 0.91	1.09 q < 0.661	-1.02 q < 1	1.02 q < 1
GPNMB	glycoprotein (transmembrane) nmb	Ssc.18572.2.S1_at	-1.03 q < 0.804	-1.06 q < 1	1.01 q < 0.988	-1.34 q < 0.0126	1.06 q < 1	1.13 q < 1
GPNMB	glycoprotein (transmembrane) nmb	Ssc.18572.1.S1_at	-1.05 q < 0.865	-1 q < 1	1 q < 1	-2 q < 0.00295	1.42 q < 0.885	1.09 q < 1
CAPZA2	capping protein (actin filament) muscle Z-line, alpha 2	Ssc.4047.3.S1_at	1.03 q < 0.812	-1.04 q < 1	-1.19 q < 0.404	1.33 q < 0.0168	1.03 q < 1	1.05 q < 1
CDK6	cyclin-dependent kinase 6	Ssc.9474.1.A1_at	1.03 q < 0.814	-1.12 q < 0.972	-1 q < 0.997	1.29 q < 0.0397	-1.03 q < 1	1.05 q < 1
ANKRD52	ankyrin repeat domain 52	Ssc.19594.1.S1_at	1.03 q < 0.82	-1.05 q < 1	-1.08 q < 0.883	1.29 q < 0.0448	-1.01 q < 1	1.04 q < 1
CYP3A7	cytochrome P450, family 3, subfamily A, polypeptide 7	Ssc.203.1.S1_s_at	1.06 q < 0.829	-1.04 q < 1	1.89 q < 0.0125	1.02 q < 0.968	1.18 q < 1	-1.6 q < 0.165
SLC26A11	solute carrier family 26, member 11	Ssc.18186.1.S1_at	-1.05 q < 0.83	1.11 q < 1	-1.77 q < 0.000807	1.35 q < 0.183	-1.12 q < 1	1.05 q < 1
SLC41A1	solute carrier family 41, member 1	Ssc.12083.3.A1_at	1.03 q < 0.833	-1.06 q < 1	1.02 q < 0.981	1.3 q < 0.0393	-1.09 q < 1	1 q < 1
MAP1B	microtubule-associated protein 1B	Ssc.6093.1.S1_at	1.04 q < 0.835	1.57 q < 0.00216	-1.02 q < 0.989	-1.12 q < 0.638	1.04 q < 1	-1.19 q < 0.938
MAP1B	microtubule-associated protein 1B	Ssc.6093.2.A1_at	1.04 q < 0.882	1.61 q < 0.00899	-1.07 q < 0.944	-1.1 q < 0.751	1.06 q < 1	-1.23 q < 0.87
DAO	D-amino-acid oxidase	Ssc.8031.1.A1_at	-1.04 q < 0.838	1.14 q < 1	1.29 q < 0.217	-1.47 q < 0.00887	-1.11 q < 1	-1.07 q < 1
DAO	amiloride binding protein 1 (amine oxidase (copper-containing))	Ssc.8031.1.A1_at	-1.04 q < 0.838	1.14 q < 1	1.29 q < 0.217	-1.47 q < 0.00887	-1.11 q < 1	-1.07 q < 1
PNKD	paroxysmal nonkinesigenic dyskinesia	Ssc.3681.1.S1_at	1.05 q < 0.851	1.29 q < 0.805	-1.36 q < 0.433	1.62 q < 0.0364	-1.17 q < 1	-1.03 q < 1
ZNF655	zinc finger protein 655	Ssc.2910.1.A1_at	1.05 q < 0.853	-1.22 q < 0.972	-1.18 q < 0.828	1.57 q < 0.0398	1.25 q < 1	-1.08 q < 1
TNNI3K	TNNI3 interacting kinase	Ssc.26763.1.S1_at	1.03 q < 0.856	-1.04 q < 1	1 q < 0.997	1.34 q < 0.014	-1.05 q < 1	-1.08 q < 1
SLCO4C1	solute carrier organic anion transporter family, member 4C1	Ssc.27370.1.S1_at	1.06 q < 0.861	1.04 q < 1	-1.47 q < 0.488	1.86 q < 0.0486	-1.07 q < 1	1.12 q < 1
CN166389	-	Ssc.27411.1.A1_at	1.03 q < 0.862	-1.55 q < 0.00241	-1.04 q < 0.964	1.23 q < 0.298	1.22 q < 1	1.26 q < 0.46
LIF	leukemia inhibitory factor (cholinergic differentiation factor)	Ssc.21256.1.S2_at	1.07 q < 0.863	1.06 q < 1	-1.02 q < 0.996	2.19 q < 0.00883	-1.45 q < 1	-1.14 q < 1
PRDX1	peroxiredoxin 1	Ssc.5380.1.S1_at	-1.05 q < 0.863	1.72 q < 0.0261	-1.3 q < 0.593	1.01 q < 0.981	-1.35 q < 1	-1.26 q < 1
HECW2	HECT, C2 and WW domain containing E3 ubiquitin protein ligase 2	Ssc.1451.1.S1_at	1.03 q < 0.868	1.11 q < 1	-1.24 q < 0.448	-1.04 q < 0.901	-1.11 q < 1	1.5 q < 0.0112
UBL3	ubiquitin-like 3	Ssc.6872.2.S1_at	1.04 q < 0.869	-1.04 q < 1	-1.21 q < 0.734	1.52 q < 0.0401	-1.07 q < 1	1.07 q < 1
SEPW1	selenoprotein W, 1	Ssc.5062.1.S1_at	-1.03 q < 0.871	1.33 q < 0.178	-1.6 q < 0.00114	-1.02 q < 0.939	-1.07 q < 1	1.12 q < 1
GYG1	glycogenin 1	Ssc.4204.1.S1_at	1.05 q < 0.879	2.05 q < 0.00235	-1.32 q < 0.63	1.13 q < 0.754	-1.14 q < 1	-1.22 q < 1
SPARCL1	SPARC-like 1 (hevin)	Ssc.6531.1.A1_at	-1.05 q < 0.885	1.04 q < 1	-2.19 q < 0.00112	1.33 q < 0.431	-1.24 q < 1	1.54 q < 0.307
KIF20A	kinesin family member 20A	Ssc.3227.1.A1_a_at	1.02 q < 0.887	1.35 q < 0.0246	-1.07 q < 0.913	-1.03 q < 0.902	-1.03 q < 1	-1.06 q < 1

Gene Name	Gene Description	Probe Set ID	Breed MS-WC	Day 25 MS-WC	Day 45 MS-WC	Day 65 MS-WC	Day 85 MS-WC	Day 105 MS-WC
CC2D2A	coiled-coil and C2 domain containing 2A	Ssc.18099.1.A1_at	1.02 q < 0.892	-1.02 q < 1	-1.08 q < 0.878	1.32 q < 0.0115	-1.09 q < 1	1 q < 1
Q8N8A0	-	Ssc.10548.1.A1_at	1.03 q < 0.897	1.02 q < 1	-1.1 q < 0.885	-1.01 q < 0.976	-1.14 q < 1	1.41 q < 0.0335
FRRS1	ferric-chelate reductase 1	Ssc.25253.1.S1_at	-1.04 q < 0.901	-1.71 q < 0.0103	-1.02 q < 0.99	1.17 q < 0.618	1.24 q < 1	1.01 q < 1
PGD	phosphogluconate dehydrogenase	Ssc.16347.1.S1_at	-1.03 q < 0.902	1.61 q < 0.0243	-1.52 q < 0.0676	-1.12 q < 0.719	-1.03 q < 1	-1.09 q < 1
ZFP36	zinc finger protein 36, C3H type, homolog (mouse)	Ssc.3411.1.A1_at	1.03 q < 0.902	-1.09 q < 1	1.02 q < 0.992	1.57 q < 0.0243	-1.08 q < 1	-1.15 q < 1
OSBPL6	oxysterol binding protein-like 6	Ssc.9483.1.A1_s_at	1.03 q < 0.908	1.04 q < 1	-1.66 q < 0.00161	1.14 q < 0.603	1.2 q < 1	1.34 q < 0.283
CCL5	chemokine (C-C motif) ligand 5	Ssc.22030.1.S1_at	-1.02 q < 0.917	1.01 q < 1	1.04 q < 0.946	1.09 q < 0.654	1.07 q < 1	-1.34 q < 0.0252
NGFRAP1	nerve growth factor receptor (TNFRSF16) associated protein 1	Ssc.7039.1.A1_at	1.04 q < 0.92	1.27 q < 1	-2.06 q < 0.00351	1.28 q < 0.505	1.52 q < 0.547	1 q < 1
TNN	tenascin N	Ssc.5738.1.A1_at	1.02 q < 0.92	-1.14 q < 0.911	1.36 q < 0.015	1 q < 0.988	-1.07 q < 1	-1.03 q < 1
UBE2T	ubiquitin-conjugating enzyme E2T (putative)	Ssc.19169.1.S1_at	1.02 q < 0.92	1.37 q < 0.0425	-1.06 q < 0.938	-1.04 q < 0.854	-1.07 q < 1	-1.06 q < 1
F5	coagulation factor V (proaccelerin, labile factor)	Ssc.15822.1.S1_at	1.03 q < 0.923	1.7 q < 0.0328	-1.07 q < 0.956	-1.12 q < 0.751	-1.01 q < 1	-1.21 q < 1
ANKRD17	ankyrin repeat domain 17	Ssc.30178.1.A1_at	1.02 q < 0.923	-1.1 q < 1	1 q < 0.999	1.36 q < 0.0448	-1.1 q < 1	-1.02 q < 1
NCL	nucleolin	Ssc.2695.1.S1_at	-1.03 q < 0.925	-1.06 q < 1	-2.03 q < 0.00454	1.68 q < 0.0625	1.15 q < 1	-1.07 q < 1
HNRNPL	heterogeneous nuclear ribonucleoprotein L	Ssc.16767.1.S1_at	1.03 q < 0.927	-1.53 q < 0.31	-1.54 q < 0.221	2.99 q < 0.000000247	1.01 q < 1	-1.08 q < 1
TPX2	TPX2, microtubule-associated, homolog (Xenopus laevis)	Ssc.12104.1.S1_at	1.02 q < 0.931	1.52 q < 0.0178	-1.12 q < 0.879	-1.07 q < 0.796	-1.04 q < 1	-1.1 q < 1
GLRX3	glutaredoxin 3	Ssc.20426.1.S1_at	-1.02 q < 0.932	1.44 q < 0.0243	-1.23 q < 0.436	-1.09 q < 0.734	-1.01 q < 1	-1.17 q < 0.99
GRM7	glutamate receptor, metabotropic 7	Ssc.25253.2.A1_at	-1.02 q < 0.932	-1.68 q < 0.0114	1.01 q < 0.997	1.2 q < 0.532	1.15 q < 1	1.07 q < 1
USO1	USO1 vesicle docking protein homolog (yeast)	Ssc.30740.1.A1_at	1.01 q < 0.933	-1.16 q < 0.554	-1.01 q < 0.993	1.28 q < 0.0323	-1.01 q < 1	-1.01 q < 1
DBI	diazepam binding inhibitor (GABA receptor modulator, acyl-CoA binding protein)	Ssc.396.1.S1_a_at	1.03 q < 0.935	1.93 q < 0.00208	-1.36 q < 0.466	-1.09 q < 0.822	-1.14 q < 1	1 q < 1
DBI	translocator protein (18kDa)	Ssc.396.1.S1_a_at	1.03 q < 0.935	1.93 q < 0.00208	-1.36 q < 0.466	-1.09 q < 0.822	-1.14 q < 1	1 q < 1
LENG8	leukocyte receptor cluster (LRC) member 8	Ssc.21653.1.S1_at	1.02 q < 0.935	-1.05 q < 1	-1.29 q < 0.222	1.44 q < 0.017	-1.01 q < 1	1.05 q < 1
LENG8	leukocyte receptor cluster (LRC) member 8	Ssc.21653.2.S1_a_at	1.01 q < 0.98	1 q < 1	-1.39 q < 0.0485	1.36 q < 0.0582	1 q < 1	1.06 q < 1
AP2B1	adaptor-related protein complex 2, beta 1 subunit	Ssc.6040.2.S1_at	-1.01 q < 0.945	-1.12 q < 0.683	-1 q < 0.996	-1.22 q < 0.0382	1.15 q < 0.722	1.14 q < 0.528
TUSC4	nitrogen permease regulator-like 2 (S. cerevisiae)	Ssc.3460.1.S1_at	1.02 q < 0.95	1.1 q < 1	-1.25 q < 0.356	1.38 q < 0.044	-1.03 q < 1	-1.09 q < 1
SRGAP2	SLIT-ROBO Rho GTPase activating protein 2	Ssc.8412.1.A1_at	1.01 q < 0.95	-1.08 q < 1	-1.05 q < 0.943	1.33 q < 0.0398	-1.04 q < 1	-1.06 q < 1
SRGAP2	SLIT-ROBO Rho GTPase activating protein 3	Ssc.8412.1.A1_at	1.01 q < 0.95	-1.08 q < 1	-1.05 q < 0.943	1.33 q < 0.0398	-1.04 q < 1	-1.06 q < 1
MAFF	v-maf musculoaponeurotic fibrosarcoma oncogene homolog F (avian)	Ssc.14224.1.A1_at	1.02 q < 0.951	-1.08 q < 1	-1.07 q < 0.94	1.59 q < 0.00273	-1.14 q < 1	-1.11 q < 1
NP_997182	-	Ssc.7217.1.A1_at	-1.01 q < 0.954	-1.35 q < 0.0168	1.01 q < 0.996	1.04 q < 0.833	1.04 q < 1	1.17 q < 0.752
WWC3	WWC family member 3	Ssc.23073.1.A1_at	1.02 q < 0.955	1.06 q < 1	-1.45 q < 0.273	1.62 q < 0.0493	-1.09 q < 1	1.02 q < 1
GCLC	glutamate-cysteine ligase, catalytic subunit	Ssc.2166.1.A1_at	1.02 q < 0.955	1.03 q < 1	-1.19 q < 0.718	1.59 q < 0.0052	-1.32 q < 0.622	1.04 q < 1
MCM5	minichromosome maintenance complex component 5	Ssc.16649.1.S1_a_at	1.01 q < 0.955	1.41 q < 0.0132	-1.08 q < 0.895	-1.04 q < 0.864	-1.03 q < 1	-1.13 q < 1
TAF7L	TAF7-like RNA polymerase II, TATA box binding protein (TBP)-associated factor, 50kDa	Ssc.19829.1.S1_at	1.01 q < 0.959	-1.01 q < 1	-1.14 q < 0.827	1.52 q < 0.0068	-1.26 q < 0.83	1.02 q < 1
SPC24	SPC24, NDC80 kinetochore complex component, homolog (S. cerevisiae)	Ssc.19196.1.A1_at	1.01 q < 0.959	1.29 q < 0.0467	-1.04 q < 0.951	-1.01 q < 0.95	-1.05 q < 1	-1.12 q < 1
RYR2	ryanodine receptor 2 (cardiac)	Ssc.25615.1.A1_at	-1.02 q < 0.965	-2.1 q < 0.0313	-1.03 q < 0.992	1.45 q < 0.389	1.15 q < 1	1.14 q < 1
DDHD2	DDHD domain containing 2	Ssc.1796.1.A1_at	1.02 q < 0.967	-1.79 q < 0.0149	1.02 q < 0.991	1.25 q < 0.503	-1.04 q < 1	1.58 q < 0.12
RNF157	ring finger protein 157	Ssc.25948.1.S1_at	-1.01 q < 0.967	1.45 q < 0.00959	-1.1 q < 0.879	-1.03 q < 0.924	-1.16 q < 1	-1.17 q < 0.923
CCNB1	cyclin B1	Ssc.14243.1.S1_at	1.01 q < 0.968	1.61 q < 0.0185	-1.12 q < 0.897	-1.04 q < 0.913	-1.09 q < 1	-1.18 q < 1
EIF4EBP3	eukaryotic translation initiation factor 4E binding protein 3	Ssc.26393.1.S1_at	-1.01 q < 0.97	1.02 q < 1	1.09 q < 0.777	-1.24 q < 0.0349	1.03 q < 1	1.06 q < 1
COX17	COX17 cytochrome c oxidase assembly homolog (S. cerevisiae)	Ssc.16607.1.A1_at	1.01 q < 0.976	1.48 q < 0.0000507	-1.26 q < 0.0703	-1.08 q < 0.627	1.03 q < 1	-1.08 q < 1
PROM1	prominin 1	Ssc.4065.1.A1_at	1.01 q < 0.976	1.3 q < 0.00543	-1.02 q < 0.977	-1.21 q < 0.0796	-1.21 q < 0.187	1.18 q < 0.3
DPP6	dipeptidyl-peptidase 6	Ssc.9815.1.A1_at	1.01 q < 0.978	-1.18 q < 0.869	-1.16 q < 0.752	1.52 q < 0.00581	-1.07 q < 1	1.01 q < 1
NOS2A	nitric oxide synthase 2, inducible	Ssc.16117.1.S1_at	1.01 q < 0.982	-1.14 q < 1	-1.21 q < 0.492	1.49 q < 0.00404	-1.05 q < 1	-1 q < 1
Q6ZVE2	-	Ssc.17403.1.S1_at	-1.01 q < 0.984	-1.81 q < 0.000086	1.01 q < 0.997	1.28 q < 0.253	1.08 q < 1	1.26 q < 0.676
PODXL	podocalyxin-like	Ssc.26067.1.S1_at	-1.01 q < 0.994	2.06 q < 0.0457	-1.32 q < 0.789	-1.14 q < 0.813	-1.22 q < 1	-1.16 q < 1
LAPTM4B	lysosomal protein transmembrane 4 beta	Ssc.13846.1.A1_at	-1 q < 0.996	1.45 q < 0.372	-1.17 q < 0.878	-1.73 q < 0.0223	1.11 q < 1	1.24 q < 1
